# Genetic variation in *CADM2* as a link between psychological traits and obesity

**DOI:** 10.1038/s41598-019-43861-9

**Published:** 2019-05-14

**Authors:** Julia Morris, Mark E. S. Bailey, Damiano Baldassarre, Breda Cullen, Ulf de Faire, Amy Ferguson, Bruna Gigante, Philippe Giral, Anuj Goel, Nicholas Graham, Anders Hamsten, Steve E. Humphries, Keira J. A. Johnston, Donald M. Lyall, Laura M. Lyall, Bengt Sennblad, Angela Silveira, Andries J. Smit, Elena Tremoli, Fabrizio Veglia, Joey Ward, Hugh Watkins, Daniel J. Smith, Rona J. Strawbridge

**Affiliations:** 1 Institute of Health and Wellbeing, University of Glasgow, Glasgow, UK; 2 School of Life Sciences, College of Medical, Veterinary and Life Sciences, University of Glasgow, Glasgow, UK; 3 Department of Medical Biotechnology and Translational Medicine, Università degli Studi di Milano, Milan, Italy; 4 Centro Cardiologico Monzino, IRCCS, Milan, Italy; 5 Unit of Cardiovascular Epidemiology, Institute of Environmental Medicine, Karolinska Institutet, Stockholm, Sweden; 6 Division of Cardiovascular Medicine, Department of Clinical Sciences, Danderyd University Hospital, Stockholm, Sweden; 7 Assistance Publique-Hopitaux de Paris, Service Endocrinologie-Metabolisme, Groupe Hôpitalier Pitie-Salpetriere, Unités de Prévention Cardiovasculaire, Paris, France; 8 Division of Cardiovascular Medicine, Radcliffe Department of Medicine, University of Oxford, Oxford, UK; 9 Wellcome Centre for Human Genetics, University of Oxford, Oxford, UK; 10 Cardiovascular Medicine Unit, Department of Medicine Solna, Karolinska Institutet, Stockholm, Sweden; 11 Centre for Cardiovascular Genetics, Institute Cardiovascular Science, University College London, London, UK; 12 Division of Psychiatry, College of Medicine, University of Edinburgh, Edinburgh, UK; 13 Department of Cell and Molecular Biology, National Bioinformatics Infrastructure Sweden, Science for Life Laboratory, Uppsala University, Uppsala, Sweden; 14 Department of Medicine, University Medical Center Groningen and University of Groningen, Groningen, The Netherlands; 15 Dipartimento di Scienze Farmacologiche e Biomolecolari, Università di Milano, Milan, Italy

**Keywords:** Cardiovascular diseases, Endocrine system and metabolic diseases, Psychiatric disorders, Genetics research

## Abstract

*CADM2* has been associated with a range of behavioural and metabolic traits, including physical activity, risk-taking, educational attainment, alcohol and cannabis use and obesity. Here, we set out to determine whether *CADM2* contributes to mechanisms shared between mental and physical health disorders. We assessed genetic variants in the *CADM2* locus for association with phenotypes in the UK Biobank, IMPROVE, PROCARDIS and SCARFSHEEP studies, before performing meta-analyses. A wide range of metabolic phenotypes were meta-analysed. Psychological phenotypes analysed in UK Biobank only were major depressive disorder, generalised anxiety disorder, bipolar disorder, neuroticism, mood instability and risk-taking behaviour. In UK Biobank, four, 88 and 172 genetic variants were significantly (p < 1 × 10^−5^) associated with neuroticism, mood instability and risk-taking respectively. In meta-analyses of 4 cohorts, we identified 362, 63 and 11 genetic variants significantly (p < 1 × 10^−5^) associated with BMI, SBP and CRP respectively. Genetic effects on BMI, CRP and risk-taking were all positively correlated, and were consistently inversely correlated with genetic effects on SBP, mood instability and neuroticism. Conditional analyses suggested an overlap in the signals for physical and psychological traits. Many significant variants had genotype-specific effects on *CADM2* expression levels in adult brain and adipose tissues. *CADM2* variants influence a wide range of both psychological and metabolic traits, suggesting common biological mechanisms across phenotypes via regulation of *CADM2* expression levels in adipose tissue. Functional studies of *CADM2* are required to fully understand mechanisms connecting mental and physical health conditions.

## Introduction

The link between mental illness and poor physical health is well-established but reasons for this are poorly understood. Patients with severe psychiatric illness have 2–3 times higher rate of metabolic syndrome than the general population worldwide, likely due to a combination of factors including the effects of psychotropic drugs, negative health behaviours, hormone dysregulation and shared genetic risk factors^1,2^. A number of potential shared pathways between mood disorders and cardiometabolic disease have been suggested, including abnormal circadian rhythms, hypothalamic-pituitary-adrenal (HPA) axis dysfunction and inflammation. However, the molecular mechanisms of these pathways are poorly understood.

Single nucleotide polymorphisms (SNPs) in the locus encoding the synaptic cell adhesion molecule 2 (*CADM2*) on chromosome 3 have been associated with a number of psychological traits, including educational attainment^3^, alcohol consumption^4^, cannabis use^5^, physical activity habits^6^, risk-taking behaviour^7,8^, attention-deficit/hyperactivity disorder^9^ and obesity^10^. Several lines of evidence point to *CADM2* being the gene through which SNPs are having their effects, including genotype-specific effects on CADM2 mRNA expression levels^7,8^, C*ADM2* being predominantly expressed in the brain, and *cadm2* knockout models demonstrating relevant phenotypes. Specifically, *cadm2*-knockout mice have reduced adiposity, reduced systemic glucose levels, improved insulin sensitivity, increased locomotor activity, increased energy expenditure rate and raised core body temperature, suggesting an important role in systemic energy homeostasis^11^.

We set out to systematically evaluate the relationship between *CADM2* SNPs and psychological and physical traits, and assess whether there is evidence for distinct signals influencing metabolic versus psychological traits.

## Materials and Methods

### CADM2 locus

We defined the *CADM2* locus as the *CADM2* gene plus 250 kb up and downstream (Chromosome 3:84758000-86374000, UCSC genome browser, https://genome-euro.ucsc.edu/).

### Study cohorts

High CVD risk population: IMPROVE is a cohort of individuals with no symptoms or history of cardiovascular disease, but with a least three classic risk factors (namely any combination of the following: family history of CVD, type 2 diabetes, smoking, hypertension, dyslipidaemia, male sex or women at least 5 years post-menopause)^12^. In brief, 3,711 participants were recruited from 7 centres across 5 European countries (Finland, Sweden, the Netherlands, France and Italy) between January 2004 and June 2005. Participants completed a structured medical history and lifestyle questionnaire at baseline, as well as standard biochemical tests and genotyping. Ethics committee approval was granted by the Institutional review board (IRB) at each recruitment centre: Karolinska Institutet, Stockholm, Sweden; University of Milan, Milan, Italy; University of Kuopio and Kuopio Research Institute of Exercise Medicine, Kupio, Finland; University Hospital Groningen, Groningen, The Netherlands; University of Perugia, Perugia, Italy; Groupe Hôpital Pitie-Salpetriere, Paris, France. Informed consent was provided by all participants. The study was conducted in accordance with the Helsinki Declaration.

Young CVD case-control cohort: SCARFSHEEP is a case-control cohort of Swedish participants (N = 2,513) recruited in Stockholm^13,14^. Cases were those with a first myocardial infarction before 60 years of age. Controls were age and sex-matched from the general population of the same county. Standard biochemical phenotyping was available for all participants. Approval was granted by the Karolinska Hospital and Karolinska Institutet Ethics Committees (for SCARF and SHEEP respectively). Informed consent was provided by all participants. The study conducted in accordance with the Helsinki Declaration.

CVD case-control cohort: PROCARDIS is a case-control cohort^15^, where cases (n = 5,688) were diagnosed with coronary artery disease before 66 years and controls (n = 2,310) are unrelated participants without coronary artery disease at 66 years. Participants were recruited from 4 centres across 4 European countries (Sweden, the UK, Germany and Italy). Participants completed a questionnaire at baseline. Standard biochemical phenotyping was available for all participants. Ethics Committee approval was granted by the IRB at each recruitment centre: the Regional Ethics Review Board at Karolinska Institutet, Stockholm in Sweden, the IRB at the University of Munster, Munster, in Germany, the IRB at the Mario Negri Institute, Milano in Italy and the IRB at the University of Oxford, Oxford, United Kingdom. Informed consent was provided by all participants. The study conducted in accordance with the Helsinki Declaration.

General population cohort: UK Biobank is a cohort of over 500,000 participants aged 40–69 at baseline^16^. Participants were recruited from 22 centres across the UK between 2006 and 2010. Participants completed a wide variety of baseline questionnaires and assessments. For consistency with the other cohorts, only white British participants were included. All participants provided informed consent. This study was carried out under the generic approval from the NHS National Research Ethics Service (approval letter dated 13 May 2016, Ref 16/NW/0274) and under UK Biobank applications #6553 (PI Daniel Smith) and #17689 (PI Donald Lyall). The study was conducted in accordance with the Helsinki Declaration.

### Genetic data

IMPROVE participants were genotyped using both Illumina Cardio-Metabo^17^ and Immuno^18^ arrays at the SNP&SEQ Technology Platform in Uppsala. SCARFSHEEP were genotyped using the Illumina Cardio-Metabo chip^17^ at the SNP&SEQ Technology Platform in Uppsala. For both cohorts, imputation to the 1000 Genomes reference panel was conducted according to standard protocols, as described previously^19^.

PROCARDIS participants were genotyped at the Centre National du Genotypage, Paris and the SNP&SEQ Technology Platform in Uppsala, using the Illumina 1 M and 610 K arrays. Imputation to the 1000 Genomes panel was conducted according to standard protocols, as described^20^.

UK Biobank participants were genotyped using either the Affymetrix UK Biobank Axion or the Affymetrix BiLEVE Axion array^16^. A modified version of SHAPEIT2 was used for phasing and IMPUTE2 for imputation. The data from UK Biobank was released in two phases. The UK Biobank was imputed to the 1000 Genomes, UK10K haplotype (first release) and Haplotype Reference Consortium (merged with the first release for the second release) reference panels^21^.

We applied standard quality control procedures to all cohorts, including SNP exclusion for low call rate (<95%), minor allele frequency (MAF < 1%), deviation from Hardy-Weinberg equilibrium (p < 5 × 10^−6^) or imputation quality score <0·4 and subject exclusion for sex mismatch, cryptic relatedness, low call rate (<95%) and non-Caucasian ancestry (self-reported or based on principle component analysis). For UK Biobank exclusions, further exclusions based on relatedness were applied (one of each pair of individuals with a KING-estimate kindship coefficient >0·0442 was randomly removed). After quality control, 5,684, 2,786, 5,452 and 5,361 SNPs were available for IMPROVE, SCARFSHEEP, PROCARDIS and UK Biobank respectively. In total, 2,123 SNPs were available in all four cohorts, with 2,133 overlapping between the three CAD case-control cohorts and 2,434 overlapping in the three cohorts with biomarker data.

### Phenotypes

Psychiatric and psychological phenotypes were only available in the UK Biobank. The baseline questionnaire included questions to assess mood instability (“does your mood often go up and down?” variable #1920) and risk-taking behaviour (“Would you describe yourself as someone who takes risks” variable #2040). Single item questions are imperfect ways to measure psychological traits, however validity of the question used here has been demonstrated relative to more detailed phenotyping (at least for risk-taking)^22^ and in terms of the expected associations with psychiatric disorders (for mood instability^23^ and risk-taking^7,8,24,25^). Neuroticism was assessed using the Eysenck Personality Questionnaire (Revised Short Form), where 12 yes/no questions were asked. These were summed, resulting in a score between one and 12 for each individual^26^. Phenotyping in relation to psychiatric disorders was based upon the online “Thoughts and Feelings” questionnaire^27^, which requested information on lifetime symptoms of mental disorders. This enabled classification of likely major depressive disorder (MDD), bipolar disorder (BD), generalised anxiety disorder (GAD) and addiction).

Anthropometric and blood pressure phenotypes (BMI, waist and hip circumferences, SBP and DBP) were assessed in a standardised and comparable manner. Waist to hip circumference ratio adjusted for BMI (WHRadjBMI) was calculated as per Shungin *et al*.^28^. For those on anti-hypertensive medication, values of SBP and DBP were adjusted, with 15 and 10 mmHG, respectively, being added prior to analysis^29^. Current smoking was assessed by questionnaire in all cohorts.

Metabolic parameters were available in IMPROVE, SCARFSHEEP and PROCARDIS, where fasting glucose, lipid (HDL, LDL and TG) and CRP levels were measured using standard methodology at the Department of Clinical Chemistry, Karolinska University Hospital. Fasting insulin levels were measured by radio-immunoassay^30,31^. HOMA indices were calculated from fasting glucose and insulin levels as described^32^. Type 2 diabetes was defined as diagnosis, medication and/or fasting glucose levels ≥7 mmol/L for IMPROVE, SCARFSHEEP and PROCARDIS. The definition of T2D in UK Biobank has been described^33^, and is generally comparable with the assessment used for the other cohorts.

Coronary vascular disease (CVD) was defined as clinically diagnosed myocardial infarction, symptomatic acute coronary syndrome, angina or coronary artery revascularisation before the age of 66 years for PROCARDIS^15^. Criteria for inclusion as a CVD case in the SCARFSHEEP study was clinical diagnosis of myocardial infarction diagnosis^34^. For UK Biobank, CVD was defined as clinical diagnosis of heart attack/myocardial infarction or angina (variable # 6150).

### Statistical analyses

All continuous phenotypes were assessed for normality and, where necessary, were natural log transformed prior to analysis. For each cohort, phenotypes were analysed in PLINK 1.07 using linear or logistic regression (for continuous vs. binary traits respectively), assuming additive allelic effects. With the exception of WHRadjBMI, all models included age, sex and population structure (3 principal components for PROCARDIS, SCARSHEEP and IMPROVE, 8 for UK Biobank), with further adjustment for genotyping chip being applied for UK Biobank and PROCARDIS analyses. For analysis of lipid traits, lipid-lowering medication and CVD case-control status were included as a covariates. For glucometabolic traits, individuals with type 2 diabetes were excluded and CVD case-control status was included as a covariate.

Results from the individual studies were combined in inverse variance-weighted meta-analyses using METAL^35^ (with binary effect sizes being analysed as Beta coefficients). Inverse variance-weighted meta-analysis was chosen, as the phenotype measurements and data transformation were comparable (including consistent units) between studies. Averages and standard errors of allele frequencies were computed. No additional filters were applied. Supplementary Table 1 summarises the phenotypes analysed in each cohort, covariates used and total sample number in the meta-analyses. Only SNPs present in 3 (of 3 or 4) cohorts were considered. Despite the prior knowledge implicating this locus in mental and physical health traits, we used a conservative approach, with genome-wide significance being set at p < 5 × 10^−8^ and suggestive evidence of association being set at p < 1 × 10^−5^. Locuszoom was used to visualise the results^36^.

### Genetic architecture

In order to determine whether the different traits have distinct signals, or whether there is one signal influencing all traits, two approaches were used:

Firstly, SNPs meeting suggestive or genome-wide significance thresholds for at least one phenotype (candidate SNPs) were identified and linkage disequilibrium (LD) assessed. For analysis of genetic architecture, a random subset of 1000 unrelated white British participants from the UK Biobank were selected. In this subset of UK Biobank, candidate SNPs were filtered to leave only independent SNPs, using PLINK (independent pairwise selection with default settings, including LD r^2^ threshold 0·5). LD between the independent SNPs and lead/index SNPs was calculated and visualised using Haploview^37^.

Secondly, conditional analyses were performed to further examine the possibility of multiple signals in the *CADM2* locus. Here, the risk-taking and BMI analyses were repeated, with the index SNP (coded as an additive genetic effect, namely 0 for common homozygote, 1 for heterozygotes and 2 for rare homozygotes) from each other phenotype in turn included as a covariate.

### Data-mining

The GWAS catalogue (https://www.ebi.ac.uk/gwas/, accessed 2018–09–04, 3:84,758,000–86,374,001) was used to identify *CADM2* locus SNPs previously associated with relevant phenotypes (specifically cardio-metabolic and psychiatric disorder-related traits). All SNPs in the *CADM2* locus with suggestive or genome-wide evidence for association with at least one phenotype were assessed for predicted functional effects using the Variant Effect Predictor^38^. For lead and index SNPS, the GTEx portal^39^ was queried to identify genotype-specific gene expression patterns (or expression quantitative traits loci (eQTLs)).

## Results

The cohort characteristics are presented in Table 1 and the phenotypes assessed are presented in Table 2.

**Table 1:** Cohort characteristics.

	UK Biobank	IMPROVE	PROCARDIS	SCARFSHEEP
N	408961	3390	7998	3417
Male	221052 (54.0)	1634 (48.2)	6009 (75.1)	2459 (72.0)
CAD cases	8319 (2.0)	0 (0)	5688 (71.1)	1525 (44.6)
T2D cases	17766 (4.3)	908 (26.8)	1157 (14.5)	341 (10.0)
Age (years)	56.9 (8.0)	64.2 (5.4)	60.0 (8.3)	58.1 (7.27)
BMI (kg/m^2^)	27.42 (4.76)	27.2 (4.24)	27.9 (4.4)	26.3 (3.9)
Waist:hip ratio	0.87 (0.09)	0.91 (0.09)	0.97 (0.08)	0.94 (0.09)
SBP (mmHg)	137 (19)	142 (18)	135 (20)	135 (21)
DBP (mmHG)	82 (10)	82 (10)	81 (11)	82 (10)
Current smoking	144005 (35.3)	507 (15.0)	1137 (15.4)	1133 (33.8)
Lipid-lowering medication	43424 (26.0)	1676 (49.5)	4175 (56.6)	235 (6.8)
Anti-hypertensive medication	85463 (21.0)	1950 (57.5)	4592 (62.3)	1644 (48.1)
LDL cholesterol (mmol/L)	na	3.54 (1.00)	2.98 (0.87)	4.02 (0.99)
HDL cholesterol (mmol/L)	na	1.26 (0.36)	1.22 (0.36)	1.17 (0.36)
Triglycerides (mmol/L)	na	5.93 (1.66)	1.83 (1.26)	1.70 (1.16)
CRP (mmol/L)	na	2.97 (5.76)	3.44 (7.20)	2.94 (5.69)
Fasting glucose (mmol/L)*	na	5.28 (0.66)	5.38 (0.60)	5.10 (0.67)
Fasting insulin (pmol/L)*	na	44.6 (61.8)	57.9 (54.3)	58.6 (45.2)
HOMA B*	na	69.1 (54.1)	79.8 (32.6)	25.7 (11.0)
HOMA IR*	na	0.83 (1.10)	1.02 (0.72)	0.16 (0.11)
BD	1899 (1.4)	na	na	na
GAD	9251 (10.2)	na	na	na
MDD	31338 (28.2)	na	na	na
Addiction	7575 (5.8)	na	na	na
Mood instability	180743 (44.2)	na	na	na
Risk-takers	32735 (25.5)	na	na	na
Neuroticism score	4.11 (3.26)	na	na	na

Where: CAD, coronary artery disease; BMI, body mass index; BD, bipolar disorder; GAD, generalised anxiety disorder; MDD, major depressive disorder. *In non T2D subjects. Continuous variables are presented as mean (sd), binary variables are presented as n (%).

**Table 2:** Phenotypes and N available.

Phenotype	UK Biobank	Procardis	IMPROVE	SCARFSHEEP	Maximum N
BMI*	Y	Y	Y	Y	416136
WHRadjBMI*	Y	Y	Y	Y	416136
SBP*	Y	Y	Y	Y	397163
DBP*	Y	Y	Y	Y	397163
Diabetes*	Y	Y	Y	Y	417615
Current smoking*	Y	Y	Y	Y	416136
CVD	Y	Y		Y	414116
TGs*		Y	Y	Y	14799
CRP*		Y	Y	Y	14799
HDL*		Y	Y	Y	14799
LDL*		Y	Y	Y	14799
Glucose*^		Y	Y	Y	10128
Insulin*^		Y	Y	Y	10128
HOMA-IR*^		Y	Y	Y	10128
HOMA-B*^		Y	Y	Y	10128
BD	Y				129366
GAD	Y				90536
MDD	Y				109436
Addiction	Y				129858
Mood instability	Y				393367
Risk taking	Y				328339
Neuroticism	Y				328087

Where: WHRadjBMI, waist:hip ratio adjusted for BMI; ISH, ischemic heat disease, including myocardial infarction and coronary artery disease; BD, bipolar disorder; GAD, general anxiety disorder; MDD, major depressive disorder; addiction, any addiction. All models adjusted for age, sex and population structure. *Additional adjustment for CVD status. ^Participants with T2D excluded from these analyses.

### Meta-analysis of cardiovascular and metabolic phenotypes

Only SNPs present in at least 3 of the 4 cohorts were considered. It is worth noting that the heterogeneity I^2^ value was high for many SNPs in the meta-analysis of UK Biobank, IMPROVE, PROCARDIS and SCARFHSEEP. This could be due to selection of UK vs European or population vs case-control participants. Therefore we present results for both a lead SNP (defined as the SNP with the lowest P-value) and an index SNP (defined as the SNP with the lowest p-value with heterogeneity I^2^ = 0), but for robustness we focus on the Index SNPs.

**Figure 1:**
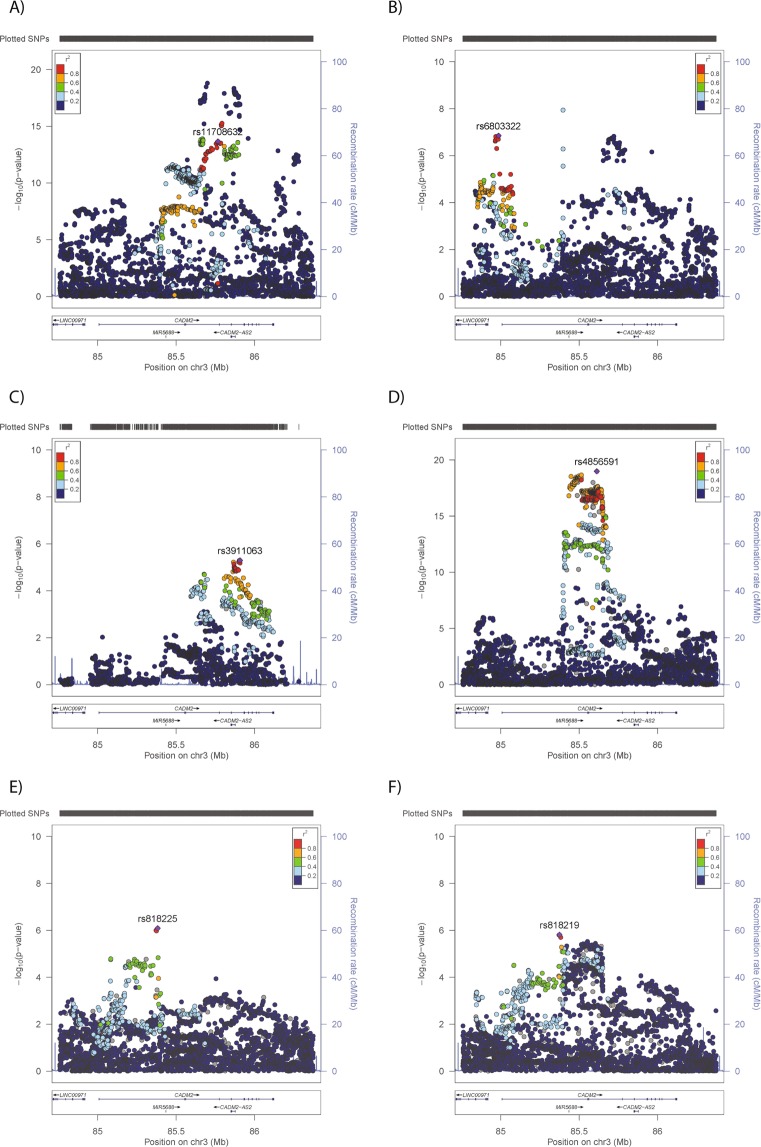
Regional plots for phenotypes with GWAS significant (p < 5 × 10^−8^) or suggestive (p < 1 × 10^−5^) evidence of association with the *CADM2* locus. Results from meta-analysis of (**A**) BMI, (**B**) SBP, (**C**) CRP levels or UK Biobank-only analyses of (**D**) risk-taking behaviour, (**E**) mood instability and (**F**) neuroticism.

Evidence of association was observed for BMI, with 908 SNPs reaching genome-wide significance (Index SNP rs11708632-A, Beta 0·085, p = 2·18 × 10^−14^) (Fig. 1A and Table 3), of which 166 had heterogeneity I^2^ = 0. A further 362 SNPs met the threshold for suggestive evidence of association with BMI (p < 1 × 10^−5^), 140 of which had heterogeneity I^2^ = 0 (Supplementary Table 1). For SBP, only one SNP reached genome-wide significance but heterogeneity was high (heterogeneity I^2^ = 69%, rs146071762-A, Beta 0·248, p = 1·14 × 10^−8^). An additional 63 SNPs demonstrated suggestive associations with SBP, with 10 showing heterogeneity I^2^ = 0 (Index SNP, rs6803322-A, Beta −0·024, p = 1·41 × 10^−7^) (Fig. 1B, Table 3, Supplementary Table 1).

**Table 3:** CADM2 SNPs with suggestive or genome-wide evidence of association.

Phenotype	Chr	SNP	Position	A1	A1F	Fse	Beta	Se	P	Effect	I^2^	P_het_	Description	N GWAS SNPs	N suggestive SNPs
BMI	3	rs11915747	85699040	C	0.64	0.01	0.090	0.010	**1.58E-19**	++++	73	0.011	lead SNP	908	362
BMI	3	rs11708632	85766667	A	0.24	0.00	0.085	0.011	**2.18E-14**	++++	0	0.417	Index SNP with I2 = 0		
SBP	3	rs146071762	85396778	A	0.51	0.02	0.248	0.044	**1.14E-08**	+−+?	69	0.039	lead SNP	1	63
SBP	3	rs6803322	84986088	A	0.32	0.00	−0.243	0.046	1.41E-07	−+−?	0	0.619	Index SNP with I2 = 0		
CRP	3	3:85906663**	85906663	T	0.31	0.01	−0.072	0.015	2.69E-06	−−	16	0.303	lead SNP		11
CRP	3	rs11708024	85865269	A	0.30	0.01	−0.069	0.015	6.05E-06	−−	0	0.674	Index SNP with I2 = 0		
risk-taking*	3	rs4856591	85612550	T	0.38		0.056	0.006	**1.02E-19**				Index SNP	809	172
mood instability*	3	rs818225	85382140	T	0.46		−0.023	0.005	8.12E-07				Index SNP		4
neuroticism*	3	rs818219	85374589	C	0.46		−0.038	0.008	1.54E-06				Index SNP		88

Where: A1, effect allele; A1F, effect allele frequency; I2, measure of heterogeneity (Higgens *et al*. 2013), Phet, heterogeneity Pvalue. ^*^In UK Biobank only. Lead SNP, SNP with lowest Pvalue; Index SNP, lowest Pvalue SNP with heterogeneity I2 = 0. **Not available in UKB.

No associations were observed for WHRadjBMI, DBP, T2D or current smoking (Supplementary Fig. 1A–D). For CVD analysis, SNPs were only considered if they were present in all three cohorts. None met the threshold for suggestive significance (Supplementary Fig. 1E).

### Meta-analysis of cardio-metabolic biomarkers

In the meta-analysis of cardio-metabolic biomarkers, only SNPs present in all 3 clinical cohorts were considered. Eleven SNPs demonstrated suggestive associations (p < 1 × 10^−5^) with CRP levels, of which five demonstrated heterogeneity I^2^ = 0 (index SNP, rs11708024-A, Beta −0·069, p = 6·05 × 10^−6^. For six CRP-associated SNPs, heterogeneity was moderate (I2 16–35%) but non-significant (Table 3). No evidence for associations with lipid levels (HDL-C, LDL-C, TGs) or glucometabolic biomarkers (fasting glucose, insulin, HOMA-B, HOMA-IR) were observed (Supplementary Fig. 2).

### CADM2 vs psychological and psychiatric phenotypes in the UK Biobank

As previously reported^7,8^, *CADM2* variants were associated with risk-taking behaviour, with 809 SNPs demonstrating genome-wide evidence of association (p < 5 × 10^−8^ for risk-taking behaviour (Index SNP rs4856591-T, Beta = 0.056, p = 1.02 × 10^−19^); Fig. 1D and Table 3). In addition, suggestive evidence of association was observed for neuroticism (88 SNPs, index SNP rs818219-C, beta = −0·038, p = 1.54 × 10^−6^) and mood instability (4 SNPs, index SNP rs818225-T, beta = −0.02283, p = 8.12 × 10^−7^; Fig. 1E,F and Table 3). No SNPs demonstrated suggestive evidence of association with MDD, GAD, BD or addiction (Supplementary Fig. 3).

### Cross-trait observations

A total of 49 SNPs demonstrated at least suggestive associations with multiple phenotypes (Supplementary Table 2). This observation demonstrates, firstly that the same SNPs influence both metabolic and psychological traits, and secondly that effects on risk-taking, BMI and CRP were positively correlated and these were inversely correlated with effects on neuroticism, mood instability and SBP.

### Genetic architecture of CADM2

**Figure 2:**
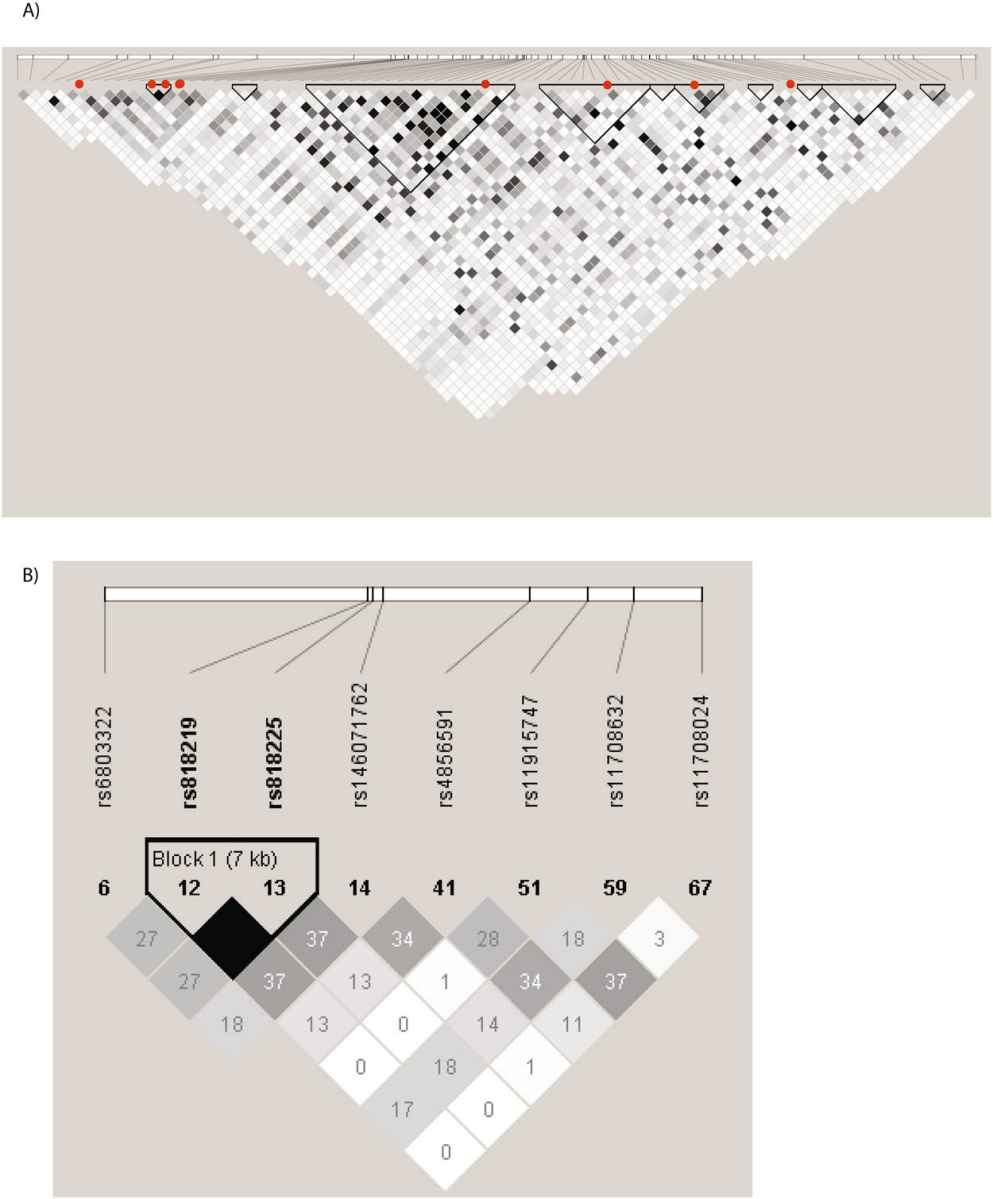
Linkage disequilibrium in the *CADM2* locus in UK Biobank with (**A**) the lead SNPs, Index SNPs and independent loci, (**B**) the lead and index SNPs. SNPs are arranged in order of base pair position. Red dots indicate the lead and index SNPs.

In order to determine whether the associations with psychological and physical traits reflect the same or distinct signals, two approaches were used. Firstly, filtering the 1,533 candidate SNPs (SNPs meeting suggestive significance of association with any phenotype) in the 1.62 Mb *CADM2* locus by LD gave 75 independent loci (Fig. 2A). The index SNPs for neuroticism (rs818219) and mood instability (rs818225) are in perfect LD, therefore represent the same signal, whereas LD between index SNPs for other traits (BMI, rs11708632; SBP, rs6803322; CRP, rs11708024; risk-taking, rs485659) is low (maximum r^2^ = 0·37, Fig. 2B), which could indicate independent signals for each other phenotype.

**Figure 3:**
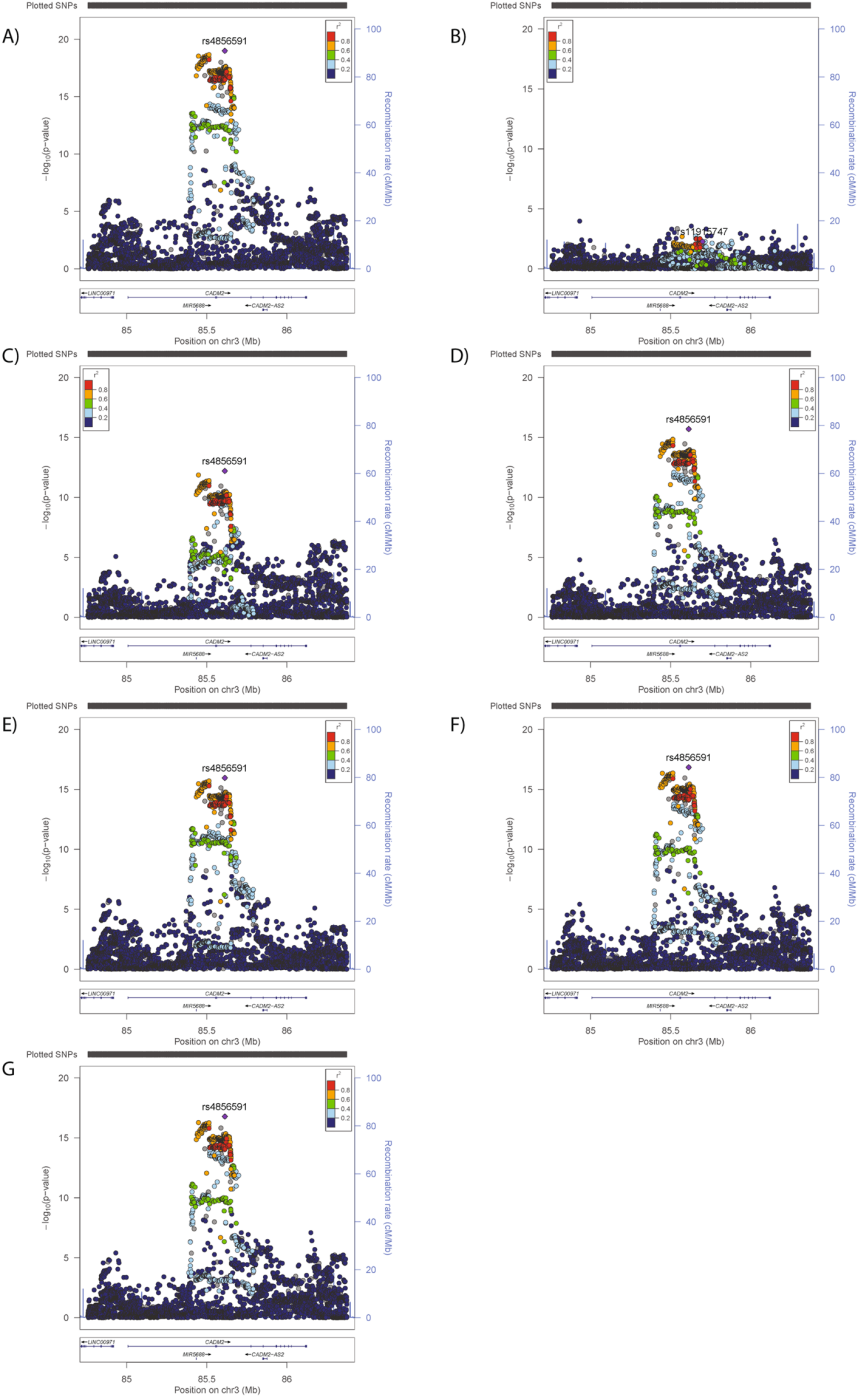
Regional plots for analyses of (**A**) risk-taking, conditioned on (**B**) risk-taking index SNP, (**C**) BMI index SNP, (**D**) SBP index SNP, (**E**) CRP index SNP, (**F**) mood instability index SNP, (**G**) neuroticism index SNP.

Secondly, conditional analysis using the index SNPs (BMI, rs11708632; SBP, rs6803322; CRP, rs11708024; risk-taking, rs4856591; neuroticism, rs818219; mood instability, rs818225) was performed, using the UK Biobank data. If there is only one signal in the locus, then adjusting for the index SNP would remove the effects/significance of other SNPs in the locus. Alternatively, if there are additional signals in the locus which are independent of the index SNP, then adjusting for the index SNP would have little or no impact on the independent signal in terms of effect size or significance. The risk-taking results demonstrated that inclusion of index SNPs had some effect on the p-value of the risk-taking signal (Fig. 3), but the effect size was stable (primary risks analysis Beta = 0.056, conditional Betas = 0.054–0.057), which concurs with the LD analysis suggestion that the signals are independent. The BMI results were similar, but the effect size was less stable (primary BMI analysis Beta = −0.099, conditional Betas = −0.087–0.101) and the plots support the possibility of more than one signal in this region (Supplementary Fig. 4).

### eQTL analysis

**Figure 4:**
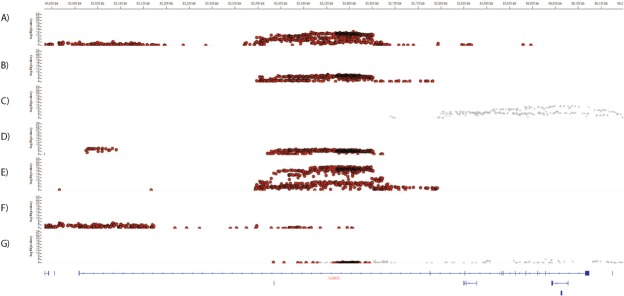
IGV regional plot of associations of SNPs with expression levels of *CADM2* in (**A**) subcutaneous adipose tissue, (**B**) visceral adipose tissue, (**C**) brain-cerebellum, (**D**) heart-left ventricle, (**E**) skeletal muscle, (**F**) lung and (**G**) tibial nerve. Position in base pairs is given on the X-axis (top) and in relation to the gene (bottom). The Y-axis gives the p value for the association in each tissue. Red indicates SNPs with significant associations with *CADM2* (at FDR < 5%), Grey indicates SNPs with significant associations with *CADM2-AS1* (at FDR < 5%).

Firstly, SNPs with genotype-specific effects on mRNA *CADM2* levels were identified using the GTEx portal (Fig. 4). The location of the eQTLs appears to be tissue specific (Fig. 4): In subcutaneous adipose tissue, eQTLs are located upstream and centrally relative to the gene location, whereas those in visceral adipose tissue are restricted to the central part of the gene (Fig. 4A,B). In heart (left ventricle) tissue and skeletal muscle, eQTLs are preferentially (but not exclusively) located centrally, whereas eQTLs in lung tissue are mainly upstream (Fig. 4D,F). In contrast, there are no eQTLs for *CADM2* in the brain (cerebellum), instead the eQTLs in this brain region are for *CADM2-AS1* (Fig. 4C). These findings suggest the potential for differential regulation of *CADM2* levels across a range of tissues, thereby explaining how different SNPs within the locus can influence a variety of traits.

Secondly, 1747 SNPs with eQTL effects on *CADM2* and *CADM2-AS1* were identified and are presented in Supplementary Table 3. No eQTLs for *CADM2-AS2* were identified. For *CADM2* there were 3702 eQTLS (each SNP can have eQTL effects in more than one tissue), of which 41% were in adipose tissue (subcutaneous or visceral) and 5% in brain tissues. For *CADM2-AS1*, 1628 eQTLs were identified, of which 90% were in brain tissues and none were in adipose tissues.

**Figure 5:**
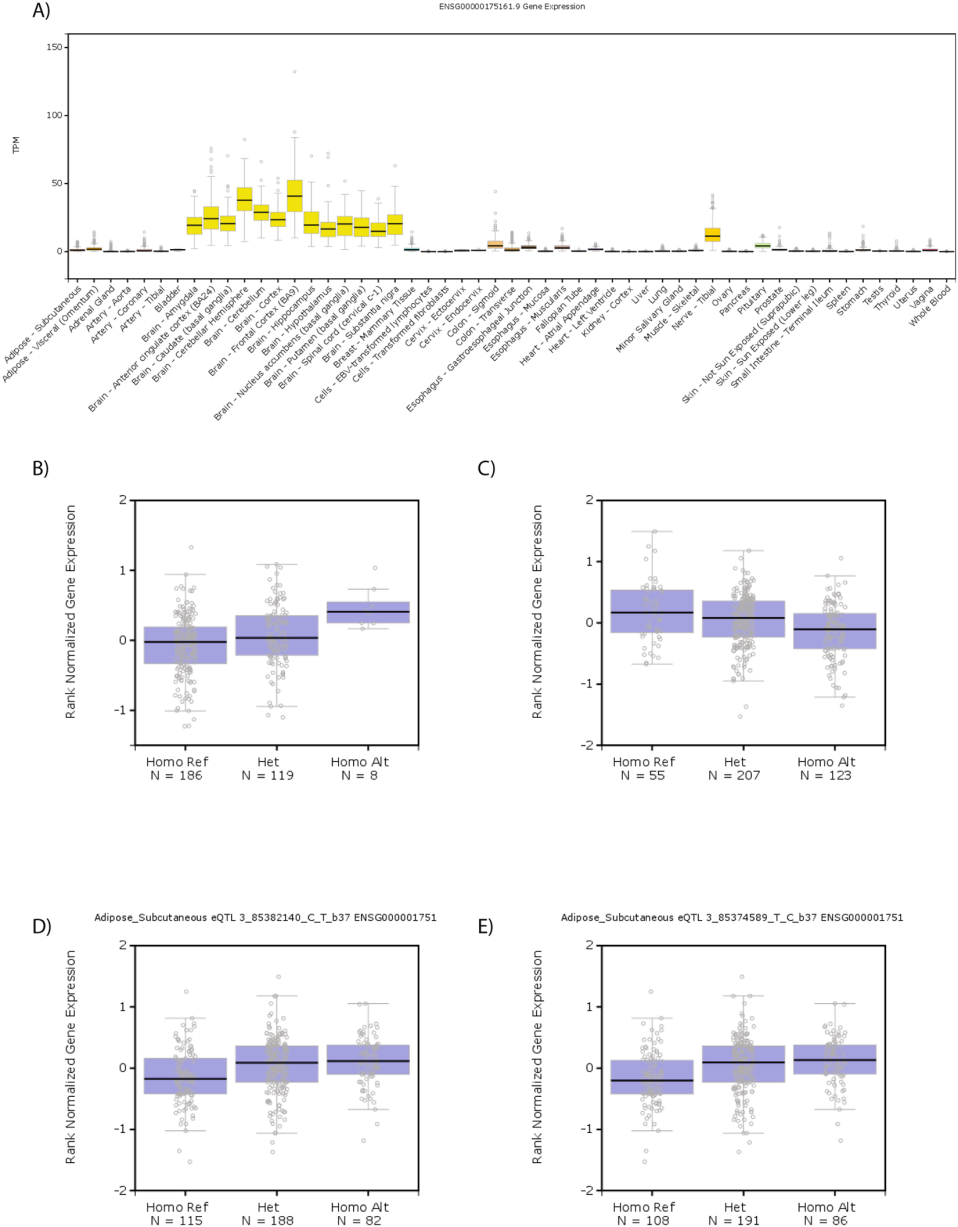
Expression of *CADM2* mRNA is (**A**) predominantly observed in the brain and higher levels are associated with (**B**) the BMI-increasing allele of rs11708632 (A, Alt), (**C**) the risk-taking allele of rs4856591 (T, Ref), (**D**) the mood instability-decreasing allele of rs818225 (T, Alt) and the neuroticism-decreasing allele of rs818219 (C, Alt).

Finally, all of the index SNPs available in GTEx (rs1170802 was not available) demonstrated eQTLs for *CADM2* and none were eQTLs for *CADM2-AS1* (Table 4). Expression levels of *CADM2* are highest in the brain (Fig. 5A). However, it was interesting to note that the trait-increasing alleles of rs11708632 (BMI) and rs4856591 (risk-taking) were associated with increased *CADM2* expression levels in adipose tissue (Fig. 5B,C). Consistent with their inverse correlations with BMI and risk-taking, the trait-decreasing alleles of rs818225 (mood instability) and rs818219 (neuroticism) were associated with increased *CADM2* expression in adipose tissue (Fig. 5D,E).

**Table 4:** eQTLs of lead and index SNPs.

SNP	Gene Symbol	SNP Id	P-Value	EA	NES	Tissue	Trait-increasing allele
BMI lead	*CADM2*	rs11915747	8.60E-07	G	−0.23	Lung	C
BMI lead	*CADM2-AS1*	rs11915747	1.30E-04	G	0.25	Nerve - Tibial	C
BMI index	*CADM2*	rs11708632	6.40E-06	A	0.27	Lung	A
BMI index	*CADM2*	rs11708632	5.10E-05	A	0.23	Adipose - Visceral (Omentum)	A
SBP index	*CADM2*	rs6803322	1.10E-08	A	0.71	Brain - Spinal cord (cervical c-1)	C
SBP index	*CADM2*	rs6803322	1.50E-06	A	0.19	Muscle - Skeletal	C
SBP index	*CADM2*	rs6803322	7.20E-06	A	0.2	Adipose - Subcutaneous	C
CRP lead	*CADM2-AS1*	3:85906663	2.80E-08	T	0.52	Brain - Cerebellum	TGTTGCTCAG
CRP lead	*CADM2-AS1*	3:85906663	1.30E-06	T	0.56	Brain - Caudate (basal ganglia)	TGTTGCTCAG
CRP lead	*CADM2-AS1*	3:85906663	1.60E-05	T	0.29	Nerve - Tibial	TGTTGCTCAG
CRP lead	*CADM2-AS1*	3:85906663	3.60E-05	T	0.49	Brain - Cerebellar Hemisphere	TGTTGCTCAG
risk-taking	*CADM2*	rs4856591	6.30E-16	G	−0.39	Lung	T
risk-taking	*CADM2*	rs4856591	6.30E-07	G	−0.22	Adipose - Subcutaneous	T
risk-taking	*CADM2*	rs4856591	1.70E-06	G	−0.39	Heart - Left Ventricle	T
risk-taking	*CADM2*	rs4856591	2.10E-06	G	−0.22	Adipose - Visceral (Omentum)	T
neuroticism	*CADM2*	rs818219	3.80E-06	C	0.19	Adipose - Subcutaneous	T
neuroticism	*CADM2*	rs818219	1.20E-04	C	0.14	Muscle - Skeletal	T
mood instability	*CADM2*	rs818225	4.20E-05	T	0.17	Adipose - Subcutaneous	C

NES, Normalised effect size.

### Data-mining

None of the index SNPs have previously been associated with any traits in the GWAS catalogue. Additionally, none of the candidate SNPs were predicted by Variant Effect Predictor to have more than low or modifier impact.

SNPs in the *CADM2* locus which have previously been associated with psychological or metabolic traits are presented in Supplementary Table 4. Where comparison was possible, SNPs with reported effects on measures of obesity demonstrated consistent effect directions in our study compared to those previously reported^10,28,40,41^, with one exception: rs12495178 has the opposite effect directions in the Japanese population^42^ compared to our study. It is noteworthy that SNPs associated with educational attainment or intelligence^3,43^, were associated with increased BMI, which is somewhat surprising, but consistent with effects on risk-taking behaviour^7,8^. Maybe unsurprisingly, rs62253088-T (associated with strenuous exercise^6^) had positive associations with risk-taking and negative associations with neuroticism. Other associations with physical activity habits^6^ and alcohol consumption^4^ demonstrate less consistent associations with risk-taking.

## Discussion

We identified novel associations between *CADM2* genetic variants and SBP, CRP levels, neuroticism and mood instability, and have highlighted a possible link between SNPs associated with psychological traits and adiposity via *CADM2* expression levels in adipose tissue.

Associations between *CADM2* SNPs and obesity have previously been reported^10,28,40–42,44–47^ and were observed here. It is possible that the associations of *CADM2* SNPs with CRP and SBP are secondary to the effects on obesity, as increased fat accumulation is associated with systemic inflammation^2^ and reduced cardiovascular fitness^48^. Associations between *CADM2* SNPs and risk-taking behaviour have also been reported^7,8,25^. We previously suggested that the association between risk-taking and obesity might be behavioural, with risk-takers choosing to disregard health-related advice and/or are prone to aberrant reward circuitry predisposing them to poor dietary choices and excessive intake^7^.

Pleiotropy, where genetic variants influence more than one trait, is a concern in genetic studies. Pleiotropy can be classified as biological (where a genetic variant has true effects on more than one trait), mediated (where a genetic variant has a true effect on one trait, but because there is a causal relationship between that trait and a second trait, effects of the genetic variant are seen on the second trait as well) or spurious (where biases in the study result in genetic effects on multiple traits)^49^. When considering the effects of *CADM2* variants on both psychological and obesity traits, a possible explanation is mediated pleiotropy, for example through physical exercise. If the *CADM2* variant effects on behavioural traits such as risk-taking, neuroticism, mood instability (observed here) and physical exercise^6^ are true, then the effects on obesity might be knock-on effects of physical exercise. Whilst possible, the *CADM2* variants associated with increased physical activity were associated with increased BMI^6^, therefore this logic of this argument is flawed. Spurious pleiotropy is another possibility; however there are consistent effects of *CADM2* on psychological and obesity traits in a number of cohorts with different recruitment and study designs (population-based, CVD case-control, high CVD risk) and populations (European, UK, north American, Pakistan), which would be expected to differ in their biases. Whether spurious pleiotropy can result from such a variety of biases is doubtful.

In contrast, biological pleiotropy is supported by the body of evidence indicating a role for *CADM2* in psychological and obesity traits from other types of studies. Mouse models demonstrate a clear effect of *Cadm2* on obesity and gluco-metabolic parameters: A global *Cadm2*-knockout mouse demonstrated reduced body weight, improved insulin sensitivity and improved glucose tolerance^50^. Furthermore, this effect was maintained when *Cadm2*-knockout mice were crossed with the traditional obesity model, the *Leptin*-knockout mouse^11^. Rat models also demonstrate that increased *Cadm2* (via a knockdown of a cadm2 regulator) reduced neurite outgrowth in response to ischemic damage^51^ whilst errors in axon pathfinding and neurite outgrowth were observed in C*adm2*-deficient chick embryos^52^. *In vivo* and *in vitro* studies of tumour models have demonstrated that increased expression of *CADM2* mRNA or protein is associated with reduced cell viability, proliferation, migration and invasion in glioma^53^, retinoblastoma^54^, renal cell^55^, hepatocellular^56,57^, endometrial^58^, prostate^59^ and oesophageal squamous cell carcinomas^60^. The wide range of cell types suggests that *CADM2* regulation of cell turnover could be ubiquitous. With this in mind, neuronal remodelling is important for health and pathology and requires cell turnover, so levels of *CADM2* likely influence plasticity of the brain. Similarly, to increase the fat that can be stored, adipocytes either increase in volume (metabolically detrimental) or in number (metabolically benign)^61^. *CADM2* levels could be a part of the volume vs number fate determination.

Further support for biological pleiotropy comes from the observation that *CADM2* SNPs associated with risk-taking (rs4856591), neuroticism (rs818219) and mood instability (rs818225) had eQTLs for adipose tissue (and therefore potentially direct effects on adiposity). This is especially interesting in light of the established risk of obesity in psychiatric disorders and the relevance of these traits to a wider range of psychiatric disorders (MDD, GAD, SCZ, BD) than risk-taking (SCZ and BD). A common biological mechanism, such as *CADM2*, might also be consistent with the recent observation of a bi-directional link between depression and obesity^62^.

This study did not find evidence for effects of *CADM2* on psychiatric diagnoses, although it should be noted that the number of cases for these analyses were low. We also cannot exclude psychiatric medication as a confounder in our analyses of cardiometabolic variables, however the very low percentage of individuals on psychiatric medication means that this is very unlikely. No associations were identified for CVD, which is unlikely to be due to the number of cases present. We also note that, in comparison to the *Cadm2*-knockout mice, no effects on glucose-related traits were observed. This may be due to a smaller sample size (N = 10,128) and thus reduced power for these biomarkers, or selection bias due to studying cardiovascular cohorts. It is also possible that effects of *CADM2* on insulin sensitivity and glucose levels are secondary to effects on BMI, making it harder to discern.

A surprising finding of this study was the long-range linkage disequilibrium within the *CADM2* locus, despite low LD, with effects being evident over a region of nearly 1 Mb. This means that the SNPs identified for psychological and metabolic traits are not independent. Haplotype analysis would be of value here, however the standard approaches rely on higher LD and smaller regions, so this has yet to be attempted. This consideration, combined with a plethora of eQTLs in a variety of tissues, results in complexity regarding the regulation of *CADM2* levels. Further functional investigation of *CADM2* is required for complete mechanistic understanding of this locus.

There are some limitations to this study, notably incomplete genetic coverage of the locus in the IMPROVE and SCARFSHEEP cohorts and reduced sample sizes for the biomarker analyses. In addition, only the UK Biobank had both psychological and cardio-metabolic phenotyping. As is typical for the majority of cardio-metabolic studies, history of psychiatric illness was an exclusion criterion for IMPROVE, SCARFSHEEP and PROCARDIS, therefore it is possible that these cohorts have lower levels of variants associated with psychiatric disorders than the general population. Conversely, these cohorts have higher rates of cardiovascular risk factors and disease than the general population (average/general population, to moderate/early CVD, to high risk/at least 3 CVD risk factors). Whilst this is a strength when looking at cardiovascular phenotypes, it is likely to contribute to the high I^2^ demonstrated for some SNPs in the meta-analyses. Despite this, there are a number of variants that show significant associations, with I^2^ = 0, and these were used for the follow-up analyses. The consistency in effect sizes and directions for these associations with BMI and CRP are striking, especially being irrespective of CVD risk burden. These findings also demonstrate that the effects of *CADM2* variants on cardio-metabolic parameters are generalizable to a wider European ancestry population. The same cannot be assumed for the psychological phenotypes, which were analysed exclusively in white British UK Biobank participants. Strengths of the study include large sample sizes for most analyses. The meta-analyses of several cohorts provides robust results, whereas consistent phenotyping is a clear advantage of the UK Biobank study.

In conclusion, we have conducted a systematic, large-scale analysis of multiple datasets providing evidence that *CADM2* represents a putative shared biological link between metabolic and psychological disorders. Future work, including animal models which investigate both metabolic and behavioural traits in the same animals, is now needed to understand the functional biological mechanisms that might explain this link.

## Supplementary information

Supplementary data

## Data Availability

Data is available on request, contact either UK Biobank (UK Biobank-only analyses) or the corresponding author (all other analyses).

## References

[1] Amare AT, Schubert KO, Klingler-Hoffmann M, Cohen-Woods S, Baune BT (2017). The genetic overlap between mood disorders and cardiometabolic diseases: a systematic review of genome wide and candidate gene studies. Transl Psychiatry.

[2] Ouakinin SRS, Barreira DP, Gois CJ (2018). Depression and Obesity: Integrating the Role of Stress, Neuroendocrine Dysfunction and Inflammatory Pathways. Front Endocrinol (Lausanne).

[3] Okbay A (2016). Genome-wide association study identifies 74 loci associated with educational attainment. Nature.

[4] Clarke TK (2017). Genome-wide association study of alcohol consumption and genetic overlap with other health-related traits in UK Biobank (N = 112 117). Mol Psychiatry.

[5] Pasman JA (2018). GWAS of lifetime cannabis use reveals new risk loci, genetic overlap with psychiatric traits, and a causal influence of schizophrenia. Nat Neurosci.

[6] Klimentidis YC (2018). Genome-wide association study of habitual physical activity in over 377,000 UK Biobank participants identifies multiple variants including CADM2 and APOE. Int J Obes (Lond).

[7] Strawbridge RJ (2018). Genome-wide analysis of self-reported risk-taking behaviour and cross-disorder genetic correlations in the UK Biobank cohort. Transl Psychiatry.

[8] Strawbridge RJ (2018). Genetics of self-reported risk-taking behaviour, trans-ethnic consistency and relevance to brain gene expression. Transl Psychiatry.

[9] Albayrak O (2013). Common obesity risk alleles in childhood attention-deficit/hyperactivity disorder. Am J Med Genet B Neuropsychiatr Genet.

[10] Graff M (2017). Genome-wide physical activity interactions in adiposity - A meta-analysis of 200,452 adults. PLoS Genet.

[11] Yan X (2018). Cadm2 regulates body weight and energy homeostasis in mice. Mol Metab.

[12] Baldassarre D (2010). Cross-sectional analysis of baseline data to identify the major determinants of carotid intima-media thickness in a European population: the IMPROVE study. Eur Heart J.

[13] Reuterwall C (1999). Higher relative, but lower absolute risks of myocardial infarction in women than in men: analysis of some major risk factors in the SHEEP study. The SHEEP Study Group. J Intern Med.

[14] Samnegard A (2005). Serum matrix metalloproteinase-3 concentration is influenced by MMP-3 -1612 5A/6A promoter genotype and associated with myocardial infarction. J Intern Med.

[15] Farrall M (2006). Genome-wide mapping of susceptibility to coronary artery disease identifies a novel replicated locus on chromosome 17. PLoS Genet.

[16] Biobank, U. Genotyping of 500,000 UK Biobank participants. Description of sample processing workflow and preparation of DNA for genotyping, 11 September 2015 (2015).

[17] Voight BF (2012). The metabochip, a custom genotyping array for genetic studies of metabolic, cardiovascular, and anthropometric traits. PLoS Genet.

[18] Trynka G (2011). Dense genotyping identifies and localizes multiple common and rare variant association signals in celiac disease. Nat Genet.

[19] Scott Robert A., Scott Laura J., Mägi Reedik, Marullo Letizia, Gaulton Kyle J., Kaakinen Marika, Pervjakova Natalia, Pers Tune H., Johnson Andrew D., Eicher John D., Jackson Anne U., Ferreira Teresa, Lee Yeji, Ma Clement, Steinthorsdottir Valgerdur, Thorleifsson Gudmar, Qi Lu, Van Zuydam Natalie R., Mahajan Anubha, Chen Han, Almgren Peter, Voight Ben F., Grallert Harald, Müller-Nurasyid Martina, Ried Janina S., Rayner Nigel W., Robertson Neil, Karssen Lennart C., van Leeuwen Elisabeth M., Willems Sara M., Fuchsberger Christian, Kwan Phoenix, Teslovich Tanya M., Chanda Pritam, Li Man, Lu Yingchang, Dina Christian, Thuillier Dorothee, Yengo Loic, Jiang Longda, Sparso Thomas, Kestler Hans A., Chheda Himanshu, Eisele Lewin, Gustafsson Stefan, Frånberg Mattias, Strawbridge Rona J., Benediktsson Rafn, Hreidarsson Astradur B., Kong Augustine, Sigurðsson Gunnar, Kerrison Nicola D., Luan Jian'an, Liang Liming, Meitinger Thomas, Roden Michael, Thorand Barbara, Esko Tõnu, Mihailov Evelin, Fox Caroline, Liu Ching-Ti, Rybin Denis, Isomaa Bo, Lyssenko Valeriya, Tuomi Tiinamaija, Couper David J., Pankow James S., Grarup Niels, Have Christian T., Jørgensen Marit E., Jørgensen Torben, Linneberg Allan, Cornelis Marilyn C., van Dam Rob M., Hunter David J., Kraft Peter, Sun Qi, Edkins Sarah, Owen Katharine R., Perry John R.B., Wood Andrew R., Zeggini Eleftheria, Tajes-Fernandes Juan, Abecasis Goncalo R., Bonnycastle Lori L., Chines Peter S., Stringham Heather M., Koistinen Heikki A., Kinnunen Leena, Sennblad Bengt, Mühleisen Thomas W., Nöthen Markus M., Pechlivanis Sonali, Baldassarre Damiano, Gertow Karl, Humphries Steve E., Tremoli Elena, Klopp Norman, Meyer Julia, Steinbach Gerald, Wennauer Roman, Eriksson Johan G., Mӓnnistö Satu, Peltonen Leena, Tikkanen Emmi, Charpentier Guillaume, Eury Elodie, Lobbens Stéphane, Gigante Bruna, Leander Karin, McLeod Olga, Bottinger Erwin P., Gottesman Omri, Ruderfer Douglas, Blüher Matthias, Kovacs Peter, Tonjes Anke, Maruthur Nisa M., Scapoli Chiara, Erbel Raimund, Jöckel Karl-Heinz, Moebus Susanne, de Faire Ulf, Hamsten Anders, Stumvoll Michael, Deloukas Panagiotis, Donnelly Peter J., Frayling Timothy M., Hattersley Andrew T., Ripatti Samuli, Salomaa Veikko, Pedersen Nancy L., Boehm Bernhard O., Bergman Richard N., Collins Francis S., Mohlke Karen L., Tuomilehto Jaakko, Hansen Torben, Pedersen Oluf, Barroso Inês, Lannfelt Lars, Ingelsson Erik, Lind Lars, Lindgren Cecilia M., Cauchi Stephane, Froguel Philippe, Loos Ruth J.F., Balkau Beverley, Boeing Heiner, Franks Paul W., Barricarte Gurrea Aurelio, Palli Domenico, van der Schouw Yvonne T., Altshuler David, Groop Leif C., Langenberg Claudia, Wareham Nicholas J., Sijbrands Eric, van Duijn Cornelia M., Florez Jose C., Meigs James B., Boerwinkle Eric, Gieger Christian, Strauch Konstantin, Metspalu Andres, Morris Andrew D., Palmer Colin N.A., Hu Frank B., Thorsteinsdottir Unnur, Stefansson Kari, Dupuis Josée, Morris Andrew P., Boehnke Michael, McCarthy Mark I., Prokopenko Inga (2017). An Expanded Genome-Wide Association Study of Type 2 Diabetes in Europeans. Diabetes.

[20] Nikpay M (2015). A comprehensive 1,000 Genomes-based genome-wide association meta-analysis of coronary artery disease. Nat Genet.

[21] Biobank, U. Genotype imputation and genetic association studies of UK Biobank, Interim Data Release, 11 September 2015 (2015).

[22] Frey R, Pedroni A, Mata R, Rieskamp J, Hertwig R (2017). Risk preference shares the psychometric structure of major psychological traits. Sci Adv.

[23] Ardila Alfredo, Bernal Byron, Rosselli Monica (2017). Executive Functions Brain System: An Activation Likelihood Estimation Meta-analytic Study. Archives of Clinical Neuropsychology.

[24] Boutwell B (2017). Replication and characterization of CADM2 and MSRA genes on human behavior. Heliyon.

[25] Day FR (2016). Physical and neurobehavioral determinants of reproductive onset and success. Nat Genet.

[26] Smith DJ (2016). Genome-wide analysis of over 106 000 individuals identifies 9 neuroticism-associated loci. Mol Psychiatry.

[27] Davis K (2018). Mental health in UK Biobank – implementation and results of an online questionnaire in 157,366 participants. BJPsych Open.

[28] Shungin D (2015). New genetic loci link adipose and insulin biology to body fat distribution. Nature.

[29] Ehret GB (2016). The genetics of blood pressure regulation and its target organs from association studies in 342, 415 individuals. Nat Genet.

[30] Strawbridge RJ (2011). Genome-wide association identifies nine common variants associated with fasting proinsulin levels and provides new insights into the pathophysiology of type 2 diabetes. Diabetes.

[31] Strawbridge Rona J., Silveira Angela, Hoed Marcel den, Gustafsson Stefan, Luan Jian'an, Rybin Denis, Dupuis Josée, Li-Gao Ruifang, Kavousi Maryam, Dehghan Abbas, Haljas Kadri, Lahti Jari, Gådin Jesper R., Bäcklund Alexandra, de Faire Ulf, Gertow Karl, Giral Phillipe, Goel Anuj, Humphries Steve E., Kurl Sudhir, Langenberg Claudia, Lannfelt Lars L., Lind Lars, Lindgren Cecilia C.M., Mannarino Elmo, Mook-Kanamori Dennis O., Morris Andrew P., de Mutsert Renée, Rauramaa Rainer, Saliba-Gustafsson Peter, Sennblad Bengt, Smit Andries J., Syvänen Ann-Christine, Tremoli Elena, Veglia Fabrizio, Zethelius Björn, Björck Hanna M., Eriksson Johan G., Hofman Albert, Franco Oscar H., Watkins Hugh, Jukema J. Wouter, Florez Jose C., Wareham Nicholas J., Meigs James B., Ingelsson Erik, Baldassarre Damiano, Hamsten Anders (2017). Identification of a novel proinsulin-associated SNP and demonstration that proinsulin is unlikely to be a causal factor in subclinical vascular remodelling using Mendelian randomisation. Atherosclerosis.

[32] Wallace TM, Levy JC, Matthews DR (2004). Use and abuse of HOMA modeling. Diabetes Care.

[33] Eastwood SV (2016). Algorithms for the Capture and Adjudication of Prevalent and Incident Diabetes in UK Biobank. PLoS One.

[34] Gigante B (2015). Analysis of the role of interleukin 6 receptor haplotypes in the regulation of circulating levels of inflammatory biomarkers and risk of coronary heart disease. PLoS One.

[35] Willer CJ, Li Y, Abecasis GR (2010). METAL: fast and efficient meta-analysis of genomewide association scans. Bioinformatics.

[36] Pruim RJ (2010). LocusZoom: regional visualization of genome-wide association scan results. Bioinformatics.

[37] Barrett JC, Fry B, Maller J, Daly MJ (2005). Haploview: analysis and visualization of LD and haplotype maps. Bioinformatics.

[38] McLaren W (2016). The Ensembl Variant Effect Predictor. Genome Biol.

[39] Consortium GT (2013). The Genotype-Tissue Expression (GTEx) project. Nat Genet.

[40] Locke AE (2015). Genetic studies of body mass index yield new insights for obesity biology. Nature.

[41] Berndt SI (2013). Genome-wide meta-analysis identifies 11 new loci for anthropometric traits and provides insights into genetic architecture. Nat Genet.

[42] Akiyama M (2017). Genome-wide association study identifies 112 new loci for body mass index in the Japanese population. Nat Genet.

[43] Hill W. D., Marioni R. E., Maghzian O., Ritchie S. J., Hagenaars S. P., McIntosh A. M., Gale C. R., Davies G., Deary I. J. (2018). A combined analysis of genetically correlated traits identifies 187 loci and a role for neurogenesis and myelination in intelligence. Molecular Psychiatry.

[44] Fox CS (2012). Genome-wide association for abdominal subcutaneous and visceral adipose reveals a novel locus for visceral fat in women. PLoS Genet.

[45] Justice AE (2017). Genome-wide meta-analysis of 241,258 adults accounting for smoking behaviour identifies novel loci for obesity traits. Nat Commun.

[46] Speliotes EK (2010). Association analyses of 249,796 individuals reveal 18 new loci associated with body mass index. Nat Genet.

[47] Voracek M, Loibl LM (2007). Genetics of suicide: a systematic review of twin studies. Wien Klin Wochenschr.

[48] Ferrucci L, Fabbri E (2018). Inflammageing: chronic inflammation in ageing, cardiovascular disease, and frailty. Nat Rev Cardiol.

[49] Solovieff N, Cotsapas C, Lee PH, Purcell SM, Smoller JW (2013). Pleiotropy in complex traits: challenges and strategies. Nat Rev Genet.

[50] Rathjen T (2017). Regulation of body weight and energy homeostasis by neuronal cell adhesion molecule 1. Nat Neurosci.

[51] Liu Y (2017). MicroRNA-125a-3p is involved in early behavioral disorders in stroke-afflicted rats through the regulation of Cadm2. Int J Mol Med.

[52] Frei JA, Andermatt I, Gesemann M, Stoeckli ET (2014). The SynCAM synaptic cell adhesion molecules are involved in sensory axon pathfinding by regulating axon-axon contacts. J Cell Sci.

[53] Liu, N. *et al*. CADM2 inhibits human glioma proliferation, migration and invasion. *Oncol Rep*, 10.3892/or.2019.7010 (2019). 10.3892/or.2019.7010 30816549

[54] Huang YX (2018). Downregulation of microRNA182 inhibits cell viability, invasion and angiogenesis in retinoblastoma through inhibition of the PI3K/AKT pathway and CADM2 upregulation. Int J Oncol.

[55] He W (2013). Aberrant methylation and loss of CADM2 tumor suppressor expression is associated with human renal cell carcinoma tumor progression. Biochem Biophys Res Commun.

[56] Li D (2018). CADM2, as a new target of miR-10b, promotes tumor metastasis through FAK/AKT pathway in hepatocellular carcinoma. J Exp Clin Cancer Res.

[57] Yang S (2014). Low CADM2 expression predicts high recurrence risk of hepatocellular carcinoma patients after hepatectomy. J Cancer Res Clin Oncol.

[58] He Z, Xu H, Meng Y, Kuang Y (2017). miR-944 acts as a prognostic marker and promotes the tumor progression in endometrial cancer. Biomed Pharmacother.

[59] Chang G (2010). Hypoexpression and epigenetic regulation of candidate tumor suppressor gene CADM-2 in human prostate cancer. Clin Cancer Res.

[60] Li X (2018). The CADM2/Akt pathway is involved in the inhibitory effect of miR-21-5p downregulation on proliferation and apoptosis in esophageal squamous cell carcinoma cells. Chem Biol Interact.

[61] Lundback V (2018). FAM13A and POM121C are candidate genes for fasting insulin: functional follow-up analysis of a genome-wide association study. Diabetologia.

[62] Pan A (2012). Bidirectional association between depression and obesity in middle-aged and older women. Int J Obes (Lond).

