# Supplementary material for: Genetic variation in CADM2 as a link between psychological traits and obesity
Source: Sci Rep. 2019 May 14;9:7339. doi: 10.1038/s41598-019-43861-9 (PMC6517397; doi:10.1038/s41598-019-43861-9)
Supplement: Supplementary file 1 — Supplementary data [file 41598_2019_43861_MOESM1_ESM.pdf]

Online Data Supplement

Genetic variation in *CADM2* as a link between psychological traits and obesity

Julia Morris[1], Mark E. S. Bailey[2], Damiano Baldassarre[3,4], Breda Cullen[1], Ulf de Faire[5], Amy Ferguson[1], Bruna Gigante[5,6], Philippe Giral[7], Anuj Goel[8,9], Nicholas Graham[1], Anders Hamsten[10], Steve E. Humphries[11], Keira J. A. Johnston[1,12,13], Donald M. Lyall[1], Laura M. Lyall[1], Bengt Sennblad[14], Angela Silveira[10], Andries J. Smit[15], Elena Tremoli[4,16], Fabrizio Veglia[4], Joey Ward[1], Hugh Watkins[8,9], Daniel J Smith[1] and Rona J. Strawbridge[1,10*]

Supplemental members of the IMPROVE study group

C.R. Sirtori[a], S. Castelnuovo[a], M. Amato[b], B. Frigerio[b], A. Ravani[b], D. Sansaro[b], C. Tedesco[b], A. Bonomi[b], M. Ahl[c,d], G. Blomgren[c,d], M.J. Eriksson[c,d], P. Fahlstadius[c,d], M. Heinonen[c,d], L. Nilson[c,d], J. Cooper[e], J. Acharya[e], K. Huttunen[f], E. Rauramaa[f], H Pekkarinen[f], I.M. Penttila[f], J. Törrönen[f], A.I. van Gessel[g], A.M van Roon[g], G.C. Teune[g], W.D. Kuipers[g], M. Bruin[g], A. Nicolai[g], P. Haarsma-Jorritsma[g], D.J. Mulder[g], H.J.G. Bilo[g], G.H. Smeets[g], J.L. Beaudeux[h], J.F. Kahn[h], V. Carreau[h], A. Kontush[h], J. Karppi[i], T. Nurmi[i], K. Nyyssönen[i], R. Salonen[i], T.P. Tuomainen[i], J.Tuomainen[i], J. Kauhanen[i], G. Vaudo[j], A. Alaeddin[j], D. Siepi[j], G. Lupattelli[j], G. Schillaci[j].

a) Dipartimento di Scienze Farmacologiche e Biomolecolari, Università di Milano, Milan, Italy.
b) Centro Cardiologico Monzino, IRCCS, Milan Italy.
c) Atherosclerosis Research Unit, Departments of Medicine and Cardiology, Solna, Karolinska Institutet, Stockholm, Sweden.
d) Division of Cardiovascular Epidemiology, Institute of Environmental Medicine, Karolinska Institutet, Stockholm, Sweden
e) University College of London, Department of Medicine, Rayne Institute, London, United Kingdom
f) Foundation for Research in Health Exercise and Nutrition, Kuopio Research Institute of Exercise Medicine, Kuopio, Finland.

g) Department of Medicine, University Medical Center Groningen, Groningen & Isala Clinics Zwolle, Department of Medicine; the Netherlands
h) Assistance Publique - Hopitaux de Paris; Service Endocrinologie-Metabolisme, Groupe Hôpitalier Pitie-Salpetriere, Unités de Prévention Cardiovasculaire, Paris, France.
i) Institute of Public Health and Clinical Nutrition, University of Eastern Finland, Kuopio Campus.
j) Internal Medicine, Angiology and Arteriosclerosis Diseases, Department of Clinical and Experimental Medicine, University of Perugia, Perugia, Italy.

Supplemental Methods

Population structure analysis

Population structure was assessed using multidimensional scaling, in PLINK (using default parameters as suggested by the authors of PLINK) [1]. The procedure used for UK Biobank has been described in the data release documentation [2, 3].

UK Biobank participants were genotyped using either the Affymetrix UK Biobank Axion or the Affymetrix BiLEVE Axion array [3]. A modified version of SHAPEIT2 was used for phasing and IMPUTE2 for imputation. The data from UK Biobank was released in two phases. The UK Biobank was imputed to the 1000 Genomes, UK10K haplotype (first release) and Haplotype Reference Consortium (merged with the first release for the second release) reference panels [2].

References

1. Purcell, S., et al., *PLINK: a tool set for whole-genome association and population-based linkage analyses.* Am J Hum Genet, 2007. **81**(3): p. 559-75.
2. Biobank, U., *Genotype imputation and genetic association studies of UK Biobank, Interim Data Release.* 2015. **11 September 2015**.
3. Biobank, U., *Genotyping of 500,000 UK Biobank participants. Description of sample processing workflow and preparation of DNA for genotyping.* 2015. **11 September 2015**.

Supplementary Figure Legends

Supplementary Figure 1: Locuszoom plots for results of meta-analyses of A) WHRadjBMI, B) DBP, C) T2D, D) current smoking, E) CAD.

Supplementary Figure 2: Locuszoom plots for meta-analyses results of biomarker levels: A) glucose, B) insulin, C) HOMA-B, D) HOMA-IR, E) HDL, F) LDL and G) TGs.

Supplementary Figure 3: Locuszoom plots for psychiatric and psychological phenotypes in UK Biobank: A) MDD, B) BPD, C) GAD and D) addiction.

Supplementary Figure 4: Regional plots for analyses of A) BMI, conditioned on B) BMI index SNP, C) SBP index SNP , D) CRP index SNP, E) mood instability index SNP, F) neuroticism index SNP, G) risk-taking index SNP.

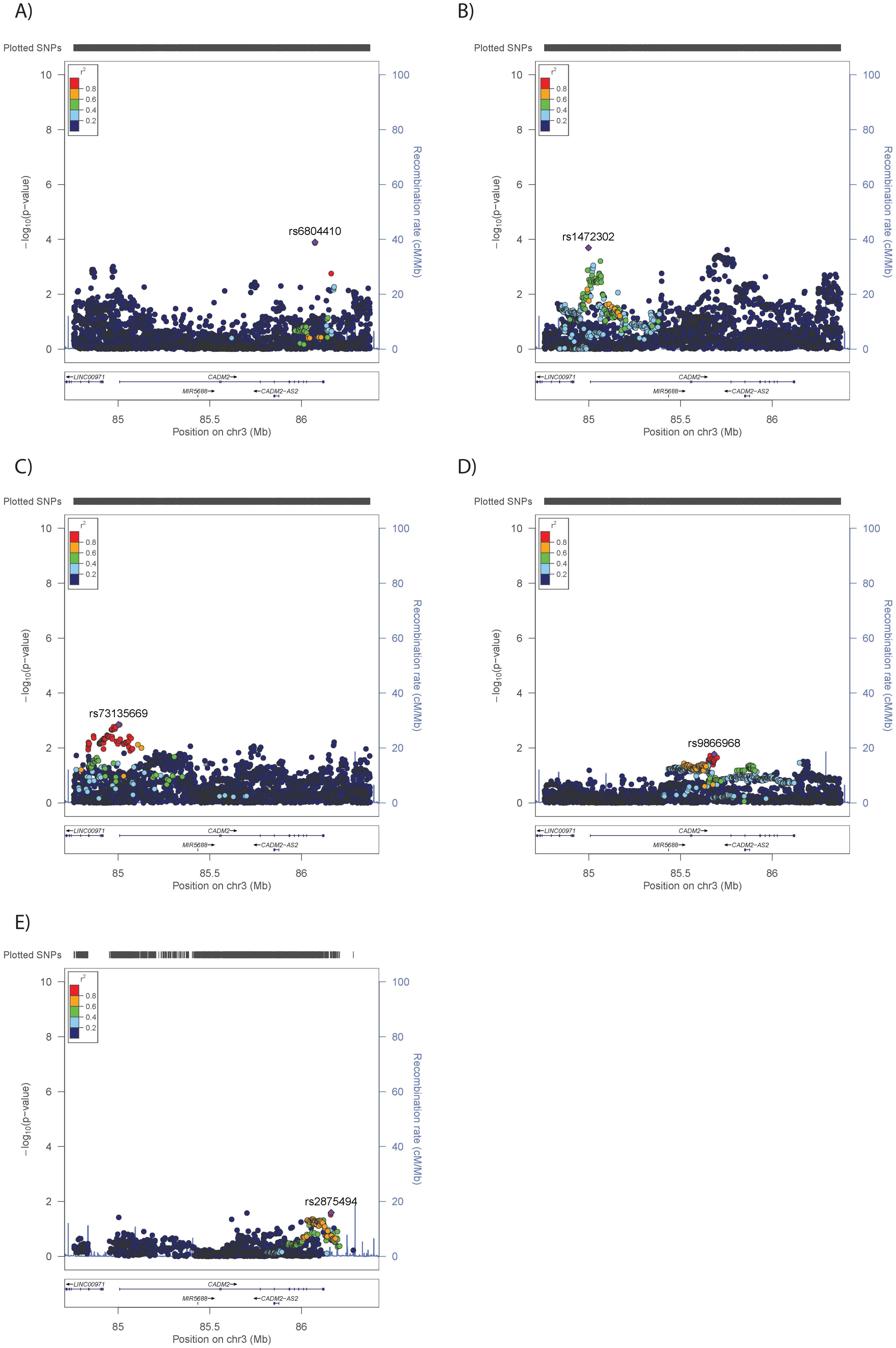
A)
B)
C)
D)
E)
Plotted SNPs
r²
0.8
0.6
0.4
0.2
−log10(p−value)
Recombination rate (cM/Mb)
rs6804410
rs1472302
rs73135669
rs9866968
rs2875494
LINC00971
CADM2
MIR5688
CADM2−AS2
85
85.5
86
Position on chr3 (Mb)

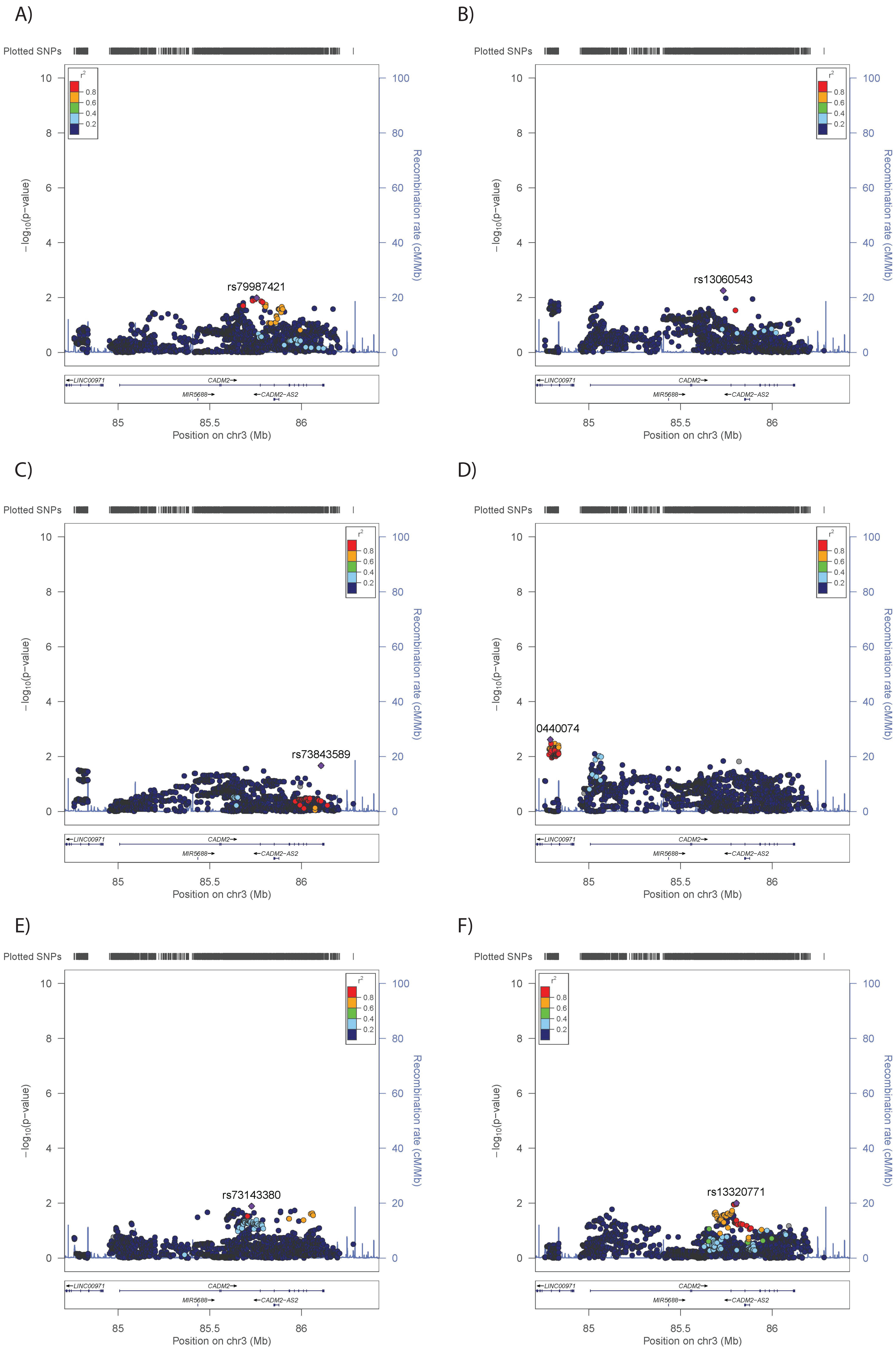

A)
B)
C)
D)
E)
F)
Plotted SNPs
rs79987421
rs13060543
rs73843589
0440074
rs73143380
rs13320771
r²
0.8
0.6
0.4
0.2
−log10(p−value)
Recombination rate (cM/Mb)
LINC00971
CADM2
MIR5688
CADM2−AS2
Position on chr3 (Mb)

G)

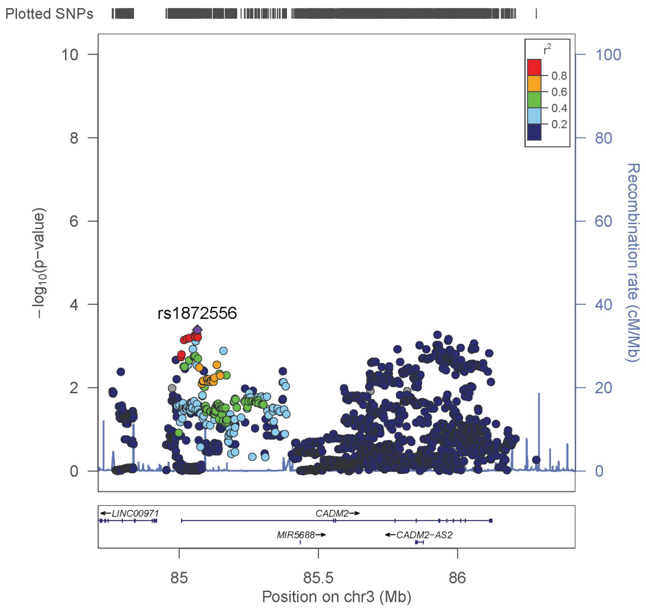
Plotted SNPs
10
8
6
4
2
0
100
80
60
40
20
0
−log10(p−value)
Recombination rate (cM/Mb)
r2
0.8
0.6
0.4
0.2
rs1872556
LINC00971
CADM2
MIR5688
CADM2−AS2
85
85.5
86
Position on chr3 (Mb)

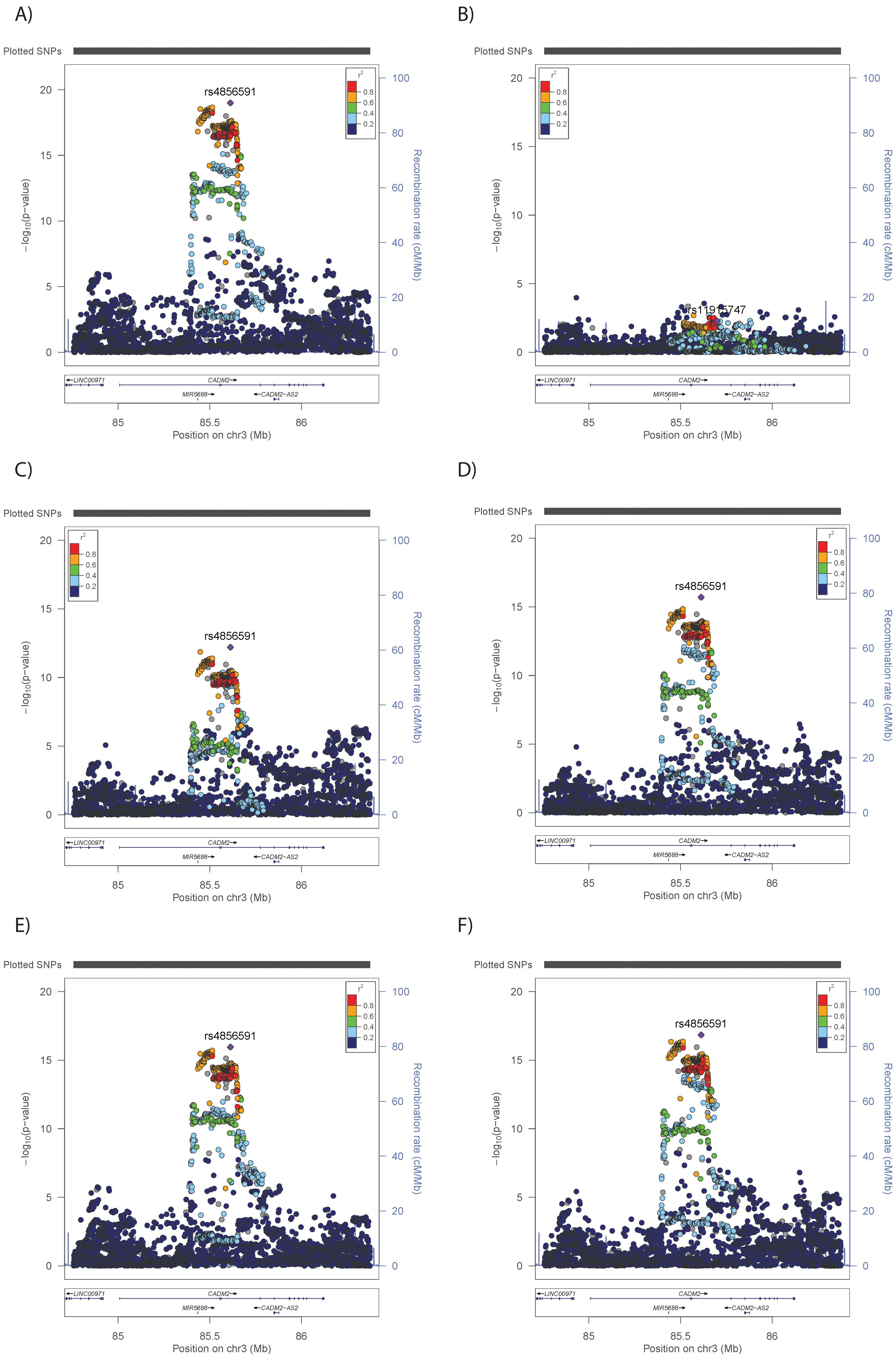
A)
Plotted SNPs
rs4856591
r²
0.8
0.6
0.4
0.2
−log10(p−value)
Recombination rate (cM/Mb)
LINC00971
CADM2
MIR5688
CADM2−AS2
85
85.5
86
Position on chr3 (Mb)
B)
Plotted SNPs
rs11915747
−log10(p−value)
Recombination rate (cM/Mb)
Position on chr3 (Mb)
C)
Plotted SNPs
rs4856591
−log10(p−value)
Recombination rate (cM/Mb)
Position on chr3 (Mb)
D)
Plotted SNPs
rs4856591
−log10(p−value)
Recombination rate (cM/Mb)
Position on chr3 (Mb)
E)
Plotted SNPs
rs4856591
−log10(p−value)
Recombination rate (cM/Mb)
Position on chr3 (Mb)
F)
Plotted SNPs
rs4856591
−log10(p−value)
Recombination rate (cM/Mb)
Position on chr3 (Mb)

G)

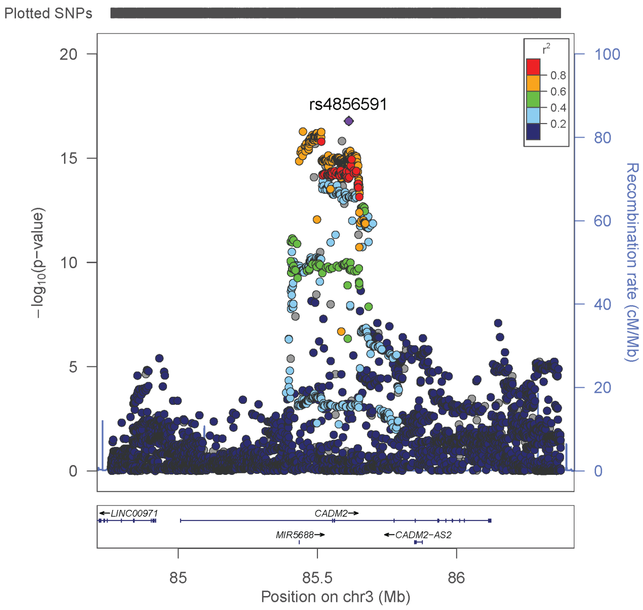

Plotted SNPs
rs4856591
r²
0.8
0.6
0.4
0.2
−log10(p−value)
Recombination rate (cM/Mb)
20
15
10
5
0
100
80
60
40
20
0
LINC00971
CADM2
MIR5688
CADM2−AS2
85
85.5
86
Position on chr3 (Mb)

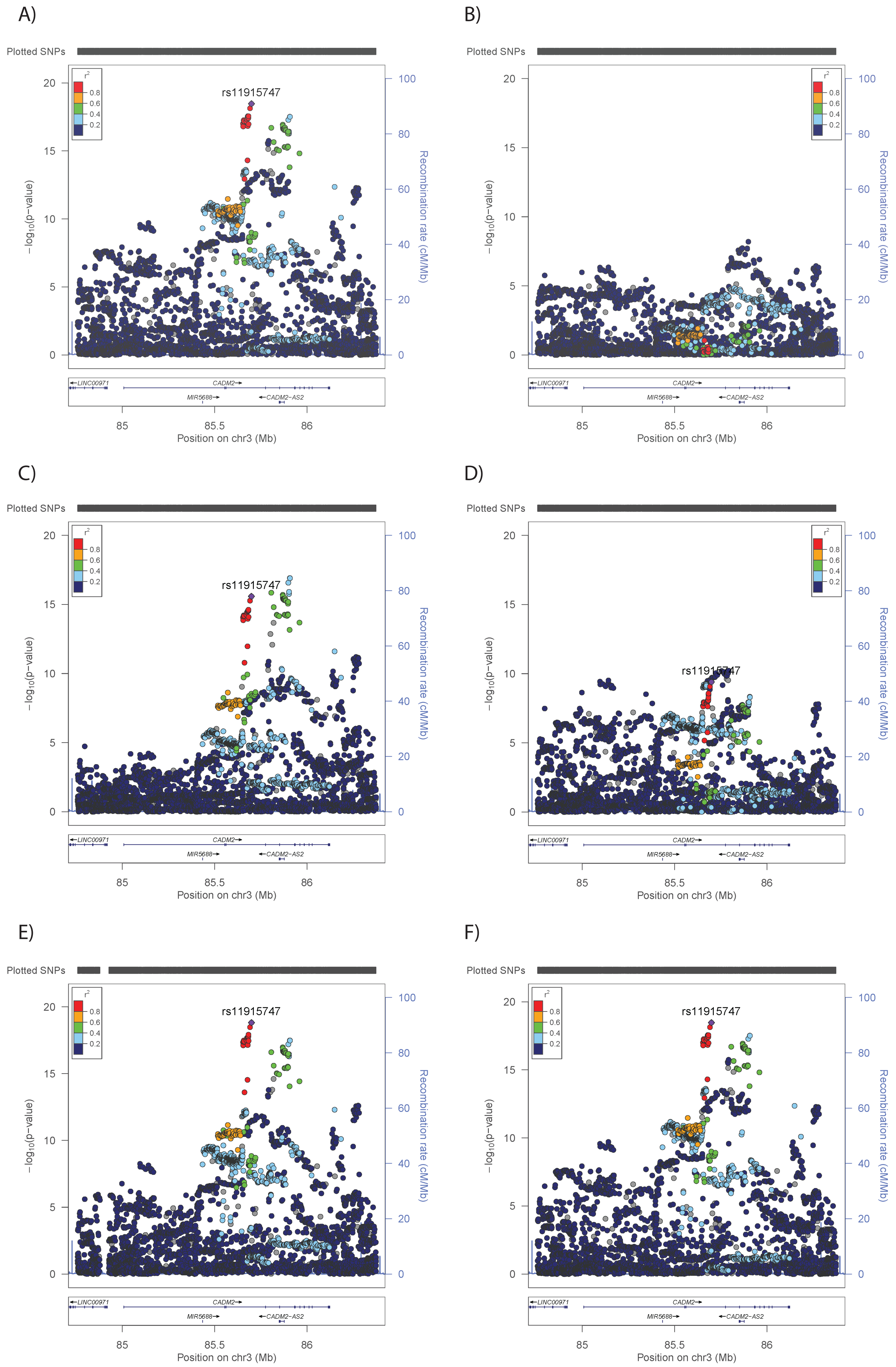
A)
Plotted SNPs
rs11915747
r2
0.8
0.6
0.4
0.2
−log10(p−value)
Recombination rate (cM/Mb)
LINC00971
CADM2
MIR5688
CADM2−AS2
85
85.5
86
Position on chr3 (Mb)
B)
Plotted SNPs
−log10(p−value)
Recombination rate (cM/Mb)
Position on chr3 (Mb)
C)
Plotted SNPs
rs11915747
−log10(p−value)
Recombination rate (cM/Mb)
Position on chr3 (Mb)
D)
Plotted SNPs
−log10(p−value)
Recombination rate (cM/Mb)
Position on chr3 (Mb)
E)
Plotted SNPs
rs11915747
−log10(p−value)
Recombination rate (cM/Mb)
Position on chr3 (Mb)
F)
Plotted SNPs
rs11915747
−log10(p−value)
Recombination rate (cM/Mb)
Position on chr3 (Mb)

G)

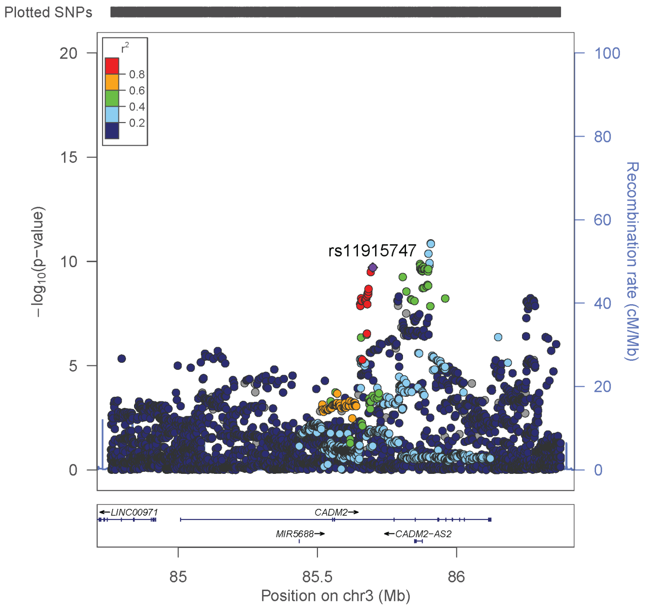

Plotted SNPs
r²
0.8
0.6
0.4
0.2
rs11915747
−log10(p−value)
Recombination rate (cM/Mb)
LINC00971
CADM2
MIR5688
CADM2−AS2
85
85.5
86
Position on chr3 (Mb)

Supplementary Table 1: Meta-analysis results for BMI and SBP

			BMI (4 possible cohorts)								SBP (4 possible cohorts)					
MarkerName	A1	A2	Freq1	Freq SE	Effect	Std Err	P	Dir	Het I2	Het P	Effect	Std Err	P	Dir	Het I2	Het P
rs11915747	c	g	**0.64**	**0.01**	**0.090**	**0.010**	**1.58E-19**	**++++**	**73**	**0.011**						
rs35623690	c	g	**0.36**	**0.01**	**-0.089**	**0.010**	**3.31E-19**	**----**	**72.8**	**0.012**						
rs10865612	t	c	**0.64**	**0.01**	**0.089**	**0.010**	**4.50E-19**	**+-++**	**70.7**	**0.017**						
rs11127908	c	g	**0.36**	**0.01**	**-0.089**	**0.010**	**6.15E-19**	**-+--**	**70.8**	**0.016**						
rs61439785	a	g	**0.64**	**0.01**	**0.088**	**0.010**	**7.54E-19**	**+-++**	**71**	**0.016**						
rs58382609	t	g	**0.64**	**0.01**	**0.088**	**0.010**	**7.61E-19**	**+-++**	**70.9**	**0.016**						
rs11914525	a	g	**0.64**	**0.01**	**0.088**	**0.010**	**1.04E-18**	**+-++**	**69.1**	**0.021**						
rs11926422	t	g	**0.64**	**0.01**	**0.088**	**0.010**	**1.06E-18**	**+-++**	**69.1**	**0.021**						
rs7431895	t	c	**0.64**	**0.01**	**0.088**	**0.010**	**1.07E-18**	**+-++**	**68.5**	**0.023**						
rs1448614	t	g	**0.64**	**0.01**	**0.088**	**0.010**	**1.19E-18**	**+-++**	**68.9**	**0.022**						
rs17023377	t	c	**0.36**	**0.01**	**-0.088**	**0.010**	**1.20E-18**	**-+--**	**69**	**0.022**						
rs9876664	t	g	**0.38**	**0.01**	**-0.087**	**0.010**	**1.26E-18**	**-+--**	**63.6**	**0.041**						
rs1375217	a	g	**0.64**	**0.01**	**0.088**	**0.010**	**1.30E-18**	**+-++**	**69.5**	**0.020**						
rs10511068	t	g	**0.64**	**0.01**	**0.088**	**0.010**	**1.32E-18**	**+-++**	**68.6**	**0.023**						
rs11714569	t	c	**0.64**	**0.01**	**0.088**	**0.010**	**1.35E-18**	**+-++**	**69.6**	**0.020**						
rs60732670	t	c	**0.64**	**0.01**	**0.088**	**0.010**	**1.39E-18**	**+-++**	**69.6**	**0.020**						
rs10511067	a	g	**0.64**	**0.01**	**0.088**	**0.010**	**1.42E-18**	**+-++**	**68.9**	**0.022**						
rs62261721	c	g	**0.64**	**0.01**	**0.088**	**0.010**	**1.46E-18**	**+-++**	**69.6**	**0.020**						
rs12631564	a	g	**0.36**	**0.01**	**-0.088**	**0.010**	**1.47E-18**	**-+--**	**68.9**	**0.022**						
rs12495178	t	c	**0.64**	**0.01**	**0.088**	**0.010**	**1.52E-18**	**+-++**	**69.4**	**0.020**						
rs9824301	a	c	**0.65**	**0.01**	**0.088**	**0.010**	**1.56E-18**	**++++**	**74.1**	**0.009**						
rs1448613	a	g	**0.36**	**0.01**	**-0.088**	**0.010**	**1.57E-18**	**-+--**	**69.7**	**0.019**						
rs12493563	t	g	**0.35**	**0.01**	**-0.089**	**0.010**	**1.68E-18**	**----**	**71.5**	**0.015**						
rs55782528	a	c	**0.36**	**0.01**	**-0.087**	**0.010**	**2.14E-18**	**-+--**	**69.6**	**0.020**						
rs12493621	c	g	**0.35**	**0.01**	**-0.088**	**0.010**	**2.21E-18**	**----**	**74.2**	**0.009**						
rs55776605	a	g	**0.64**	**0.01**	**0.087**	**0.010**	**2.23E-18**	**+-++**	**69.6**	**0.020**						
rs62261667	a	g	**0.65**	**0.01**	**0.088**	**0.010**	**2.47E-18**	**++++**	**74.5**	**0.008**						

rs62263929	t	c	**0.35**	**0.01**	**-0.088**	**0.010**	**2.99E-18**	**----**	**74.2**	**0.009**
rs6787851	t	c	**0.36**	**0.01**	**-0.087**	**0.010**	**3.03E-18**	**-+--**	**69.5**	**0.020**
rs67219198	a	g	**0.35**	**0.01**	**-0.088**	**0.010**	**3.27E-18**	**----**	**75**	**0.007**
rs62263923	a	g	**0.64**	**0.01**	**0.087**	**0.010**	**3.31E-18**	**++++**	**75.2**	**0.007**
rs9866968	a	g	**0.35**	**0.01**	**-0.088**	**0.010**	**3.74E-18**	**----**	**70.4**	**0.017**
rs55686445	t	c	**0.64**	**0.01**	**0.087**	**0.010**	**3.97E-18**	**++++**	**75.1**	**0.007**
rs66568921	t	g	**0.64**	**0.01**	**0.087**	**0.010**	**4.34E-18**	**++++**	**75.1**	**0.007**
rs62263913	c	g	**0.36**	**0.01**	**-0.087**	**0.010**	**5.36E-18**	**----**	**74.8**	**0.008**
rs9841044	t	c	**0.36**	**0.01**	**-0.087**	**0.010**	**6.61E-18**	**----**	**73.8**	**0.009**
rs73141547	a	t	**0.65**	**0.01**	**0.087**	**0.010**	**6.70E-18**	**++++**	**74.7**	**0.008**
rs1449398	a	g	**0.35**	**0.01**	**-0.087**	**0.010**	**7.83E-18**	**----**	**74.7**	**0.008**
rs1375561	t	c	**0.65**	**0.01**	**0.087**	**0.010**	**8.42E-18**	**++++**	**74.8**	**0.008**
rs62263914	c	g	**0.64**	**0.01**	**0.086**	**0.010**	**9.32E-18**	**++++**	**74.5**	**0.008**
rs62261725	a	g	**0.67**	**0.00**	**0.088**	**0.010**	**9.95E-18**	**+-++**	**76.9**	**0.005**
rs3911063	t	c	**0.68**	**0.00**	**0.088**	**0.010**	**1.11E-17**	**+-++**	**78.2**	**0.003**
rs6549058	t	c	**0.60**	**0.09**	**0.085**	**0.010**	**1.13E-17**	**++++**	**68.4**	**0.023**
rs4856605	a	g	**0.32**	**0.00**	**-0.088**	**0.010**	**1.22E-17**	**-+--**	**78.7**	**0.003**
rs7629375	a	c	**0.37**	**0.01**	**-0.085**	**0.010**	**1.26E-17**	**----**	**69.1**	**0.021**
rs3902500	a	g	**0.40**	**0.09**	**-0.085**	**0.010**	**1.32E-17**	**----**	**68.9**	**0.022**
rs7613695	a	t	**0.60**	**0.09**	**0.085**	**0.010**	**1.35E-17**	**++++**	**60.6**	**0.055**
rs3916054	t	c	**0.60**	**0.09**	**0.085**	**0.010**	**1.38E-17**	**++++**	**68.9**	**0.022**
rs2325036	a	c	**0.62**	**0.01**	**0.084**	**0.010**	**1.72E-17**	**+-++**	**64**	**0.040**
rs2875529	a	g	**0.62**	**0.01**	**0.083**	**0.010**	**5.84E-17**	**+-++**	**66.5**	**0.030**
rs6549055	a	g	**0.62**	**0.01**	**0.082**	**0.010**	**6.79E-17**	**+-++**	**65.1**	**0.035**
rs9836755	a	t	**0.38**	**0.01**	**-0.082**	**0.010**	**7.75E-17**	**-+--**	**65.2**	**0.035**
rs57153235	t	g	**0.68**	**0.00**	**0.086**	**0.010**	**1.08E-16**	**+-++**	**77.6**	**0.004**
rs57533494	a	c	**0.68**	**0.00**	**0.085**	**0.010**	**1.38E-16**	**+-++**	**77.4**	**0.004**
rs7355953	t	c	**0.79**	**0.01**	**-0.095**	**0.012**	**5.41E-16**	**----**	**48.1**	**0.123**
rs5023755	a	g	**0.66**	**0.01**	**0.085**	**0.011**	**6.09E-16**	**++++**	**68.2**	**0.024**
rs9858244	a	g	**0.21**	**0.01**	**0.095**	**0.012**	**7.39E-16**	**++++**	**46**	**0.135**

rs7612213	a	g	**0.21**	**0.01**	**0.095**	**0.012**	**8.52E-16**	**++++**	**45.6**	**0.138**
rs9825885	a	g	**0.79**	**0.01**	**-0.095**	**0.012**	**9.03E-16**	**----**	**45.5**	**0.139**
rs7644190	c	g	**0.41**	**0.08**	**-0.079**	**0.010**	**1.99E-15**	**----**	**73**	**0.011**
rs62261746	c	g	**0.70**	**0.00**	**0.081**	**0.011**	**1.17E-14**	**+-++**	**83.3**	**0.000**
rs62263917	a	g	**0.35**	**0.01**	**0.078**	**0.010**	**1.26E-14**	**++++**	**21.6**	**0.281**
rs67391933	a	g	**0.35**	**0.01**	**0.078**	**0.010**	**1.27E-14**	**++++**	**21.6**	**0.281**
rs4856600	a	c	**0.35**	**0.01**	**0.078**	**0.010**	**1.49E-14**	**++++**	**20.9**	**0.285**
rs9826759	t	c	**0.35**	**0.01**	**0.078**	**0.010**	**1.58E-14**	**++++**	**20**	**0.290**
rs7627780	a	g	**0.35**	**0.01**	**0.078**	**0.010**	**1.72E-14**	**++++**	**20.3**	**0.288**
rs9831610	t	c	**0.35**	**0.01**	**0.078**	**0.010**	**1.77E-14**	**++++**	**19.3**	**0.294**
rs9816536	a	g	**0.35**	**0.01**	**0.078**	**0.010**	**1.77E-14**	**++++**	**20.1**	**0.289**
rs6799195	t	c	**0.35**	**0.01**	**0.077**	**0.010**	**2.02E-14**	**++++**	**19.7**	**0.291**
rs11708632	a	t	**0.24**	**0.00**	**0.085**	**0.011**	**2.18E-14**	**++++**	**0**	**0.417**
rs10511073	a	g	**0.35**	**0.01**	**0.077**	**0.010**	**2.34E-14**	**++++**	**18.2**	**0.300**
rs13076821	t	g	**0.76**	**0.00**	**-0.085**	**0.011**	**2.59E-14**	**----**	**0**	**0.397**
rs9828595	t	g	**0.79**	**0.01**	**-0.090**	**0.012**	**2.60E-14**	**----**	**0**	**0.586**
rs11928368	t	g	**0.33**	**0.01**	**-0.078**	**0.010**	**2.65E-14**	**----**	**69.6**	**0.020**
rs62263916	t	g	**0.65**	**0.01**	**-0.077**	**0.010**	**2.73E-14**	**----**	**20.8**	**0.285**
rs4473564	a	g	**0.21**	**0.01**	**0.090**	**0.012**	**2.73E-14**	**++++**	**0**	**0.478**
rs12714636	a	g	**0.24**	**0.00**	**0.085**	**0.011**	**2.74E-14**	**++++**	**0**	**0.439**
rs1448608	a	g	**0.24**	**0.00**	**0.085**	**0.011**	**2.80E-14**	**++++**	**0**	**0.441**
rs11923343	a	g	**0.35**	**0.01**	**0.077**	**0.010**	**2.91E-14**	**++++**	**20.5**	**0.287**
rs62263918	a	g	**0.35**	**0.01**	**0.077**	**0.010**	**2.97E-14**	**++++**	**20**	**0.290**
rs71626890	a	c	**0.24**	**0.00**	**0.085**	**0.011**	**3.04E-14**	**++++**	**0**	**0.432**
rs12637027	c	g	**0.35**	**0.01**	**0.076**	**0.010**	**3.27E-14**	**++++**	**19.9**	**0.291**
rs1448602	a	g	**0.76**	**0.00**	**-0.085**	**0.011**	**3.32E-14**	**----**	**0**	**0.439**
rs17521052	a	g	**0.35**	**0.01**	**0.076**	**0.010**	**3.39E-14**	**++++**	**19.8**	**0.291**
rs13087042	a	g	**0.24**	**0.00**	**0.085**	**0.011**	**3.46E-14**	**++++**	**8.9**	**0.348**
rs6549046	t	g	**0.65**	**0.01**	**-0.076**	**0.010**	**3.63E-14**	**----**	**19.5**	**0.293**
rs1449400	a	t	**0.65**	**0.01**	**-0.076**	**0.010**	**3.65E-14**	**----**	**19.4**	**0.293**

rs6779752	a	g	**0.65**	**0.01**	**-0.076**	**0.010**	**3.67E-14**	**----**	**19.6**	**0.292**
rs7632056	a	g	**0.35**	**0.01**	**0.076**	**0.010**	**3.69E-14**	**++++**	**19.3**	**0.293**
rs6549045	c	g	**0.35**	**0.01**	**0.076**	**0.010**	**3.71E-14**	**++++**	**19.6**	**0.292**
rs1375564	t	c	**0.65**	**0.01**	**-0.076**	**0.010**	**4.10E-14**	**----**	**18.6**	**0.298**
rs62263915	t	c	**0.65**	**0.01**	**-0.076**	**0.010**	**4.22E-14**	**----**	**19**	**0.296**
rs2122042	t	g	**0.20**	**0.01**	**0.089**	**0.012**	**5.52E-14**	**++++**	**0**	**0.426**
rs12714637	t	g	**0.80**	**0.01**	**-0.090**	**0.012**	**5.82E-14**	**----**	**0**	**0.425**
rs9818122	t	c	**0.80**	**0.01**	**-0.089**	**0.012**	**6.39E-14**	**----**	**0**	**0.432**
rs7620339	a	g	**0.20**	**0.01**	**0.089**	**0.012**	**6.49E-14**	**++++**	**0**	**0.432**
rs7620168	t	c	**0.20**	**0.01**	**0.089**	**0.012**	**6.87E-14**	**++++**	**0**	**0.431**
rs7611991	a	g	**0.25**	**0.00**	**0.083**	**0.011**	**7.73E-14**	**++++**	**0**	**0.511**
rs4129299	a	g	**0.76**	**0.00**	**-0.083**	**0.011**	**8.50E-14**	**----**	**23**	**0.273**
rs11915638	a	g	**0.79**	**0.01**	**-0.087**	**0.012**	**9.75E-14**	**----**	**0**	**0.562**
rs11721040	a	c	**0.80**	**0.01**	**-0.088**	**0.012**	**1.03E-13**	**----**	**0**	**0.415**
rs4444725	t	c	**0.80**	**0.01**	**-0.088**	**0.012**	**1.09E-13**	**----**	**0**	**0.414**
rs13096280	a	g	**0.76**	**0.00**	**-0.083**	**0.011**	**1.14E-13**	**----**	**21.3**	**0.282**
rs4464471	a	g	**0.24**	**0.00**	**0.083**	**0.011**	**1.38E-13**	**++++**	**13.6**	**0.324**
rs13068138	t	c	**0.20**	**0.01**	**0.089**	**0.012**	**1.39E-13**	**++++**	**0**	**0.441**
rs73138150	a	t	**0.68**	**0.00**	**0.083**	**0.011**	**1.44E-13**	**+++?**	**57.5**	**0.095**
rs9834708	a	g	**0.24**	**0.00**	**0.083**	**0.011**	**1.51E-13**	**++++**	**13.2**	**0.327**
rs4306872	a	g	**0.24**	**0.00**	**0.083**	**0.011**	**1.52E-13**	**++++**	**13.4**	**0.326**
rs7640660	t	c	**0.20**	**0.01**	**0.087**	**0.012**	**1.68E-13**	**++++**	**0**	**0.426**
rs9816144	a	g	**0.80**	**0.01**	**-0.087**	**0.012**	**1.69E-13**	**----**	**0**	**0.426**
rs10049108	t	g	**0.24**	**0.00**	**0.083**	**0.011**	**1.69E-13**	**++++**	**12.7**	**0.329**
rs13083873	a	g	**0.24**	**0.00**	**0.083**	**0.011**	**1.70E-13**	**++++**	**12.7**	**0.329**
rs13101056	t	g	**0.24**	**0.00**	**0.083**	**0.011**	**1.71E-13**	**++++**	**11.6**	**0.335**
rs9309986	t	c	**0.76**	**0.00**	**-0.082**	**0.011**	**1.74E-13**	**----**	**12.5**	**0.330**
rs9840636	a	c	**0.76**	**0.00**	**-0.082**	**0.011**	**1.75E-13**	**----**	**12.6**	**0.330**
rs1597213	c	g	**0.20**	**0.01**	**0.087**	**0.012**	**1.86E-13**	**++++**	**0**	**0.435**
rs9309990	a	g	**0.20**	**0.01**	**0.088**	**0.012**	**2.18E-13**	**++++**	**0**	**0.457**

rs13078960	t	g	**0.80**	**0.01**	**-0.088**	**0.012**	**2.63E-13**	**----**	**0**	**0.452**
rs13097865	a	g	**0.76**	**0.00**	**-0.082**	**0.011**	**2.64E-13**	**----**	**10.5**	**0.340**
rs9880272	t	g	**0.76**	**0.00**	**-0.082**	**0.011**	**2.76E-13**	**----**	**10.3**	**0.342**
rs9309991	c	g	**0.80**	**0.01**	**-0.088**	**0.012**	**2.91E-13**	**----**	**0**	**0.472**
rs9852859	t	c	**0.80**	**0.01**	**-0.088**	**0.012**	**2.94E-13**	**----**	**0**	**0.471**
rs13085840	t	c	**0.80**	**0.01**	**-0.088**	**0.012**	**2.98E-13**	**----**	**0**	**0.474**
rs9824971	a	t	**0.80**	**0.01**	**-0.088**	**0.012**	**3.00E-13**	**----**	**0**	**0.475**
rs7622475	t	c	**0.80**	**0.01**	**-0.088**	**0.012**	**3.01E-13**	**----**	**0**	**0.473**
rs13064386	t	g	**0.20**	**0.01**	**0.087**	**0.012**	**3.02E-13**	**++++**	**0**	**0.476**
rs9875880	a	g	**0.80**	**0.01**	**-0.088**	**0.012**	**3.07E-13**	**----**	**0**	**0.470**
rs9869320	t	c	**0.20**	**0.01**	**0.087**	**0.012**	**3.12E-13**	**++++**	**0**	**0.474**
rs9820014	a	g	**0.80**	**0.01**	**-0.087**	**0.012**	**3.15E-13**	**----**	**0**	**0.474**
rs1512929	t	c	**0.61**	**0.00**	**-0.078**	**0.011**	**3.18E-13**	**---?**	**0**	**0.880**
rs9852127	a	g	**0.20**	**0.01**	**0.087**	**0.012**	**3.19E-13**	**++++**	**0**	**0.472**
rs13098327	a	g	**0.20**	**0.01**	**0.087**	**0.012**	**3.22E-13**	**++++**	**0**	**0.474**
rs17355368	a	g	**0.20**	**0.01**	**0.087**	**0.012**	**3.22E-13**	**++++**	**0**	**0.508**
rs9852478	a	g	**0.20**	**0.01**	**0.087**	**0.012**	**3.22E-13**	**++++**	**0**	**0.475**
rs9309994	t	c	**0.80**	**0.01**	**-0.087**	**0.012**	**3.23E-13**	**----**	**0**	**0.511**
rs35806405	a	g	**0.80**	**0.01**	**-0.087**	**0.012**	**3.33E-13**	**----**	**0**	**0.479**
rs13064170	a	g	**0.80**	**0.01**	**-0.087**	**0.012**	**3.36E-13**	**----**	**0**	**0.479**
rs7640855	a	g	**0.20**	**0.01**	**0.087**	**0.012**	**3.47E-13**	**++++**	**0**	**0.506**
rs11926524	a	g	**0.20**	**0.01**	**0.087**	**0.012**	**3.54E-13**	**++++**	**0**	**0.486**
rs9873441	t	g	**0.20**	**0.01**	**0.087**	**0.012**	**3.76E-13**	**++++**	**0**	**0.484**
rs1448612	a	g	**0.80**	**0.01**	**-0.087**	**0.012**	**3.82E-13**	**----**	**0**	**0.500**
rs1877512	t	c	**0.61**	**0.00**	**-0.078**	**0.011**	**3.91E-13**	**---?**	**0**	**0.845**
rs9826482	a	g	**0.20**	**0.01**	**0.087**	**0.012**	**3.91E-13**	**++++**	**0**	**0.501**
rs13077697	a	g	**0.39**	**0.00**	**0.078**	**0.011**	**4.00E-13**	**+++?**	**0**	**0.855**
rs13078807	a	g	**0.80**	**0.01**	**-0.087**	**0.012**	**4.11E-13**	**----**	**0**	**0.518**
rs13099202	t	c	**0.39**	**0.00**	**0.078**	**0.011**	**4.31E-13**	**+++?**	**0**	**0.854**
rs12714640	a	c	**0.20**	**0.01**	**0.087**	**0.012**	**4.98E-13**	**++++**	**0**	**0.492**

rs9837525	t	c	**0.20**	**0.01**	**0.086**	**0.012**	**5.62E-13**	**++++**	**0**	**0.494**
rs6773947	a	t	**0.39**	**0.00**	**0.077**	**0.011**	**5.65E-13**	**+++?**	**0**	**0.902**
rs1512914	t	g	**0.61**	**0.00**	**-0.077**	**0.011**	**6.30E-13**	**---?**	**0**	**0.901**
rs7653219	a	g	**0.39**	**0.00**	**0.077**	**0.011**	**6.30E-13**	**+++?**	**0**	**0.845**
rs12487215	a	g	**0.39**	**0.00**	**0.077**	**0.011**	**6.67E-13**	**+++?**	**0**	**0.904**
rs9813495	a	g	**0.80**	**0.01**	**-0.086**	**0.012**	**6.90E-13**	**----**	**0**	**0.495**
rs28475482	t	c	**0.76**	**0.01**	**-0.081**	**0.011**	**7.04E-13**	**----**	**0**	**0.477**
rs2048589	t	c	**0.37**	**0.00**	**0.078**	**0.011**	**7.12E-13**	**+++?**	**0**	**0.883**
rs1949197	a	g	**0.39**	**0.00**	**0.077**	**0.011**	**7.15E-13**	**+++?**	**0**	**0.865**
rs9813436	a	g	**0.76**	**0.00**	**-0.081**	**0.011**	**7.51E-13**	**----**	**0**	**0.419**
rs2048591	t	c	**0.63**	**0.00**	**-0.077**	**0.011**	**7.65E-13**	**---?**	**0**	**0.883**
rs7428796	t	c	**0.39**	**0.00**	**0.076**	**0.011**	**8.06E-13**	**+++?**	**0**	**0.907**
rs13090253	a	g	**0.80**	**0.01**	**-0.087**	**0.012**	**8.25E-13**	**----**	**0**	**0.455**
rs35326689	a	g	**0.76**	**0.00**	**-0.080**	**0.011**	**8.57E-13**	**----**	**25.4**	**0.259**
rs62252461	a	g	**0.36**	**0.00**	**-0.078**	**0.011**	**9.06E-13**	**---?**	**71.5**	**0.030**
rs4688890	c	g	**0.39**	**0.00**	**0.076**	**0.011**	**1.01E-12**	**+++?**	**0**	**0.939**
rs4396896	a	t	**0.39**	**0.00**	**0.076**	**0.011**	**1.06E-12**	**+++?**	**0**	**0.888**
rs9819476	a	c	**0.76**	**0.00**	**-0.080**	**0.011**	**1.14E-12**	**----**	**0**	**0.441**
rs2137491	c	g	**0.39**	**0.00**	**0.076**	**0.011**	**1.17E-12**	**+++?**	**0**	**0.898**
rs35117966	a	g	**0.61**	**0.00**	**-0.077**	**0.011**	**1.26E-12**	**---?**	**0**	**0.874**
rs2137492	a	g	**0.37**	**0.00**	**0.077**	**0.011**	**1.33E-12**	**+++?**	**0**	**0.880**
rs4073109	t	c	**0.37**	**0.00**	**0.076**	**0.011**	**2.12E-12**	**+++?**	**0**	**0.884**
rs35384579	c	g	**0.62**	**0.00**	**-0.076**	**0.011**	**2.27E-12**	**---?**	**0**	**0.734**
rs2326309	t	g	**0.37**	**0.01**	**0.070**	**0.010**	**2.40E-12**	**++++**	**20.4**	**0.287**
rs2326310	a	g	**0.63**	**0.01**	**-0.070**	**0.010**	**2.47E-12**	**----**	**20.3**	**0.288**
rs11922956	a	g	**0.37**	**0.01**	**0.069**	**0.010**	**3.13E-12**	**++++**	**15.2**	**0.316**
rs2082556	a	g	**0.62**	**0.01**	**-0.069**	**0.010**	**3.18E-12**	**----**	**18.7**	**0.297**
rs1897699	a	t	**0.38**	**0.01**	**0.069**	**0.010**	**3.28E-12**	**++++**	**18.7**	**0.297**
rs1821351	t	c	**0.63**	**0.01**	**-0.069**	**0.010**	**3.50E-12**	**----**	**22**	**0.278**
rs4856572	t	c	**0.38**	**0.01**	**0.069**	**0.010**	**3.56E-12**	**++++**	**19.3**	**0.294**

rs1865251	a	c	**0.38**	**0.01**	**0.069**	**0.010**	**3.60E-12**	**++++**	**19.2**	**0.294**
rs4856571	c	g	**0.62**	**0.01**	**-0.069**	**0.010**	**3.62E-12**	**----**	**17.7**	**0.302**
rs1549979	t	c	**0.62**	**0.01**	**-0.068**	**0.010**	**3.73E-12**	**----**	**19.4**	**0.293**
rs1865250	t	c	**0.38**	**0.01**	**0.069**	**0.010**	**3.86E-12**	**++++**	**18.7**	**0.297**
rs13061681	c	g	**0.76**	**0.00**	**-0.078**	**0.011**	**3.87E-12**	**----**	**33.7**	**0.210**
rs60311538	t	g	**0.38**	**0.01**	**0.069**	**0.010**	**3.90E-12**	**++++**	**17.7**	**0.302**
rs7636243	t	c	**0.63**	**0.01**	**-0.069**	**0.010**	**3.92E-12**	**----**	**22.8**	**0.274**
rs35894540	t	c	**0.38**	**0.01**	**0.069**	**0.010**	**3.92E-12**	**++++**	**18.5**	**0.298**
rs62250687	a	t	**0.63**	**0.01**	**-0.069**	**0.010**	**4.00E-12**	**----**	**19.3**	**0.294**
rs11929525	t	c	**0.38**	**0.01**	**0.069**	**0.010**	**4.01E-12**	**++++**	**18**	**0.301**
rs1433708	t	c	**0.37**	**0.01**	**0.069**	**0.010**	**4.13E-12**	**++++**	**19.5**	**0.292**
rs6762267	a	c	**0.62**	**0.01**	**-0.069**	**0.010**	**4.14E-12**	**----**	**18.3**	**0.299**
rs2196099	c	g	**0.63**	**0.01**	**-0.069**	**0.010**	**4.17E-12**	**----**	**19.2**	**0.294**
rs62250712	t	c	**0.62**	**0.01**	**-0.069**	**0.010**	**4.17E-12**	**----**	**20.6**	**0.287**
rs11918899	a	g	**0.38**	**0.01**	**0.068**	**0.010**	**4.17E-12**	**++++**	**18.9**	**0.296**
rs35827242	a	g	**0.62**	**0.01**	**-0.069**	**0.010**	**4.19E-12**	**----**	**19.1**	**0.295**
rs2217720	t	g	**0.63**	**0.01**	**-0.069**	**0.010**	**4.21E-12**	**----**	**19.5**	**0.293**
rs9863488	a	g	**0.63**	**0.01**	**-0.069**	**0.010**	**4.21E-12**	**----**	**19.2**	**0.294**
rs1865252	a	g	**0.37**	**0.01**	**0.069**	**0.010**	**4.22E-12**	**++++**	**18.6**	**0.297**
rs6762695	a	g	**0.38**	**0.01**	**0.069**	**0.010**	**4.23E-12**	**++++**	**16.3**	**0.310**
rs4856573	a	g	**0.62**	**0.01**	**-0.069**	**0.010**	**4.26E-12**	**----**	**17.1**	**0.306**
rs2117153	a	g	**0.62**	**0.01**	**-0.069**	**0.010**	**4.36E-12**	**----**	**16.2**	**0.310**
rs7627044	a	g	**0.62**	**0.01**	**-0.069**	**0.010**	**4.36E-12**	**----**	**18.8**	**0.297**
rs10511084	a	g	**0.37**	**0.00**	**-0.075**	**0.011**	**4.41E-12**	**---?**	**67.2**	**0.047**
rs993716	t	c	**0.62**	**0.01**	**-0.069**	**0.010**	**4.46E-12**	**----**	**16**	**0.311**
rs993136	t	c	**0.38**	**0.01**	**0.069**	**0.010**	**4.46E-12**	**++++**	**16.2**	**0.311**
rs12629036	t	c	**0.38**	**0.01**	**0.069**	**0.010**	**4.46E-12**	**++++**	**18.8**	**0.297**
rs1433714	t	g	**0.38**	**0.01**	**0.069**	**0.010**	**4.50E-12**	**++++**	**16.2**	**0.311**
rs6774985	t	c	**0.38**	**0.01**	**0.069**	**0.010**	**4.51E-12**	**++++**	**16.4**	**0.310**
rs12637461	t	g	**0.62**	**0.01**	**-0.069**	**0.010**	**4.51E-12**	**----**	**17.9**	**0.301**

rs62250711	a	t	**0.38**	**0.01**	**0.069**	**0.010**	**4.55E-12**	**++++**	**16.7**	**0.308**
rs12638482	t	c	**0.62**	**0.01**	**-0.068**	**0.010**	**4.56E-12**	**----**	**17.8**	**0.302**
rs62250686	a	t	**0.62**	**0.01**	**-0.074**	**0.011**	**4.59E-12**	**---?**	**0**	**0.836**
rs4856569	a	g	**0.38**	**0.01**	**0.068**	**0.010**	**4.59E-12**	**++++**	**19.8**	**0.291**
rs57630146	a	g	**0.62**	**0.01**	**-0.069**	**0.010**	**4.61E-12**	**----**	**20.4**	**0.288**
rs6808400	t	c	**0.37**	**0.01**	**0.069**	**0.010**	**4.73E-12**	**++++**	**19.3**	**0.293**
rs6780968	c	g	**0.37**	**0.01**	**0.068**	**0.010**	**4.93E-12**	**++++**	**18.4**	**0.299**
rs56063967	a	c	**0.63**	**0.00**	**0.074**	**0.011**	**4.95E-12**	**+++?**	**70.2**	**0.035**
rs7638953	a	g	**0.63**	**0.01**	**-0.069**	**0.010**	**4.97E-12**	**----**	**18.3**	**0.299**
rs13082188	t	c	**0.76**	**0.00**	**-0.077**	**0.011**	**5.01E-12**	**----**	**43.7**	**0.149**
rs62250685	a	g	**0.38**	**0.01**	**0.068**	**0.010**	**5.04E-12**	**++++**	**18.2**	**0.300**
rs62252460	a	g	**0.63**	**0.00**	**0.074**	**0.011**	**5.25E-12**	**+++?**	**67.7**	**0.045**
rs2053108	a	c	**0.63**	**0.01**	**-0.068**	**0.010**	**5.26E-12**	**----**	**18.9**	**0.296**
rs993137	t	c	**0.62**	**0.01**	**-0.069**	**0.010**	**5.35E-12**	**----**	**17.1**	**0.306**
rs62250759	a	g	**0.63**	**0.00**	**0.074**	**0.011**	**5.44E-12**	**+++?**	**68.2**	**0.043**
rs1368742	a	g	**0.38**	**0.01**	**0.068**	**0.010**	**5.45E-12**	**++++**	**14.3**	**0.321**
rs2122236	t	g	**0.63**	**0.00**	**0.074**	**0.011**	**5.48E-12**	**+++?**	**60.7**	**0.079**
rs190777270	t	g	**0.37**	**0.00**	**-0.074**	**0.011**	**5.49E-12**	**---?**	**73.5**	**0.023**
rs17022938	a	t	**0.63**	**0.00**	**0.074**	**0.011**	**5.52E-12**	**+++?**	**70.2**	**0.035**
rs9836967	a	c	**0.24**	**0.00**	**0.077**	**0.011**	**5.54E-12**	**++++**	**43.3**	**0.152**
rs7643091	a	g	**0.24**	**0.00**	**0.077**	**0.011**	**5.57E-12**	**++++**	**38.2**	**0.183**
rs1449388	a	g	**0.63**	**0.00**	**0.074**	**0.011**	**5.61E-12**	**+++?**	**67.9**	**0.044**
rs2033526	a	c	**0.38**	**0.00**	**0.069**	**0.010**	**5.67E-12**	**++++**	**12.1**	**0.332**
rs7650284	t	c	**0.37**	**0.01**	**0.068**	**0.010**	**5.84E-12**	**++++**	**17.8**	**0.302**
rs9880010	t	c	**0.24**	**0.00**	**0.077**	**0.011**	**5.91E-12**	**++++**	**43.5**	**0.151**
rs11920184	a	g	**0.63**	**0.00**	**0.074**	**0.011**	**5.93E-12**	**+++?**	**67.5**	**0.046**
rs1449396	a	g	**0.24**	**0.00**	**0.077**	**0.011**	**6.07E-12**	**++++**	**43.4**	**0.151**
rs1449395	a	c	**0.24**	**0.00**	**0.077**	**0.011**	**6.15E-12**	**++++**	**43.4**	**0.151**
rs68102233	a	g	**0.37**	**0.00**	**-0.074**	**0.011**	**6.17E-12**	**---?**	**68**	**0.044**
rs12636904	a	c	**0.63**	**0.00**	**0.074**	**0.011**	**6.17E-12**	**+++?**	**65.8**	**0.054**

rs1375559	t	c	**0.37**	**0.00**	**-0.074**	**0.011**	**6.21E-12**	**---?**	**68**	**0.044**
rs7609594	a	g	**0.63**	**0.01**	**-0.068**	**0.010**	**6.35E-12**	**----**	**18.2**	**0.300**
rs10511087	a	g	**0.38**	**0.01**	**0.068**	**0.010**	**6.40E-12**	**++++**	**14.7**	**0.318**
rs62252504	t	c	**0.37**	**0.00**	**-0.074**	**0.011**	**6.58E-12**	**---?**	**68.8**	**0.040**
rs2167048	a	g	**0.63**	**0.00**	**0.074**	**0.011**	**6.71E-12**	**+++?**	**68.3**	**0.043**
rs112523595	t	c	**0.37**	**0.00**	**0.070**	**0.010**	**6.78E-12**	**++++**	**26.1**	**0.255**
rs66963835	a	c	**0.37**	**0.00**	**-0.074**	**0.011**	**7.00E-12**	**---?**	**68.5**	**0.042**
rs62252499	a	g	**0.38**	**0.02**	**0.074**	**0.011**	**7.02E-12**	**+++?**	**36.2**	**0.209**
rs62250755	a	c	**0.37**	**0.00**	**-0.074**	**0.011**	**7.15E-12**	**---?**	**66.7**	**0.050**
rs7624108	t	g	**0.37**	**0.00**	**-0.074**	**0.011**	**7.20E-12**	**---?**	**66.7**	**0.050**
rs10433500	a	g	**0.64**	**0.01**	**-0.069**	**0.010**	**7.27E-12**	**----**	**9.8**	**0.344**
rs9812845	a	c	**0.76**	**0.00**	**-0.077**	**0.011**	**7.42E-12**	**----**	**42.6**	**0.156**
rs12635683	a	g	**0.24**	**0.00**	**0.077**	**0.011**	**7.45E-12**	**++++**	**42.6**	**0.156**
rs62250724	t	c	**0.37**	**0.00**	**-0.074**	**0.011**	**7.45E-12**	**---?**	**66.3**	**0.052**
rs7637879	t	c	**0.63**	**0.00**	**0.074**	**0.011**	**7.46E-12**	**+++?**	**66.6**	**0.050**
rs11127903	t	g	**0.24**	**0.00**	**0.077**	**0.011**	**7.49E-12**	**++++**	**42.8**	**0.155**
rs9309985	c	g	**0.24**	**0.00**	**0.077**	**0.011**	**7.49E-12**	**++++**	**42.6**	**0.156**
rs7626594	a	g	**0.37**	**0.00**	**-0.074**	**0.011**	**7.50E-12**	**---?**	**67.1**	**0.048**
rs17022915	a	g	**0.37**	**0.00**	**-0.074**	**0.011**	**7.78E-12**	**---?**	**68.4**	**0.042**
rs58834564	t	c	**0.63**	**0.00**	**0.074**	**0.011**	**7.86E-12**	**+++?**	**67**	**0.049**
rs1991872	a	g	**0.62**	**0.01**	**-0.067**	**0.010**	**8.03E-12**	**----**	**17.3**	**0.305**
rs12637798	a	g	**0.38**	**0.01**	**0.067**	**0.010**	**8.33E-12**	**++++**	**17.2**	**0.305**
rs112911909	a	g	**0.36**	**0.01**	**-0.074**	**0.011**	**8.45E-12**	**---?**	**71.2**	**0.031**
rs17022886	a	g	**0.37**	**0.00**	**-0.073**	**0.011**	**8.86E-12**	**---?**	**66.8**	**0.049**
rs67874563	t	g	**0.37**	**0.00**	**-0.074**	**0.011**	**9.16E-12**	**---?**	**67.2**	**0.048**
rs12632140	t	c	**0.37**	**0.00**	**-0.073**	**0.011**	**9.34E-12**	**---?**	**67**	**0.048**
rs67174538	t	c	**0.63**	**0.00**	**0.073**	**0.011**	**1.01E-11**	**+++?**	**67.8**	**0.045**
rs1433715	a	c	**0.62**	**0.01**	**-0.068**	**0.010**	**1.11E-11**	**----**	**11.9**	**0.333**
rs62250717	c	g	**0.63**	**0.00**	**0.073**	**0.011**	**1.16E-11**	**+++?**	**66.3**	**0.052**
rs960986	t	c	**0.36**	**0.00**	**-0.073**	**0.011**	**1.26E-11**	**---?**	**62.9**	**0.067**

rs62250713	a	g	**0.36**	**0.00**	**0.068**	**0.010**	**1.30E-11**	**++++**	**11.2**	**0.337**
rs62252500	t	c	**0.62**	**0.02**	**-0.073**	**0.011**	**1.42E-11**	**---?**	**0**	**0.494**
rs7614148	a	g	**0.63**	**0.01**	**-0.068**	**0.010**	**1.47E-11**	**----**	**4.4**	**0.371**
rs9867437	a	c	**0.53**	**0.03**	**0.065**	**0.010**	**1.49E-11**	**+-++**	**63**	**0.044**
rs17515196	a	g	**0.63**	**0.01**	**-0.068**	**0.010**	**1.59E-11**	**----**	**11.6**	**0.335**
rs11708024	a	g	**0.30**	**0.01**	**-0.071**	**0.011**	**1.60E-11**	**-+--**	**72.5**	**0.012**
rs72615728	c	g	**0.37**	**0.01**	**0.069**	**0.010**	**1.67E-11**	**++++**	**30.9**	**0.227**
rs67336646	a	t	**0.63**	**0.01**	**-0.067**	**0.010**	**1.70E-11**	**----**	**11.1**	**0.337**
rs17516546	a	g	**0.37**	**0.01**	**0.067**	**0.010**	**1.73E-11**	**++++**	**31.2**	**0.225**
rs11127896	t	c	**0.63**	**0.01**	**-0.068**	**0.010**	**1.73E-11**	**----**	**11.9**	**0.333**
rs73130364	t	c	**0.38**	**0.00**	**0.068**	**0.010**	**1.75E-11**	**++++**	**37.6**	**0.187**
rs73130366	a	g	**0.62**	**0.00**	**-0.068**	**0.010**	**1.76E-11**	**----**	**37.5**	**0.187**
rs1542248	a	g	**0.63**	**0.01**	**-0.067**	**0.010**	**1.76E-11**	**----**	**12.8**	**0.329**
rs62250750	t	c	**0.37**	**0.01**	**0.067**	**0.010**	**1.76E-11**	**++++**	**16.4**	**0.310**
rs7621381	c	g	**0.37**	**0.01**	**0.067**	**0.010**	**1.77E-11**	**++++**	**12.9**	**0.328**
rs11127895	t	g	**0.37**	**0.01**	**0.067**	**0.010**	**1.79E-11**	**++++**	**11.9**	**0.333**
rs57872438	a	g	**0.63**	**0.01**	**-0.067**	**0.010**	**1.84E-11**	**----**	**12.7**	**0.329**
rs62250719	t	g	**0.63**	**0.01**	**-0.067**	**0.010**	**1.96E-11**	**----**	**4.2**	**0.372**
rs2069123	t	g	**0.37**	**0.01**	**0.067**	**0.010**	**2.00E-11**	**++++**	**10.3**	**0.341**
rs9713905	a	g	**0.37**	**0.00**	**-0.072**	**0.011**	**2.04E-11**	**-+-?**	**70.9**	**0.032**
rs7618429	a	g	**0.37**	**0.01**	**0.067**	**0.010**	**2.11E-11**	**++++**	**15.4**	**0.315**
rs10511085	a	c	**0.63**	**0.01**	**-0.067**	**0.010**	**2.18E-11**	**----**	**10.3**	**0.341**
rs55897272	a	g	**0.63**	**0.00**	**-0.067**	**0.010**	**2.32E-11**	**----**	**9.4**	**0.346**
rs1551043	t	g	**0.63**	**0.01**	**-0.067**	**0.010**	**2.33E-11**	**----**	**14.8**	**0.318**
rs1368750	t	c	**0.37**	**0.01**	**0.067**	**0.010**	**2.36E-11**	**++++**	**9.8**	**0.344**
rs62250754	t	g	**0.63**	**0.01**	**-0.067**	**0.010**	**2.39E-11**	**----**	**15.9**	**0.312**
rs1155666	t	c	**0.37**	**0.01**	**0.067**	**0.010**	**2.40E-11**	**++++**	**9.9**	**0.344**
rs1449391	t	g	**0.63**	**0.01**	**-0.067**	**0.010**	**2.40E-11**	**----**	**15.6**	**0.314**
rs4426693	a	g	**0.63**	**0.01**	**0.067**	**0.010**	**2.56E-11**	**+-++**	**74.8**	**0.008**
rs1449390	a	g	**0.63**	**0.01**	**-0.067**	**0.010**	**2.64E-11**	**----**	**20.9**	**0.285**

rs12495758	a	g	**0.65**	**0.01**	**-0.073**	**0.011**	**2.91E-11**	**---?**	**0**	**0.700**
rs12486239	a	g	**0.64**	**0.00**	**-0.069**	**0.010**	**3.00E-11**	**----**	**30.6**	**0.229**
rs1470636	a	c	**0.37**	**0.01**	**0.067**	**0.010**	**3.07E-11**	**++++**	**17.8**	**0.302**
rs10511082	t	c	**0.63**	**0.01**	**0.066**	**0.010**	**3.22E-11**	**++++**	**74.1**	**0.009**
rs2077839	t	c	**0.65**	**0.01**	**-0.068**	**0.010**	**3.29E-11**	**----**	**0**	**0.436**
rs34495106	a	g	**0.36**	**0.01**	**0.067**	**0.010**	**3.35E-11**	**++++**	**25**	**0.261**
rs62250758	t	g	**0.63**	**0.01**	**-0.066**	**0.010**	**3.37E-11**	**----**	**18.4**	**0.299**
rs10511083	a	g	**0.37**	**0.01**	**0.066**	**0.010**	**3.38E-11**	**++++**	**18.2**	**0.300**
rs1947221	a	t	**0.63**	**0.01**	**-0.066**	**0.010**	**3.39E-11**	**----**	**23.8**	**0.268**
rs4856598	a	c	**0.36**	**0.01**	**0.067**	**0.010**	**3.39E-11**	**++++**	**21**	**0.284**
rs1813696	a	g	**0.35**	**0.01**	**0.068**	**0.010**	**3.39E-11**	**++++**	**0**	**0.433**
rs57755423	a	c	**0.35**	**0.01**	**0.068**	**0.010**	**3.40E-11**	**++++**	**0**	**0.424**
rs62250464	t	c	**0.63**	**0.01**	**0.066**	**0.010**	**3.40E-11**	**++++**	**73.5**	**0.010**
rs6764988	a	c	**0.37**	**0.01**	**0.066**	**0.010**	**3.41E-11**	**++++**	**18.1**	**0.300**
rs67416405	t	c	**0.35**	**0.01**	**0.068**	**0.010**	**3.42E-11**	**++++**	**0**	**0.423**
rs62250714	a	g	**0.63**	**0.01**	**-0.066**	**0.010**	**3.44E-11**	**----**	**9.9**	**0.344**
rs2029130	a	t	**0.63**	**0.01**	**-0.066**	**0.010**	**3.47E-11**	**----**	**25.7**	**0.257**
rs113351222	a	c	**0.37**	**0.00**	**0.066**	**0.010**	**3.48E-11**	**++++**	**27.3**	**0.248**
rs11127893	t	c	**0.65**	**0.01**	**-0.067**	**0.010**	**3.48E-11**	**----**	**0**	**0.440**
rs62250715	a	g	**0.63**	**0.01**	**-0.066**	**0.010**	**3.50E-11**	**----**	**9.8**	**0.344**
rs1375560	t	g	**0.63**	**0.01**	**-0.066**	**0.010**	**3.51E-11**	**----**	**18.3**	**0.299**
rs7618124	a	g	**0.35**	**0.01**	**0.067**	**0.010**	**3.52E-11**	**++++**	**4**	**0.373**
rs62252464	a	g	**0.37**	**0.01**	**0.066**	**0.010**	**3.55E-11**	**++++**	**17.1**	**0.306**
rs62252462	a	g	**0.63**	**0.01**	**-0.066**	**0.010**	**3.58E-11**	**----**	**19.8**	**0.291**
rs17515586	a	g	**0.37**	**0.01**	**0.066**	**0.010**	**3.60E-11**	**++++**	**19**	**0.295**
rs7647981	a	g	**0.35**	**0.01**	**0.067**	**0.010**	**3.60E-11**	**++++**	**0**	**0.439**
rs1449394	t	c	**0.63**	**0.01**	**-0.066**	**0.010**	**3.61E-11**	**----**	**18.2**	**0.300**
rs62252467	c	g	**0.63**	**0.01**	**-0.066**	**0.010**	**3.62E-11**	**----**	**21**	**0.284**
rs62252495	a	g	**0.63**	**0.01**	**-0.066**	**0.010**	**3.64E-11**	**----**	**18.6**	**0.298**
rs1972992	t	c	**0.37**	**0.01**	**-0.066**	**0.010**	**3.66E-11**	**----**	**70.4**	**0.017**

rs62252463	a	g	**0.63**	**0.01**	**-0.066**	**0.010**	**3.66E-11**	**----**	**19.4**	**0.293**
rs58828944	t	c	**0.37**	**0.01**	**0.066**	**0.010**	**3.66E-11**	**++++**	**19.4**	**0.293**
rs35438712	t	c	**0.37**	**0.01**	**0.066**	**0.010**	**3.66E-11**	**++++**	**5.7**	**0.364**
rs4355295	a	t	**0.63**	**0.01**	**0.066**	**0.010**	**3.67E-11**	**++++**	**72**	**0.013**
rs62252466	a	g	**0.37**	**0.01**	**0.066**	**0.010**	**3.68E-11**	**++++**	**19.5**	**0.293**
rs35738543	t	g	**0.37**	**0.01**	**0.066**	**0.010**	**3.69E-11**	**++++**	**19.9**	**0.290**
rs1868532	t	c	**0.63**	**0.01**	**-0.066**	**0.010**	**3.69E-11**	**----**	**18**	**0.301**
rs66505109	a	c	**0.37**	**0.01**	**0.066**	**0.010**	**3.70E-11**	**++++**	**20.1**	**0.290**
rs17456263	a	c	**0.63**	**0.01**	**-0.066**	**0.010**	**3.70E-11**	**----**	**19.9**	**0.291**
rs4856583	a	g	**0.37**	**0.01**	**0.066**	**0.010**	**3.73E-11**	**++++**	**20.1**	**0.289**
rs56262138	a	t	**0.30**	**0.00**	**-0.076**	**0.012**	**3.74E-11**	**---?**	**0**	**0.408**
rs6804845	a	c	**0.37**	**0.01**	**0.066**	**0.010**	**3.74E-11**	**++++**	**15.4**	**0.315**
rs59225869	a	g	**0.37**	**0.01**	**0.066**	**0.010**	**3.75E-11**	**++++**	**19.9**	**0.291**
rs60427790	t	c	**0.37**	**0.01**	**0.066**	**0.010**	**3.76E-11**	**++++**	**19.9**	**0.290**
rs56778912	t	c	**0.63**	**0.01**	**-0.066**	**0.010**	**3.77E-11**	**----**	**20**	**0.290**
rs17516256	a	g	**0.63**	**0.01**	**-0.066**	**0.010**	**3.79E-11**	**----**	**18.9**	**0.296**
rs7614552	a	t	**0.65**	**0.01**	**-0.067**	**0.010**	**3.80E-11**	**----**	**0**	**0.403**
rs7628237	a	g	**0.37**	**0.01**	**0.066**	**0.010**	**3.81E-11**	**++++**	**20**	**0.290**
rs6549030	t	c	**0.35**	**0.01**	**0.067**	**0.010**	**3.89E-11**	**++++**	**0**	**0.396**
rs57671246	c	g	**0.37**	**0.01**	**0.066**	**0.010**	**3.89E-11**	**++++**	**19.6**	**0.292**
rs12638798	t	c	**0.35**	**0.01**	**0.067**	**0.010**	**3.90E-11**	**++++**	**0**	**0.392**
rs62250540	t	c	**0.63**	**0.01**	**-0.067**	**0.010**	**3.92E-11**	**----**	**16.5**	**0.309**
rs4856580	a	t	**0.63**	**0.01**	**-0.066**	**0.010**	**3.94E-11**	**----**	**19.2**	**0.294**
rs4856579	t	c	**0.37**	**0.01**	**0.066**	**0.010**	**3.94E-11**	**++++**	**19.2**	**0.294**
rs1449393	t	c	**0.63**	**0.01**	**-0.066**	**0.010**	**3.95E-11**	**----**	**19.3**	**0.294**
rs59825726	a	g	**0.63**	**0.01**	**-0.066**	**0.010**	**3.95E-11**	**----**	**19.1**	**0.295**
rs73137895	a	g	**0.63**	**0.01**	**0.066**	**0.010**	**3.97E-11**	**++++**	**74.6**	**0.008**
rs56777875	a	g	**0.63**	**0.01**	**-0.066**	**0.010**	**3.97E-11**	**----**	**19.7**	**0.291**
rs62253963	a	g	**0.37**	**0.01**	**-0.067**	**0.010**	**3.99E-11**	**----**	**72.1**	**0.013**
rs62252497	t	c	**0.63**	**0.01**	**-0.066**	**0.010**	**4.00E-11**	**----**	**21.7**	**0.280**

rs10433499	t	c	**0.65**	**0.01**	**-0.067**	**0.010**	**4.02E-11**	**----**	**0**	**0.402**
rs4508797	a	g	**0.37**	**0.01**	**-0.066**	**0.010**	**4.03E-11**	**----**	**74.6**	**0.008**
rs10433525	t	c	**0.35**	**0.01**	**0.067**	**0.010**	**4.05E-11**	**++++**	**0**	**0.403**
rs2875907	a	g	**0.35**	**0.01**	**0.067**	**0.010**	**4.08E-11**	**++++**	**0**	**0.443**
rs56187922	a	c	**0.63**	**0.01**	**-0.066**	**0.010**	**4.10E-11**	**----**	**18.4**	**0.299**
rs62252465	t	c	**0.63**	**0.01**	**-0.066**	**0.010**	**4.12E-11**	**----**	**16.6**	**0.308**
rs4856586	t	g	**0.37**	**0.00**	**0.066**	**0.010**	**4.13E-11**	**++++**	**10.3**	**0.342**
rs1375545	t	g	**0.63**	**0.01**	**-0.066**	**0.010**	**4.16E-11**	**----**	**27.3**	**0.248**
rs62250471	a	c	**0.37**	**0.01**	**-0.066**	**0.010**	**4.19E-11**	**----**	**73.4**	**0.010**
rs72615725	t	g	**0.37**	**0.01**	**-0.066**	**0.010**	**4.20E-11**	**-+--**	**74.5**	**0.008**
rs1551044	t	c	**0.35**	**0.01**	**0.067**	**0.010**	**4.21E-11**	**++++**	**0**	**0.404**
rs6549034	c	g	**0.37**	**0.01**	**0.066**	**0.010**	**4.23E-11**	**++++**	**9.3**	**0.346**
rs62252507	t	c	**0.63**	**0.01**	**-0.066**	**0.010**	**4.25E-11**	**----**	**20**	**0.290**
rs17516284	a	g	**0.63**	**0.01**	**-0.066**	**0.010**	**4.25E-11**	**----**	**20.9**	**0.285**
rs62263912	a	g	**0.63**	**0.01**	**-0.066**	**0.010**	**4.29E-11**	**----**	**23.8**	**0.268**
rs56779213	a	g	**0.37**	**0.01**	**0.066**	**0.010**	**4.31E-11**	**++++**	**19.6**	**0.292**
rs17516580	c	g	**0.63**	**0.01**	**-0.066**	**0.010**	**4.32E-11**	**----**	**23.4**	**0.270**
rs11923525	a	g	**0.63**	**0.00**	**-0.066**	**0.010**	**4.33E-11**	**----**	**25.7**	**0.258**
rs59770976	a	g	**0.37**	**0.01**	**0.066**	**0.010**	**4.34E-11**	**++++**	**19.4**	**0.293**
rs10511075	a	g	**0.63**	**0.01**	**-0.066**	**0.010**	**4.34E-11**	**----**	**19.4**	**0.293**
rs57756567	a	t	**0.63**	**0.01**	**-0.066**	**0.010**	**4.34E-11**	**----**	**19.5**	**0.292**
rs17516346	a	g	**0.37**	**0.01**	**0.066**	**0.010**	**4.36E-11**	**++++**	**19.4**	**0.293**
rs58796643	a	t	**0.37**	**0.01**	**0.066**	**0.010**	**4.36E-11**	**++++**	**17.2**	**0.305**
rs12637791	t	g	**0.35**	**0.01**	**0.067**	**0.010**	**4.37E-11**	**++++**	**0**	**0.444**
rs10511076	a	g	**0.37**	**0.01**	**0.066**	**0.010**	**4.37E-11**	**++++**	**19.4**	**0.293**
rs956281	t	g	**0.37**	**0.01**	**0.067**	**0.010**	**4.40E-11**	**++++**	**23.9**	**0.268**
rs4637303	t	c	**0.65**	**0.01**	**-0.067**	**0.010**	**4.42E-11**	**----**	**0**	**0.402**
rs66625173	a	g	**0.37**	**0.01**	**0.066**	**0.010**	**4.42E-11**	**++++**	**23.3**	**0.271**
rs62252506	t	c	**0.37**	**0.01**	**0.066**	**0.010**	**4.43E-11**	**++++**	**28**	**0.244**
rs62263910	t	g	**0.64**	**0.01**	**-0.067**	**0.010**	**4.45E-11**	**----**	**10.4**	**0.341**

rs4856275	a	c	**0.63**	**0.00**	**-0.066**	**0.010**	**4.48E-11**	**----**	**14.5**	**0.320**
rs4856587	t	g	**0.63**	**0.00**	**-0.066**	**0.010**	**4.50E-11**	**----**	**1.8**	**0.383**
rs1375546	a	g	**0.63**	**0.01**	**-0.065**	**0.010**	**4.51E-11**	**----**	**26.9**	**0.250**
rs6797840	a	c	**0.47**	**0.03**	**-0.064**	**0.010**	**4.56E-11**	**-+--**	**63.4**	**0.042**
rs17457050	t	c	**0.63**	**0.01**	**-0.066**	**0.010**	**4.58E-11**	**----**	**18.4**	**0.299**
rs55939743	a	g	**0.63**	**0.01**	**-0.066**	**0.010**	**4.60E-11**	**----**	**20.3**	**0.288**
rs17022974	t	c	**0.63**	**0.01**	**0.066**	**0.010**	**4.61E-11**	**+-++**	**73.9**	**0.009**
rs4856581	a	g	**0.63**	**0.01**	**-0.066**	**0.010**	**4.61E-11**	**----**	**18.6**	**0.298**
rs67544605	a	t	**0.37**	**0.01**	**0.066**	**0.010**	**4.66E-11**	**++++**	**19.5**	**0.293**
rs1470635	t	c	**0.63**	**0.01**	**-0.066**	**0.010**	**4.80E-11**	**----**	**18.9**	**0.296**
rs59967234	a	g	**0.37**	**0.01**	**0.066**	**0.010**	**4.81E-11**	**++++**	**19.1**	**0.295**
rs7427346	t	c	**0.37**	**0.01**	**0.066**	**0.010**	**4.82E-11**	**++++**	**19**	**0.295**
rs62252496	a	g	**0.63**	**0.01**	**-0.066**	**0.010**	**4.83E-11**	**----**	**17.7**	**0.302**
rs34467301	t	c	**0.37**	**0.01**	**0.066**	**0.010**	**4.85E-11**	**++++**	**11.8**	**0.334**
rs62252501	a	g	**0.38**	**0.01**	**0.066**	**0.010**	**4.91E-11**	**++++**	**28.6**	**0.241**
rs62250463	t	g	**0.37**	**0.01**	**0.065**	**0.010**	**4.91E-11**	**++++**	**26**	**0.256**
rs11927145	a	g	**0.63**	**0.01**	**0.066**	**0.010**	**4.92E-11**	**++++**	**73.9**	**0.009**
rs1449389	a	g	**0.37**	**0.01**	**0.066**	**0.010**	**4.96E-11**	**++++**	**19.1**	**0.295**
rs62253961	t	c	**0.63**	**0.01**	**-0.066**	**0.010**	**5.01E-11**	**----**	**8.7**	**0.350**
rs60597959	a	t	**0.63**	**0.01**	**-0.066**	**0.010**	**5.03E-11**	**----**	**8.9**	**0.349**
rs1449402	a	g	**0.65**	**0.01**	**-0.067**	**0.010**	**5.07E-11**	**----**	**30**	**0.233**
rs17457189	a	g	**0.63**	**0.01**	**-0.066**	**0.010**	**5.10E-11**	**----**	**14.1**	**0.322**
rs2167047	a	t	**0.37**	**0.01**	**0.066**	**0.010**	**5.12E-11**	**++++**	**12.8**	**0.328**
rs17457217	a	t	**0.37**	**0.01**	**0.066**	**0.010**	**5.15E-11**	**++++**	**12.3**	**0.331**
rs62252503	c	g	**0.63**	**0.01**	**-0.066**	**0.010**	**5.18E-11**	**----**	**19.7**	**0.292**
rs62252505	t	c	**0.63**	**0.01**	**-0.066**	**0.010**	**5.23E-11**	**----**	**19.7**	**0.291**
rs1900916	t	g	**0.35**	**0.01**	**0.067**	**0.010**	**5.28E-11**	**++++**	**28.9**	**0.238**
rs67874038	t	c	**0.63**	**0.01**	**0.065**	**0.010**	**5.30E-11**	**++++**	**74.2**	**0.009**
rs1449410	c	g	**0.37**	**0.01**	**0.065**	**0.010**	**5.30E-11**	**++++**	**26.2**	**0.255**
rs73133800	t	c	**0.63**	**0.01**	**-0.065**	**0.010**	**5.32E-11**	**----**	**26.5**	**0.253**

rs35701422	t	c	**0.37**	**0.01**	**0.066**	**0.010**	**5.33E-11**	**++++**	**24.5**	**0.264**
rs78288623	a	c	**0.63**	**0.00**	**-0.066**	**0.010**	**5.34E-11**	**----**	**22.5**	**0.276**
rs2029133	a	g	**0.63**	**0.01**	**-0.065**	**0.010**	**5.35E-11**	**----**	**25.9**	**0.256**
rs2029132	a	c	**0.63**	**0.01**	**-0.065**	**0.010**	**5.35E-11**	**----**	**25.9**	**0.256**
rs62252509	t	c	**0.63**	**0.01**	**-0.066**	**0.010**	**5.38E-11**	**----**	**19.2**	**0.294**
rs77852438	t	c	**0.63**	**0.00**	**-0.066**	**0.010**	**5.42E-11**	**----**	**23**	**0.273**
rs1900918	a	c	**0.63**	**0.01**	**-0.065**	**0.010**	**5.43E-11**	**----**	**19.6**	**0.292**
rs58919842	a	g	**0.63**	**0.01**	**-0.066**	**0.010**	**5.48E-11**	**----**	**18.7**	**0.297**
rs17456763	t	c	**0.63**	**0.01**	**-0.066**	**0.010**	**5.49E-11**	**----**	**18.8**	**0.297**
rs67018424	a	g	**0.63**	**0.01**	**-0.066**	**0.010**	**5.49E-11**	**----**	**18.8**	**0.297**
rs59491876	c	g	**0.37**	**0.01**	**0.066**	**0.010**	**5.51E-11**	**++++**	**18.8**	**0.296**
rs17456820	a	t	**0.63**	**0.01**	**-0.066**	**0.010**	**5.52E-11**	**----**	**18.9**	**0.296**
rs2044723	a	g	**0.35**	**0.01**	**0.067**	**0.010**	**5.54E-11**	**++++**	**29.4**	**0.236**
rs72615726	a	g	**0.63**	**0.01**	**-0.065**	**0.010**	**5.56E-11**	**----**	**22.2**	**0.278**
rs2122235	t	c	**0.63**	**0.01**	**-0.065**	**0.010**	**5.70E-11**	**----**	**12.4**	**0.331**
rs1449401	t	c	**0.65**	**0.01**	**-0.067**	**0.010**	**5.70E-11**	**----**	**29.6**	**0.235**
rs66781790	a	g	**0.37**	**0.01**	**0.065**	**0.010**	**5.73E-11**	**++++**	**13.7**	**0.324**
rs62252513	t	c	**0.63**	**0.01**	**-0.065**	**0.010**	**5.77E-11**	**----**	**12.4**	**0.331**
rs17516857	a	c	**0.37**	**0.01**	**0.065**	**0.010**	**5.79E-11**	**++++**	**12.5**	**0.330**
rs4856271	a	g	**0.37**	**0.01**	**0.065**	**0.010**	**5.81E-11**	**++++**	**14.5**	**0.320**
rs6766493	a	g	**0.63**	**0.01**	**-0.065**	**0.010**	**5.82E-11**	**----**	**14.7**	**0.319**
rs4856276	a	t	**0.37**	**0.00**	**0.065**	**0.010**	**5.85E-11**	**++++**	**0**	**0.432**
rs73133799	t	c	**0.37**	**0.01**	**0.065**	**0.010**	**5.85E-11**	**++++**	**29.4**	**0.236**
rs17460541	t	g	**0.63**	**0.01**	**-0.065**	**0.010**	**5.88E-11**	**----**	**24.5**	**0.264**
rs17457377	t	g	**0.37**	**0.01**	**0.065**	**0.010**	**5.91E-11**	**++++**	**14**	**0.322**
rs62252519	a	t	**0.63**	**0.01**	**-0.065**	**0.010**	**5.93E-11**	**----**	**14.1**	**0.322**
rs1375544	a	g	**0.37**	**0.01**	**0.065**	**0.010**	**5.96E-11**	**++++**	**25.6**	**0.258**
rs35608699	a	t	**0.63**	**0.00**	**-0.066**	**0.010**	**5.97E-11**	**----**	**22.2**	**0.277**
rs66631011	a	g	**0.63**	**0.01**	**-0.065**	**0.010**	**5.99E-11**	**----**	**21.6**	**0.281**
rs62252518	a	t	**0.37**	**0.01**	**0.065**	**0.010**	**5.99E-11**	**++++**	**14**	**0.322**

rs726610	t	c	**0.66**	**0.01**	**-0.067**	**0.010**	**6.01E-11**	**----**	**0**	**0.398**
rs67568006	t	g	**0.63**	**0.01**	**-0.065**	**0.010**	**6.10E-11**	**----**	**25**	**0.261**
rs17517080	c	g	**0.37**	**0.01**	**0.065**	**0.010**	**6.13E-11**	**++++**	**17**	**0.306**
rs1375567	a	g	**0.63**	**0.01**	**0.065**	**0.010**	**6.16E-11**	**++++**	**73.3**	**0.011**
rs77657121	a	g	**0.63**	**0.01**	**-0.066**	**0.010**	**6.19E-11**	**----**	**27.7**	**0.246**
rs17455991	t	c	**0.35**	**0.01**	**0.066**	**0.010**	**6.19E-11**	**++++**	**3.2**	**0.377**
rs17516470	c	g	**0.37**	**0.01**	**0.065**	**0.010**	**6.25E-11**	**++++**	**18.5**	**0.298**
rs17516504	t	c	**0.37**	**0.01**	**0.065**	**0.010**	**6.25E-11**	**++++**	**18.4**	**0.298**
rs66922686	a	t	**0.37**	**0.01**	**0.065**	**0.010**	**6.32E-11**	**++++**	**19**	**0.295**
rs6779501	t	c	**0.37**	**0.01**	**0.065**	**0.010**	**6.33E-11**	**++++**	**14.4**	**0.320**
rs17023016	a	c	**0.63**	**0.01**	**0.065**	**0.010**	**6.33E-11**	**++++**	**74.3**	**0.009**
rs17517238	c	g	**0.63**	**0.01**	**-0.065**	**0.010**	**6.35E-11**	**----**	**19**	**0.295**
rs1992967	a	g	**0.65**	**0.01**	**-0.066**	**0.010**	**6.35E-11**	**----**	**4.7**	**0.370**
rs17517142	a	c	**0.37**	**0.01**	**0.065**	**0.010**	**6.35E-11**	**++++**	**18.9**	**0.296**
rs6790090	a	t	**0.63**	**0.01**	**-0.065**	**0.010**	**6.37E-11**	**----**	**19.4**	**0.293**
rs1463206	a	t	**0.63**	**0.01**	**-0.065**	**0.010**	**6.42E-11**	**----**	**22.6**	**0.275**
rs7637527	t	c	**0.37**	**0.01**	**0.065**	**0.010**	**6.46E-11**	**++++**	**22.1**	**0.278**
rs10865611	c	g	**0.65**	**0.01**	**-0.066**	**0.010**	**6.48E-11**	**----**	**28.7**	**0.240**
rs17516683	a	g	**0.63**	**0.01**	**-0.065**	**0.010**	**6.50E-11**	**----**	**18.2**	**0.300**
rs12491722	t	c	**0.35**	**0.01**	**0.066**	**0.010**	**6.50E-11**	**++++**	**5.8**	**0.364**
rs6777456	t	g	**0.63**	**0.01**	**-0.065**	**0.010**	**6.53E-11**	**----**	**18.2**	**0.299**
rs6772956	c	g	**0.63**	**0.01**	**-0.065**	**0.010**	**6.54E-11**	**----**	**16.6**	**0.309**
rs62250465	t	c	**0.63**	**0.01**	**-0.065**	**0.010**	**6.57E-11**	**----**	**25**	**0.261**
rs62252512	a	t	**0.37**	**0.01**	**0.065**	**0.010**	**6.58E-11**	**++++**	**18.3**	**0.299**
rs11919099	t	g	**0.63**	**0.01**	**-0.065**	**0.010**	**6.67E-11**	**----**	**22.2**	**0.277**
rs68049270	t	c	**0.63**	**0.01**	**-0.065**	**0.010**	**6.68E-11**	**----**	**25.4**	**0.259**
rs17518584	t	c	**0.63**	**0.01**	**-0.065**	**0.010**	**6.70E-11**	**----**	**25.3**	**0.260**
rs62250504	a	g	**0.37**	**0.01**	**-0.065**	**0.010**	**6.72E-11**	**----**	**74.6**	**0.008**
rs11127897	t	c	**0.35**	**0.01**	**0.066**	**0.010**	**6.74E-11**	**++++**	**0**	**0.397**
rs1449375	t	g	**0.63**	**0.01**	**-0.065**	**0.010**	**6.78E-11**	**----**	**28.6**	**0.241**

rs1449372	t	g	**0.37**	**0.01**	**0.065**	**0.010**	**6.79E-11**	**++++**	**28.5**	**0.241**
rs1551045	a	c	**0.37**	**0.01**	**0.065**	**0.010**	**6.85E-11**	**++++**	**19.6**	**0.292**
rs1551047	t	g	**0.63**	**0.01**	**-0.065**	**0.010**	**6.86E-11**	**----**	**19.7**	**0.291**
rs62250502	a	g	**0.37**	**0.01**	**0.065**	**0.010**	**6.88E-11**	**++++**	**22.3**	**0.277**
rs1463207	t	c	**0.63**	**0.01**	**-0.065**	**0.010**	**6.93E-11**	**----**	**20.1**	**0.289**
rs1597315	t	c	**0.63**	**0.01**	**-0.065**	**0.010**	**6.93E-11**	**----**	**14.2**	**0.321**
rs1551048	t	g	**0.63**	**0.01**	**-0.065**	**0.010**	**6.95E-11**	**----**	**19.9**	**0.290**
rs17457454	t	c	**0.37**	**0.01**	**0.065**	**0.010**	**6.97E-11**	**++++**	**13.5**	**0.325**
rs61586601	a	t	**0.37**	**0.01**	**0.065**	**0.010**	**6.97E-11**	**++++**	**16.3**	**0.310**
rs1463205	a	g	**0.63**	**0.01**	**-0.065**	**0.010**	**6.98E-11**	**----**	**20.1**	**0.289**
rs1449373	a	g	**0.63**	**0.01**	**-0.065**	**0.010**	**7.02E-11**	**----**	**28.7**	**0.240**
rs1449374	t	c	**0.63**	**0.01**	**-0.065**	**0.010**	**7.02E-11**	**----**	**28.7**	**0.240**
rs17457426	a	c	**0.37**	**0.01**	**0.065**	**0.010**	**7.02E-11**	**++++**	**13.3**	**0.326**
rs62250491	t	g	**0.63**	**0.01**	**-0.065**	**0.010**	**7.02E-11**	**----**	**24.1**	**0.267**
rs6549033	a	t	**0.37**	**0.01**	**0.065**	**0.010**	**7.04E-11**	**++++**	**19.2**	**0.294**
rs62252516	t	c	**0.63**	**0.01**	**-0.065**	**0.010**	**7.05E-11**	**----**	**13.6**	**0.324**
rs12054328	t	c	**0.35**	**0.01**	**0.066**	**0.010**	**7.09E-11**	**++++**	**28.8**	**0.239**
rs724304	a	c	**0.37**	**0.01**	**0.065**	**0.010**	**7.11E-11**	**++++**	**28.5**	**0.241**
rs66544515	a	g	**0.63**	**0.01**	**-0.065**	**0.010**	**7.12E-11**	**----**	**24.9**	**0.262**
rs11127900	a	g	**0.37**	**0.01**	**0.065**	**0.010**	**7.13E-11**	**++++**	**16.4**	**0.309**
rs4856585	a	t	**0.63**	**0.01**	**-0.065**	**0.010**	**7.13E-11**	**----**	**20.9**	**0.285**
rs4856273	a	g	**0.63**	**0.01**	**-0.065**	**0.010**	**7.14E-11**	**----**	**20.8**	**0.285**
rs1463208	a	c	**0.63**	**0.01**	**-0.065**	**0.010**	**7.15E-11**	**----**	**20**	**0.290**
rs17517121	a	g	**0.63**	**0.01**	**-0.065**	**0.010**	**7.16E-11**	**----**	**20.7**	**0.286**
rs17457669	t	c	**0.37**	**0.01**	**0.065**	**0.010**	**7.18E-11**	**++++**	**18.7**	**0.297**
rs4856277	a	g	**0.37**	**0.01**	**0.065**	**0.010**	**7.19E-11**	**++++**	**25.3**	**0.260**
rs17459563	a	g	**0.63**	**0.01**	**-0.065**	**0.010**	**7.19E-11**	**----**	**27.3**	**0.248**
rs10511081	c	g	**0.37**	**0.01**	**0.065**	**0.010**	**7.19E-11**	**++++**	**23.4**	**0.271**
rs7640828	a	g	**0.65**	**0.01**	**-0.066**	**0.010**	**7.19E-11**	**----**	**21.9**	**0.279**
rs17457642	t	c	**0.63**	**0.01**	**-0.065**	**0.010**	**7.20E-11**	**----**	**18.7**	**0.297**

rs62250492	t	c	**0.37**	**0.01**	**0.065**	**0.010**	**7.20E-11**	**++++**	**23.4**	**0.271**
rs4603966	t	c	**0.37**	**0.01**	**0.065**	**0.010**	**7.21E-11**	**++++**	**20.5**	**0.287**
rs7620313	t	c	**0.63**	**0.01**	**-0.065**	**0.010**	**7.22E-11**	**----**	**18.7**	**0.297**
rs62253964	t	c	**0.63**	**0.01**	**-0.065**	**0.010**	**7.23E-11**	**----**	**20.6**	**0.286**
rs2029134	t	g	**0.35**	**0.01**	**0.066**	**0.010**	**7.27E-11**	**++++**	**21.9**	**0.279**
rs4441668	a	g	**0.37**	**0.01**	**0.065**	**0.010**	**7.27E-11**	**++++**	**25.2**	**0.261**
rs68028504	a	g	**0.37**	**0.01**	**0.065**	**0.010**	**7.28E-11**	**++++**	**25.1**	**0.261**
rs17519241	t	c	**0.63**	**0.01**	**-0.065**	**0.010**	**7.29E-11**	**----**	**25.3**	**0.260**
rs1530738	a	g	**0.37**	**0.01**	**0.065**	**0.010**	**7.30E-11**	**++++**	**25.3**	**0.260**
rs67277278	a	c	**0.63**	**0.01**	**-0.065**	**0.010**	**7.34E-11**	**----**	**25.6**	**0.258**
rs1449370	t	c	**0.63**	**0.01**	**-0.065**	**0.010**	**7.35E-11**	**----**	**25.5**	**0.259**
rs4543024	t	c	**0.37**	**0.01**	**0.065**	**0.010**	**7.35E-11**	**++++**	**25.5**	**0.259**
rs6549039	a	g	**0.37**	**0.01**	**0.065**	**0.010**	**7.35E-11**	**++++**	**24.7**	**0.263**
rs6549040	t	g	**0.63**	**0.01**	**-0.065**	**0.010**	**7.36E-11**	**----**	**25.1**	**0.261**
rs1900914	a	g	**0.63**	**0.01**	**-0.065**	**0.010**	**7.37E-11**	**----**	**25.3**	**0.260**
rs1449380	t	c	**0.63**	**0.01**	**-0.065**	**0.010**	**7.37E-11**	**----**	**25.3**	**0.260**
rs2122233	a	g	**0.63**	**0.01**	**-0.065**	**0.010**	**7.37E-11**	**----**	**25.4**	**0.259**
rs7653790	t	g	**0.63**	**0.01**	**-0.065**	**0.010**	**7.38E-11**	**----**	**25.1**	**0.261**
rs1449371	t	c	**0.37**	**0.01**	**0.065**	**0.010**	**7.40E-11**	**++++**	**25.1**	**0.261**
rs11127899	a	g	**0.37**	**0.01**	**0.065**	**0.010**	**7.40E-11**	**++++**	**19.4**	**0.293**
rs4502590	t	c	**0.63**	**0.01**	**-0.065**	**0.010**	**7.42E-11**	**----**	**19.7**	**0.291**
rs57276248	a	g	**0.37**	**0.01**	**0.065**	**0.010**	**7.42E-11**	**++++**	**23**	**0.273**
rs6765991	a	g	**0.37**	**0.01**	**0.065**	**0.010**	**7.42E-11**	**++++**	**19.6**	**0.292**
rs1463203	a	t	**0.37**	**0.01**	**0.065**	**0.010**	**7.43E-11**	**++++**	**19.4**	**0.293**
rs66499081	a	g	**0.37**	**0.01**	**0.065**	**0.010**	**7.43E-11**	**++++**	**25.1**	**0.261**
rs1463204	t	c	**0.37**	**0.01**	**0.065**	**0.010**	**7.44E-11**	**++++**	**19.4**	**0.293**
rs6765881	a	g	**0.37**	**0.01**	**0.065**	**0.010**	**7.46E-11**	**++++**	**19.5**	**0.292**
rs17519948	a	c	**0.37**	**0.01**	**0.065**	**0.010**	**7.47E-11**	**++++**	**22.8**	**0.274**
rs62250468	t	c	**0.63**	**0.01**	**-0.065**	**0.010**	**7.49E-11**	**----**	**24.8**	**0.263**
rs1530739	a	t	**0.63**	**0.01**	**-0.065**	**0.010**	**7.50E-11**	**----**	**24.8**	**0.263**

rs59835020	a	g	**0.37**	**0.01**	**0.065**	**0.010**	**7.51E-11**	**++++**	**23.3**	**0.271**
rs7616458	t	c	**0.37**	**0.01**	**0.065**	**0.010**	**7.51E-11**	**++++**	**22.8**	**0.274**
rs6549037	a	g	**0.63**	**0.01**	**-0.065**	**0.010**	**7.54E-11**	**----**	**23.7**	**0.269**
rs1449378	t	c	**0.63**	**0.01**	**-0.065**	**0.010**	**7.54E-11**	**----**	**25.1**	**0.261**
rs1551046	c	g	**0.63**	**0.01**	**-0.065**	**0.010**	**7.54E-11**	**----**	**19.8**	**0.291**
rs1530740	a	g	**0.63**	**0.01**	**-0.065**	**0.010**	**7.55E-11**	**----**	**25.7**	**0.257**
rs1449405	t	c	**0.37**	**0.01**	**0.064**	**0.010**	**7.56E-11**	**++++**	**23.4**	**0.271**
rs2122234	a	g	**0.63**	**0.01**	**-0.065**	**0.010**	**7.58E-11**	**----**	**24.9**	**0.262**
rs2167045	a	g	**0.63**	**0.01**	**-0.065**	**0.010**	**7.59E-11**	**----**	**24.7**	**0.263**
rs2167044	t	c	**0.63**	**0.01**	**-0.065**	**0.010**	**7.61E-11**	**----**	**25**	**0.261**
rs78015688	t	g	**0.37**	**0.00**	**0.065**	**0.010**	**7.62E-11**	**++++**	**20.6**	**0.287**
rs2167043	t	c	**0.37**	**0.01**	**0.065**	**0.010**	**7.62E-11**	**++++**	**25**	**0.261**
rs59073108	a	g	**0.37**	**0.01**	**0.065**	**0.010**	**7.62E-11**	**++++**	**25.1**	**0.261**
rs62253965	a	g	**0.63**	**0.01**	**-0.065**	**0.010**	**7.64E-11**	**----**	**20.4**	**0.288**
rs62252508	a	g	**0.65**	**0.01**	**-0.066**	**0.010**	**7.66E-11**	**----**	**7**	**0.358**
rs12638040	c	g	**0.37**	**0.01**	**0.065**	**0.010**	**7.68E-11**	**++++**	**23.3**	**0.271**
rs17517273	a	c	**0.37**	**0.01**	**0.065**	**0.010**	**7.68E-11**	**++++**	**17.8**	**0.302**
rs6777458	a	g	**0.63**	**0.01**	**-0.065**	**0.010**	**7.68E-11**	**----**	**17.8**	**0.302**
rs62253970	c	g	**0.63**	**0.01**	**-0.064**	**0.010**	**7.68E-11**	**----**	**23.8**	**0.269**
rs1449408	c	g	**0.63**	**0.01**	**-0.064**	**0.010**	**7.72E-11**	**----**	**24**	**0.267**
rs7636401	a	g	**0.56**	**0.00**	**-0.070**	**0.011**	**7.73E-11**	**---?**	**0**	**0.726**
rs1449406	a	c	**0.37**	**0.01**	**0.064**	**0.010**	**7.76E-11**	**++++**	**23.4**	**0.270**
rs17518082	t	c	**0.63**	**0.01**	**-0.064**	**0.010**	**7.77E-11**	**----**	**22.3**	**0.277**
rs6792295	t	c	**0.37**	**0.01**	**0.065**	**0.010**	**7.78E-11**	**++++**	**21.3**	**0.282**
rs12494446	a	g	**0.63**	**0.01**	**-0.065**	**0.010**	**7.79E-11**	**----**	**15.9**	**0.312**
rs4856588	a	g	**0.63**	**0.01**	**-0.065**	**0.010**	**7.80E-11**	**----**	**28.3**	**0.242**
rs1449379	t	c	**0.37**	**0.01**	**0.065**	**0.010**	**7.80E-11**	**++++**	**24.7**	**0.263**
rs2167046	a	g	**0.63**	**0.01**	**-0.065**	**0.010**	**7.80E-11**	**----**	**11.3**	**0.336**
rs6549036	t	g	**0.63**	**0.01**	**-0.064**	**0.010**	**7.81E-11**	**----**	**23.7**	**0.269**
rs17460701	t	c	**0.63**	**0.01**	**-0.065**	**0.010**	**7.87E-11**	**----**	**22.6**	**0.275**

rs62253969	a	g	**0.63**	**0.01**	**-0.064**	**0.010**	**7.87E-11**	**----**	**22.4**	**0.276**
rs17519262	a	g	**0.37**	**0.01**	**0.065**	**0.010**	**7.88E-11**	**++++**	**24.6**	**0.264**
rs62253966	a	g	**0.63**	**0.01**	**-0.064**	**0.010**	**7.89E-11**	**----**	**22.2**	**0.278**
rs4856589	a	g	**0.37**	**0.01**	**0.065**	**0.010**	**7.91E-11**	**++++**	**25.8**	**0.257**
rs4301022	t	g	**0.63**	**0.01**	**-0.065**	**0.010**	**7.92E-11**	**----**	**22.9**	**0.273**
rs7618494	a	g	**0.35**	**0.01**	**0.065**	**0.010**	**7.96E-11**	**++++**	**20.8**	**0.285**
rs6804626	a	g	**0.37**	**0.01**	**0.065**	**0.010**	**7.98E-11**	**++++**	**22.8**	**0.274**
rs7617323	t	g	**0.63**	**0.01**	**-0.065**	**0.010**	**8.03E-11**	**----**	**15.7**	**0.313**
rs7617356	t	c	**0.63**	**0.01**	**-0.065**	**0.010**	**8.18E-11**	**----**	**22.5**	**0.276**
rs17458574	t	g	**0.37**	**0.01**	**0.064**	**0.010**	**8.21E-11**	**++++**	**22.3**	**0.277**
rs62250490	t	c	**0.63**	**0.01**	**-0.065**	**0.010**	**8.25E-11**	**----**	**23**	**0.273**
rs55677942	a	g	**0.63**	**0.01**	**0.064**	**0.010**	**8.26E-11**	**++++**	**74.1**	**0.009**
rs62250489	a	c	**0.63**	**0.01**	**-0.065**	**0.010**	**8.27E-11**	**----**	**23.1**	**0.273**
rs17458609	t	c	**0.63**	**0.01**	**-0.064**	**0.010**	**8.29E-11**	**----**	**23.1**	**0.272**
rs4856593	t	c	**0.37**	**0.01**	**0.065**	**0.010**	**8.29E-11**	**++++**	**24.1**	**0.267**
rs62250472	a	c	**0.63**	**0.01**	**-0.065**	**0.010**	**8.29E-11**	**----**	**24.1**	**0.267**
rs72615727	c	g	**0.63**	**0.01**	**-0.065**	**0.010**	**8.29E-11**	**----**	**30.1**	**0.232**
rs1449383	a	g	**0.37**	**0.01**	**0.065**	**0.010**	**8.30E-11**	**++++**	**23.3**	**0.271**
rs7629091	t	g	**0.37**	**0.01**	**0.065**	**0.010**	**8.31E-11**	**++++**	**23.3**	**0.271**
rs10511078	a	g	**0.65**	**0.01**	**-0.066**	**0.010**	**8.31E-11**	**----**	**4.7**	**0.369**
rs17459906	t	c	**0.37**	**0.01**	**0.064**	**0.010**	**8.32E-11**	**++++**	**23.9**	**0.268**
rs67999244	a	t	**0.63**	**0.01**	**-0.065**	**0.010**	**8.35E-11**	**----**	**23.7**	**0.269**
rs10865610	a	g	**0.37**	**0.01**	**0.065**	**0.010**	**8.41E-11**	**++++**	**23.5**	**0.270**
rs55994690	a	t	**0.61**	**0.01**	**-0.074**	**0.011**	**8.41E-11**	**---?**	**0**	**0.744**
rs17023032	a	c	**0.63**	**0.01**	**-0.065**	**0.010**	**8.41E-11**	**----**	**17.3**	**0.305**
rs1449385	t	c	**0.37**	**0.01**	**0.064**	**0.010**	**8.42E-11**	**++++**	**22.8**	**0.274**
rs61316596	t	c	**0.63**	**0.01**	**-0.065**	**0.010**	**8.46E-11**	**----**	**22.6**	**0.275**
rs1449382	a	g	**0.63**	**0.01**	**-0.065**	**0.010**	**8.46E-11**	**----**	**24.8**	**0.262**
rs1449384	a	t	**0.63**	**0.01**	**-0.064**	**0.010**	**8.46E-11**	**----**	**22.7**	**0.274**
rs4856592	a	g	**0.63**	**0.01**	**-0.065**	**0.010**	**8.48E-11**	**----**	**24.1**	**0.267**

rs59417256	t	g	**0.37**	**0.01**	**0.065**	**0.010**	**8.49E-11**	**++++**	**22.6**	**0.275**
rs6801823	a	t	**0.37**	**0.01**	**0.065**	**0.010**	**8.50E-11**	**++++**	**22.4**	**0.276**
rs6549043	a	t	**0.37**	**0.01**	**0.065**	**0.010**	**8.51E-11**	**++++**	**13.4**	**0.326**
rs6766690	t	c	**0.63**	**0.01**	**-0.065**	**0.010**	**8.53E-11**	**----**	**24**	**0.267**
rs6549042	a	g	**0.37**	**0.01**	**0.065**	**0.010**	**8.56E-11**	**++++**	**6.4**	**0.361**
rs1449381	a	g	**0.37**	**0.01**	**0.064**	**0.010**	**8.57E-11**	**++++**	**24.5**	**0.265**
rs59211082	t	c	**0.37**	**0.01**	**0.065**	**0.010**	**8.59E-11**	**++++**	**22.9**	**0.273**
rs67904150	a	g	**0.37**	**0.01**	**0.065**	**0.010**	**8.62E-11**	**++++**	**22.5**	**0.276**
rs12633657	a	c	**0.65**	**0.01**	**-0.066**	**0.010**	**8.66E-11**	**----**	**3.3**	**0.376**
rs6549044	a	g	**0.37**	**0.01**	**0.065**	**0.010**	**8.71E-11**	**++++**	**14.7**	**0.318**
rs4856596	t	g	**0.63**	**0.01**	**-0.065**	**0.010**	**8.74E-11**	**----**	**22.6**	**0.275**
rs62250501	c	g	**0.37**	**0.01**	**0.065**	**0.010**	**8.81E-11**	**++++**	**7.9**	**0.354**
rs11127898	c	g	**0.35**	**0.01**	**0.066**	**0.010**	**8.81E-11**	**++++**	**7.5**	**0.355**
rs62250500	t	c	**0.63**	**0.01**	**-0.065**	**0.010**	**8.84E-11**	**----**	**8**	**0.353**
rs6549041	c	g	**0.37**	**0.01**	**0.065**	**0.010**	**8.86E-11**	**++++**	**7.8**	**0.354**
rs6762733	a	g	**0.63**	**0.01**	**-0.065**	**0.010**	**8.87E-11**	**----**	**7.8**	**0.354**
rs17458504	a	g	**0.63**	**0.01**	**-0.064**	**0.010**	**8.89E-11**	**----**	**23.7**	**0.269**
rs6549035	t	c	**0.35**	**0.01**	**0.065**	**0.010**	**8.92E-11**	**++++**	**13.2**	**0.326**
rs1375554	a	g	**0.37**	**0.01**	**0.064**	**0.010**	**8.92E-11**	**++++**	**22.5**	**0.276**
rs2326320	t	c	**0.63**	**0.01**	**-0.065**	**0.010**	**8.93E-11**	**----**	**22.6**	**0.275**
rs1375555	t	c	**0.37**	**0.01**	**0.064**	**0.010**	**8.99E-11**	**++++**	**28.9**	**0.239**
rs4856590	a	g	**0.63**	**0.01**	**-0.065**	**0.010**	**9.00E-11**	**----**	**28.1**	**0.244**
rs1900915	c	g	**0.63**	**0.01**	**-0.064**	**0.010**	**9.00E-11**	**----**	**22.5**	**0.276**
rs6762535	a	g	**0.63**	**0.01**	**-0.065**	**0.010**	**9.01E-11**	**----**	**7.8**	**0.354**
rs1449407	a	g	**0.37**	**0.01**	**0.064**	**0.010**	**9.04E-11**	**++++**	**22.9**	**0.274**
rs1375551	t	c	**0.37**	**0.01**	**0.064**	**0.010**	**9.06E-11**	**++++**	**22.4**	**0.277**
rs6788098	a	t	**0.37**	**0.01**	**0.064**	**0.010**	**9.11E-11**	**++++**	**22.2**	**0.278**
rs4856584	a	t	**0.35**	**0.01**	**0.066**	**0.010**	**9.12E-11**	**++++**	**3.1**	**0.377**
rs68001049	a	g	**0.37**	**0.01**	**0.064**	**0.010**	**9.17E-11**	**++++**	**21.1**	**0.283**
rs6783137	t	c	**0.63**	**0.01**	**-0.064**	**0.010**	**9.20E-11**	**----**	**22.7**	**0.275**

rs62250496	t	g	**0.63**	**0.01**	**-0.065**	**0.010**	**9.22E-11**	**----**	**23.2**	**0.272**
rs62250467	t	c	**0.37**	**0.01**	**0.064**	**0.010**	**9.24E-11**	**++++**	**21.4**	**0.282**
rs62250503	a	t	**0.63**	**0.01**	**-0.064**	**0.010**	**9.25E-11**	**----**	**13.3**	**0.326**
rs1375558	a	g	**0.37**	**0.01**	**0.065**	**0.010**	**9.47E-11**	**++++**	**14.8**	**0.318**
rs4508796	a	g	**0.37**	**0.01**	**0.064**	**0.010**	**9.50E-11**	**++++**	**21.2**	**0.283**
rs6794866	a	t	**0.63**	**0.01**	**-0.065**	**0.010**	**9.50E-11**	**----**	**21.5**	**0.281**
rs7617306	t	c	**0.63**	**0.01**	**-0.064**	**0.010**	**9.56E-11**	**----**	**25.8**	**0.257**
rs6783138	c	g	**0.37**	**0.01**	**0.064**	**0.010**	**9.67E-11**	**++++**	**22.4**	**0.277**
rs67955391	c	g	**0.37**	**0.01**	**0.065**	**0.010**	**9.69E-11**	**++++**	**21.6**	**0.281**
rs62253971	a	g	**0.37**	**0.01**	**0.064**	**0.010**	**9.79E-11**	**++++**	**23.5**	**0.270**
rs11127907	a	g	**0.71**	**0.01**	**0.069**	**0.011**	**9.81E-11**	**+-++**	**78**	**0.003**
rs7634761	a	g	**0.63**	**0.01**	**-0.065**	**0.010**	**9.86E-11**	**----**	**21.8**	**0.280**
rs1375557	t	g	**0.63**	**0.01**	**-0.065**	**0.010**	**9.95E-11**	**----**	**14.6**	**0.319**
rs2029131	a	c	**0.37**	**0.01**	**0.064**	**0.010**	**9.99E-11**	**++++**	**23.7**	**0.269**
rs1375565	a	g	**0.37**	**0.01**	**0.065**	**0.010**	**1.01E-10**	**++++**	**21.1**	**0.284**
rs2326319	a	g	**0.65**	**0.01**	**-0.065**	**0.010**	**1.01E-10**	**----**	**16.5**	**0.309**
rs12629607	t	c	**0.34**	**0.01**	**0.066**	**0.010**	**1.03E-10**	**++++**	**6.6**	**0.360**
rs13059866	a	g	**0.89**	**0.01**	**-0.100**	**0.016**	**1.03E-10**	**----**	**0**	**0.726**
rs62253968	t	c	**0.35**	**0.01**	**0.065**	**0.010**	**1.03E-10**	**++++**	**21.1**	**0.284**
rs62253967	a	g	**0.65**	**0.01**	**-0.065**	**0.010**	**1.03E-10**	**----**	**21.1**	**0.284**
rs1449386	t	g	**0.36**	**0.01**	**0.065**	**0.010**	**1.03E-10**	**++++**	**15.6**	**0.314**
rs12492753	a	t	**0.65**	**0.01**	**-0.065**	**0.010**	**1.03E-10**	**----**	**19.9**	**0.290**
rs13068761	t	c	**0.44**	**0.00**	**-0.068**	**0.011**	**1.04E-10**	**---?**	**1.7**	**0.362**
rs4856274	c	g	**0.35**	**0.01**	**0.065**	**0.010**	**1.05E-10**	**++++**	**20.6**	**0.287**
rs6807666	a	c	**0.35**	**0.01**	**0.065**	**0.010**	**1.05E-10**	**++++**	**15**	**0.317**
rs67584463	a	t	**0.65**	**0.01**	**-0.065**	**0.010**	**1.05E-10**	**----**	**19.9**	**0.290**
rs58889493	a	t	**0.63**	**0.01**	**-0.064**	**0.010**	**1.06E-10**	**----**	**21.8**	**0.279**
rs1375550	a	g	**0.37**	**0.01**	**0.064**	**0.010**	**1.09E-10**	**++++**	**37**	**0.190**
rs1375549	t	c	**0.37**	**0.01**	**0.064**	**0.010**	**1.10E-10**	**++++**	**37**	**0.190**
rs1375566	a	g	**0.63**	**0.01**	**-0.064**	**0.010**	**1.10E-10**	**----**	**14.6**	**0.319**

rs6790699	a	g	**0.37**	**0.01**	**0.064**	**0.010**	**1.13E-10**	**++++**	**19.9**	**0.290**
rs11921010	t	c	**0.39**	**0.01**	**0.064**	**0.010**	**1.15E-10**	**++++**	**0**	**0.466**
rs60541362	a	g	**0.35**	**0.01**	**0.065**	**0.010**	**1.16E-10**	**++++**	**20.9**	**0.285**
rs7652808	t	g	**0.34**	**0.01**	**0.065**	**0.010**	**1.21E-10**	**++++**	**22**	**0.279**
rs12639564	a	g	**0.36**	**0.01**	**0.064**	**0.010**	**1.21E-10**	**++++**	**17**	**0.306**
rs7652683	t	c	**0.34**	**0.01**	**0.065**	**0.010**	**1.22E-10**	**++++**	**22.2**	**0.277**
rs17023019	a	g	**0.35**	**0.01**	**0.065**	**0.010**	**1.25E-10**	**++++**	**21**	**0.284**
rs2326318	c	g	**0.37**	**0.01**	**0.064**	**0.010**	**1.35E-10**	**++++**	**16.3**	**0.310**
rs56031423	t	c	**0.65**	**0.01**	**-0.065**	**0.010**	**1.36E-10**	**----**	**19.7**	**0.292**
rs2044725	t	c	**0.66**	**0.01**	**-0.065**	**0.010**	**1.42E-10**	**----**	**31.9**	**0.221**
rs6782190	a	g	**0.65**	**0.01**	**-0.065**	**0.010**	**1.45E-10**	**----**	**24.3**	**0.266**
rs62250537	c	g	**0.35**	**0.01**	**0.065**	**0.010**	**1.59E-10**	**++++**	**24.2**	**0.266**
rs1551042	a	c	**0.35**	**0.01**	**0.064**	**0.010**	**1.65E-10**	**++++**	**7.8**	**0.354**
rs12495144	a	g	**0.44**	**0.00**	**-0.067**	**0.011**	**1.69E-10**	**---?**	**19.7**	**0.288**
rs62261676	a	g	**0.79**	**0.00**	**0.075**	**0.012**	**1.73E-10**	**+-++**	**33.7**	**0.210**
rs112069021	t	c	**0.37**	**0.01**	**0.064**	**0.010**	**1.81E-10**	**++++**	**23.7**	**0.269**
rs2875908	t	c	**0.37**	**0.01**	**0.063**	**0.010**	**1.90E-10**	**++++**	**17.5**	**0.303**
rs17025272	t	c	**0.54**	**0.00**	**0.067**	**0.011**	**1.95E-10**	**+++?**	**34.1**	**0.219**
rs73137879	a	c	**0.63**	**0.01**	**-0.064**	**0.010**	**2.06E-10**	**----**	**23.7**	**0.269**
rs1972994	a	t	**0.35**	**0.01**	**0.064**	**0.010**	**2.83E-10**	**++++**	**13.3**	**0.326**
rs145178380	t	g	**0.44**	**0.00**	**-0.066**	**0.011**	**3.03E-10**	**---?**	**9.6**	**0.331**
rs62261692	t	c	**0.22**	**0.01**	**-0.072**	**0.011**	**3.22E-10**	**-+--**	**36.7**	**0.192**
rs7651114	a	g	**0.30**	**0.01**	**0.067**	**0.011**	**3.32E-10**	**++++**	**0**	**0.796**
rs9829032	a	g	**0.59**	**0.01**	**-0.062**	**0.010**	**3.53E-10**	**----**	**53.7**	**0.091**
rs4266190	t	g	**0.44**	**0.00**	**-0.066**	**0.011**	**4.10E-10**	**---?**	**14.8**	**0.309**
rs62261691	t	g	**0.78**	**0.01**	**0.071**	**0.011**	**4.27E-10**	**+-++**	**36.1**	**0.196**
rs62263332	t	c	**0.44**	**0.00**	**-0.065**	**0.011**	**5.44E-10**	**---?**	**18**	**0.296**
rs62261689	c	g	**0.78**	**0.01**	**0.071**	**0.011**	**5.78E-10**	**+-++**	**38.2**	**0.183**
rs4575895	a	g	**0.44**	**0.00**	**-0.065**	**0.011**	**5.81E-10**	**---?**	**17.2**	**0.299**
rs12632674	a	g	**0.63**	**0.01**	**0.062**	**0.010**	**6.18E-10**	**++++**	**72.9**	**0.011**

rs4856591	t	g	**0.38**	**0.00**	**0.064**	**0.010**	**6.70E-10**	**++++**	**12.7**	**0.329**
rs7643534	a	g	**0.22**	**0.01**	**-0.071**	**0.011**	**6.86E-10**	**-+--**	**39.4**	**0.176**
rs34974070	a	g	**0.44**	**0.00**	**-0.065**	**0.011**	**6.87E-10**	**---?**	**18.2**	**0.295**
rs12493881	a	g	**0.44**	**0.00**	**-0.065**	**0.011**	**8.30E-10**	**---?**	**16.3**	**0.303**
rs11926266	c	g	**0.78**	**0.01**	**0.070**	**0.012**	**8.31E-10**	**+-++**	**38.9**	**0.178**
rs6780346	t	c	**0.63**	**0.01**	**-0.061**	**0.010**	**9.57E-10**	**----**	**17.5**	**0.304**
rs35885688	t	c	**0.56**	**0.00**	**0.064**	**0.011**	**9.66E-10**	**+++?**	**16**	**0.304**
rs11922775	c	g	**0.78**	**0.00**	**0.070**	**0.011**	**1.02E-09**	**+-++**	**43.4**	**0.151**
rs2325035	a	c	**0.26**	**0.01**	**-0.067**	**0.011**	**1.12E-09**	**-+--**	**15.5**	**0.314**
rs59778458	c	g	**0.30**	**0.00**	**0.069**	**0.011**	**1.39E-09**	**+++?**	**0**	**0.810**
rs12494510	t	c	**0.70**	**0.00**	**-0.068**	**0.011**	**1.59E-09**	**---?**	**0**	**0.794**
rs6805281	a	t	**0.70**	**0.00**	**-0.068**	**0.011**	**1.62E-09**	**---?**	**0**	**0.828**
rs13070166	a	t	**0.23**	**0.00**	**0.075**	**0.012**	**1.68E-09**	**+++?**	**0**	**0.721**
rs17023296	t	c	**0.74**	**0.01**	**0.066**	**0.011**	**1.76E-09**	**+-++**	**24.6**	**0.264**
rs3924693	t	c	**0.78**	**0.00**	**0.069**	**0.012**	**1.98E-09**	**+-++**	**42.3**	**0.158**
rs3158	t	c	**0.26**	**0.01**	**-0.065**	**0.011**	**2.13E-09**	**-+--**	**55.8**	**0.079**
rs67414003	a	c	**0.22**	**0.01**	**-0.069**	**0.012**	**2.22E-09**	**-+--**	**42.8**	**0.155**
rs34799934	a	c	**0.78**	**0.01**	**0.069**	**0.012**	**2.23E-09**	**+-++**	**42.2**	**0.159**
rs11928212	t	c	**0.22**	**0.01**	**-0.068**	**0.012**	**2.34E-09**	**-+--**	**42.8**	**0.155**
rs62263330	a	t	**0.36**	**0.02**	**-0.071**	**0.012**	**2.59E-09**	**-+-?**	**0**	**0.388**
rs12634260	c	g	**0.46**	**0.00**	**-0.062**	**0.011**	**3.19E-09**	**---?**	**33.8**	**0.221**
rs13095644	a	g	**0.53**	**0.01**	**0.057**	**0.010**	**3.47E-09**	**+++-**	**67.3**	**0.027**
rs7628129	t	c	**0.47**	**0.01**	**-0.057**	**0.010**	**3.62E-09**	**---+**	**67.7**	**0.026**
rs9863150	t	c	**0.56**	**0.06**	**0.057**	**0.010**	**4.00E-09**	**++++**	**60.9**	**0.053**
rs7630382	t	c	**0.53**	**0.01**	**0.057**	**0.010**	**4.43E-09**	**+++-**	**66.3**	**0.031**
rs13074075	a	c	**0.48**	**0.02**	**-0.057**	**0.010**	**4.60E-09**	**---+**	**64.4**	**0.038**
rs9835904	a	g	**0.47**	**0.01**	**-0.056**	**0.010**	**5.34E-09**	**---+**	**66.8**	**0.029**
rs9878472	t	c	**0.47**	**0.01**	**-0.057**	**0.010**	**5.39E-09**	**---+**	**66.9**	**0.029**
rs55829275	t	c	**0.73**	**0.01**	**0.062**	**0.011**	**6.40E-09**	**+-++**	**29.8**	**0.234**
rs62261700	c	g	**0.73**	**0.01**	**0.062**	**0.011**	**6.55E-09**	**+-++**	**27.6**	**0.246**

rs12497036	t	c	**0.27**	**0.01**	**-0.062**	**0.011**	**6.58E-09**	**-+--**	**31.7**	**0.222**
rs6549075	a	g	**0.31**	**0.00**	**0.066**	**0.011**	**6.63E-09**	**+++?**	**0**	**0.819**
rs3159	t	g	**0.12**	**0.01**	**0.086**	**0.015**	**7.06E-09**	**++++**	**0**	**0.940**
rs1448610	t	c	**0.73**	**0.01**	**0.062**	**0.011**	**7.18E-09**	**+-++**	**31.2**	**0.225**
rs4507269	a	g	**0.22**	**0.01**	**-0.067**	**0.012**	**7.46E-09**	**-+--**	**39.8**	**0.173**
rs4516626	t	c	**0.53**	**0.02**	**0.055**	**0.010**	**7.51E-09**	**+++-**	**67.5**	**0.026**
rs56101762	t	c	**0.73**	**0.01**	**0.062**	**0.011**	**7.65E-09**	**+-++**	**31.6**	**0.223**
rs9838091	t	c	**0.53**	**0.02**	**0.055**	**0.010**	**8.04E-09**	**+++-**	**67.4**	**0.027**
rs11708023	t	c	**0.27**	**0.01**	**-0.062**	**0.011**	**9.23E-09**	**-+--**	**32.7**	**0.216**
rs13099482	a	t	**0.39**	**0.01**	**-0.056**	**0.010**	**9.81E-09**	**-+--**	**70.5**	**0.017**
rs1003984	a	t	**0.86**	**0.01**	**0.080**	**0.014**	**9.84E-09**	**++++**	**0**	**0.882**
rs9846802	t	c	**0.47**	**0.02**	**-0.055**	**0.010**	**1.04E-08**	**---+**	**67**	**0.028**
rs7627287	a	g	**0.61**	**0.01**	**0.056**	**0.010**	**1.07E-08**	**+-++**	**68.8**	**0.022**
rs6808586	t	c	**0.53**	**0.02**	**0.055**	**0.010**	**1.07E-08**	**+++-**	**66**	**0.032**
rs9874740	a	g	**0.24**	**0.01**	**0.065**	**0.011**	**1.08E-08**	**++++**	**0**	**0.457**
rs9841144	a	t	**0.78**	**0.01**	**-0.067**	**0.012**	**1.09E-08**	**----**	**12.8**	**0.329**
rs7620825	t	g	**0.39**	**0.01**	**-0.056**	**0.010**	**1.09E-08**	**-+--**	**69.8**	**0.019**
rs13071488	a	g	**0.39**	**0.01**	**-0.056**	**0.010**	**1.10E-08**	**-+--**	**70.2**	**0.018**
rs6808159	t	c	**0.24**	**0.01**	**0.065**	**0.011**	**1.11E-08**	**++++**	**0**	**0.460**
rs9821041	t	c	**0.24**	**0.01**	**0.065**	**0.011**	**1.12E-08**	**++++**	**0**	**0.457**
rs9849399	a	c	**0.78**	**0.01**	**-0.067**	**0.012**	**1.14E-08**	**----**	**13.8**	**0.324**
rs1003986	t	g	**0.14**	**0.01**	**-0.080**	**0.014**	**1.14E-08**	**----**	**0**	**0.893**
rs1368739	t	c	**0.76**	**0.01**	**-0.065**	**0.011**	**1.15E-08**	**----**	**0**	**0.455**
rs35498642	t	c	**0.39**	**0.01**	**-0.056**	**0.010**	**1.15E-08**	**-+--**	**67.1**	**0.028**
rs9309982	t	c	**0.76**	**0.01**	**-0.065**	**0.011**	**1.16E-08**	**----**	**0**	**0.455**
rs9309980	a	g	**0.76**	**0.01**	**-0.065**	**0.011**	**1.18E-08**	**----**	**0**	**0.457**
rs9309981	a	g	**0.76**	**0.01**	**-0.065**	**0.011**	**1.18E-08**	**----**	**0**	**0.457**
rs13081230	a	g	**0.24**	**0.01**	**0.065**	**0.011**	**1.20E-08**	**++++**	**0**	**0.462**
rs6809805	a	c	**0.22**	**0.01**	**0.067**	**0.012**	**1.24E-08**	**++++**	**17.2**	**0.305**
rs17879296	a	c	**0.39**	**0.01**	**-0.056**	**0.010**	**1.24E-08**	**-+--**	**68.6**	**0.023**

rs12633762	t	c	**0.76**	**0.01**	**-0.065**	**0.011**	**1.24E-08**	**----**	**0**	**0.456**		
rs9835441	a	g	**0.24**	**0.01**	**0.065**	**0.011**	**1.25E-08**	**++++**	**0**	**0.461**		
rs13084531	c	g	**0.78**	**0.01**	**-0.067**	**0.012**	**1.25E-08**	**----**	**10.9**	**0.339**		
rs11127890	t	c	**0.24**	**0.01**	**0.065**	**0.011**	**1.27E-08**	**++++**	**0**	**0.462**		
rs11127891	t	c	**0.76**	**0.01**	**-0.065**	**0.011**	**1.27E-08**	**----**	**0**	**0.458**		
rs1003985	a	g	**0.14**	**0.01**	**-0.079**	**0.014**	**1.28E-08**	**----**	**0**	**0.904**		
rs10433498	a	g	**0.24**	**0.01**	**0.064**	**0.011**	**1.30E-08**	**++++**	**0**	**0.453**		
rs11713902	t	c	**0.22**	**0.01**	**0.066**	**0.012**	**1.35E-08**	**++++**	**25.6**	**0.258**		
rs9880919	a	g	**0.24**	**0.01**	**0.064**	**0.011**	**1.37E-08**	**++++**	**0**	**0.467**		
rs9838811	a	c	**0.24**	**0.01**	**0.065**	**0.011**	**1.38E-08**	**++++**	**0**	**0.459**		
rs9813512	t	g	**0.76**	**0.01**	**-0.065**	**0.011**	**1.38E-08**	**----**	**0**	**0.467**		
rs1025531	a	t	**0.24**	**0.01**	**0.065**	**0.011**	**1.38E-08**	**++++**	**0**	**0.466**		
rs13093396	a	g	**0.54**	**0.00**	**0.059**	**0.011**	**1.40E-08**	**+++?**	**22.9**	**0.273**		
rs9865745	a	t	**0.24**	**0.01**	**0.064**	**0.011**	**1.40E-08**	**++++**	**0**	**0.469**		
rs744580	a	t	**0.24**	**0.01**	**0.065**	**0.011**	**1.41E-08**	**++++**	**0**	**0.481**		
rs1025533	t	c	**0.24**	**0.01**	**0.064**	**0.011**	**1.43E-08**	**++++**	**0**	**0.469**		
rs9844512	a	c	**0.22**	**0.01**	**0.066**	**0.012**	**1.43E-08**	**++++**	**15.2**	**0.316**		
rs13092627	t	c	**0.24**	**0.01**	**0.064**	**0.011**	**1.44E-08**	**++++**	**0**	**0.468**		
rs9868427	t	c	**0.24**	**0.01**	**0.065**	**0.011**	**1.45E-08**	**++++**	**0**	**0.432**		
rs12487728	t	c	**0.14**	**0.01**	**-0.079**	**0.014**	**1.48E-08**	**----**	**0**	**0.906**		
rs9865191	t	c	**0.24**	**0.01**	**0.065**	**0.011**	**1.49E-08**	**++++**	**0**	**0.474**		
rs17023388	a	g	**0.76**	**0.01**	**0.063**	**0.011**	**1.51E-08**	**+-++**	**60.5**	**0.055**		
rs1030721	t	c	**0.76**	**0.01**	**-0.064**	**0.011**	**1.52E-08**	**----**	**0**	**0.473**		
rs12488483	a	g	**0.14**	**0.01**	**-0.079**	**0.014**	**1.53E-08**	**----**	**0**	**0.907**		
rs6549024	t	c	**0.76**	**0.01**	**-0.064**	**0.011**	**1.57E-08**	**----**	**0**	**0.474**		
rs4856604	a	g	**0.75**	**0.01**	**0.063**	**0.011**	**1.60E-08**	**+-++**	**46.1**	**0.135**		
rs12494658	t	c	**0.76**	**0.01**	**0.063**	**0.011**	**1.64E-08**	**+-++**	**59.6**	**0.060**		
rs11127918	t	c	**0.54**	**0.00**	**0.059**	**0.011**	**1.71E-08**	**+++?**	**20.1**	**0.286**		
rs2117151	a	c	**0.24**	**0.01**	**0.064**	**0.011**	**1.73E-08**	**++++**	**0**	**0.478**		
rs9879025	t	c	**0.24**	**0.00**	**0.064**	**0.011**	**1.76E-08**	**++++**	**0**	**0.490**		

rs12493461	a	g	**0.76**	**0.01**	**-0.064**	**0.011**	**1.78E-08**	**----**	**0**	**0.480**
rs1368743	a	g	**0.76**	**0.01**	**-0.064**	**0.011**	**1.78E-08**	**----**	**0**	**0.488**
rs6807456	c	g	**0.76**	**0.01**	**-0.064**	**0.011**	**1.79E-08**	**----**	**0**	**0.478**
rs9309977	t	c	**0.24**	**0.01**	**0.064**	**0.011**	**1.87E-08**	**++++**	**0**	**0.491**
rs9819830	t	c	**0.76**	**0.01**	**-0.064**	**0.011**	**1.87E-08**	**----**	**0**	**0.482**
rs13085424	t	c	**0.74**	**0.00**	**-0.066**	**0.012**	**1.88E-08**	**-+-?**	**16.2**	**0.303**
rs12491181	t	c	**0.45**	**0.00**	**-0.059**	**0.011**	**1.88E-08**	**---?**	**5.5**	**0.347**
rs2326313	a	t	**0.24**	**0.01**	**0.064**	**0.011**	**1.90E-08**	**++++**	**0**	**0.493**
rs1821349	a	g	**0.76**	**0.00**	**-0.064**	**0.011**	**1.92E-08**	**----**	**0**	**0.476**
rs13085170	a	c	**0.24**	**0.01**	**0.064**	**0.011**	**1.95E-08**	**++++**	**0**	**0.482**
rs9654015	a	c	**0.74**	**0.00**	**-0.066**	**0.012**	**1.96E-08**	**-+-?**	**17.2**	**0.299**
rs9841829	t	g	**0.78**	**0.01**	**-0.065**	**0.012**	**1.96E-08**	**----**	**18.5**	**0.298**
rs6770276	a	c	**0.74**	**0.00**	**-0.066**	**0.012**	**1.97E-08**	**-+-?**	**17.5**	**0.297**
rs9861497	a	t	**0.74**	**0.00**	**-0.066**	**0.012**	**2.02E-08**	**-+-?**	**25.2**	**0.263**
rs11716886	t	c	**0.26**	**0.00**	**0.066**	**0.012**	**2.03E-08**	**+-+?**	**16.3**	**0.303**
rs9822731	t	c	**0.78**	**0.00**	**-0.070**	**0.013**	**2.05E-08**	**---?**	**0**	**0.767**
rs1433711	t	c	**0.24**	**0.01**	**0.064**	**0.011**	**2.06E-08**	**++++**	**0**	**0.494**
rs4279114	c	g	**0.76**	**0.01**	**-0.064**	**0.011**	**2.11E-08**	**----**	**0**	**0.485**
rs9814919	c	g	**0.24**	**0.01**	**0.064**	**0.011**	**2.14E-08**	**++++**	**0**	**0.491**
rs9851444	t	c	**0.78**	**0.01**	**-0.065**	**0.012**	**2.14E-08**	**----**	**18.8**	**0.297**
rs9866761	t	c	**0.78**	**0.00**	**-0.065**	**0.012**	**2.14E-08**	**----**	**44.3**	**0.146**
rs9883729	t	c	**0.26**	**0.00**	**0.066**	**0.012**	**2.15E-08**	**+-+?**	**16.3**	**0.303**
rs9873400	a	g	**0.22**	**0.01**	**0.065**	**0.012**	**2.19E-08**	**++++**	**23**	**0.273**
rs6803322	a	c	**0.32**	**0.00**	**0.063**	**0.011**	**2.21E-08**	**+++?**	**0**	**0.707**
rs13100969	a	g	**0.55**	**0.00**	**0.059**	**0.011**	**2.23E-08**	**+++?**	**12.3**	**0.320**
rs6772991	a	g	**0.76**	**0.01**	**-0.063**	**0.011**	**2.30E-08**	**----**	**2.1**	**0.382**
rs11920096	a	c	**0.12**	**0.01**	**-0.082**	**0.015**	**2.34E-08**	**----**	**14.8**	**0.318**
rs7650834	a	c	**0.24**	**0.01**	**0.063**	**0.011**	**2.37E-08**	**++++**	**0**	**0.491**
rs7628948	t	c	**0.22**	**0.01**	**0.065**	**0.012**	**2.38E-08**	**++++**	**25.5**	**0.259**
rs9866089	t	c	**0.76**	**0.00**	**-0.067**	**0.012**	**2.41E-08**	**---?**	**0**	**0.904**

rs2087011	t	g	**0.36**	**0.01**	**-0.057**	**0.010**	**2.41E-08**	**-+--**	**3.9**	**0.373**
rs4261888	t	g	**0.74**	**0.00**	**-0.066**	**0.012**	**2.42E-08**	**-+-?**	**21.5**	**0.280**
rs9880953	a	c	**0.26**	**0.00**	**0.066**	**0.012**	**2.43E-08**	**+-+?**	**20**	**0.287**
rs9863620	t	c	**0.76**	**0.01**	**-0.063**	**0.011**	**2.43E-08**	**----**	**0**	**0.498**
rs12714629	t	c	**0.78**	**0.01**	**-0.065**	**0.012**	**2.43E-08**	**----**	**26.7**	**0.252**
rs11712915	a	c	**0.78**	**0.01**	**-0.065**	**0.012**	**2.44E-08**	**----**	**25.8**	**0.257**
rs2196098	a	g	**0.78**	**0.01**	**-0.065**	**0.012**	**2.45E-08**	**----**	**4.5**	**0.371**
rs9833312	a	t	**0.26**	**0.00**	**0.066**	**0.012**	**2.46E-08**	**+-+?**	**18.6**	**0.293**
rs1449404	c	g	**0.78**	**0.01**	**-0.065**	**0.012**	**2.46E-08**	**----**	**25.4**	**0.259**
rs62261744	a	g	**0.61**	**0.01**	**0.055**	**0.010**	**2.48E-08**	**+-++**	**70.1**	**0.018**
rs13092437	a	t	**0.78**	**0.01**	**-0.065**	**0.012**	**2.48E-08**	**----**	**25.6**	**0.258**
rs12494242	t	c	**0.68**	**0.00**	**-0.062**	**0.011**	**2.49E-08**	**---?**	**0**	**0.577**
rs9827900	c	g	**0.26**	**0.00**	**0.066**	**0.012**	**2.55E-08**	**+-+?**	**19.1**	**0.291**
rs3887138	t	g	**0.22**	**0.00**	**0.065**	**0.012**	**2.57E-08**	**++++**	**42.1**	**0.159**
rs9827763	a	g	**0.26**	**0.00**	**0.066**	**0.012**	**2.57E-08**	**+-+?**	**19.7**	**0.288**
rs11708377	a	t	**0.39**	**0.01**	**-0.055**	**0.010**	**2.58E-08**	**-+--**	**69.7**	**0.019**
rs13068434	a	g	**0.78**	**0.01**	**-0.065**	**0.012**	**2.59E-08**	**----**	**4.4**	**0.371**
rs9833314	a	g	**0.24**	**0.01**	**0.063**	**0.011**	**2.59E-08**	**++++**	**0**	**0.494**
rs13076830	t	c	**0.22**	**0.00**	**0.069**	**0.012**	**2.64E-08**	**+++?**	**0**	**0.791**
rs9866322	t	c	**0.78**	**0.00**	**-0.065**	**0.012**	**2.66E-08**	**----**	**43.4**	**0.151**
rs71626887	t	g	**0.24**	**0.01**	**0.063**	**0.011**	**2.67E-08**	**++++**	**0**	**0.481**
rs10154865	t	c	**0.22**	**0.00**	**0.069**	**0.012**	**2.68E-08**	**+++?**	**0**	**0.789**
rs1448609	t	g	**0.25**	**0.01**	**-0.062**	**0.011**	**2.68E-08**	**-+--**	**48.7**	**0.119**
rs1449399	t	c	**0.78**	**0.01**	**0.065**	**0.012**	**2.68E-08**	**+-++**	**44.7**	**0.143**
rs13079014	t	g	**0.39**	**0.01**	**-0.055**	**0.010**	**2.69E-08**	**-+--**	**69.9**	**0.019**
rs9827318	t	g	**0.26**	**0.00**	**0.066**	**0.012**	**2.80E-08**	**+-+?**	**19.5**	**0.289**
rs12631402	a	g	**0.55**	**0.00**	**0.058**	**0.011**	**2.80E-08**	**+++?**	**13.2**	**0.316**
rs9828679	t	c	**0.78**	**0.01**	**-0.065**	**0.012**	**2.83E-08**	**----**	**3.9**	**0.373**
rs9846211	a	c	**0.22**	**0.00**	**0.069**	**0.012**	**2.84E-08**	**+++?**	**0**	**0.792**
rs13097857	a	g	**0.26**	**0.00**	**0.066**	**0.012**	**2.84E-08**	**+-+?**	**19.5**	**0.289**

rs9820228	t	c	**0.22**	**0.01**	**0.065**	**0.012**	**2.89E-08**	**++++**	**3.9**	**0.373**
rs9847516	t	g	**0.74**	**0.00**	**-0.066**	**0.012**	**2.92E-08**	**-+-?**	**22.8**	**0.274**
rs9309989	t	c	**0.89**	**0.00**	**0.085**	**0.015**	**2.92E-08**	**++++**	**38.7**	**0.180**
rs6801271	a	g	**0.64**	**0.01**	**0.057**	**0.010**	**2.93E-08**	**+-++**	**5.4**	**0.366**
rs9810724	a	g	**0.78**	**0.01**	**-0.064**	**0.012**	**2.97E-08**	**----**	**32.1**	**0.220**
rs10511071	t	c	**0.89**	**0.00**	**0.085**	**0.015**	**2.97E-08**	**++++**	**38.6**	**0.180**
rs11716233	t	c	**0.22**	**0.01**	**0.065**	**0.012**	**2.98E-08**	**++++**	**3.9**	**0.373**
rs9968137	t	g	**0.86**	**0.01**	**0.077**	**0.014**	**3.01E-08**	**++++**	**0**	**0.866**
rs6810250	t	g	**0.26**	**0.00**	**0.066**	**0.012**	**3.02E-08**	**+-+?**	**12**	**0.321**
rs13082397	a	c	**0.74**	**0.00**	**-0.065**	**0.012**	**3.03E-08**	**-+-?**	**39.7**	**0.191**
rs6764381	c	g	**0.74**	**0.00**	**-0.066**	**0.012**	**3.05E-08**	**-+-?**	**11.1**	**0.325**
rs34184235	t	c	**0.44**	**0.00**	**-0.058**	**0.011**	**3.07E-08**	**---?**	**26.7**	**0.255**
rs13077660	a	c	**0.78**	**0.01**	**-0.064**	**0.012**	**3.10E-08**	**----**	**33**	**0.214**
rs13084351	t	g	**0.74**	**0.00**	**-0.066**	**0.012**	**3.12E-08**	**-+-?**	**15.4**	**0.306**
rs35344466	a	c	**0.78**	**0.00**	**-0.064**	**0.012**	**3.28E-08**	**----**	**37.2**	**0.189**
rs72914842	c	g	**0.89**	**0.00**	**0.085**	**0.015**	**3.30E-08**	**++++**	**37.4**	**0.188**
rs9826458	c	g	**0.78**	**0.00**	**-0.064**	**0.012**	**3.31E-08**	**----**	**38.6**	**0.181**
rs13085795	a	g	**0.74**	**0.00**	**-0.065**	**0.012**	**3.33E-08**	**-+-?**	**21.2**	**0.281**
rs1901985	t	c	**0.74**	**0.00**	**-0.065**	**0.012**	**3.34E-08**	**-+-?**	**22**	**0.277**
rs6549007	a	g	**0.77**	**0.00**	**0.069**	**0.012**	**3.34E-08**	**+-+?**	**0**	**0.678**
rs17023276	t	c	**0.89**	**0.00**	**0.085**	**0.015**	**3.35E-08**	**++++**	**38.5**	**0.181**
rs12489145	t	c	**0.40**	**0.01**	**-0.054**	**0.010**	**3.36E-08**	**-+--**	**64.2**	**0.039**
rs9867790	a	g	**0.89**	**0.00**	**0.085**	**0.015**	**3.37E-08**	**++++**	**37.1**	**0.190**
rs9309988	a	g	**0.89**	**0.00**	**0.085**	**0.015**	**3.38E-08**	**++++**	**38.8**	**0.179**
rs13089401	c	g	**0.26**	**0.00**	**0.065**	**0.012**	**3.41E-08**	**+-+?**	**32.3**	**0.228**
rs13353478	t	c	**0.22**	**0.00**	**0.064**	**0.012**	**3.47E-08**	**++++**	**34.9**	**0.203**
rs6791236	t	c	**0.78**	**0.01**	**-0.065**	**0.012**	**3.51E-08**	**----**	**3.5**	**0.375**
rs17023258	a	g	**0.11**	**0.00**	**-0.085**	**0.015**	**3.54E-08**	**----**	**39.3**	**0.176**
rs10511072	a	g	**0.89**	**0.00**	**0.085**	**0.015**	**3.54E-08**	**++++**	**37.1**	**0.189**
rs9860792	a	t	**0.39**	**0.01**	**-0.054**	**0.010**	**3.56E-08**	**-+--**	**65.7**	**0.033**

rs12714613	a	g	**0.26**	**0.00**	**0.065**	**0.012**	**3.56E-08**	**+-+?**	**16.7**	**0.301**
rs13068788	a	g	**0.74**	**0.00**	**-0.065**	**0.012**	**3.66E-08**	**-+-?**	**26.8**	**0.255**
rs112374913	a	g	**0.40**	**0.02**	**-0.060**	**0.011**	**3.75E-08**	**---?**	**0**	**0.848**
rs77823788	a	g	**0.11**	**0.00**	**-0.085**	**0.015**	**3.76E-08**	**----**	**39.7**	**0.174**
rs17023290	t	c	**0.89**	**0.00**	**0.085**	**0.015**	**3.80E-08**	**++++**	**29.2**	**0.237**
rs1314615	a	g	**0.55**	**0.00**	**0.058**	**0.011**	**3.84E-08**	**+++?**	**0**	**0.492**
rs9872327	a	t	**0.89**	**0.00**	**0.085**	**0.015**	**3.84E-08**	**++++**	**29**	**0.238**
rs9844801	t	c	**0.89**	**0.00**	**0.085**	**0.015**	**3.89E-08**	**++++**	**37.5**	**0.187**
rs13085427	t	c	**0.39**	**0.01**	**-0.054**	**0.010**	**3.89E-08**	**-+--**	**69.6**	**0.020**
rs13095729	t	c	**0.74**	**0.00**	**-0.065**	**0.012**	**3.93E-08**	**-+-?**	**15.7**	**0.306**
rs9835478	c	g	**0.11**	**0.00**	**-0.085**	**0.015**	**3.96E-08**	**----**	**37.5**	**0.187**
rs9854869	a	c	**0.22**	**0.01**	**0.065**	**0.012**	**4.01E-08**	**++++**	**10.3**	**0.341**
rs7648172	a	g	**0.74**	**0.00**	**-0.065**	**0.012**	**4.04E-08**	**-+-?**	**30.4**	**0.238**
rs9882516	c	g	**0.74**	**0.00**	**-0.065**	**0.012**	**4.05E-08**	**-+-?**	**28**	**0.249**
rs6777505	t	c	**0.74**	**0.00**	**-0.065**	**0.012**	**4.07E-08**	**-+-?**	**21.5**	**0.280**
rs58783194	a	g	**0.45**	**0.01**	**-0.058**	**0.011**	**4.15E-08**	**---?**	**0**	**0.474**
rs2196096	t	c	**0.78**	**0.01**	**-0.064**	**0.012**	**4.16E-08**	**----**	**2.1**	**0.382**
rs1375552	t	g	**0.22**	**0.01**	**0.064**	**0.012**	**4.17E-08**	**+-++**	**45.3**	**0.139**
rs7617565	t	c	**0.26**	**0.00**	**0.065**	**0.012**	**4.21E-08**	**+-+?**	**7.7**	**0.338**
rs71316818	t	g	**0.61**	**0.01**	**0.054**	**0.010**	**4.24E-08**	**+-++**	**65.6**	**0.033**
rs1472302	t	c	**0.47**	**0.02**	**0.054**	**0.010**	**4.24E-08**	**+++-**	**54.8**	**0.084**
rs6764277	t	c	**0.26**	**0.00**	**0.065**	**0.012**	**4.27E-08**	**+-+?**	**15.8**	**0.305**
rs13071144	t	c	**0.74**	**0.00**	**-0.065**	**0.012**	**4.30E-08**	**-+-?**	**30.2**	**0.239**
rs2196097	t	c	**0.22**	**0.01**	**0.064**	**0.012**	**4.31E-08**	**++++**	**1.7**	**0.384**
rs11127912	a	t	**0.61**	**0.01**	**0.054**	**0.010**	**4.33E-08**	**+-++**	**66**	**0.032**
rs34803122	t	c	**0.61**	**0.01**	**0.054**	**0.010**	**4.35E-08**	**+-++**	**65.2**	**0.035**
rs2163971	t	c	**0.44**	**0.02**	**0.054**	**0.010**	**4.36E-08**	**++++**	**0**	**0.797**
rs12633520	a	g	**0.39**	**0.01**	**-0.054**	**0.010**	**4.37E-08**	**-+--**	**65.8**	**0.032**
rs4464472	t	c	**0.61**	**0.01**	**0.054**	**0.010**	**4.38E-08**	**+-++**	**66.7**	**0.029**
rs11917003	t	c	**0.78**	**0.01**	**0.064**	**0.012**	**4.40E-08**	**+-++**	**44**	**0.148**

rs13070794	a	c	**0.26**	**0.00**	**0.065**	**0.012**	**4.42E-08**	**+-+?**	**13.8**	**0.314**						
rs7635835	a	g	**0.26**	**0.00**	**0.065**	**0.012**	**4.43E-08**	**+-+?**	**30**	**0.240**						
rs13077814	a	t	**0.26**	**0.00**	**0.065**	**0.012**	**4.45E-08**	**+-+?**	**30**	**0.240**						
rs7653685	t	c	**0.89**	**0.00**	**0.084**	**0.015**	**4.53E-08**	**++++**	**37**	**0.190**						
rs12633350	a	g	**0.14**	**0.01**	**-0.075**	**0.014**	**4.53E-08**	**----**	**59.1**	**0.062**						
rs17026843	t	c	**0.44**	**0.00**	**0.058**	**0.011**	**4.62E-08**	**+++?**	**0**	**0.888**						
rs13098134	c	g	**0.26**	**0.00**	**0.065**	**0.012**	**4.65E-08**	**+-+?**	**30**	**0.240**						
rs13082138	t	g	**0.39**	**0.00**	**-0.054**	**0.010**	**4.70E-08**	**-+--**	**66**	**0.032**						
rs11707605	t	c	**0.74**	**0.00**	**-0.064**	**0.012**	**4.80E-08**	**-+-?**	**21.5**	**0.280**						
rs12493341	a	g	**0.39**	**0.01**	**-0.053**	**0.010**	**4.92E-08**	**-+--**	**64.9**	**0.036**						
rs13074892	a	g	**0.74**	**0.00**	**-0.065**	**0.012**	**4.99E-08**	**-+-?**	**13.5**	**0.315**						
rs146071762	a	t	**0.51**	**0.02**							**0.248**	**0.044**	**1.14E-08**	**+-+?**	**69.2**	**0.039**
rs4298053	t	g	**0.30**	**0.00**							**0.127**	**0.024**	**1.49E-07**	**++++**	**62.9**	**0.044**
rs76847001	t	c	**0.32**	**0.00**							**-0.242**	**0.046**	**1.52E-07**	**-+-?**	**0**	**0.652**
rs9820133	t	g	**0.70**	**0.00**							**-0.126**	**0.024**	**1.77E-07**	**----**	**62.1**	**0.048**
rs7624423	a	g	**0.30**	**0.00**							**0.126**	**0.024**	**1.78E-07**	**++++**	**63.6**	**0.041**
rs4320069	a	t	**0.70**	**0.00**							**-0.126**	**0.024**	**1.78E-07**	**----**	**62.9**	**0.044**
rs11127905	t	c	**0.30**	**0.00**							**0.126**	**0.024**	**1.79E-07**	**++++**	**62.7**	**0.045**
rs12494196	t	c	**0.30**	**0.00**							**0.126**	**0.024**	**1.81E-07**	**++++**	**62.7**	**0.045**
rs4312666	t	c	**0.30**	**0.00**							**0.126**	**0.024**	**1.82E-07**	**++++**	**62.7**	**0.045**
rs9820262	c	g	**0.70**	**0.00**							**-0.126**	**0.024**	**1.82E-07**	**----**	**61.8**	**0.049**
rs9844720	t	c	**0.70**	**0.00**							**-0.126**	**0.024**	**1.92E-07**	**----**	**61.4**	**0.051**
rs28693686	t	c	**0.68**	**0.00**							**0.240**	**0.046**	**1.94E-07**	**+-+?**	**0**	**0.637**
rs9878917	a	g	**0.70**	**0.00**							**-0.125**	**0.024**	**1.95E-07**	**----**	**62.5**	**0.046**
rs4482674	t	c	**0.30**	**0.00**							**0.125**	**0.024**	**1.96E-07**	**++++**	**63.1**	**0.043**
rs4305433	c	g	**0.70**	**0.00**							**-0.125**	**0.024**	**1.98E-07**	**----**	**63**	**0.044**
rs6771804	a	t	**0.68**	**0.00**							**0.239**	**0.046**	**2.00E-07**	**+-+?**	**0**	**0.637**
rs4302392	a	g	**0.30**	**0.00**							**0.125**	**0.024**	**2.05E-07**	**++++**	**62.1**	**0.048**
rs6549047	a	c	**0.70**	**0.00**							**-0.125**	**0.024**	**2.08E-07**	**----**	**62.7**	**0.045**
rs4618235	a	g	**0.70**	**0.00**							**-0.125**	**0.024**	**2.11E-07**	**----**	**61.9**	**0.049**

rs10470567	a	t	**0.70**	**0.00**	**-0.125**	**0.024**	**2.12E-07**	**----**	**61.8**	**0.049**
rs9849768	t	c	**0.32**	**0.00**	**-0.239**	**0.046**	**2.19E-07**	**-+-?**	**0**	**0.647**
rs9812833	a	g	**0.68**	**0.00**	**0.238**	**0.046**	**2.19E-07**	**+-+?**	**0**	**0.644**
rs9835724	a	g	**0.68**	**0.00**	**0.238**	**0.046**	**2.41E-07**	**+-+?**	**0**	**0.638**
rs4129298	c	g	**0.70**	**0.00**	**-0.124**	**0.024**	**2.76E-07**	**----**	**58.9**	**0.063**
rs73147239	t	g	**0.30**	**0.00**	**0.125**	**0.024**	**2.78E-07**	**++++**	**61.8**	**0.049**
rs9817232	t	c	**0.30**	**0.00**	**0.124**	**0.024**	**2.85E-07**	**++++**	**63.6**	**0.041**
rs11127904	t	g	**0.70**	**0.00**	**-0.124**	**0.024**	**2.88E-07**	**----**	**63.1**	**0.043**
rs6792588	t	c	**0.30**	**0.00**	**0.124**	**0.024**	**2.96E-07**	**++++**	**61.3**	**0.052**
rs115164602	a	t	**0.29**	**0.00**	**0.127**	**0.025**	**3.12E-07**	**++++**	**63.7**	**0.041**
rs6549048	a	g	**0.30**	**0.00**	**0.124**	**0.024**	**3.23E-07**	**++++**	**62.5**	**0.046**
rs6781999	a	g	**0.30**	**0.00**	**0.123**	**0.024**	**3.37E-07**	**++++**	**62.3**	**0.047**
rs17334733	t	c	**0.70**	**0.00**	**-0.124**	**0.024**	**3.53E-07**	**----**	**61.9**	**0.049**
rs73147238	a	t	**0.70**	**0.00**	**-0.124**	**0.024**	**3.57E-07**	**----**	**62.4**	**0.047**
rs73147218	a	g	**0.70**	**0.00**	**-0.122**	**0.024**	**4.59E-07**	**----**	**62.9**	**0.044**
rs1947180	t	c	**0.70**	**0.00**	**-0.122**	**0.024**	**4.63E-07**	**----**	**61.8**	**0.049**
rs2017676	a	g	**0.30**	**0.00**	**0.122**	**0.024**	**4.68E-07**	**++++**	**61.5**	**0.050**
rs13315526	a	g	**0.31**	**0.00**	**-0.232**	**0.046**	**4.94E-07**	**-+-?**	**0**	**0.606**
rs76508707	t	c	**0.49**	**0.00**	**0.218**	**0.043**	**5.17E-07**	**+-+?**	**50.8**	**0.131**
rs73142855	a	g	**0.30**	**0.00**	**0.122**	**0.024**	**5.66E-07**	**++++**	**47.7**	**0.126**
rs73142850	c	g	**0.30**	**0.00**	**0.122**	**0.024**	**5.91E-07**	**++++**	**48.2**	**0.122**
rs10511074	t	c	**0.29**	**0.00**	**0.122**	**0.024**	**5.92E-07**	**++++**	**48.9**	**0.118**
rs2044654	a	t	**0.71**	**0.00**	**-0.123**	**0.025**	**5.95E-07**	**----**	**45.6**	**0.138**
rs73142849	a	t	**0.70**	**0.00**	**-0.121**	**0.024**	**6.29E-07**	**----**	**47.6**	**0.126**
rs2029051	c	g	**0.30**	**0.00**	**0.121**	**0.024**	**6.42E-07**	**++++**	**52.6**	**0.097**
rs73147253	t	c	**0.70**	**0.00**	**-0.121**	**0.024**	**6.69E-07**	**----**	**52**	**0.100**
rs2029052	a	g	**0.70**	**0.00**	**-0.121**	**0.024**	**6.78E-07**	**----**	**52.1**	**0.100**
rs6549061	a	g	**0.66**	**0.01**	**-0.116**	**0.023**	**6.91E-07**	**----**	**30.1**	**0.232**
rs4435654	a	g	**0.70**	**0.00**	**-0.120**	**0.024**	**7.25E-07**	**----**	**58.9**	**0.063**
rs73147242	a	t	**0.70**	**0.00**	**-0.120**	**0.024**	**7.58E-07**	**----**	**56.3**	**0.076**

rs7615964	c	g	**0.71**	**0.00**	**-0.121**	**0.024**	**8.14E-07**	**----**	**51.4**	**0.104**
rs2029053	a	t	**0.70**	**0.00**	**-0.120**	**0.024**	**8.21E-07**	**----**	**50.4**	**0.110**
rs114517674	a	t	**0.32**	**0.00**	**0.120**	**0.024**	**9.06E-07**	**++++**	**58.9**	**0.063**
rs9860249	a	g	**0.69**	**0.01**	**-0.117**	**0.024**	**1.20E-06**	**----**	**66.5**	**0.030**
rs4481168	c	g	**0.29**	**0.00**	**0.119**	**0.025**	**1.29E-06**	**++++**	**54.3**	**0.087**
rs4416395	a	g	**0.71**	**0.00**	**-0.118**	**0.025**	**1.31E-06**	**----**	**54.5**	**0.086**
rs73141536	t	g	**0.30**	**0.00**	**0.118**	**0.025**	**1.62E-06**	**++++**	**64.2**	**0.039**
rs6549052	a	c	**0.28**	**0.01**	**0.120**	**0.025**	**1.64E-06**	**++++**	**65.4**	**0.034**
rs2044653	a	g	**0.71**	**0.00**	**-0.121**	**0.025**	**1.64E-06**	**----**	**52.7**	**0.096**
rs67824428	t	c	**0.51**	**0.00**	**-0.200**	**0.043**	**2.79E-06**	**-+-?**	**24.7**	**0.265**
rs13062439	t	c	**0.70**	**0.02**	**0.123**	**0.027**	**6.06E-06**	**+-++**	**69.6**	**0.020**
rs13075494	t	c	**0.28**	**0.02**	**-0.120**	**0.027**	**6.25E-06**	**-+--**	**75.2**	**0.007**
rs7625311	t	c	**0.48**	**0.00**	**0.193**	**0.043**	**6.86E-06**	**-++?**	**0**	**0.811**
rs1694933	a	c	**0.48**	**0.00**	**0.193**	**0.043**	**7.08E-06**	**-++?**	**0**	**0.802**

Supplementary Table 2: SNPs reaching suggestive evidence of association with more than one phenotype.

			UK Biobank only									Meta-analysis									EA match
				risk-taking		mood instability		neuroticism				BMI			SBP			CRP			
SNP	A1	A2	A1F	BETA	P	BETA	P	BETA	P	A1	A2	A1F	BETA	P	A1F	BETA	P	A1F	BETA	P	
rs11708024	A	G								A	G	0.30	-0.07	1.60E-11				0.30	-0.069	6.05E-06	TRUE
rs4856605	A	G								A	G	0.32	-0.09	1.22E-17				0.32	-0.067	6.51E-06	TRUE
rs818225	T	C				-0.023	8.12E-07	-0.038	1.99E-06	T	C										TRUE
rs6803322	A	C								A	C	0.32	0.06	2.21E-08	0.32	-0.243	1.41E-07				TRUE
rs76847001	T	C								T	C	0.32	0.06	1.35E-07	0.32	-0.242	1.52E-07				TRUE
rs9849768	T	C								T	C	0.32	0.06	6.88E-08	0.32	-0.239	2.19E-07				TRUE
rs13315526	A	G								A	G	0.31	0.06	1.02E-07	0.31	-0.232	4.94E-07				TRUE
rs76508707	T	C	0.49	-0.035	1.50E-09			0.036	7.95E-06	T	C				0.49	0.218	5.17E-07				TRUE
rs7625311	T	C	0.48	-0.028	1.25E-06					T	C				0.48	0.193	6.86E-06				TRUE
rs1694933	A	C	0.48	-0.028	1.85E-06					A	C				0.48	0.193	7.08E-06				TRUE
rs7618124	A	G	0.35	0.051	2.02E-17			-0.039	3.30E-06	A	G	0.35	0.07	3.52E-11							TRUE
rs7618494	A	G	0.35	0.051	4.01E-17			-0.038	3.57E-06	A	G	0.35	0.07	7.96E-11							TRUE
rs1551044	T	C	0.35	0.051	2.82E-17			-0.038	3.71E-06	T	C	0.35	0.07	4.21E-11							TRUE
rs12637791	T	G	0.35	0.051	3.25E-17			-0.038	3.73E-06	T	G	0.35	0.07	4.37E-11							TRUE
rs10433525	T	C	0.35	0.051	2.77E-17			-0.038	3.76E-06	T	C	0.35	0.07	4.05E-11							TRUE
rs6549030	T	C	0.35	0.051	2.64E-17			-0.038	3.89E-06	T	C	0.35	0.07	3.89E-11							TRUE
rs62250713	A	G	0.36	0.053	5.96E-19			-0.038	3.98E-06	A	G	0.36	0.07	1.30E-11							TRUE
rs7652808	T	G	0.35	0.051	2.89E-17			-0.038	4.14E-06	T	G	0.34	0.06	1.21E-10							TRUE
rs4856584	A	T	0.35	0.051	3.33E-17			-0.038	4.17E-06	A	T	0.35	0.07	9.12E-11							TRUE
rs7652683	T	C	0.35	0.051	2.92E-17			-0.038	4.20E-06	T	C	0.34	0.06	1.22E-10							TRUE
rs3911063	C	T								T	C	0.68	0.09	1.11E-17				0.68	0.068	5.07E-06	FALSE
rs57533494	C	A								A	C	0.68	0.09	1.38E-16				0.68	0.069	5.09E-06	FALSE
rs57153235	G	T								T	G	0.68	0.09	1.08E-16				0.68	0.068	5.11E-06	FALSE
rs62261725	G	A								A	G	0.67	0.09	9.95E-18				0.68	0.067	5.92E-06	FALSE
rs10865612	C	T	0.35	-0.028	3.15E-06					T	C	0.64	0.09	4.50E-19				0.63	0.065	7.26E-06	FALSE
rs7431895	C	T	0.35	-0.028	4.33E-06					T	C	0.64	0.09	1.07E-18				0.63	0.064	9.61E-06	FALSE
rs61439785	G	A	0.35	-0.028	3.72E-06					A	G	0.64	0.09	7.54E-19				0.64	0.064	9.68E-06	FALSE
rs58382609	G	T	0.35	-0.028	3.61E-06					T	G	0.64	0.09	7.61E-19				0.64	0.064	9.85E-06	FALSE
rs818215	C	A				-0.023	9.96E-07	-0.038	1.58E-06	A	C										FALSE
rs818219	C	T				-0.023	1.05E-06	-0.038	1.54E-06	T	C										FALSE

rs146071762	T	A	0.49	0.031	1.41E-07			A	T				0.51	0.248	1.14E-08	FALSE
rs28693686	C	T						T	C	0.68	-0.06	6.02E-08	0.68	0.240	1.94E-07	FALSE
rs6771804	T	A						A	T	0.68	-0.06	5.22E-08	0.68	0.239	2.00E-07	FALSE
rs9812833	G	A						A	G	0.68	-0.06	6.80E-08	0.68	0.238	2.19E-07	FALSE
rs9835724	G	A						A	G	0.68	-0.06	6.51E-08	0.68	0.238	2.41E-07	FALSE
rs67824428	C	T	0.49	-0.030	2.74E-07	0.036	6.34E-06	T	C				0.51	-0.200	2.79E-06	FALSE
rs13062439	C	T						T	C	0.68	-0.05	1.00E-06	0.70	0.123	6.06E-06	FALSE
rs4637303	C	T	0.35	0.051	3.36E-17	-0.039	2.97E-06	T	C	0.65	-0.07	4.42E-11				FALSE
rs12495758	G	A	0.35	0.051	3.09E-17	-0.038	3.50E-06	A	G	0.65	-0.07	2.91E-11				FALSE
rs726610	C	T	0.35	0.051	2.04E-17	-0.038	3.78E-06	T	C	0.66	-0.07	6.01E-11				FALSE
rs10433499	C	T	0.35	0.051	2.92E-17	-0.038	3.82E-06	T	C	0.65	-0.07	4.02E-11				FALSE
rs754061121	ATC	A	0.35	0.051	5.29E-17	-0.039	2.84E-06									
rs557667709	T	TCTC	0.35	0.050	7.45E-17	-0.039	3.27E-06									
rs568187754	G	A	0.22	0.050	5.14E-13	-0.044	3.55E-06									
rs530949414	G	A	0.22	0.050	5.18E-13	-0.044	3.55E-06									
rs549942803	A	T	0.22	0.050	5.18E-13	-0.044	3.55E-06									
rs551414092	G	A	0.22	0.050	5.18E-13	-0.044	3.55E-06									
rs569822025	A	T	0.22	0.050	5.18E-13	-0.044	3.55E-06									
3:85541934	CT	C	0.35	0.051	3.56E-17	-0.038	4.02E-06									

Supplementary Table 3: EQTLs for *CADM2* and *CADM2-AS1*

Gene Symbol	SNP Id	P-Value	NES	Tissue
CADM2		7.10E-05	0.23	Adipose - Visceral (Omentum)
CADM2	rs10049108	2.50E-06	0.28	Lung
CADM2-AS1		1.70E-05	0.56	Brain - Caudate (basal ganglia)
CADM2-AS1		4.60E-08	0.66	Brain - Cerebellar Hemisphere
CADM2-AS1		6.60E-08	0.52	Brain - Cerebellum
CADM2-AS1		3.60E-05	0.48	Brain - Cortex
CADM2-AS1		1.10E-06	0.61	Brain - Frontal Cortex (BA9)
CADM2-AS1	rs10084716	4.70E-06	0.55	Brain - Nucleus accumbens (basal ganglia)
CADM2	rs1013839	1.20E-04	0.14	Muscle - Skeletal
CADM2	rs1014796	1.30E-07	-0.35	Lung
CADM2		1.60E-07	0.27	Adipose - Subcutaneous
CADM2		8.00E-06	0.25	Adipose - Visceral (Omentum)
CADM2	rs10154865	4.00E-09	0.35	Lung
CADM2		1.10E-06	0.2	Adipose - Subcutaneous
CADM2	rs1016305	1.10E-07	0.19	Muscle - Skeletal
CADM2		1.00E-06	0.2	Adipose - Subcutaneous
CADM2	rs1016306	5.60E-08	0.2	Muscle - Skeletal
CADM2		1.50E-08	0.27	Adipose - Subcutaneous
CADM2		4.40E-05	0.21	Adipose - Visceral (Omentum)
CADM2	rs1025531	1.60E-06	0.27	Lung
CADM2		3.50E-08	0.26	Adipose - Subcutaneous
CADM2		1.80E-05	0.22	Adipose - Visceral (Omentum)
CADM2	rs1025533	8.50E-07	0.27	Lung
CADM2		7.90E-05	-0.43	Heart - Left Ventricle
CADM2	rs1030719	2.80E-07	-0.34	Lung
CADM2		1.30E-05	-0.47	Heart - Left Ventricle
CADM2	rs1030720	1.30E-06	-0.31	Lung
CADM2		3.20E-08	0.26	Adipose - Subcutaneous
CADM2		2.20E-05	0.22	Adipose - Visceral (Omentum)
CADM2	rs1030721	8.50E-07	0.27	Lung
CADM2-AS1	rs1033139	8.80E-05	-0.28	Nerve - Tibial
CADM2		3.10E-08	0.26	Adipose - Subcutaneous
CADM2		2.00E-05	0.22	Adipose - Visceral (Omentum)
CADM2	rs10433498	1.10E-06	0.27	Lung
CADM2		5.00E-09	-0.24	Adipose - Subcutaneous
CADM2		5.50E-07	-0.23	Adipose - Visceral (Omentum)
CADM2		2.00E-07	-0.4	Heart - Left Ventricle
CADM2	rs10433499	4.20E-19	-0.41	Lung
CADM2		7.30E-10	-0.26	Adipose - Subcutaneous
CADM2		1.10E-07	-0.24	Adipose - Visceral (Omentum)
CADM2		1.50E-06	-0.38	Heart - Left Ventricle
CADM2	rs10433500	2.70E-16	-0.39	Lung
CADM2	rs10433523	5.80E-05	0.16	Adipose - Subcutaneous
CADM2		3.70E-09	-0.24	Adipose - Subcutaneous
CADM2		1.00E-07	-0.24	Adipose - Visceral (Omentum)
CADM2		1.90E-07	-0.39	Heart - Left Ventricle
CADM2	rs10433525	2.10E-19	-0.41	Lung
CADM2	rs10490895	1.40E-08	1.1	Heart - Left Ventricle
CADM2-AS1	rs10511067	1.90E-07	0.59	Brain - Caudate (basal ganglia)

CADM2-AS1		3.10E-05	0.47	Brain - Cerebellar Hemisphere
CADM2-AS1		3.10E-07	0.48	Brain - Cerebellum
CADM2-AS1		1.50E-07	0.62	Brain - Caudate (basal ganglia)
CADM2-AS1		2.80E-06	0.54	Brain - Cerebellar Hemisphere
CADM2-AS1		3.00E-08	0.53	Brain - Cerebellum
CADM2-AS1	rs10511068	4.30E-05	0.46	Brain - Cortex
CADM2		6.30E-05	-0.17	Adipose - Subcutaneous
CADM2		7.70E-05	-0.31	Heart - Left Ventricle
CADM2	rs10511073	1.90E-11	-0.32	Lung
CADM2		7.60E-12	-0.29	Adipose - Subcutaneous
CADM2		3.30E-08	-0.25	Adipose - Visceral (Omentum)
CADM2		1.30E-06	-0.38	Heart - Left Ventricle
CADM2		2.00E-18	-0.41	Lung
CADM2	rs10511075	1.80E-04	0.098	Nerve - Tibial
CADM2		6.70E-12	-0.29	Adipose - Subcutaneous
CADM2		3.10E-08	-0.25	Adipose - Visceral (Omentum)
CADM2		6.40E-07	-0.39	Heart - Left Ventricle
CADM2		1.80E-18	-0.41	Lung
CADM2	rs10511076	1.60E-04	0.099	Nerve - Tibial
CADM2		1.00E-09	-0.25	Adipose - Subcutaneous
CADM2		1.00E-07	-0.24	Adipose - Visceral (Omentum)
CADM2		2.00E-07	-0.39	Heart - Left Ventricle
CADM2	rs10511078	8.80E-20	-0.41	Lung
CADM2		6.80E-12	-0.29	Adipose - Subcutaneous
CADM2		3.20E-08	-0.25	Adipose - Visceral (Omentum)
CADM2		7.60E-07	-0.39	Heart - Left Ventricle
CADM2		5.10E-18	-0.4	Lung
CADM2	rs10511081	1.60E-04	0.099	Nerve - Tibial
CADM2		1.20E-06	-0.21	Adipose - Subcutaneous
CADM2	rs10511082	6.60E-09	-0.27	Lung
CADM2		3.70E-12	-0.29	Adipose - Subcutaneous
CADM2		3.10E-08	-0.25	Adipose - Visceral (Omentum)
CADM2		6.60E-07	-0.39	Heart - Left Ventricle
CADM2	rs10511083	3.10E-18	-0.41	Lung
CADM2		7.10E-06	-0.19	Adipose - Subcutaneous
CADM2		9.20E-10	-0.28	Lung
CADM2-AS1	rs10511084	1.40E-04	0.25	Nerve - Tibial
CADM2		1.20E-10	-0.27	Adipose - Subcutaneous
CADM2		4.10E-07	-0.24	Adipose - Visceral (Omentum)
CADM2		1.50E-06	-0.38	Heart - Left Ventricle
CADM2	rs10511085	6.00E-17	-0.4	Lung
CADM2		1.90E-10	-0.27	Adipose - Subcutaneous
CADM2		3.50E-07	-0.24	Adipose - Visceral (Omentum)
CADM2		5.30E-07	-0.42	Heart - Left Ventricle
CADM2	rs10511087	4.30E-15	-0.38	Lung
CADM2	rs10511088	9.20E-06	-0.66	Brain - Hippocampus
CADM2	rs10537979	6.10E-05	0.27	Lung
CADM2		2.70E-10	-0.26	Adipose - Subcutaneous
CADM2		3.30E-08	-0.24	Adipose - Visceral (Omentum)
CADM2		2.10E-07	-0.4	Heart - Left Ventricle
CADM2	rs10542190	1.00E-14	-0.36	Lung

CADM2		7.10E-05	-0.15	Muscle - Skeletal
CADM2		1.30E-04	0.2	Adipose - Subcutaneous
CADM2	rs10547651	2.80E-06	0.28	Lung
CADM2-AS1		3.80E-05	0.5	Brain - Cerebellar Hemisphere
CADM2-AS1	rs10563317	3.10E-05	0.48	Brain - Nucleus accumbens (basal ganglia)
CADM2	rs10605493	2.10E-05	0.17	Muscle - Skeletal
CADM2-AS1		1.20E-05	0.58	Brain - Caudate (basal ganglia)
CADM2-AS1		5.40E-08	0.66	Brain - Cerebellar Hemisphere
CADM2-AS1		2.70E-07	0.49	Brain - Cerebellum
CADM2-AS1		9.00E-06	0.5	Brain - Cortex
CADM2-AS1		1.40E-06	0.61	Brain - Frontal Cortex (BA9)
CADM2-AS1	rs10623835	2.80E-06	0.56	Brain - Nucleus accumbens (basal ganglia)
CADM2	rs10689782	5.80E-06	0.18	Muscle - Skeletal
CADM2		2.90E-12	-0.29	Adipose - Subcutaneous
CADM2		9.00E-09	-0.26	Adipose - Visceral (Omentum)
CADM2		6.40E-07	-0.39	Heart - Left Ventricle
CADM2	rs10716596	8.00E-18	-0.4	Lung
CADM2-AS1		8.30E-06	0.57	Brain - Caudate (basal ganglia)
CADM2-AS1		1.20E-07	0.63	Brain - Cerebellar Hemisphere
CADM2-AS1		2.00E-08	0.53	Brain - Cerebellum
CADM2-AS1		3.70E-05	0.48	Brain - Cortex
CADM2-AS1		4.80E-06	0.57	Brain - Frontal Cortex (BA9)
CADM2-AS1	rs10779988	9.20E-06	0.51	Brain - Nucleus accumbens (basal ganglia)
CADM2	rs10865609	1.20E-07	-0.35	Lung
CADM2		1.00E-11	-0.28	Adipose - Subcutaneous
CADM2		3.00E-08	-0.25	Adipose - Visceral (Omentum)
CADM2		3.10E-07	-0.4	Heart - Left Ventricle
CADM2		3.80E-16	-0.37	Lung
CADM2	rs10865610	7.20E-05	-0.15	Muscle - Skeletal
CADM2		2.30E-09	-0.25	Adipose - Subcutaneous
CADM2		4.40E-07	-0.23	Adipose - Visceral (Omentum)
CADM2		3.50E-07	-0.38	Heart - Left Ventricle
CADM2	rs10865611	4.00E-19	-0.41	Lung
CADM2-AS1		1.10E-06	0.55	Brain - Caudate (basal ganglia)
CADM2-AS1		8.70E-08	0.49	Brain - Cerebellum
CADM2-AS1	rs10865612	1.30E-04	0.25	Nerve - Tibial
CADM2	rs11127870	2.80E-08	1	Heart - Left Ventricle
CADM2	rs11127871	2.80E-08	1	Heart - Left Ventricle
CADM2	rs11127872	4.40E-08	1	Heart - Left Ventricle
CADM2		2.30E-05	0.16	Adipose - Subcutaneous
CADM2	rs11127873	1.50E-04	0.13	Muscle - Skeletal
CADM2	rs11127874	4.20E-06	-0.69	Brain - Hippocampus
CADM2	rs11127875	4.20E-06	-0.69	Brain - Hippocampus
CADM2		7.10E-05	0.15	Adipose - Subcutaneous
CADM2	rs11127876	8.80E-06	0.16	Muscle - Skeletal
CADM2		3.10E-05	0.16	Adipose - Subcutaneous
CADM2	rs11127877	7.00E-05	0.14	Muscle - Skeletal
CADM2	rs11127880	1.50E-04	0.15	Adipose - Subcutaneous
CADM2	rs11127885	1.10E-05	-0.75	Testis

CADM2	rs11127886	1.50E-05	-0.74	Testis
CADM2		1.50E-08	0.26	Adipose - Subcutaneous
CADM2		7.30E-05	0.2	Adipose - Visceral (Omentum)
CADM2		6.10E-05	0.21	Breast - Mammary Tissue
CADM2	rs11127887	2.90E-06	0.26	Lung
CADM2		3.00E-08	0.26	Adipose - Subcutaneous
CADM2		1.50E-05	0.22	Adipose - Visceral (Omentum)
CADM2	rs11127890	8.20E-07	0.28	Lung
CADM2		1.70E-08	0.27	Adipose - Subcutaneous
CADM2		2.00E-05	0.22	Adipose - Visceral (Omentum)
CADM2	rs11127891	1.50E-06	0.27	Lung
CADM2		2.50E-09	-0.25	Adipose - Subcutaneous
CADM2		1.80E-07	-0.23	Adipose - Visceral (Omentum)
CADM2		1.40E-07	-0.4	Heart - Left Ventricle
CADM2	rs11127893	1.90E-19	-0.41	Lung
CADM2		3.70E-12	-0.29	Adipose - Subcutaneous
CADM2		1.10E-08	-0.25	Adipose - Visceral (Omentum)
CADM2		5.90E-07	-0.39	Heart - Left Ventricle
CADM2	rs11127895	4.60E-18	-0.41	Lung
CADM2		5.00E-12	-0.29	Adipose - Subcutaneous
CADM2		1.50E-08	-0.25	Adipose - Visceral (Omentum)
CADM2		1.70E-06	-0.37	Heart - Left Ventricle
CADM2	rs11127896	4.00E-19	-0.42	Lung
CADM2		2.60E-09	-0.25	Adipose - Subcutaneous
CADM2		1.50E-07	-0.24	Adipose - Visceral (Omentum)
CADM2		1.50E-07	-0.4	Heart - Left Ventricle
CADM2	rs11127897	6.50E-20	-0.42	Lung
CADM2		1.80E-09	-0.25	Adipose - Subcutaneous
CADM2		9.50E-08	-0.24	Adipose - Visceral (Omentum)
CADM2		1.20E-07	-0.4	Heart - Left Ventricle
CADM2	rs11127898	2.20E-19	-0.41	Lung
CADM2		7.30E-12	-0.29	Adipose - Subcutaneous
CADM2		1.40E-08	-0.25	Adipose - Visceral (Omentum)
CADM2		9.10E-07	-0.39	Heart - Left Ventricle
CADM2		2.00E-18	-0.41	Lung
CADM2	rs11127899	1.70E-04	0.099	Nerve - Tibial
CADM2		6.80E-12	-0.29	Adipose - Subcutaneous
CADM2		1.50E-08	-0.25	Adipose - Visceral (Omentum)
CADM2		7.40E-07	-0.39	Heart - Left Ventricle
CADM2		1.80E-18	-0.41	Lung
CADM2	rs11127900	1.60E-04	0.099	Nerve - Tibial
CADM2		1.30E-04	0.2	Adipose - Subcutaneous
CADM2		1.60E-05	0.26	Lung
CADM2-AS1	rs11127903	8.30E-05	-0.3	Nerve - Tibial
CADM2-AS1	rs11127905	3.10E-05	0.47	Brain - Nucleus accumbens (basal ganglia)
CADM2-AS1		3.40E-05	0.56	Brain - Caudate (basal ganglia)
CADM2-AS1	rs11127907	1.20E-08	0.6	Brain - Cerebellum
CADM2-AS1		3.60E-07	0.6	Brain - Caudate (basal ganglia)
CADM2-AS1		5.50E-06	0.53	Brain - Cerebellar Hemisphere
CADM2-AS1	rs11127908	3.00E-08	0.53	Brain - Cerebellum
CADM2-AS1		4.80E-06	0.58	Brain - Caudate (basal ganglia)
CADM2-AS1	rs11127911	5.60E-08	0.64	Brain - Cerebellar Hemisphere

CADM2-AS1		8.20E-08	0.51	Brain - Cerebellum
CADM2-AS1		1.80E-05	0.5	Brain - Cortex
CADM2-AS1		1.40E-06	0.59	Brain - Frontal Cortex (BA9)
CADM2-AS1		2.70E-06	0.54	Brain - Nucleus accumbens (basal ganglia)
CADM2-AS1		1.60E-06	0.58	Brain - Caudate (basal ganglia)
CADM2-AS1		6.10E-10	0.7	Brain - Cerebellar Hemisphere
CADM2-AS1		1.30E-10	0.61	Brain - Cerebellum
CADM2-AS1		1.40E-05	0.53	Brain - Frontal Cortex (BA9)
CADM2-AS1		2.90E-05	0.54	Brain - Hippocampus
CADM2-AS1	rs11127912	3.60E-07	0.61	Brain - Nucleus accumbens (basal ganglia)
CADM2-AS1		4.60E-07	0.62	Brain - Caudate (basal ganglia)
CADM2-AS1		6.00E-10	0.71	Brain - Cerebellar Hemisphere
CADM2-AS1		1.40E-11	0.63	Brain - Cerebellum
CADM2-AS1		5.30E-06	0.57	Brain - Frontal Cortex (BA9)
CADM2-AS1		1.50E-05	0.58	Brain - Hippocampus
CADM2-AS1		3.20E-07	0.61	Brain - Nucleus accumbens (basal ganglia)
CADM2-AS1	rs11127913	5.70E-05	0.26	Nerve - Tibial
CADM2-AS1		1.50E-07	0.62	Brain - Caudate (basal ganglia)
CADM2-AS1		2.50E-10	0.71	Brain - Cerebellar Hemisphere
CADM2-AS1		1.40E-11	0.62	Brain - Cerebellum
CADM2-AS1		1.50E-05	0.53	Brain - Frontal Cortex (BA9)
CADM2-AS1		3.40E-07	0.59	Brain - Nucleus accumbens (basal ganglia)
CADM2-AS1	rs11127914	5.40E-05	0.26	Nerve - Tibial
CADM2-AS1		3.40E-05	0.54	Brain - Cerebellar Hemisphere
CADM2-AS1		2.10E-06	0.47	Brain - Cerebellum
CADM2-AS1	rs11127917	4.70E-05	0.47	Brain - Cortex
CADM2-AS1		7.50E-07	0.61	Brain - Caudate (basal ganglia)
CADM2-AS1		1.80E-07	0.66	Brain - Cerebellar Hemisphere
CADM2-AS1		8.70E-09	0.56	Brain - Cerebellum
CADM2-AS1		3.50E-07	0.7	Brain - Frontal Cortex (BA9)
CADM2-AS1	rs11127918	1.10E-05	0.57	Brain - Nucleus accumbens (basal ganglia)
CADM2-AS1	rs11127920	3.50E-05	0.4	Brain - Cerebellum
CADM2-AS1	rs111313874	3.10E-05	0.47	Brain - Nucleus accumbens (basal ganglia)
CADM2	rs111566692	1.30E-06	0.84	Heart - Left Ventricle
CADM2		5.20E-08	0.25	Adipose - Subcutaneous
CADM2		3.80E-05	0.2	Adipose - Visceral (Omentum)
CADM2	rs111758983	2.50E-06	0.26	Lung
CADM2	rs111871374	9.20E-06	-0.66	Brain - Hippocampus
CADM2	rs112042139	3.90E-05	0.51	Heart - Left Ventricle
CADM2	rs11277588	2.30E-05	-0.2	Lung
CADM2		2.10E-09	-0.25	Adipose - Subcutaneous
CADM2		1.10E-07	-0.24	Adipose - Visceral (Omentum)
CADM2		1.60E-07	-0.4	Heart - Left Ventricle
CADM2	rs11288859	8.80E-20	-0.41	Lung
CADM2		6.20E-06	-0.2	Adipose - Subcutaneous
CADM2		2.30E-09	-0.27	Lung
CADM2-AS1	rs112911909	1.20E-04	0.25	Nerve - Tibial
CADM2	rs11293900	2.20E-08	0.26	Adipose - Subcutaneous

CADM2		1.80E-05	0.22	Adipose - Visceral (Omentum)
CADM2		1.20E-06	0.27	Lung
CADM2	rs112987098	5.30E-07	-0.57	Brain - Spinal cord (cervical c-1)
CADM2		7.10E-12	-0.29	Adipose - Subcutaneous
CADM2		1.50E-08	-0.25	Adipose - Visceral (Omentum)
CADM2		6.70E-07	-0.39	Heart - Left Ventricle
CADM2		1.80E-18	-0.41	Lung
CADM2	rs113351222	1.60E-04	0.1	Nerve - Tibial
CADM2		3.40E-10	-0.28	Adipose - Subcutaneous
CADM2		2.00E-09	-0.29	Adipose - Visceral (Omentum)
CADM2		1.50E-05	-0.37	Heart - Left Ventricle
CADM2		1.90E-15	-0.4	Lung
CADM2	rs11337112	1.30E-04	-0.16	Muscle - Skeletal
CADM2-AS1		3.40E-05	0.54	Brain - Cerebellar Hemisphere
CADM2-AS1		2.10E-06	0.47	Brain - Cerebellum
CADM2-AS1	rs113626937	4.70E-05	0.47	Brain - Cortex
CADM2		1.80E-11	-0.28	Adipose - Subcutaneous
CADM2		4.40E-08	-0.25	Adipose - Visceral (Omentum)
CADM2		2.00E-07	-0.41	Heart - Left Ventricle
CADM2	rs11365888	5.00E-16	-0.38	Lung
CADM2	rs11371724	1.10E-05	-0.75	Testis
CADM2-AS1	rs11372396	3.10E-05	0.47	Brain - Nucleus accumbens (basal ganglia)
CADM2		1.80E-10	-0.28	Adipose - Subcutaneous
CADM2		1.80E-08	-0.26	Adipose - Visceral (Omentum)
CADM2		6.50E-07	-0.39	Heart - Left Ventricle
CADM2		3.80E-14	-0.36	Lung
CADM2	rs11376660	4.50E-05	-0.16	Muscle - Skeletal
CADM2	rs113850292	2.60E-08	1	Heart - Left Ventricle
CADM2		1.60E-05	0.16	Adipose - Subcutaneous
CADM2	rs11427455	6.60E-05	0.14	Muscle - Skeletal
CADM2	rs1146751	5.30E-07	-0.57	Brain - Spinal cord (cervical c-1)
CADM2	rs115224651	3.70E-05	0.2	Brain - Cerebellum
CADM2	rs115433211	2.20E-05	0.78	Heart - Atrial Appendage
CADM2		1.60E-12	-0.31	Adipose - Subcutaneous
CADM2		6.60E-08	-0.26	Adipose - Visceral (Omentum)
CADM2		1.20E-07	-0.43	Heart - Left Ventricle
CADM2		2.00E-13	-0.36	Lung
CADM2	rs1155666	1.10E-04	0.11	Nerve - Tibial
CADM2		3.20E-05	0.18	Adipose - Subcutaneous
CADM2	rs11705775	9.20E-05	0.2	Lung
CADM2		2.90E-07	0.67	Brain - Spinal cord (cervical c-1)
CADM2	rs11707605	3.80E-05	0.17	Muscle - Skeletal
CADM2-AS1	rs11708023	5.50E-05	0.41	Brain - Cerebellum
CADM2-AS1		2.20E-05	0.56	Brain - Caudate (basal ganglia)
CADM2-AS1		1.50E-05	0.58	Brain - Cerebellar Hemisphere
CADM2-AS1	rs11708024	4.60E-08	0.58	Brain - Cerebellum
CADM2-AS1		4.20E-07	0.6	Brain - Caudate (basal ganglia)
CADM2-AS1		6.50E-09	0.66	Brain - Cerebellar Hemisphere
CADM2-AS1		2.90E-10	0.58	Brain - Cerebellum
CADM2-AS1		1.70E-05	0.52	Brain - Frontal Cortex (BA9)
CADM2-AS1	rs11708377	1.30E-05	0.56	Brain - Hippocampus

CADM2-AS1		3.50E-06	0.53	Brain - Nucleus accumbens (basal ganglia)
CADM2		3.80E-05	0.19	Adipose - Subcutaneous
CADM2		4.50E-07	0.64	Brain - Spinal cord (cervical c-1)
CADM2	rs11708447	9.00E-05	0.16	Muscle - Skeletal
CADM2-AS1		3.80E-05	0.5	Brain - Cerebellar Hemisphere
CADM2-AS1	rs11708601	3.10E-05	0.48	Brain - Nucleus accumbens (basal ganglia)
CADM2		5.10E-05	0.23	Adipose - Visceral (Omentum)
CADM2	rs11708632	6.40E-06	0.27	Lung
CADM2-AS1		2.40E-06	0.54	Brain - Cerebellar Hemisphere
CADM2-AS1		7.20E-08	0.51	Brain - Cerebellum
CADM2-AS1	rs11711972	2.70E-05	0.5	Brain - Nucleus accumbens (basal ganglia)
CADM2		3.10E-08	0.29	Adipose - Subcutaneous
CADM2		6.80E-06	0.25	Adipose - Visceral (Omentum)
CADM2	rs11712915	4.50E-09	0.34	Lung
CADM2	rs11713152	5.10E-06	0.64	Brain - Spinal cord (cervical c-1)
CADM2	rs11713170	1.90E-06	0.17	Muscle - Skeletal
CADM2		5.90E-08	0.28	Adipose - Subcutaneous
CADM2		6.80E-06	0.25	Adipose - Visceral (Omentum)
CADM2	rs11713902	4.70E-09	0.34	Lung
CADM2-AS1		3.80E-05	0.5	Brain - Cerebellar Hemisphere
CADM2-AS1	rs11714008	3.10E-05	0.48	Brain - Nucleus accumbens (basal ganglia)
CADM2		3.90E-05	-0.48	Heart - Left Ventricle
CADM2	rs11714549	2.60E-05	-0.29	Lung
CADM2-AS1		1.40E-07	0.62	Brain - Caudate (basal ganglia)
CADM2-AS1		2.80E-06	0.54	Brain - Cerebellar Hemisphere
CADM2-AS1		2.00E-08	0.54	Brain - Cerebellum
CADM2-AS1	rs11714569	2.00E-05	0.5	Brain - Nucleus accumbens (basal ganglia)
CADM2		1.50E-04	0.17	Adipose - Subcutaneous
CADM2	rs11715683	4.20E-07	0.73	Brain - Spinal cord (cervical c-1)
CADM2-AS1		2.10E-08	0.66	Brain - Cerebellar Hemisphere
CADM2-AS1		1.40E-06	0.46	Brain - Cerebellum
CADM2-AS1		2.60E-05	0.47	Brain - Cortex
CADM2-AS1		2.60E-06	0.59	Brain - Frontal Cortex (BA9)
CADM2-AS1	rs11715949	3.40E-06	0.55	Brain - Nucleus accumbens (basal ganglia)
CADM2		1.40E-07	0.27	Adipose - Subcutaneous
CADM2		1.60E-05	0.24	Adipose - Visceral (Omentum)
CADM2	rs11716233	5.50E-09	0.34	Lung
CADM2		3.60E-05	0.19	Adipose - Subcutaneous
CADM2		4.50E-07	0.64	Brain - Spinal cord (cervical c-1)
CADM2	rs11716886	2.80E-05	0.17	Muscle - Skeletal
CADM2		2.90E-07	0.67	Brain - Spinal cord (cervical c-1)
CADM2	rs11719183	4.80E-05	0.17	Muscle - Skeletal
CADM2		1.50E-04	0.18	Adipose - Subcutaneous
CADM2	rs11719276	3.30E-07	0.77	Brain - Spinal cord (cervical c-1)
CADM2-AS1		2.70E-07	0.62	Brain - Caudate (basal ganglia)
CADM2-AS1		1.80E-10	0.71	Brain - Cerebellar Hemisphere
CADM2-AS1		1.90E-12	0.63	Brain - Cerebellum
CADM2-AS1		2.30E-06	0.57	Brain - Frontal Cortex (BA9)
CADM2-AS1		2.40E-05	0.56	Brain - Hippocampus
CADM2-AS1	rs11719943	8.20E-08	0.62	Brain - Nucleus accumbens (basal ganglia)
CADM2	rs11721040	7.70E-05	0.21	Adipose - Subcutaneous

CADM2-AS1		1.40E-07	0.59	Brain - Caudate (basal ganglia)
CADM2-AS1		3.00E-05	0.47	Brain - Cerebellar Hemisphere
CADM2-AS1	rs11914525	1.10E-07	0.49	Brain - Cerebellum
CADM2		8.60E-07	-0.23	Lung
CADM2-AS1	rs11915747	1.30E-04	0.25	Nerve - Tibial
CADM2	rs11917003	1.00E-04	-0.2	Adipose - Subcutaneous
CADM2		2.90E-09	-0.27	Adipose - Subcutaneous
CADM2		2.40E-07	-0.26	Adipose - Visceral (Omentum)
CADM2		5.20E-05	-0.21	Breast - Mammary Tissue
CADM2	rs11917490	5.10E-10	-0.33	Lung
CADM2		1.30E-10	-0.27	Adipose - Subcutaneous
CADM2		7.00E-08	-0.25	Adipose - Visceral (Omentum)
CADM2		2.10E-07	-0.42	Heart - Left Ventricle
CADM2	rs11918899	6.00E-17	-0.4	Lung
CADM2		2.20E-11	-0.28	Adipose - Subcutaneous
CADM2		4.70E-08	-0.24	Adipose - Visceral (Omentum)
CADM2		1.30E-06	-0.38	Heart - Left Ventricle
CADM2	rs11919099	3.60E-18	-0.4	Lung
CADM2		4.30E-06	-0.2	Adipose - Subcutaneous
CADM2	rs11920184	5.70E-10	-0.28	Lung
CADM2-AS1		4.90E-05	0.53	Brain - Cerebellar Hemisphere
CADM2-AS1	rs11920350	2.20E-06	0.47	Brain - Cerebellum
CADM2		1.90E-09	-0.25	Adipose - Subcutaneous
CADM2		1.30E-06	-0.22	Adipose - Visceral (Omentum)
CADM2		5.00E-05	-0.19	Breast - Mammary Tissue
CADM2		1.70E-06	-0.38	Heart - Left Ventricle
CADM2		1.20E-15	-0.38	Lung
CADM2	rs11921010	7.30E-05	0.1	Nerve - Tibial
CADM2		1.70E-11	-0.29	Adipose - Subcutaneous
CADM2		2.30E-08	-0.25	Adipose - Visceral (Omentum)
CADM2		2.50E-06	-0.37	Heart - Left Ventricle
CADM2	rs11922956	3.10E-17	-0.4	Lung
CADM2		2.80E-06	-0.2	Adipose - Subcutaneous
CADM2		1.00E-04	-0.17	Adipose - Visceral (Omentum)
CADM2	rs11923343	4.30E-11	-0.32	Lung
CADM2		2.80E-11	-0.28	Adipose - Subcutaneous
CADM2		9.70E-09	-0.26	Adipose - Visceral (Omentum)
CADM2		5.50E-07	-0.39	Heart - Left Ventricle
CADM2		8.50E-18	-0.4	Lung
CADM2	rs11923525	1.80E-04	0.099	Nerve - Tibial
CADM2-AS1		3.70E-05	0.55	Brain - Caudate (basal ganglia)
CADM2-AS1		1.60E-07	0.65	Brain - Cerebellar Hemisphere
CADM2-AS1		7.20E-08	0.52	Brain - Cerebellum
CADM2-AS1		6.00E-06	0.51	Brain - Cortex
CADM2-AS1		1.10E-06	0.61	Brain - Frontal Cortex (BA9)
CADM2-AS1	rs11924631	1.30E-06	0.57	Brain - Nucleus accumbens (basal ganglia)
CADM2-AS1	rs11925916	3.40E-05	0.49	Brain - Cerebellar Hemisphere
CADM2	rs11926317	1.20E-04	0.14	Muscle - Skeletal
CADM2-AS1		1.40E-07	0.59	Brain - Caudate (basal ganglia)
CADM2-AS1		3.30E-05	0.47	Brain - Cerebellar Hemisphere
CADM2-AS1	rs11926422	6.00E-08	0.5	Brain - Cerebellum

CADM2		1.30E-06	-0.21	Adipose - Subcutaneous
CADM2	rs11927145	3.20E-09	-0.27	Lung
CADM2		2.10E-05	-0.21	Lung
CADM2-AS1	rs11928368	1.00E-04	0.26	Nerve - Tibial
CADM2		1.20E-05	-0.21	Adipose - Subcutaneous
CADM2	rs11929182	2.40E-06	-0.25	Lung
CADM2		1.10E-09	-0.26	Adipose - Subcutaneous
CADM2		1.50E-07	-0.24	Adipose - Visceral (Omentum)
CADM2		5.10E-07	-0.41	Heart - Left Ventricle
CADM2		2.10E-17	-0.41	Lung
CADM2		4.30E-05	-0.16	Muscle - Skeletal
CADM2	rs11929525	1.50E-04	0.1	Nerve - Tibial
CADM2	rs12053927	1.10E-05	-0.65	Brain - Hippocampus
CADM2		1.10E-09	-0.25	Adipose - Subcutaneous
CADM2		3.40E-07	-0.23	Adipose - Visceral (Omentum)
CADM2		4.90E-07	-0.38	Heart - Left Ventricle
CADM2	rs12054328	1.10E-18	-0.41	Lung
CADM2	rs12163548	2.10E-08	1.1	Heart - Left Ventricle
CADM2-AS1	rs12397645	6.40E-05	-0.28	Nerve - Tibial
CADM2-AS1		4.30E-07	0.61	Brain - Caudate (basal ganglia)
CADM2-AS1		2.00E-10	0.73	Brain - Cerebellar Hemisphere
CADM2-AS1		1.40E-10	0.6	Brain - Cerebellum
CADM2-AS1		4.50E-06	0.56	Brain - Frontal Cortex (BA9)
CADM2-AS1		7.60E-06	0.59	Brain - Hippocampus
CADM2-AS1	rs12485709	3.10E-08	0.65	Brain - Nucleus accumbens (basal ganglia)
CADM2-AS1		5.20E-08	0.68	Brain - Caudate (basal ganglia)
CADM2-AS1		1.50E-09	0.73	Brain - Cerebellar Hemisphere
CADM2-AS1		4.60E-12	0.66	Brain - Cerebellum
CADM2-AS1		5.70E-06	0.56	Brain - Cortex
CADM2-AS1		4.60E-06	0.61	Brain - Frontal Cortex (BA9)
CADM2-AS1		1.10E-06	0.68	Brain - Hippocampus
CADM2-AS1		2.60E-08	0.67	Brain - Nucleus accumbens (basal ganglia)
CADM2-AS1	rs12486099	1.10E-04	0.26	Nerve - Tibial
CADM2	rs12486576	2.70E-05	0.29	Lung
CADM2-AS1		1.40E-07	0.65	Brain - Caudate (basal ganglia)
CADM2-AS1		5.10E-11	0.75	Brain - Cerebellar Hemisphere
CADM2-AS1		6.70E-12	0.63	Brain - Cerebellum
CADM2-AS1		3.50E-07	0.58	Brain - Cortex
CADM2-AS1		1.50E-07	0.65	Brain - Frontal Cortex (BA9)
CADM2-AS1		1.60E-06	0.67	Brain - Hippocampus
CADM2-AS1	rs12486982	1.80E-09	0.71	Brain - Nucleus accumbens (basal ganglia)
CADM2	rs12487446	2.20E-06	-0.32	Lung
CADM2	rs1248802	6.80E-05	-0.14	Muscle - Skeletal
CADM2	rs1248808	7.30E-05	-0.14	Muscle - Skeletal
CADM2	rs1248809	5.20E-05	-0.15	Muscle - Skeletal
CADM2	rs1248819	6.00E-05	-0.15	Muscle - Skeletal
CADM2	rs1248820	5.10E-05	-0.15	Muscle - Skeletal
CADM2	rs1248825	3.00E-06	0.18	Muscle - Skeletal

CADM2	rs1248834	5.30E-05	0.41	Heart - Left Ventricle
CADM2	rs1248836	3.10E-05	0.42	Heart - Left Ventricle
CADM2	rs1248850	9.20E-07	-0.57	Brain - Spinal cord (cervical c-1)
CADM2		4.90E-05	0.17	Adipose - Subcutaneous
CADM2	rs1248857	9.80E-05	0.15	Muscle - Skeletal
CADM2	rs1248858	1.80E-05	0.17	Muscle - Skeletal
CADM2	rs1248859	1.30E-05	0.18	Muscle - Skeletal
CADM2	rs1248860	3.80E-05	0.17	Adipose - Subcutaneous
CADM2	rs1248860	1.40E-05	0.16	Muscle - Skeletal
CADM2		7.10E-05	0.18	Adipose - Subcutaneous
CADM2	rs1248867	2.40E-07	0.21	Muscle - Skeletal
CADM2-AS1		1.80E-06	0.59	Brain - Caudate (basal ganglia)
CADM2-AS1		2.50E-10	0.74	Brain - Cerebellar Hemisphere
CADM2-AS1		1.80E-12	0.65	Brain - Cerebellum
CADM2-AS1		1.50E-06	0.56	Brain - Cortex
CADM2-AS1		5.40E-07	0.64	Brain - Frontal Cortex (BA9)
CADM2-AS1		1.90E-06	0.66	Brain - Hippocampus
CADM2-AS1		1.30E-08	0.67	Brain - Nucleus accumbens (basal ganglia)
CADM2-AS1	rs12488773	1.50E-05	0.57	Brain - Putamen (basal ganglia)
CADM2-AS1		8.00E-07	0.63	Brain - Caudate (basal ganglia)
CADM2-AS1		4.50E-07	0.63	Brain - Cerebellar Hemisphere
CADM2-AS1		1.60E-09	0.62	Brain - Cerebellum
CADM2-AS1	rs12489145	3.60E-05	0.29	Nerve - Tibial
CADM2	rs12489307	8.10E-05	0.27	Lung
CADM2	rs12491020	2.00E-05	-0.21	Lung
CADM2-AS1		7.00E-07	0.62	Brain - Caudate (basal ganglia)
CADM2-AS1		9.30E-08	0.68	Brain - Cerebellar Hemisphere
CADM2-AS1		2.30E-08	0.56	Brain - Cerebellum
CADM2-AS1		7.80E-07	0.69	Brain - Frontal Cortex (BA9)
CADM2-AS1	rs12491181	8.50E-06	0.59	Brain - Nucleus accumbens (basal ganglia)
CADM2-AS1		7.80E-07	0.59	Brain - Caudate (basal ganglia)
CADM2-AS1		1.00E-10	0.72	Brain - Cerebellar Hemisphere
CADM2-AS1		1.30E-11	0.62	Brain - Cerebellum
CADM2-AS1		1.10E-06	0.58	Brain - Frontal Cortex (BA9)
CADM2-AS1		8.00E-06	0.59	Brain - Hippocampus
CADM2-AS1	rs12491363	3.00E-08	0.64	Brain - Nucleus accumbens (basal ganglia)
CADM2		1.90E-09	-0.25	Adipose - Subcutaneous
CADM2		9.50E-08	-0.24	Adipose - Visceral (Omentum)
CADM2		1.50E-07	-0.4	Heart - Left Ventricle
CADM2	rs12491722	7.70E-20	-0.41	Lung
CADM2-AS1		7.10E-07	0.62	Brain - Caudate (basal ganglia)
CADM2-AS1		3.30E-10	0.74	Brain - Cerebellar Hemisphere
CADM2-AS1		1.80E-12	0.65	Brain - Cerebellum
CADM2-AS1		1.60E-06	0.56	Brain - Cortex
CADM2-AS1		5.40E-07	0.64	Brain - Frontal Cortex (BA9)
CADM2-AS1		1.60E-06	0.67	Brain - Hippocampus
CADM2-AS1		9.70E-09	0.68	Brain - Nucleus accumbens (basal ganglia)
CADM2-AS1	rs12491891	1.80E-05	0.57	Brain - Putamen (basal ganglia)

CADM2-AS1		1.10E-06	0.59	Brain - Caudate (basal ganglia)
CADM2-AS1		6.00E-11	0.73	Brain - Cerebellar Hemisphere
CADM2-AS1		1.10E-11	0.62	Brain - Cerebellum
CADM2-AS1		1.50E-06	0.57	Brain - Frontal Cortex (BA9)
CADM2-AS1		4.90E-06	0.6	Brain - Hippocampus
CADM2-AS1		2.80E-08	0.64	Brain - Nucleus accumbens (basal ganglia)
CADM2-AS1	rs12492459	7.70E-05	0.26	Nerve - Tibial
CADM2		2.00E-09	-0.25	Adipose - Subcutaneous
CADM2		4.30E-07	-0.23	Adipose - Visceral (Omentum)
CADM2		6.60E-07	-0.38	Heart - Left Ventricle
CADM2	rs12492753	1.50E-17	-0.4	Lung
CADM2-AS1		8.90E-07	0.59	Brain - Caudate (basal ganglia)
CADM2-AS1		9.10E-10	0.69	Brain - Cerebellar Hemisphere
CADM2-AS1		2.10E-10	0.59	Brain - Cerebellum
CADM2-AS1		1.70E-05	0.52	Brain - Frontal Cortex (BA9)
CADM2-AS1		2.40E-05	0.55	Brain - Hippocampus
CADM2-AS1	rs12493341	1.00E-06	0.57	Brain - Nucleus accumbens (basal ganglia)
CADM2		3.50E-08	0.26	Adipose - Subcutaneous
CADM2		1.80E-05	0.22	Adipose - Visceral (Omentum)
CADM2	rs12493461	8.50E-07	0.27	Lung
CADM2	rs12493563	9.90E-06	-0.22	Lung
CADM2-AS1	rs12493563	7.30E-05	0.27	Nerve - Tibial
CADM2		7.90E-07	-0.23	Lung
CADM2-AS1	rs12493621	3.50E-05	0.27	Nerve - Tibial
CADM2-AS1		2.40E-06	0.58	Brain - Caudate (basal ganglia)
CADM2-AS1		4.90E-08	0.64	Brain - Cerebellar Hemisphere
CADM2-AS1		1.00E-09	0.55	Brain - Cerebellum
CADM2-AS1		5.80E-06	0.51	Brain - Cortex
CADM2-AS1		3.50E-06	0.62	Brain - Frontal Cortex (BA9)
CADM2-AS1		1.30E-05	0.59	Brain - Hippocampus
CADM2-AS1	rs12493881	1.90E-06	0.59	Brain - Nucleus accumbens (basal ganglia)
CADM2	rs12494141	2.60E-08	1	Heart - Left Ventricle
CADM2-AS1	rs12494196	3.10E-05	0.47	Brain - Nucleus accumbens (basal ganglia)
CADM2		8.00E-05	0.17	Adipose - Subcutaneous
CADM2	rs12494242	7.90E-05	0.2	Lung
CADM2		1.40E-11	-0.28	Adipose - Subcutaneous
CADM2		6.30E-08	-0.24	Adipose - Visceral (Omentum)
CADM2		6.30E-07	-0.39	Heart - Left Ventricle
CADM2		2.90E-19	-0.42	Lung
CADM2	rs12494446	1.50E-04	0.1	Nerve - Tibial
CADM2-AS1		1.00E-05	0.57	Brain - Caudate (basal ganglia)
CADM2-AS1		1.20E-08	0.66	Brain - Cerebellar Hemisphere
CADM2-AS1		7.60E-08	0.5	Brain - Cerebellum
CADM2-AS1		4.70E-06	0.51	Brain - Cortex
CADM2-AS1		1.30E-06	0.59	Brain - Frontal Cortex (BA9)
CADM2-AS1	rs12494511	6.80E-07	0.58	Brain - Nucleus accumbens (basal ganglia)
CADM2-AS1		4.20E-06	0.62	Brain - Caudate (basal ganglia)
CADM2-AS1	rs12494658	4.40E-07	0.56	Brain - Cerebellum

CADM2	rs12495099	7.00E-06	0.74	Heart - Left Ventricle
CADM2-AS1		2.40E-06	0.59	Brain - Caudate (basal ganglia)
CADM2-AS1		4.60E-08	0.65	Brain - Cerebellar Hemisphere
CADM2-AS1		1.00E-09	0.55	Brain - Cerebellum
CADM2-AS1		5.80E-06	0.51	Brain - Cortex
CADM2-AS1		3.90E-06	0.62	Brain - Frontal Cortex (BA9)
CADM2-AS1		1.60E-05	0.59	Brain - Hippocampus
CADM2-AS1	rs12495144	2.20E-06	0.59	Brain - Nucleus accumbens (basal ganglia)
CADM2-AS1		1.50E-07	0.62	Brain - Caudate (basal ganglia)
CADM2-AS1		2.60E-06	0.55	Brain - Cerebellar Hemisphere
CADM2-AS1		5.20E-08	0.53	Brain - Cerebellum
CADM2-AS1	rs12495178	2.60E-05	0.49	Brain - Nucleus accumbens (basal ganglia)
CADM2		1.90E-08	-0.24	Adipose - Subcutaneous
CADM2		1.20E-08	-0.27	Adipose - Visceral (Omentum)
CADM2		3.30E-07	-0.39	Heart - Left Ventricle
CADM2	rs12495758	1.50E-18	-0.41	Lung
CADM2	rs12496044	2.60E-08	1	Heart - Left Ventricle
CADM2	rs12497566	2.60E-08	1	Heart - Left Ventricle
CADM2		2.10E-07	0.24	Adipose - Subcutaneous
CADM2		9.70E-05	0.2	Adipose - Visceral (Omentum)
CADM2		4.20E-05	0.22	Breast - Mammary Tissue
CADM2	rs12498010	2.10E-06	0.26	Lung
CADM2	rs12498070	6.50E-05	0.27	Lung
CADM2		1.70E-08	-0.23	Adipose - Subcutaneous
CADM2		4.10E-08	-0.26	Adipose - Visceral (Omentum)
CADM2		5.20E-07	-0.38	Heart - Left Ventricle
CADM2	rs12629607	6.90E-20	-0.41	Lung
CADM2	rs12629860	5.60E-05	0.2	Brain - Cerebellum
CADM2		5.60E-06	-0.48	Heart - Left Ventricle
CADM2	rs12631046	1.80E-06	-0.31	Lung
CADM2		4.20E-06	-0.49	Heart - Left Ventricle
CADM2	rs12631345	6.50E-06	-0.3	Lung
CADM2-AS1		6.70E-07	0.63	Brain - Caudate (basal ganglia)
CADM2-AS1		1.20E-06	0.66	Brain - Cerebellar Hemisphere
CADM2-AS1		6.00E-07	0.51	Brain - Cerebellum
CADM2-AS1		1.20E-06	0.69	Brain - Frontal Cortex (BA9)
CADM2-AS1	rs12631402	9.20E-06	0.6	Brain - Nucleus accumbens (basal ganglia)
CADM2-AS1		2.00E-07	0.59	Brain - Caudate (basal ganglia)
CADM2-AS1		4.60E-05	0.46	Brain - Cerebellar Hemisphere
CADM2-AS1	rs12631564	2.40E-07	0.48	Brain - Cerebellum
CADM2		6.90E-06	-0.19	Adipose - Subcutaneous
CADM2		3.30E-09	-0.27	Lung
CADM2-AS1	rs12632140	1.40E-04	0.25	Nerve - Tibial
CADM2		3.00E-06	-0.2	Adipose - Subcutaneous
CADM2	rs12632674	3.30E-08	-0.25	Lung
CADM2-AS1		1.90E-07	0.62	Brain - Caudate (basal ganglia)
CADM2-AS1		2.90E-09	0.67	Brain - Cerebellar Hemisphere
CADM2-AS1		3.40E-10	0.58	Brain - Cerebellum
CADM2-AS1	rs12633520	2.10E-05	0.52	Brain - Frontal Cortex (BA9)

CADM2-AS1		1.40E-05	0.57	Brain - Hippocampus
CADM2-AS1		1.60E-06	0.56	Brain - Nucleus accumbens (basal ganglia)
CADM2		1.00E-09	-0.25	Adipose - Subcutaneous
CADM2		1.20E-07	-0.24	Adipose - Visceral (Omentum)
CADM2		2.00E-07	-0.39	Heart - Left Ventricle
CADM2	rs12633657	1.10E-19	-0.41	Lung
CADM2		2.20E-08	0.26	Adipose - Subcutaneous
CADM2		1.90E-05	0.22	Adipose - Visceral (Omentum)
CADM2	rs12633762	1.20E-06	0.27	Lung
CADM2-AS1		9.40E-08	0.67	Brain - Caudate (basal ganglia)
CADM2-AS1		1.10E-08	0.72	Brain - Cerebellar Hemisphere
CADM2-AS1		2.50E-07	0.54	Brain - Cerebellum
CADM2-AS1		1.70E-07	0.73	Brain - Frontal Cortex (BA9)
CADM2-AS1	rs12634260	1.20E-05	0.59	Brain - Nucleus accumbens (basal ganglia)
CADM2		7.20E-05	0.17	Adipose - Subcutaneous
CADM2		6.90E-06	0.21	Adipose - Visceral (Omentum)
CADM2	rs12634949	1.20E-06	0.24	Lung
CADM2		4.30E-05	0.21	Adipose - Subcutaneous
CADM2	rs12635683	2.70E-05	0.24	Lung
CADM2-AS1		1.00E-06	0.6	Brain - Caudate (basal ganglia)
CADM2-AS1		1.30E-07	0.62	Brain - Cerebellar Hemisphere
CADM2-AS1		1.20E-08	0.53	Brain - Cerebellum
CADM2-AS1		4.80E-06	0.49	Brain - Cortex
CADM2-AS1		3.20E-06	0.56	Brain - Frontal Cortex (BA9)
CADM2-AS1	rs12636273	1.30E-06	0.55	Brain - Nucleus accumbens (basal ganglia)
CADM2		4.10E-06	-0.2	Adipose - Subcutaneous
CADM2	rs12636904	5.20E-10	-0.28	Lung
CADM2		2.60E-06	-0.2	Adipose - Subcutaneous
CADM2		6.80E-05	-0.18	Adipose - Visceral (Omentum)
CADM2	rs12637027	9.60E-11	-0.32	Lung
CADM2		1.90E-10	-0.27	Adipose - Subcutaneous
CADM2		5.60E-08	-0.25	Adipose - Visceral (Omentum)
CADM2		1.30E-06	-0.39	Heart - Left Ventricle
CADM2	rs12637461	2.10E-17	-0.4	Lung
CADM2		5.20E-09	-0.24	Adipose - Subcutaneous
CADM2		3.00E-07	-0.23	Adipose - Visceral (Omentum)
CADM2		7.70E-08	-0.41	Heart - Left Ventricle
CADM2	rs12637791	2.30E-18	-0.4	Lung
CADM2		6.40E-11	-0.27	Adipose - Subcutaneous
CADM2		3.70E-08	-0.25	Adipose - Visceral (Omentum)
CADM2		1.50E-07	-0.42	Heart - Left Ventricle
CADM2	rs12637798	9.00E-17	-0.4	Lung
CADM2		2.40E-11	-0.28	Adipose - Subcutaneous
CADM2		4.50E-08	-0.24	Adipose - Visceral (Omentum)
CADM2		1.40E-06	-0.38	Heart - Left Ventricle
CADM2	rs12638040	3.80E-18	-0.4	Lung
CADM2		2.10E-10	-0.27	Adipose - Subcutaneous
CADM2		6.50E-08	-0.25	Adipose - Visceral (Omentum)
CADM2		4.20E-07	-0.42	Heart - Left Ventricle
CADM2		2.40E-17	-0.4	Lung
CADM2	rs12638482	1.70E-04	-0.14	Muscle - Skeletal

CADM2		2.90E-09	-0.24	Adipose - Subcutaneous
CADM2		1.20E-07	-0.24	Adipose - Visceral (Omentum)
CADM2		1.60E-07	-0.4	Heart - Left Ventricle
CADM2	rs12638798	4.20E-19	-0.41	Lung
CADM2		1.30E-05	-0.46	Heart - Left Ventricle
CADM2	rs12639001	1.40E-06	-0.31	Lung
CADM2		1.40E-11	-0.28	Adipose - Subcutaneous
CADM2		5.60E-08	-0.24	Adipose - Visceral (Omentum)
CADM2		6.80E-07	-0.39	Heart - Left Ventricle
CADM2		2.90E-19	-0.42	Lung
CADM2	rs12639564	1.30E-04	0.1	Nerve - Tibial
CADM2		8.30E-05	0.18	Adipose - Subcutaneous
CADM2		1.50E-06	0.61	Brain - Spinal cord (cervical c-1)
CADM2	rs12714613	3.60E-06	0.19	Muscle - Skeletal
CADM2		6.10E-05	0.19	Adipose - Subcutaneous
CADM2		9.40E-06	0.28	Brain - Caudate (basal ganglia)
CADM2		2.90E-06	0.68	Brain - Spinal cord (cervical c-1)
CADM2	rs12714616	8.70E-05	0.16	Muscle - Skeletal
CADM2		3.50E-05	0.19	Adipose - Subcutaneous
CADM2		1.00E-05	0.28	Brain - Caudate (basal ganglia)
CADM2		2.90E-06	0.68	Brain - Spinal cord (cervical c-1)
CADM2	rs12714617	2.30E-05	0.17	Muscle - Skeletal
CADM2	rs12714625	5.80E-05	0.17	Adipose - Subcutaneous
CADM2		5.90E-08	0.28	Adipose - Subcutaneous
CADM2		6.80E-06	0.25	Adipose - Visceral (Omentum)
CADM2	rs12714629	4.70E-09	0.34	Lung
CADM2	rs12714630	4.20E-05	-0.27	Lung
CADM2	rs12714631	2.40E-05	-0.28	Lung
CADM2-AS1		3.80E-05	0.5	Brain - Cerebellar Hemisphere
CADM2-AS1	rs12714632	3.10E-05	0.48	Brain - Nucleus accumbens (basal ganglia)
CADM2		5.20E-05	0.23	Adipose - Visceral (Omentum)
CADM2	rs12714636	7.10E-06	0.27	Lung
CADM2-AS1		1.30E-05	0.57	Brain - Caudate (basal ganglia)
CADM2-AS1		6.20E-08	0.66	Brain - Cerebellar Hemisphere
CADM2-AS1		1.50E-07	0.51	Brain - Cerebellum
CADM2-AS1		3.10E-05	0.49	Brain - Cortex
CADM2-AS1		1.30E-06	0.61	Brain - Frontal Cortex (BA9)
CADM2-AS1	rs12714642	4.60E-06	0.55	Brain - Nucleus accumbens (basal ganglia)
CADM2-AS1	rs12714646	1.40E-05	-0.42	Nerve - Tibial
CADM2-AS1	rs12714648	1.30E-04	-0.36	Nerve - Tibial
CADM2	rs13059287	1.00E-04	0.15	Adipose - Subcutaneous
CADM2-AS1		6.10E-07	0.62	Brain - Caudate (basal ganglia)
CADM2-AS1		2.50E-10	0.74	Brain - Cerebellar Hemisphere
CADM2-AS1		1.80E-12	0.65	Brain - Cerebellum
CADM2-AS1		1.50E-06	0.56	Brain - Cortex
CADM2-AS1		5.40E-07	0.64	Brain - Frontal Cortex (BA9)
CADM2-AS1		1.60E-06	0.67	Brain - Hippocampus
CADM2-AS1		9.70E-09	0.68	Brain - Nucleus accumbens (basal ganglia)
CADM2-AS1	rs13059349	1.80E-05	0.57	Brain - Putamen (basal ganglia)
CADM2	rs13059866	1.20E-04	0.26	Adipose - Subcutaneous
CADM2-AS1	rs13060858	2.50E-05	0.51	Brain - Cerebellar Hemisphere

CADM2-AS1		1.60E-06	0.6	Brain - Caudate (basal ganglia)
CADM2-AS1		2.50E-10	0.74	Brain - Cerebellar Hemisphere
CADM2-AS1		1.20E-12	0.66	Brain - Cerebellum
CADM2-AS1		3.20E-06	0.55	Brain - Cortex
CADM2-AS1		5.40E-07	0.64	Brain - Frontal Cortex (BA9)
CADM2-AS1		1.90E-06	0.66	Brain - Hippocampus
CADM2-AS1		9.70E-09	0.68	Brain - Nucleus accumbens (basal ganglia)
CADM2-AS1	rs13061174	1.80E-05	0.57	Brain - Putamen (basal ganglia)
CADM2-AS1	rs13061466	5.10E-06	-0.44	Nerve - Tibial
CADM2	rs13061527	1.00E-05	0.15	Muscle - Skeletal
CADM2		6.20E-05	0.22	Adipose - Subcutaneous
CADM2	rs13061681	2.30E-07	0.32	Lung
CADM2		2.10E-05	0.19	Adipose - Subcutaneous
CADM2		6.10E-06	0.28	Brain - Caudate (basal ganglia)
CADM2		2.90E-08	0.72	Brain - Spinal cord (cervical c-1)
CADM2	rs13062439	5.60E-06	0.18	Muscle - Skeletal
CADM2		9.00E-05	0.15	Adipose - Subcutaneous
CADM2	rs13063356	8.50E-05	0.14	Muscle - Skeletal
CADM2-AS1	rs13064757	2.20E-05	-0.43	Nerve - Tibial
CADM2	rs13064817	2.40E-05	0.15	Muscle - Skeletal
CADM2-AS1		2.60E-07	0.64	Brain - Caudate (basal ganglia)
CADM2-AS1		5.70E-10	0.72	Brain - Cerebellar Hemisphere
CADM2-AS1		1.50E-12	0.66	Brain - Cerebellum
CADM2-AS1		1.20E-06	0.57	Brain - Cortex
CADM2-AS1		1.00E-06	0.62	Brain - Frontal Cortex (BA9)
CADM2-AS1		1.60E-06	0.67	Brain - Hippocampus
CADM2-AS1		2.50E-08	0.66	Brain - Nucleus accumbens (basal ganglia)
CADM2-AS1	rs13065014	3.10E-05	0.56	Brain - Putamen (basal ganglia)
CADM2		5.00E-05	0.18	Adipose - Subcutaneous
CADM2		2.60E-05	0.26	Brain - Caudate (basal ganglia)
CADM2		7.80E-07	0.69	Brain - Spinal cord (cervical c-1)
CADM2	rs13066623	1.20E-05	0.17	Muscle - Skeletal
CADM2-AS1		3.00E-05	0.63	Brain - Anterior cingulate cortex (BA24)
CADM2-AS1		2.60E-05	0.54	Brain - Caudate (basal ganglia)
CADM2-AS1		2.30E-06	0.57	Brain - Cerebellar Hemisphere
CADM2-AS1		6.60E-09	0.53	Brain - Cerebellum
CADM2-AS1		3.90E-05	0.45	Brain - Cortex
CADM2-AS1		2.20E-05	0.52	Brain - Frontal Cortex (BA9)
CADM2-AS1	rs13068011	1.70E-06	0.56	Brain - Nucleus accumbens (basal ganglia)
CADM2-AS1	rs13068138	7.40E-05	-0.33	Nerve - Tibial
CADM2-AS1		8.40E-06	0.57	Brain - Caudate (basal ganglia)
CADM2-AS1		1.00E-08	0.66	Brain - Cerebellar Hemisphere
CADM2-AS1		6.80E-08	0.5	Brain - Cerebellum
CADM2-AS1		6.10E-06	0.5	Brain - Cortex
CADM2-AS1		9.20E-07	0.6	Brain - Frontal Cortex (BA9)
CADM2-AS1	rs13068185	6.80E-07	0.58	Brain - Nucleus accumbens (basal ganglia)
CADM2		1.30E-07	0.27	Adipose - Subcutaneous
CADM2		1.90E-05	0.24	Adipose - Visceral (Omentum)
CADM2	rs13068434	7.00E-09	0.34	Lung

CADM2		4.50E-05	0.19	Adipose - Subcutaneous
CADM2		4.50E-07	0.64	Brain - Spinal cord (cervical c-1)
CADM2	rs13068788	4.00E-05	0.17	Muscle - Skeletal
CADM2		1.60E-05	0.2	Adipose - Subcutaneous
CADM2	rs13069094	1.40E-06	0.63	Brain - Spinal cord (cervical c-1)
CADM2		3.20E-08	0.27	Adipose - Subcutaneous
CADM2	rs13070166	6.80E-07	0.27	Lung
CADM2	rs13070794	5.70E-07	0.64	Brain - Spinal cord (cervical c-1)
CADM2		3.10E-05	0.19	Adipose - Subcutaneous
CADM2		2.50E-06	0.57	Brain - Spinal cord (cervical c-1)
CADM2	rs13071144	2.60E-06	0.18	Muscle - Skeletal
CADM2-AS1		3.30E-08	0.66	Brain - Caudate (basal ganglia)
CADM2-AS1		8.20E-08	0.65	Brain - Cerebellar Hemisphere
CADM2-AS1		4.40E-09	0.58	Brain - Cerebellum
CADM2-AS1	rs13071488	7.30E-07	0.59	Brain - Nucleus accumbens (basal ganglia)
CADM2-AS1	rs13071604	2.80E-05	0.41	Brain - Cerebellum
CADM2		1.00E-04	0.16	Adipose - Subcutaneous
CADM2	rs13072305	3.30E-06	0.17	Muscle - Skeletal
CADM2-AS1	rs13072339	5.50E-05	0.38	Brain - Cerebellum
CADM2-AS1		2.00E-07	0.65	Brain - Caudate (basal ganglia)
CADM2-AS1		2.10E-10	0.75	Brain - Cerebellar Hemisphere
CADM2-AS1		2.10E-11	0.64	Brain - Cerebellum
CADM2-AS1		5.20E-06	0.56	Brain - Cortex
CADM2-AS1		1.40E-06	0.63	Brain - Frontal Cortex (BA9)
CADM2-AS1		1.90E-06	0.66	Brain - Hippocampus
CADM2-AS1		1.20E-08	0.69	Brain - Nucleus accumbens (basal ganglia)
CADM2-AS1	rs13073604	3.50E-05	0.56	Brain - Putamen (basal ganglia)
CADM2		8.80E-06	0.32	Brain - Hippocampus
CADM2	rs13074075	9.00E-05	0.14	Muscle - Skeletal
CADM2		1.20E-04	0.18	Adipose - Subcutaneous
CADM2		1.70E-06	0.64	Brain - Spinal cord (cervical c-1)
CADM2	rs13074892	8.30E-05	0.16	Muscle - Skeletal
CADM2		1.50E-05	-0.46	Heart - Left Ventricle
CADM2	rs13075857	1.60E-06	-0.32	Lung
CADM2		2.00E-05	0.62	Brain - Spinal cord (cervical c-1)
CADM2	rs13075873	1.30E-05	0.16	Muscle - Skeletal
CADM2		4.50E-08	0.25	Adipose - Subcutaneous
CADM2		4.10E-05	0.21	Adipose - Visceral (Omentum)
CADM2	rs13076632	2.00E-06	0.26	Lung
CADM2		1.40E-07	0.27	Adipose - Subcutaneous
CADM2		1.80E-05	0.24	Adipose - Visceral (Omentum)
CADM2	rs13076830	7.00E-09	0.34	Lung
CADM2-AS1		3.80E-05	0.5	Brain - Cerebellar Hemisphere
CADM2-AS1	rs13076876	3.30E-05	0.48	Brain - Nucleus accumbens (basal ganglia)
CADM2		5.90E-08	0.28	Adipose - Subcutaneous
CADM2		6.80E-06	0.25	Adipose - Visceral (Omentum)
CADM2	rs13077660	4.70E-09	0.34	Lung
CADM2		2.70E-05	0.19	Adipose - Subcutaneous
CADM2		2.50E-06	0.57	Brain - Spinal cord (cervical c-1)
CADM2	rs13077814	2.80E-06	0.18	Muscle - Skeletal
CADM2-AS1	rs13077826	3.80E-05	0.5	Brain - Cerebellar Hemisphere

CADM2-AS1		3.10E-05	0.48	Brain - Nucleus accumbens (basal ganglia)
CADM2		3.30E-05	0.32	Brain - Hippocampus
CADM2	rs13078384	1.40E-07	0.84	Brain - Spinal cord (cervical c-1)
CADM2		3.30E-05	0.16	Adipose - Subcutaneous
CADM2	rs13078494	1.40E-06	0.17	Muscle - Skeletal
CADM2		3.80E-05	0.19	Adipose - Subcutaneous
CADM2		4.50E-07	0.64	Brain - Spinal cord (cervical c-1)
CADM2	rs13078624	9.00E-05	0.16	Muscle - Skeletal
CADM2-AS1		1.60E-07	0.62	Brain - Caudate (basal ganglia)
CADM2-AS1		6.50E-09	0.66	Brain - Cerebellar Hemisphere
CADM2-AS1		2.90E-10	0.58	Brain - Cerebellum
CADM2-AS1		1.70E-05	0.52	Brain - Frontal Cortex (BA9)
CADM2-AS1		1.10E-05	0.56	Brain - Hippocampus
CADM2-AS1	rs13079014	3.50E-06	0.53	Brain - Nucleus accumbens (basal ganglia)
CADM2-AS1		3.80E-05	0.5	Brain - Cerebellar Hemisphere
CADM2-AS1	rs13080469	3.10E-05	0.48	Brain - Nucleus accumbens (basal ganglia)
CADM2		1.40E-08	0.27	Adipose - Subcutaneous
CADM2		1.10E-05	0.23	Adipose - Visceral (Omentum)
CADM2	rs13081230	9.80E-07	0.27	Lung
CADM2		1.70E-05	0.33	Adipose - Subcutaneous
CADM2	rs13081724	5.50E-05	0.36	Lung
CADM2		3.70E-05	0.19	Adipose - Subcutaneous
CADM2	rs13081745	1.20E-05	0.17	Muscle - Skeletal
CADM2-AS1		4.70E-07	0.6	Brain - Caudate (basal ganglia)
CADM2-AS1		7.10E-10	0.69	Brain - Cerebellar Hemisphere
CADM2-AS1		2.90E-10	0.58	Brain - Cerebellum
CADM2-AS1		5.80E-06	0.55	Brain - Frontal Cortex (BA9)
CADM2-AS1		3.00E-05	0.54	Brain - Hippocampus
CADM2-AS1	rs13082138	8.10E-07	0.58	Brain - Nucleus accumbens (basal ganglia)
CADM2		4.30E-05	0.21	Adipose - Subcutaneous
CADM2		3.30E-05	0.25	Lung
CADM2-AS1	rs13082188	3.40E-05	-0.31	Nerve - Tibial
CADM2	rs13082397	5.70E-07	0.64	Brain - Spinal cord (cervical c-1)
CADM2		1.20E-04	0.17	Adipose - Subcutaneous
CADM2		2.30E-05	0.27	Brain - Caudate (basal ganglia)
CADM2		7.80E-07	0.69	Brain - Spinal cord (cervical c-1)
CADM2	rs13082518	3.20E-05	0.17	Muscle - Skeletal
CADM2-AS1		1.40E-07	0.65	Brain - Caudate (basal ganglia)
CADM2-AS1		5.10E-11	0.75	Brain - Cerebellar Hemisphere
CADM2-AS1		4.80E-12	0.63	Brain - Cerebellum
CADM2-AS1		3.50E-07	0.58	Brain - Cortex
CADM2-AS1		1.50E-07	0.65	Brain - Frontal Cortex (BA9)
CADM2-AS1		1.10E-06	0.67	Brain - Hippocampus
CADM2-AS1		1.70E-09	0.71	Brain - Nucleus accumbens (basal ganglia)
CADM2-AS1	rs13083257	1.10E-04	0.25	Nerve - Tibial
CADM2		2.70E-05	0.19	Adipose - Subcutaneous
CADM2		1.70E-06	0.59	Brain - Spinal cord (cervical c-1)
CADM2	rs13083529	1.50E-06	0.19	Muscle - Skeletal
CADM2		7.70E-05	0.23	Adipose - Visceral (Omentum)
CADM2	rs13083873	2.60E-06	0.28	Lung
CADM2	rs13084351	1.00E-04	0.18	Adipose - Subcutaneous

CADM2		3.70E-07	0.65	Brain - Spinal cord (cervical c-1)
CADM2		1.80E-05	0.18	Muscle - Skeletal
CADM2		5.90E-08	0.28	Adipose - Subcutaneous
CADM2		6.80E-06	0.25	Adipose - Visceral (Omentum)
CADM2	rs13084531	4.70E-09	0.34	Lung
CADM2		4.10E-08	0.26	Adipose - Subcutaneous
CADM2		8.20E-05	0.2	Adipose - Visceral (Omentum)
CADM2	rs13085170	2.20E-06	0.27	Lung
CADM2-AS1		1.00E-05	0.57	Brain - Caudate (basal ganglia)
CADM2-AS1		1.20E-08	0.66	Brain - Cerebellar Hemisphere
CADM2-AS1		7.60E-08	0.5	Brain - Cerebellum
CADM2-AS1		4.70E-06	0.51	Brain - Cortex
CADM2-AS1		1.30E-06	0.59	Brain - Frontal Cortex (BA9)
CADM2-AS1	rs13085195	6.80E-07	0.58	Brain - Nucleus accumbens (basal ganglia)
CADM2		5.10E-05	0.19	Adipose - Subcutaneous
CADM2		6.30E-07	0.64	Brain - Spinal cord (cervical c-1)
CADM2	rs13085424	2.30E-05	0.17	Muscle - Skeletal
CADM2		5.10E-05	0.23	Adipose - Visceral (Omentum)
CADM2	rs13087042	3.90E-06	0.28	Lung
CADM2		3.60E-05	0.19	Adipose - Subcutaneous
CADM2		2.50E-06	0.57	Brain - Spinal cord (cervical c-1)
CADM2	rs13089401	2.90E-06	0.18	Muscle - Skeletal
CADM2		7.20E-05	0.18	Adipose - Subcutaneous
CADM2		2.30E-05	0.27	Brain - Caudate (basal ganglia)
CADM2		7.80E-07	0.69	Brain - Spinal cord (cervical c-1)
CADM2	rs13092125	1.90E-05	0.17	Muscle - Skeletal
CADM2-AS1		1.40E-06	0.59	Brain - Caudate (basal ganglia)
CADM2-AS1		9.00E-09	0.67	Brain - Cerebellar Hemisphere
CADM2-AS1		6.00E-11	0.62	Brain - Cerebellum
CADM2-AS1		2.20E-05	0.56	Brain - Hippocampus
CADM2-AS1		1.60E-06	0.56	Brain - Nucleus accumbens (basal ganglia)
CADM2-AS1	rs13092143	9.40E-05	0.26	Nerve - Tibial
CADM2		1.50E-07	0.27	Adipose - Subcutaneous
CADM2		1.60E-05	0.25	Adipose - Visceral (Omentum)
CADM2	rs13092437	1.10E-08	0.34	Lung
CADM2		5.00E-08	0.26	Adipose - Subcutaneous
CADM2		1.80E-05	0.22	Adipose - Visceral (Omentum)
CADM2	rs13092627	1.70E-06	0.27	Lung
CADM2-AS1		6.90E-07	0.61	Brain - Caudate (basal ganglia)
CADM2-AS1		1.70E-07	0.66	Brain - Cerebellar Hemisphere
CADM2-AS1		8.50E-09	0.56	Brain - Cerebellum
CADM2-AS1		3.50E-07	0.7	Brain - Frontal Cortex (BA9)
CADM2-AS1	rs13093396	9.50E-06	0.58	Brain - Nucleus accumbens (basal ganglia)
CADM2-AS1	rs13093477	3.90E-05	-0.3	Nerve - Tibial
CADM2		1.20E-05	0.2	Adipose - Subcutaneous
CADM2		6.10E-06	0.28	Brain - Caudate (basal ganglia)
CADM2		2.90E-08	0.72	Brain - Spinal cord (cervical c-1)
CADM2	rs13093660	2.20E-06	0.19	Muscle - Skeletal
CADM2-AS1	rs13093782	5.20E-05	-0.29	Nerve - Tibial
CADM2		5.70E-05	0.17	Adipose - Subcutaneous
CADM2	rs13094105	1.10E-04	0.2	Lung

CADM2-AS1	rs13095102	1.30E-05	-0.41	Nerve - Tibial
CADM2		2.90E-07	0.67	Brain - Spinal cord (cervical c-1)
CADM2	rs13095335	3.70E-05	0.17	Muscle - Skeletal
CADM2		8.80E-06	0.32	Brain - Hippocampus
CADM2	rs13095644	6.10E-05	0.14	Muscle - Skeletal
CADM2		4.00E-07	0.67	Brain - Spinal cord (cervical c-1)
CADM2	rs13095729	5.10E-05	0.17	Muscle - Skeletal
CADM2		5.60E-05	0.24	Adipose - Visceral (Omentum)
CADM2	rs13096280	3.30E-06	0.28	Lung
CADM2		3.40E-05	0.18	Adipose - Subcutaneous
CADM2		2.60E-05	0.26	Brain - Caudate (basal ganglia)
CADM2		7.80E-07	0.69	Brain - Spinal cord (cervical c-1)
CADM2	rs13096651	1.40E-05	0.17	Muscle - Skeletal
CADM2		6.10E-05	0.16	Adipose - Subcutaneous
CADM2	rs13097560	3.50E-05	0.15	Muscle - Skeletal
CADM2		7.00E-05	0.19	Adipose - Subcutaneous
CADM2		3.70E-07	0.65	Brain - Spinal cord (cervical c-1)
CADM2	rs13097857	1.70E-05	0.18	Muscle - Skeletal
CADM2		9.90E-05	0.22	Adipose - Visceral (Omentum)
CADM2	rs13097865	3.60E-06	0.28	Lung
CADM2		3.60E-05	0.19	Adipose - Subcutaneous
CADM2		2.50E-06	0.57	Brain - Spinal cord (cervical c-1)
CADM2	rs13098134	2.90E-06	0.18	Muscle - Skeletal
CADM2-AS1		7.30E-08	0.66	Brain - Caudate (basal ganglia)
CADM2-AS1		3.50E-08	0.65	Brain - Cerebellar Hemisphere
CADM2-AS1		2.40E-09	0.57	Brain - Cerebellum
CADM2-AS1		6.20E-06	0.6	Brain - Hippocampus
CADM2-AS1	rs13099482	4.60E-06	0.55	Brain - Nucleus accumbens (basal ganglia)
CADM2		1.90E-07	0.24	Adipose - Subcutaneous
CADM2		8.20E-05	0.2	Adipose - Visceral (Omentum)
CADM2		4.90E-05	0.21	Breast - Mammary Tissue
CADM2	rs13099750	2.10E-06	0.26	Lung
CADM2-AS1		1.90E-07	0.66	Brain - Caudate (basal ganglia)
CADM2-AS1		1.80E-07	0.69	Brain - Cerebellar Hemisphere
CADM2-AS1		1.90E-07	0.55	Brain - Cerebellum
CADM2-AS1		9.20E-07	0.71	Brain - Frontal Cortex (BA9)
CADM2-AS1	rs13100969	8.60E-06	0.6	Brain - Nucleus accumbens (basal ganglia)
CADM2	rs13101042	4.80E-06	0.17	Muscle - Skeletal
CADM2		1.10E-04	0.21	Adipose - Subcutaneous
CADM2		3.30E-05	0.24	Adipose - Visceral (Omentum)
CADM2	rs13101056	3.20E-06	0.28	Lung
CADM2	rs1314615	2.20E-06	0.17	Muscle - Skeletal
CADM2	rs1319451	1.80E-07	-0.34	Lung
CADM2		1.60E-06	0.22	Adipose - Subcutaneous
CADM2		4.40E-05	0.26	Brain - Caudate (basal ganglia)
CADM2		1.10E-08	0.73	Brain - Spinal cord (cervical c-1)
CADM2	rs13315526	9.30E-06	0.18	Muscle - Skeletal
CADM2-AS1	rs13323436	1.10E-04	-0.43	Nerve - Tibial
CADM2-AS1	rs13324340	1.00E-04	-0.36	Nerve - Tibial
CADM2-AS1	rs13325775	1.30E-05	-0.41	Nerve - Tibial
CADM2-AS1	rs13327074	1.00E-06	-0.47	Nerve - Tibial
CADM2	rs13353478	5.90E-08	0.28	Adipose - Subcutaneous

CADM2		6.80E-06	0.25	Adipose - Visceral (Omentum)
CADM2		4.70E-09	0.34	Lung
CADM2-AS1	rs13433984	1.30E-04	-0.36	Nerve - Tibial
CADM2-AS1	rs13434266	1.10E-04	-0.36	Nerve - Tibial
CADM2	rs1349602	9.20E-06	-0.66	Brain - Hippocampus
CADM2		1.30E-08	0.27	Adipose - Subcutaneous
CADM2		1.80E-05	0.22	Adipose - Visceral (Omentum)
CADM2	rs1368739	1.30E-06	0.27	Lung
CADM2		2.40E-08	0.26	Adipose - Subcutaneous
CADM2		6.20E-05	0.2	Adipose - Visceral (Omentum)
CADM2	rs1368740	2.40E-06	0.26	Lung
CADM2		3.10E-08	0.26	Adipose - Subcutaneous
CADM2		5.00E-05	0.21	Adipose - Visceral (Omentum)
CADM2	rs1368743	2.40E-06	0.27	Lung
CADM2		1.30E-05	-0.46	Heart - Left Ventricle
CADM2	rs1368744	1.40E-06	-0.31	Lung
CADM2	rs1368746	8.00E-06	-0.66	Testis
CADM2		2.00E-06	-0.21	Adipose - Subcutaneous
CADM2		4.10E-05	-0.19	Adipose - Visceral (Omentum)
CADM2	rs1368748	9.60E-08	-0.27	Lung
CADM2		6.30E-12	-0.29	Adipose - Subcutaneous
CADM2		1.30E-08	-0.25	Adipose - Visceral (Omentum)
CADM2		6.20E-07	-0.39	Heart - Left Ventricle
CADM2	rs1368750	7.30E-18	-0.4	Lung
CADM2	rs1375215	8.30E-05	-0.19	Lung
CADM2-AS1		2.00E-07	0.62	Brain - Caudate (basal ganglia)
CADM2-AS1		2.80E-06	0.54	Brain - Cerebellar Hemisphere
CADM2-AS1	rs1375217	1.10E-07	0.52	Brain - Cerebellum
CADM2-AS1		2.90E-05	0.53	Brain - Caudate (basal ganglia)
CADM2-AS1		6.50E-07	0.59	Brain - Cerebellar Hemisphere
CADM2-AS1		6.50E-08	0.52	Brain - Cerebellum
CADM2-AS1		2.10E-05	0.53	Brain - Frontal Cortex (BA9)
CADM2-AS1	rs1375218	2.00E-05	0.5	Brain - Nucleus accumbens (basal ganglia)
CADM2-AS1		1.90E-06	0.57	Brain - Cerebellar Hemisphere
CADM2-AS1		3.70E-08	0.54	Brain - Cerebellum
CADM2-AS1	rs1375219	2.00E-05	0.51	Brain - Nucleus accumbens (basal ganglia)
CADM2		8.10E-12	-0.29	Adipose - Subcutaneous
CADM2		1.80E-08	-0.25	Adipose - Visceral (Omentum)
CADM2		5.50E-07	-0.39	Heart - Left Ventricle
CADM2		2.50E-18	-0.41	Lung
CADM2	rs1375544	1.40E-04	0.1	Nerve - Tibial
CADM2		9.10E-12	-0.28	Adipose - Subcutaneous
CADM2		1.60E-08	-0.25	Adipose - Visceral (Omentum)
CADM2		7.60E-07	-0.39	Heart - Left Ventricle
CADM2		7.50E-19	-0.41	Lung
CADM2		1.40E-04	-0.14	Muscle - Skeletal
CADM2	rs1375545	1.60E-04	0.099	Nerve - Tibial
CADM2		5.60E-12	-0.29	Adipose - Subcutaneous
CADM2		1.50E-08	-0.25	Adipose - Visceral (Omentum)
CADM2		7.40E-07	-0.39	Heart - Left Ventricle
CADM2	rs1375546	1.80E-18	-0.41	Lung

CADM2		1.60E-04	0.099	Nerve - Tibial
CADM2		3.30E-08	0.29	Adipose - Subcutaneous
CADM2		2.50E-05	0.24	Adipose - Visceral (Omentum)
CADM2	rs1375547	2.70E-08	0.33	Lung
CADM2		4.80E-12	-0.29	Adipose - Subcutaneous
CADM2		2.40E-08	-0.25	Adipose - Visceral (Omentum)
CADM2		1.60E-06	-0.38	Heart - Left Ventricle
CADM2	rs1375549	1.40E-17	-0.4	Lung
CADM2		4.80E-12	-0.29	Adipose - Subcutaneous
CADM2		2.40E-08	-0.25	Adipose - Visceral (Omentum)
CADM2		1.60E-06	-0.38	Heart - Left Ventricle
CADM2	rs1375550	1.40E-17	-0.4	Lung
CADM2		7.50E-12	-0.28	Adipose - Subcutaneous
CADM2		1.10E-08	-0.25	Adipose - Visceral (Omentum)
CADM2		8.80E-07	-0.39	Heart - Left Ventricle
CADM2	rs1375551	1.20E-17	-0.4	Lung
CADM2		5.90E-08	0.28	Adipose - Subcutaneous
CADM2		6.80E-06	0.25	Adipose - Visceral (Omentum)
CADM2	rs1375552	4.70E-09	0.34	Lung
CADM2		5.90E-08	0.28	Adipose - Subcutaneous
CADM2		6.80E-06	0.25	Adipose - Visceral (Omentum)
CADM2	rs1375553	4.70E-09	0.34	Lung
CADM2		6.70E-12	-0.29	Adipose - Subcutaneous
CADM2		1.60E-08	-0.25	Adipose - Visceral (Omentum)
CADM2		8.20E-07	-0.39	Heart - Left Ventricle
CADM2		2.10E-18	-0.41	Lung
CADM2	rs1375554	1.60E-04	0.099	Nerve - Tibial
CADM2		1.00E-11	-0.28	Adipose - Subcutaneous
CADM2		1.60E-08	-0.25	Adipose - Visceral (Omentum)
CADM2		7.80E-07	-0.39	Heart - Left Ventricle
CADM2	rs1375555	6.40E-18	-0.4	Lung
CADM2	rs1375556	8.30E-08	-0.35	Lung
CADM2		3.10E-11	-0.28	Adipose - Subcutaneous
CADM2		4.30E-08	-0.24	Adipose - Visceral (Omentum)
CADM2		2.10E-06	-0.37	Heart - Left Ventricle
CADM2	rs1375557	3.70E-18	-0.4	Lung
CADM2		1.50E-11	-0.28	Adipose - Subcutaneous
CADM2		4.60E-08	-0.24	Adipose - Visceral (Omentum)
CADM2		1.30E-06	-0.38	Heart - Left Ventricle
CADM2	rs1375558	3.00E-18	-0.4	Lung
CADM2		5.30E-06	-0.2	Adipose - Subcutaneous
CADM2		5.00E-09	-0.27	Lung
CADM2-AS1	rs1375559	1.40E-04	0.25	Nerve - Tibial
CADM2		2.20E-11	-0.28	Adipose - Subcutaneous
CADM2		5.20E-08	-0.25	Adipose - Visceral (Omentum)
CADM2		1.10E-06	-0.39	Heart - Left Ventricle
CADM2	rs1375560	9.40E-17	-0.39	Lung
CADM2		7.70E-06	-0.21	Lung
CADM2-AS1	rs1375561	9.20E-05	0.26	Nerve - Tibial
CADM2		3.10E-06	-0.2	Adipose - Subcutaneous
CADM2		8.60E-05	-0.18	Adipose - Visceral (Omentum)
CADM2	rs1375564	3.40E-11	-0.33	Lung
CADM2	rs1375565	3.70E-11	-0.28	Adipose - Subcutaneous

CADM2		4.50E-08	-0.24	Adipose - Visceral (Omentum)
CADM2		2.20E-06	-0.37	Heart - Left Ventricle
CADM2		4.00E-18	-0.4	Lung
CADM2		1.70E-11	-0.28	Adipose - Subcutaneous
CADM2		1.30E-07	-0.24	Adipose - Visceral (Omentum)
CADM2		1.10E-06	-0.38	Heart - Left Ventricle
CADM2	rs1375566	5.20E-19	-0.41	Lung
CADM2		4.60E-06	-0.2	Adipose - Subcutaneous
CADM2	rs1375567	3.80E-09	-0.27	Lung
CADM2		1.30E-04	0.18	Adipose - Subcutaneous
CADM2		3.30E-05	0.32	Brain - Hippocampus
CADM2	rs1376935	1.40E-07	0.84	Brain - Spinal cord (cervical c-1)
CADM2	rs1376938	9.20E-06	-0.66	Brain - Hippocampus
CADM2	rs138343278	2.80E-05	-0.61	Brain - Hippocampus
CADM2-AS1	rs138497721	2.80E-05	-0.47	Brain - Cerebellar Hemisphere
CADM2	rs138605722	2.30E-05	-0.18	Muscle - Skeletal
CADM2	rs139166952	3.50E-05	-0.61	Brain - Hippocampus
CADM2	rs139629585	8.50E-05	-0.19	Muscle - Skeletal
CADM2	rs139639176	2.10E-05	0.4	Adipose - Subcutaneous
CADM2		7.00E-12	-0.29	Adipose - Subcutaneous
CADM2		1.40E-08	-0.25	Adipose - Visceral (Omentum)
CADM2		7.00E-07	-0.39	Heart - Left Ventricle
CADM2		1.40E-18	-0.41	Lung
CADM2	rs139750202	1.60E-04	0.1	Nerve - Tibial
CADM2	rs139751954	1.20E-05	-0.19	Muscle - Skeletal
CADM2	rs139961348	9.20E-06	-0.66	Brain - Hippocampus
CADM2-AS1		6.10E-07	0.62	Brain - Caudate (basal ganglia)
CADM2-AS1		2.50E-10	0.74	Brain - Cerebellar Hemisphere
CADM2-AS1		1.80E-12	0.65	Brain - Cerebellum
CADM2-AS1		3.50E-06	0.54	Brain - Cortex
CADM2-AS1		5.40E-07	0.64	Brain - Frontal Cortex (BA9)
CADM2-AS1		1.60E-06	0.67	Brain - Hippocampus
CADM2-AS1		9.70E-09	0.68	Brain - Nucleus accumbens (basal ganglia)
CADM2-AS1	rs141343295	1.80E-05	0.57	Brain - Putamen (basal ganglia)
CADM2-AS1	rs141593685	5.70E-05	-0.29	Nerve - Tibial
CADM2	rs142356570	6.10E-06	-0.21	Muscle - Skeletal
CADM2-AS1	rs142612147	3.80E-05	0.99	Brain - Frontal Cortex (BA9)
CADM2		1.30E-10	-0.27	Adipose - Subcutaneous
CADM2		5.80E-08	-0.24	Adipose - Visceral (Omentum)
CADM2		2.70E-07	-0.4	Heart - Left Ventricle
CADM2		1.60E-14	-0.36	Lung
CADM2	rs1433708	6.30E-05	-0.15	Muscle - Skeletal
CADM2		6.70E-05	-0.43	Heart - Left Ventricle
CADM2	rs1433710	2.30E-07	-0.34	Lung
CADM2		3.60E-08	0.26	Adipose - Subcutaneous
CADM2		1.00E-04	0.2	Adipose - Visceral (Omentum)
CADM2		5.40E-05	0.21	Breast - Mammary Tissue
CADM2	rs1433711	2.10E-06	0.27	Lung
CADM2	rs1433713	1.10E-07	-0.35	Lung
CADM2		1.90E-11	-0.28	Adipose - Subcutaneous
CADM2	rs1433714	6.20E-08	-0.24	Adipose - Visceral (Omentum)

CADM2		2.60E-07	-0.4	Heart - Left Ventricle
CADM2		1.60E-16	-0.39	Lung
CADM2		2.50E-10	-0.27	Adipose - Subcutaneous
CADM2		6.20E-07	-0.22	Adipose - Visceral (Omentum)
CADM2		1.10E-07	-0.42	Heart - Left Ventricle
CADM2	rs1433715	1.10E-13	-0.36	Lung
CADM2	rs144638060	1.40E-05	-0.64	Brain - Hippocampus
CADM2		7.80E-05	0.23	Adipose - Visceral (Omentum)
CADM2		3.70E-06	0.28	Lung
CADM2-AS1	rs1448602	1.50E-04	-0.29	Nerve - Tibial
CADM2-AS1		2.20E-06	0.54	Brain - Cerebellar Hemisphere
CADM2-AS1		1.10E-08	0.51	Brain - Cerebellum
CADM2-AS1	rs1448607	2.70E-05	0.5	Brain - Nucleus accumbens (basal ganglia)
CADM2		8.00E-05	0.23	Adipose - Visceral (Omentum)
CADM2		8.20E-06	0.27	Lung
CADM2-AS1	rs1448608	1.30E-04	-0.29	Nerve - Tibial
CADM2-AS1		5.10E-06	0.62	Brain - Caudate (basal ganglia)
CADM2-AS1	rs1448609	2.80E-06	0.51	Brain - Cerebellum
CADM2-AS1	rs1448610	3.10E-05	0.44	Brain - Cerebellum
CADM2-AS1		1.40E-07	0.6	Brain - Caudate (basal ganglia)
CADM2-AS1		3.30E-05	0.47	Brain - Cerebellar Hemisphere
CADM2-AS1	rs1448613	2.70E-07	0.48	Brain - Cerebellum
CADM2-AS1		1.40E-07	0.59	Brain - Caudate (basal ganglia)
CADM2-AS1		3.30E-05	0.47	Brain - Cerebellar Hemisphere
CADM2-AS1	rs1448614	2.70E-07	0.48	Brain - Cerebellum
CADM2-AS1		2.90E-05	0.53	Brain - Caudate (basal ganglia)
CADM2-AS1		6.50E-07	0.59	Brain - Cerebellar Hemisphere
CADM2-AS1		8.80E-08	0.51	Brain - Cerebellum
CADM2-AS1		2.10E-05	0.53	Brain - Frontal Cortex (BA9)
CADM2-AS1	rs1448616	2.00E-05	0.5	Brain - Nucleus accumbens (basal ganglia)
CADM2		2.50E-11	-0.28	Adipose - Subcutaneous
CADM2		2.70E-07	-0.24	Adipose - Visceral (Omentum)
CADM2		3.80E-07	-0.4	Heart - Left Ventricle
CADM2		3.50E-18	-0.41	Lung
CADM2	rs1449370	1.10E-04	0.1	Nerve - Tibial
CADM2		6.80E-12	-0.29	Adipose - Subcutaneous
CADM2		2.60E-08	-0.25	Adipose - Visceral (Omentum)
CADM2		1.20E-06	-0.39	Heart - Left Ventricle
CADM2		1.40E-16	-0.4	Lung
CADM2	rs1449371	1.40E-04	0.1	Nerve - Tibial
CADM2		2.70E-12	-0.29	Adipose - Subcutaneous
CADM2		2.60E-08	-0.25	Adipose - Visceral (Omentum)
CADM2		5.10E-07	-0.4	Heart - Left Ventricle
CADM2	rs1449372	1.60E-17	-0.4	Lung
CADM2		4.70E-12	-0.29	Adipose - Subcutaneous
CADM2		5.90E-08	-0.25	Adipose - Visceral (Omentum)
CADM2		5.40E-07	-0.4	Heart - Left Ventricle
CADM2		1.30E-18	-0.41	Lung
CADM2	rs1449373	1.50E-04	0.1	Nerve - Tibial
CADM2	rs1449374	2.10E-13	-0.31	Adipose - Subcutaneous

CADM2		1.10E-08	-0.26	Adipose - Visceral (Omentum)
CADM2		5.50E-07	-0.4	Heart - Left Ventricle
CADM2		4.20E-18	-0.41	Lung
CADM2		8.80E-05	0.11	Nerve - Tibial
CADM2		5.70E-12	-0.29	Adipose - Subcutaneous
CADM2		3.30E-08	-0.25	Adipose - Visceral (Omentum)
CADM2		8.20E-07	-0.39	Heart - Left Ventricle
CADM2		1.10E-17	-0.4	Lung
CADM2	rs1449375	1.80E-04	0.099	Nerve - Tibial
CADM2		6.80E-12	-0.29	Adipose - Subcutaneous
CADM2		1.50E-08	-0.25	Adipose - Visceral (Omentum)
CADM2		7.40E-07	-0.39	Heart - Left Ventricle
CADM2		1.80E-18	-0.41	Lung
CADM2	rs1449378	1.60E-04	0.099	Nerve - Tibial
CADM2		6.80E-12	-0.29	Adipose - Subcutaneous
CADM2		1.50E-08	-0.25	Adipose - Visceral (Omentum)
CADM2		7.40E-07	-0.39	Heart - Left Ventricle
CADM2		1.80E-18	-0.41	Lung
CADM2	rs1449379	1.60E-04	0.099	Nerve - Tibial
CADM2		6.80E-12	-0.29	Adipose - Subcutaneous
CADM2		1.50E-08	-0.25	Adipose - Visceral (Omentum)
CADM2		7.40E-07	-0.39	Heart - Left Ventricle
CADM2		1.80E-18	-0.41	Lung
CADM2	rs1449380	1.60E-04	0.099	Nerve - Tibial
CADM2		7.50E-12	-0.29	Adipose - Subcutaneous
CADM2		1.60E-08	-0.25	Adipose - Visceral (Omentum)
CADM2		7.40E-07	-0.39	Heart - Left Ventricle
CADM2		1.80E-18	-0.41	Lung
CADM2	rs1449381	1.70E-04	0.099	Nerve - Tibial
CADM2		6.80E-12	-0.29	Adipose - Subcutaneous
CADM2		1.50E-08	-0.25	Adipose - Visceral (Omentum)
CADM2		7.40E-07	-0.39	Heart - Left Ventricle
CADM2		1.80E-18	-0.41	Lung
CADM2	rs1449382	1.60E-04	0.099	Nerve - Tibial
CADM2		4.40E-12	-0.29	Adipose - Subcutaneous
CADM2		2.90E-08	-0.25	Adipose - Visceral (Omentum)
CADM2		4.90E-07	-0.39	Heart - Left Ventricle
CADM2		1.50E-17	-0.4	Lung
CADM2	rs1449383	1.10E-04	0.1	Nerve - Tibial
CADM2		4.50E-12	-0.29	Adipose - Subcutaneous
CADM2		3.00E-08	-0.25	Adipose - Visceral (Omentum)
CADM2		5.90E-07	-0.39	Heart - Left Ventricle
CADM2		2.00E-17	-0.4	Lung
CADM2	rs1449384	1.80E-04	0.098	Nerve - Tibial
CADM2		3.00E-12	-0.29	Adipose - Subcutaneous
CADM2		2.60E-08	-0.25	Adipose - Visceral (Omentum)
CADM2		8.90E-07	-0.39	Heart - Left Ventricle
CADM2		2.20E-17	-0.4	Lung
CADM2	rs1449385	1.20E-04	0.1	Nerve - Tibial
CADM2		3.60E-11	-0.28	Adipose - Subcutaneous
CADM2	rs1449386	1.30E-07	-0.24	Adipose - Visceral (Omentum)

CADM2		5.90E-07	-0.39	Heart - Left Ventricle
CADM2		8.30E-18	-0.4	Lung
CADM2		1.90E-06	-0.2	Adipose - Subcutaneous
CADM2	rs1449388	1.80E-09	-0.27	Lung
CADM2		1.80E-12	-0.29	Adipose - Subcutaneous
CADM2		1.50E-08	-0.25	Adipose - Visceral (Omentum)
CADM2		7.40E-07	-0.39	Heart - Left Ventricle
CADM2		3.90E-18	-0.4	Lung
CADM2	rs1449389	1.70E-04	0.099	Nerve - Tibial
CADM2		1.80E-12	-0.29	Adipose - Subcutaneous
CADM2		1.50E-08	-0.25	Adipose - Visceral (Omentum)
CADM2		7.40E-07	-0.39	Heart - Left Ventricle
CADM2		3.90E-18	-0.4	Lung
CADM2	rs1449390	1.70E-04	0.099	Nerve - Tibial
CADM2		4.40E-12	-0.29	Adipose - Subcutaneous
CADM2		1.50E-08	-0.25	Adipose - Visceral (Omentum)
CADM2		6.40E-07	-0.39	Heart - Left Ventricle
CADM2	rs1449391	3.90E-18	-0.4	Lung
CADM2		5.00E-12	-0.29	Adipose - Subcutaneous
CADM2		4.10E-08	-0.25	Adipose - Visceral (Omentum)
CADM2		1.50E-06	-0.38	Heart - Left Ventricle
CADM2	rs1449393	3.10E-17	-0.4	Lung
CADM2		3.10E-11	-0.28	Adipose - Subcutaneous
CADM2		6.30E-08	-0.25	Adipose - Visceral (Omentum)
CADM2		6.60E-07	-0.39	Heart - Left Ventricle
CADM2	rs1449394	1.30E-16	-0.39	Lung
CADM2		3.00E-05	0.22	Adipose - Subcutaneous
CADM2	rs1449395	6.90E-06	0.27	Lung
CADM2		8.20E-05	0.2	Adipose - Subcutaneous
CADM2		3.60E-05	0.24	Lung
CADM2-AS1	rs1449396	7.70E-05	-0.29	Nerve - Tibial
CADM2	rs1449398	1.00E-05	-0.21	Lung
CADM2		1.20E-06	-0.21	Adipose - Subcutaneous
CADM2	rs1449400	1.30E-10	-0.32	Lung
CADM2		2.00E-09	-0.25	Adipose - Subcutaneous
CADM2		2.70E-07	-0.23	Adipose - Visceral (Omentum)
CADM2		2.90E-07	-0.39	Heart - Left Ventricle
CADM2	rs1449401	1.40E-19	-0.42	Lung
CADM2		2.00E-09	-0.25	Adipose - Subcutaneous
CADM2		3.60E-07	-0.23	Adipose - Visceral (Omentum)
CADM2		3.70E-07	-0.38	Heart - Left Ventricle
CADM2	rs1449402	1.20E-19	-0.42	Lung
CADM2	rs1449403	1.20E-06	0.35	Lung
CADM2		3.10E-08	0.29	Adipose - Subcutaneous
CADM2		5.40E-06	0.25	Adipose - Visceral (Omentum)
CADM2	rs1449404	4.70E-09	0.34	Lung
CADM2		6.40E-12	-0.29	Adipose - Subcutaneous
CADM2		7.80E-09	-0.26	Adipose - Visceral (Omentum)
CADM2		5.00E-07	-0.39	Heart - Left Ventricle
CADM2		8.80E-19	-0.41	Lung
CADM2	rs1449405	1.60E-04	0.099	Nerve - Tibial
CADM2	rs1449406	6.80E-12	-0.29	Adipose - Subcutaneous

CADM2		1.50E-08	-0.25	Adipose - Visceral (Omentum)
CADM2		7.40E-07	-0.39	Heart - Left Ventricle
CADM2		1.80E-18	-0.41	Lung
CADM2		1.60E-04	0.099	Nerve - Tibial
CADM2		6.80E-12	-0.29	Adipose - Subcutaneous
CADM2		1.50E-08	-0.25	Adipose - Visceral (Omentum)
CADM2		7.40E-07	-0.39	Heart - Left Ventricle
CADM2		1.80E-18	-0.41	Lung
CADM2	rs1449407	1.60E-04	0.099	Nerve - Tibial
CADM2		1.90E-11	-0.28	Adipose - Subcutaneous
CADM2		7.30E-09	-0.26	Adipose - Visceral (Omentum)
CADM2		6.70E-07	-0.39	Heart - Left Ventricle
CADM2		1.70E-18	-0.41	Lung
CADM2	rs1449408	1.30E-04	0.1	Nerve - Tibial
CADM2	rs1449409	2.30E-06	-0.3	Lung
CADM2		6.80E-12	-0.29	Adipose - Subcutaneous
CADM2		1.50E-08	-0.25	Adipose - Visceral (Omentum)
CADM2		7.40E-07	-0.39	Heart - Left Ventricle
CADM2		1.80E-18	-0.41	Lung
CADM2	rs1449410	1.60E-04	0.099	Nerve - Tibial
CADM2-AS1		2.10E-06	0.59	Brain - Caudate (basal ganglia)
CADM2-AS1		6.70E-08	0.64	Brain - Cerebellar Hemisphere
CADM2-AS1		1.00E-09	0.55	Brain - Cerebellum
CADM2-AS1		6.20E-06	0.51	Brain - Cortex
CADM2-AS1		2.90E-06	0.63	Brain - Frontal Cortex (BA9)
CADM2-AS1		1.40E-05	0.59	Brain - Hippocampus
CADM2-AS1	rs145178380	2.10E-06	0.59	Brain - Nucleus accumbens (basal ganglia)
CADM2	rs1452126	1.00E-05	-0.66	Brain - Hippocampus
CADM2	rs1452127	1.20E-05	-0.65	Brain - Hippocampus
CADM2-AS1	rs145445868	4.20E-05	-0.47	Brain - Cerebellar Hemisphere
CADM2		1.90E-05	0.17	Adipose - Subcutaneous
CADM2	rs146233786	2.10E-07	0.18	Muscle - Skeletal
CADM2		6.60E-12	-0.29	Adipose - Subcutaneous
CADM2		1.00E-08	-0.26	Adipose - Visceral (Omentum)
CADM2		7.40E-07	-0.39	Heart - Left Ventricle
CADM2		2.60E-18	-0.41	Lung
CADM2	rs1463203	1.70E-04	0.099	Nerve - Tibial
CADM2		6.10E-12	-0.29	Adipose - Subcutaneous
CADM2		1.50E-08	-0.25	Adipose - Visceral (Omentum)
CADM2		7.90E-07	-0.39	Heart - Left Ventricle
CADM2		1.60E-18	-0.41	Lung
CADM2	rs1463204	1.70E-04	0.099	Nerve - Tibial
CADM2		6.10E-12	-0.29	Adipose - Subcutaneous
CADM2		9.70E-09	-0.25	Adipose - Visceral (Omentum)
CADM2		7.40E-07	-0.39	Heart - Left Ventricle
CADM2		2.40E-18	-0.41	Lung
CADM2	rs1463205	1.70E-04	0.099	Nerve - Tibial
CADM2		6.80E-12	-0.29	Adipose - Subcutaneous
CADM2		1.50E-08	-0.25	Adipose - Visceral (Omentum)
CADM2		7.40E-07	-0.39	Heart - Left Ventricle
CADM2	rs1463206	1.80E-18	-0.41	Lung

CADM2		1.60E-04	0.099	Nerve - Tibial
CADM2		6.80E-12	-0.29	Adipose - Subcutaneous
CADM2		1.50E-08	-0.25	Adipose - Visceral (Omentum)
CADM2		7.40E-07	-0.39	Heart - Left Ventricle
CADM2		1.70E-18	-0.41	Lung
CADM2	rs1463207	1.60E-04	0.099	Nerve - Tibial
CADM2		6.80E-12	-0.29	Adipose - Subcutaneous
CADM2		1.50E-08	-0.25	Adipose - Visceral (Omentum)
CADM2		7.40E-07	-0.39	Heart - Left Ventricle
CADM2		1.70E-18	-0.41	Lung
CADM2	rs1463208	1.60E-04	0.099	Nerve - Tibial
CADM2	rs1465716	2.30E-07	-0.34	Lung
CADM2-AS1		3.50E-07	0.61	Brain - Caudate (basal ganglia)
CADM2-AS1		9.10E-10	0.69	Brain - Cerebellar Hemisphere
CADM2-AS1		2.10E-10	0.59	Brain - Cerebellum
CADM2-AS1		1.70E-05	0.52	Brain - Frontal Cortex (BA9)
CADM2-AS1		2.20E-05	0.56	Brain - Hippocampus
CADM2-AS1		1.00E-06	0.57	Brain - Nucleus accumbens (basal ganglia)
CADM2-AS1	rs146678023	9.60E-05	0.25	Nerve - Tibial
CADM2		3.70E-12	-0.29	Adipose - Subcutaneous
CADM2		1.40E-08	-0.25	Adipose - Visceral (Omentum)
CADM2		5.70E-07	-0.39	Heart - Left Ventricle
CADM2	rs1470635	4.20E-18	-0.4	Lung
CADM2	rs1472302	1.80E-05	0.16	Muscle - Skeletal
CADM2-AS1	rs147955519	3.80E-05	0.99	Brain - Frontal Cortex (BA9)
CADM2	rs148222912	9.60E-05	0.33	Adipose - Subcutaneous
CADM2		1.30E-11	-0.28	Adipose - Subcutaneous
CADM2		5.40E-08	-0.24	Adipose - Visceral (Omentum)
CADM2		3.60E-07	-0.4	Heart - Left Ventricle
CADM2	rs148921522	1.00E-17	-0.4	Lung
CADM2		5.10E-05	0.23	Adipose - Visceral (Omentum)
CADM2	rs149984670	6.40E-06	0.27	Lung
CADM2	rs150919222	7.70E-05	0.19	Brain - Cerebellum
CADM2-AS1	rs1512916	5.70E-05	-0.29	Nerve - Tibial
CADM2		9.20E-12	-0.29	Adipose - Subcutaneous
CADM2		2.80E-08	-0.25	Adipose - Visceral (Omentum)
CADM2		7.40E-07	-0.39	Heart - Left Ventricle
CADM2		7.20E-18	-0.4	Lung
CADM2	rs1530738	1.10E-04	0.1	Nerve - Tibial
CADM2		6.80E-12	-0.29	Adipose - Subcutaneous
CADM2		1.50E-08	-0.25	Adipose - Visceral (Omentum)
CADM2		7.40E-07	-0.39	Heart - Left Ventricle
CADM2		1.80E-18	-0.41	Lung
CADM2	rs1530739	1.60E-04	0.099	Nerve - Tibial
CADM2		6.80E-12	-0.29	Adipose - Subcutaneous
CADM2		5.70E-08	-0.24	Adipose - Visceral (Omentum)
CADM2		9.20E-07	-0.38	Heart - Left Ventricle
CADM2	rs1530740	4.90E-18	-0.41	Lung
CADM2		2.90E-12	-0.3	Adipose - Subcutaneous
CADM2		1.40E-08	-0.26	Adipose - Visceral (Omentum)
CADM2	rs1542248	8.00E-07	-0.39	Heart - Left Ventricle

CADM2		2.10E-17	-0.41	Lung
CADM2		2.20E-10	-0.27	Adipose - Subcutaneous
CADM2		5.50E-08	-0.25	Adipose - Visceral (Omentum)
CADM2		4.40E-07	-0.41	Heart - Left Ventricle
CADM2		2.10E-17	-0.4	Lung
CADM2	rs1549979	1.70E-04	-0.14	Muscle - Skeletal
CADM2		2.60E-07	-0.22	Adipose - Subcutaneous
CADM2		2.30E-06	-0.22	Adipose - Visceral (Omentum)
CADM2		1.40E-06	-0.38	Heart - Left Ventricle
CADM2	rs1551042	1.90E-16	-0.39	Lung
CADM2		6.10E-12	-0.29	Adipose - Subcutaneous
CADM2		1.40E-08	-0.25	Adipose - Visceral (Omentum)
CADM2		6.40E-07	-0.39	Heart - Left Ventricle
CADM2	rs1551043	8.00E-18	-0.4	Lung
CADM2		3.10E-09	-0.24	Adipose - Subcutaneous
CADM2		1.10E-07	-0.24	Adipose - Visceral (Omentum)
CADM2		1.60E-07	-0.4	Heart - Left Ventricle
CADM2	rs1551044	2.40E-19	-0.41	Lung
CADM2		5.50E-12	-0.29	Adipose - Subcutaneous
CADM2		1.00E-08	-0.26	Adipose - Visceral (Omentum)
CADM2		7.40E-07	-0.39	Heart - Left Ventricle
CADM2		2.60E-18	-0.41	Lung
CADM2	rs1551045	1.70E-04	0.099	Nerve - Tibial
CADM2		8.80E-12	-0.29	Adipose - Subcutaneous
CADM2		1.60E-08	-0.25	Adipose - Visceral (Omentum)
CADM2		7.40E-07	-0.39	Heart - Left Ventricle
CADM2		1.50E-18	-0.41	Lung
CADM2	rs1551046	1.60E-04	0.1	Nerve - Tibial
CADM2		1.50E-11	-0.28	Adipose - Subcutaneous
CADM2		3.50E-08	-0.25	Adipose - Visceral (Omentum)
CADM2		4.00E-07	-0.4	Heart - Left Ventricle
CADM2	rs1551047	4.10E-18	-0.4	Lung
CADM2		1.30E-11	-0.28	Adipose - Subcutaneous
CADM2		1.40E-08	-0.26	Adipose - Visceral (Omentum)
CADM2		3.10E-07	-0.4	Heart - Left Ventricle
CADM2	rs1551048	2.40E-18	-0.41	Lung
CADM2-AS1		1.00E-05	0.57	Brain - Caudate (basal ganglia)
CADM2-AS1		2.10E-07	0.62	Brain - Cerebellar Hemisphere
CADM2-AS1		3.60E-08	0.51	Brain - Cerebellum
CADM2-AS1		4.00E-05	0.48	Brain - Cortex
CADM2-AS1		6.20E-06	0.57	Brain - Frontal Cortex (BA9)
CADM2-AS1	rs1562626	1.70E-05	0.5	Brain - Nucleus accumbens (basal ganglia)
CADM2-AS1	rs1567736	4.10E-05	-0.29	Nerve - Tibial
CADM2		7.90E-05	-0.43	Heart - Left Ventricle
CADM2	rs1594795	2.30E-07	-0.34	Lung
CADM2	rs1597213	1.10E-04	0.2	Adipose - Subcutaneous
CADM2		6.80E-12	-0.29	Adipose - Subcutaneous
CADM2		1.50E-08	-0.25	Adipose - Visceral (Omentum)
CADM2		7.40E-07	-0.39	Heart - Left Ventricle
CADM2		1.80E-18	-0.41	Lung
CADM2	rs1597315	1.60E-04	0.099	Nerve - Tibial

CADM2	rs1603201	3.20E-05	-0.19	Muscle - Skeletal
CADM2		7.30E-05	0.17	Adipose - Subcutaneous
CADM2		2.00E-05	0.56	Brain - Spinal cord (cervical c-1)
CADM2	rs1691471	4.40E-06	0.17	Muscle - Skeletal
CADM2	rs1691473	8.40E-06	0.97	Spleen
CADM2	rs1691483	5.10E-05	-0.15	Muscle - Skeletal
CADM2		1.50E-05	0.2	Adipose - Subcutaneous
CADM2	rs1694919	1.70E-07	0.21	Muscle - Skeletal
CADM2	rs1694924	4.00E-05	-0.15	Muscle - Skeletal
CADM2	rs1694927	1.70E-04	-0.15	Muscle - Skeletal
CADM2		1.50E-04	0.15	Adipose - Subcutaneous
CADM2	rs1694929	7.00E-06	0.16	Muscle - Skeletal
CADM2	rs1694933	2.60E-06	-0.54	Brain - Spinal cord (cervical c-1)
CADM2	rs1694977	4.20E-06	0.18	Muscle - Skeletal
CADM2	rs17022126	1.60E-06	1.2	Spleen
CADM2	rs17022171	2.60E-08	1	Heart - Left Ventricle
CADM2	rs17022259	1.40E-08	1.1	Heart - Left Ventricle
CADM2	rs17022281	2.60E-08	1	Heart - Left Ventricle
CADM2	rs17022298	2.60E-08	1	Heart - Left Ventricle
CADM2	rs17022305	6.40E-08	1.2	Spleen
CADM2	rs17022410	5.00E-06	-0.71	Brain - Hippocampus
CADM2	rs17022559	1.30E-05	-0.68	Brain - Hippocampus
CADM2		6.10E-05	-0.2	Adipose - Subcutaneous
CADM2		6.70E-05	-0.21	Adipose - Visceral (Omentum)
CADM2	rs17022879	3.70E-05	-0.23	Lung
CADM2		1.60E-06	-0.21	Adipose - Subcutaneous
CADM2		2.00E-09	-0.28	Lung
CADM2-AS1	rs17022886	7.40E-05	0.26	Nerve - Tibial
CADM2		8.20E-07	-0.21	Adipose - Subcutaneous
CADM2		2.10E-09	-0.27	Lung
CADM2-AS1	rs17022915	1.10E-04	0.26	Nerve - Tibial
CADM2		9.90E-07	-0.21	Adipose - Subcutaneous
CADM2	rs17022938	1.80E-09	-0.27	Lung
CADM2		1.20E-08	-0.26	Adipose - Subcutaneous
CADM2		1.20E-05	-0.21	Adipose - Visceral (Omentum)
CADM2	rs17022974	2.00E-11	-0.34	Lung
CADM2		1.20E-06	-0.21	Adipose - Subcutaneous
CADM2	rs17023016	4.90E-09	-0.27	Lung
CADM2		4.50E-10	-0.26	Adipose - Subcutaneous
CADM2		9.10E-08	-0.24	Adipose - Visceral (Omentum)
CADM2		1.40E-07	-0.4	Heart - Left Ventricle
CADM2	rs17023019	3.00E-19	-0.41	Lung
CADM2		1.50E-10	-0.27	Adipose - Subcutaneous
CADM2		1.30E-07	-0.24	Adipose - Visceral (Omentum)
CADM2		2.10E-07	-0.4	Heart - Left Ventricle
CADM2	rs17023032	5.20E-17	-0.39	Lung
CADM2-AS1		8.90E-06	0.58	Brain - Caudate (basal ganglia)
CADM2-AS1	rs17023296	5.40E-06	0.49	Brain - Cerebellum
CADM2-AS1		3.00E-07	0.58	Brain - Caudate (basal ganglia)
CADM2-AS1		3.30E-05	0.47	Brain - Cerebellar Hemisphere
CADM2-AS1	rs17023377	2.70E-07	0.48	Brain - Cerebellum

CADM2-AS1		4.20E-06	0.62	Brain - Caudate (basal ganglia)
CADM2-AS1	rs17023388	1.40E-06	0.53	Brain - Cerebellum
CADM2-AS1		1.30E-07	0.62	Brain - Caudate (basal ganglia)
CADM2-AS1		5.90E-08	0.61	Brain - Cerebellar Hemisphere
CADM2-AS1		4.20E-07	0.45	Brain - Cerebellum
CADM2-AS1		4.50E-06	0.48	Brain - Cortex
CADM2-AS1		5.90E-06	0.57	Brain - Frontal Cortex (BA9)
CADM2-AS1		1.60E-05	0.56	Brain - Hippocampus
CADM2-AS1	rs17025272	1.20E-05	0.51	Brain - Nucleus accumbens (basal ganglia)
CADM2-AS1	rs17026558	1.40E-04	-0.27	Nerve - Tibial
CADM2		9.00E-10	-0.25	Adipose - Subcutaneous
CADM2		8.20E-08	-0.24	Adipose - Visceral (Omentum)
CADM2		8.80E-08	-0.4	Heart - Left Ventricle
CADM2	rs17455991	1.60E-19	-0.41	Lung
CADM2		1.90E-11	-0.28	Adipose - Subcutaneous
CADM2		1.40E-08	-0.26	Adipose - Visceral (Omentum)
CADM2		6.90E-07	-0.39	Heart - Left Ventricle
CADM2		5.40E-18	-0.41	Lung
CADM2		6.80E-12	-0.29	Adipose - Subcutaneous
CADM2		1.50E-08	-0.25	Adipose - Visceral (Omentum)
CADM2		7.40E-07	-0.39	Heart - Left Ventricle
CADM2		1.80E-18	-0.41	Lung
CADM2	rs17456263	1.60E-04	0.099	Nerve - Tibial
CADM2		6.80E-12	-0.29	Adipose - Subcutaneous
CADM2		1.50E-08	-0.25	Adipose - Visceral (Omentum)
CADM2		7.40E-07	-0.39	Heart - Left Ventricle
CADM2		1.80E-18	-0.41	Lung
CADM2	rs17456820	1.60E-04	0.099	Nerve - Tibial
CADM2		7.70E-12	-0.29	Adipose - Subcutaneous
CADM2		3.80E-08	-0.25	Adipose - Visceral (Omentum)
CADM2		1.70E-06	-0.38	Heart - Left Ventricle
CADM2	rs17457050	2.60E-18	-0.41	Lung
CADM2		7.10E-12	-0.29	Adipose - Subcutaneous
CADM2		1.10E-08	-0.26	Adipose - Visceral (Omentum)
CADM2		9.10E-07	-0.39	Heart - Left Ventricle
CADM2	rs17457189	2.90E-18	-0.41	Lung
CADM2		3.60E-12	-0.29	Adipose - Subcutaneous
CADM2		9.70E-09	-0.26	Adipose - Visceral (Omentum)
CADM2		7.40E-07	-0.39	Heart - Left Ventricle
CADM2		1.80E-18	-0.41	Lung
CADM2	rs17457217	1.50E-04	0.1	Nerve - Tibial
CADM2		1.50E-11	-0.28	Adipose - Subcutaneous
CADM2		2.00E-07	-0.23	Adipose - Visceral (Omentum)
CADM2		3.50E-07	-0.4	Heart - Left Ventricle
CADM2		3.50E-17	-0.4	Lung
CADM2	rs17457377	1.20E-04	0.1	Nerve - Tibial
CADM2		5.20E-12	-0.29	Adipose - Subcutaneous
CADM2		1.10E-08	-0.26	Adipose - Visceral (Omentum)
CADM2		3.40E-07	-0.4	Heart - Left Ventricle
CADM2	rs17457426	5.90E-18	-0.4	Lung
CADM2	rs17457454	3.20E-12	-0.29	Adipose - Subcutaneous

CADM2		9.40E-09	-0.26	Adipose - Visceral (Omentum)
CADM2		7.00E-07	-0.39	Heart - Left Ventricle
CADM2		1.60E-18	-0.41	Lung
CADM2		1.50E-04	0.1	Nerve - Tibial
CADM2		6.80E-12	-0.29	Adipose - Subcutaneous
CADM2		1.50E-08	-0.25	Adipose - Visceral (Omentum)
CADM2		3.60E-07	-0.4	Heart - Left Ventricle
CADM2		3.30E-18	-0.4	Lung
CADM2	rs17457642	1.60E-04	0.099	Nerve - Tibial
CADM2		6.80E-12	-0.29	Adipose - Subcutaneous
CADM2		1.50E-08	-0.25	Adipose - Visceral (Omentum)
CADM2		7.40E-07	-0.39	Heart - Left Ventricle
CADM2		1.80E-18	-0.41	Lung
CADM2	rs17457669	1.60E-04	0.099	Nerve - Tibial
CADM2		6.90E-12	-0.29	Adipose - Subcutaneous
CADM2		1.60E-08	-0.25	Adipose - Visceral (Omentum)
CADM2		1.20E-06	-0.39	Heart - Left Ventricle
CADM2		2.40E-18	-0.41	Lung
CADM2	rs17458504	1.40E-04	0.1	Nerve - Tibial
CADM2		6.40E-12	-0.29	Adipose - Subcutaneous
CADM2		1.60E-08	-0.25	Adipose - Visceral (Omentum)
CADM2		7.40E-07	-0.39	Heart - Left Ventricle
CADM2		1.80E-18	-0.41	Lung
CADM2	rs17458574	1.60E-04	0.099	Nerve - Tibial
CADM2		1.00E-11	-0.28	Adipose - Subcutaneous
CADM2		1.60E-08	-0.25	Adipose - Visceral (Omentum)
CADM2		7.40E-07	-0.39	Heart - Left Ventricle
CADM2	rs17458609	5.20E-18	-0.4	Lung
CADM2		6.80E-12	-0.29	Adipose - Subcutaneous
CADM2		1.50E-08	-0.25	Adipose - Visceral (Omentum)
CADM2		7.40E-07	-0.39	Heart - Left Ventricle
CADM2		1.80E-18	-0.41	Lung
CADM2	rs17459563	1.60E-04	0.099	Nerve - Tibial
CADM2		8.30E-12	-0.29	Adipose - Subcutaneous
CADM2		1.90E-08	-0.25	Adipose - Visceral (Omentum)
CADM2		6.50E-07	-0.39	Heart - Left Ventricle
CADM2		1.70E-18	-0.41	Lung
CADM2	rs17459906	1.40E-04	0.1	Nerve - Tibial
CADM2		6.20E-12	-0.29	Adipose - Subcutaneous
CADM2		1.50E-08	-0.25	Adipose - Visceral (Omentum)
CADM2		7.40E-07	-0.39	Heart - Left Ventricle
CADM2		8.10E-19	-0.41	Lung
CADM2	rs17460541	1.60E-04	0.1	Nerve - Tibial
CADM2	rs17460569	5.00E-05	0.31	Lung
CADM2		7.40E-12	-0.29	Adipose - Subcutaneous
CADM2		1.50E-08	-0.25	Adipose - Visceral (Omentum)
CADM2		7.40E-07	-0.39	Heart - Left Ventricle
CADM2		1.80E-18	-0.41	Lung
CADM2	rs17460701	1.60E-04	0.099	Nerve - Tibial
CADM2		8.10E-11	-0.28	Adipose - Subcutaneous
CADM2	rs17515196	3.60E-08	-0.26	Adipose - Visceral (Omentum)

CADM2		3.50E-07	-0.41	Heart - Left Ventricle
CADM2		2.30E-15	-0.39	Lung
CADM2		1.20E-12	-0.3	Adipose - Subcutaneous
CADM2		2.70E-08	-0.25	Adipose - Visceral (Omentum)
CADM2		1.40E-06	-0.38	Heart - Left Ventricle
CADM2		1.40E-16	-0.39	Lung
CADM2	rs17515586	1.60E-04	0.1	Nerve - Tibial
CADM2		2.10E-11	-0.28	Adipose - Subcutaneous
CADM2		7.80E-09	-0.26	Adipose - Visceral (Omentum)
CADM2		5.00E-07	-0.39	Heart - Left Ventricle
CADM2		2.50E-18	-0.41	Lung
CADM2	rs17516256	1.70E-04	0.099	Nerve - Tibial
CADM2		3.70E-12	-0.29	Adipose - Subcutaneous
CADM2		8.60E-09	-0.26	Adipose - Visceral (Omentum)
CADM2		7.40E-07	-0.39	Heart - Left Ventricle
CADM2		1.80E-18	-0.41	Lung
CADM2	rs17516284	1.50E-04	0.1	Nerve - Tibial
CADM2		6.70E-12	-0.29	Adipose - Subcutaneous
CADM2		1.60E-08	-0.25	Adipose - Visceral (Omentum)
CADM2		7.40E-07	-0.39	Heart - Left Ventricle
CADM2		1.70E-18	-0.41	Lung
CADM2	rs17516346	1.60E-04	0.099	Nerve - Tibial
CADM2		6.90E-12	-0.29	Adipose - Subcutaneous
CADM2		3.70E-08	-0.25	Adipose - Visceral (Omentum)
CADM2		8.70E-07	-0.39	Heart - Left Ventricle
CADM2	rs17516470	3.90E-18	-0.41	Lung
CADM2		3.60E-12	-0.29	Adipose - Subcutaneous
CADM2		2.20E-08	-0.25	Adipose - Visceral (Omentum)
CADM2		1.30E-06	-0.38	Heart - Left Ventricle
CADM2		2.00E-18	-0.41	Lung
CADM2	rs17516504	1.70E-04	0.099	Nerve - Tibial
CADM2		6.80E-12	-0.29	Adipose - Subcutaneous
CADM2		3.30E-08	-0.25	Adipose - Visceral (Omentum)
CADM2		1.30E-06	-0.38	Heart - Left Ventricle
CADM2	rs17516546	2.50E-18	-0.41	Lung
CADM2		8.30E-12	-0.29	Adipose - Subcutaneous
CADM2		3.60E-08	-0.25	Adipose - Visceral (Omentum)
CADM2		1.30E-06	-0.38	Heart - Left Ventricle
CADM2		1.80E-18	-0.41	Lung
CADM2	rs17516580	1.80E-04	0.099	Nerve - Tibial
CADM2		2.40E-12	-0.3	Adipose - Subcutaneous
CADM2		2.50E-07	-0.23	Adipose - Visceral (Omentum)
CADM2		2.30E-07	-0.41	Heart - Left Ventricle
CADM2	rs17516683	1.60E-15	-0.38	Lung
CADM2		6.80E-12	-0.29	Adipose - Subcutaneous
CADM2		1.40E-08	-0.25	Adipose - Visceral (Omentum)
CADM2		7.00E-07	-0.39	Heart - Left Ventricle
CADM2		1.60E-18	-0.41	Lung
CADM2	rs17516857	1.60E-04	0.1	Nerve - Tibial
CADM2		3.70E-10	-0.28	Adipose - Subcutaneous
CADM2		3.90E-07	-0.25	Adipose - Visceral (Omentum)
CADM2	rs17517080	3.30E-06	-0.39	Heart - Left Ventricle

CADM2		5.80E-15	-0.4	Lung
CADM2		5.80E-12	-0.29	Adipose - Subcutaneous
CADM2		5.10E-08	-0.25	Adipose - Visceral (Omentum)
CADM2		1.40E-06	-0.38	Heart - Left Ventricle
CADM2	rs17517121	1.40E-18	-0.41	Lung
CADM2		6.50E-12	-0.29	Adipose - Subcutaneous
CADM2		2.50E-08	-0.25	Adipose - Visceral (Omentum)
CADM2		7.40E-07	-0.39	Heart - Left Ventricle
CADM2		2.30E-18	-0.41	Lung
CADM2	rs17517142	1.50E-04	0.1	Nerve - Tibial
CADM2		7.30E-12	-0.29	Adipose - Subcutaneous
CADM2		1.40E-08	-0.25	Adipose - Visceral (Omentum)
CADM2		9.10E-07	-0.39	Heart - Left Ventricle
CADM2		2.00E-18	-0.41	Lung
CADM2	rs17517238	1.70E-04	0.099	Nerve - Tibial
CADM2		6.80E-12	-0.29	Adipose - Subcutaneous
CADM2		1.50E-08	-0.25	Adipose - Visceral (Omentum)
CADM2		7.40E-07	-0.39	Heart - Left Ventricle
CADM2		1.80E-18	-0.41	Lung
CADM2	rs17517273	1.60E-04	0.099	Nerve - Tibial
CADM2		6.80E-12	-0.29	Adipose - Subcutaneous
CADM2		1.50E-08	-0.25	Adipose - Visceral (Omentum)
CADM2		7.40E-07	-0.39	Heart - Left Ventricle
CADM2		1.80E-18	-0.41	Lung
CADM2	rs17518082	1.60E-04	0.099	Nerve - Tibial
CADM2		1.30E-11	-0.28	Adipose - Subcutaneous
CADM2		4.00E-08	-0.25	Adipose - Visceral (Omentum)
CADM2		5.20E-07	-0.4	Heart - Left Ventricle
CADM2		1.80E-18	-0.41	Lung
CADM2	rs17518584	5.90E-05	0.11	Nerve - Tibial
CADM2		8.30E-12	-0.29	Adipose - Subcutaneous
CADM2		2.30E-08	-0.25	Adipose - Visceral (Omentum)
CADM2		7.40E-07	-0.39	Heart - Left Ventricle
CADM2		4.50E-18	-0.41	Lung
CADM2	rs17519241	1.70E-04	0.1	Nerve - Tibial
CADM2		6.80E-12	-0.29	Adipose - Subcutaneous
CADM2		1.60E-08	-0.25	Adipose - Visceral (Omentum)
CADM2		8.00E-07	-0.39	Heart - Left Ventricle
CADM2		1.70E-18	-0.41	Lung
CADM2	rs17519262	1.70E-04	0.099	Nerve - Tibial
CADM2		6.80E-12	-0.29	Adipose - Subcutaneous
CADM2		1.40E-08	-0.25	Adipose - Visceral (Omentum)
CADM2		9.00E-07	-0.39	Heart - Left Ventricle
CADM2		1.50E-18	-0.41	Lung
CADM2	rs17519948	1.60E-04	0.1	Nerve - Tibial
CADM2		1.10E-06	-0.21	Adipose - Subcutaneous
CADM2		5.20E-05	-0.18	Adipose - Visceral (Omentum)
CADM2	rs17521052	3.70E-11	-0.33	Lung
CADM2-AS1	rs17735321	5.50E-06	1	Brain - Anterior cingulate cortex (BA24)
CADM2-AS1		3.80E-07	0.57	Brain - Caudate (basal ganglia)
CADM2-AS1	rs17879296	4.40E-07	0.56	Brain - Cerebellar Hemisphere

CADM2-AS1		1.10E-09	0.53	Brain - Cerebellum
CADM2		2.90E-09	-0.25	Adipose - Subcutaneous
CADM2		2.20E-07	-0.24	Adipose - Visceral (Omentum)
CADM2		1.50E-07	-0.4	Heart - Left Ventricle
CADM2	rs1813696	2.70E-19	-0.42	Lung
CADM2		3.00E-08	0.26	Adipose - Subcutaneous
CADM2		1.70E-05	0.22	Adipose - Visceral (Omentum)
CADM2	rs1821349	8.70E-07	0.27	Lung
CADM2	rs1821350	1.40E-05	-0.74	Testis
CADM2	rs1836131	1.30E-05	-0.74	Testis
CADM2-AS1		2.20E-06	0.54	Brain - Cerebellar Hemisphere
CADM2-AS1		1.10E-08	0.51	Brain - Cerebellum
CADM2-AS1	rs1839171	2.70E-05	0.5	Brain - Nucleus accumbens (basal ganglia)
CADM2	rs1845640	2.90E-05	-0.19	Muscle - Skeletal
CADM2		1.10E-10	-0.27	Adipose - Subcutaneous
CADM2		5.80E-08	-0.25	Adipose - Visceral (Omentum)
CADM2		3.60E-07	-0.41	Heart - Left Ventricle
CADM2	rs1865250	2.20E-17	-0.41	Lung
CADM2		1.10E-10	-0.27	Adipose - Subcutaneous
CADM2		5.80E-08	-0.25	Adipose - Visceral (Omentum)
CADM2		3.60E-07	-0.41	Heart - Left Ventricle
CADM2	rs1865251	3.20E-17	-0.4	Lung
CADM2		1.10E-10	-0.27	Adipose - Subcutaneous
CADM2		4.80E-08	-0.24	Adipose - Visceral (Omentum)
CADM2		2.10E-07	-0.4	Heart - Left Ventricle
CADM2		1.20E-14	-0.36	Lung
CADM2	rs1865252	6.30E-05	-0.15	Muscle - Skeletal
CADM2		3.20E-12	-0.29	Adipose - Subcutaneous
CADM2		4.60E-08	-0.25	Adipose - Visceral (Omentum)
CADM2		1.00E-06	-0.39	Heart - Left Ventricle
CADM2	rs1868532	8.10E-18	-0.4	Lung
CADM2	rs1868533	2.20E-07	-0.34	Lung
CADM2		2.80E-06	0.19	Adipose - Subcutaneous
CADM2		4.50E-05	0.25	Brain - Caudate (basal ganglia)
CADM2	rs1872552	3.10E-06	0.17	Muscle - Skeletal
CADM2		4.90E-05	0.17	Adipose - Subcutaneous
CADM2	rs1872553	1.20E-04	0.14	Muscle - Skeletal
CADM2		1.60E-04	0.17	Adipose - Subcutaneous
CADM2	rs1872556	8.80E-06	0.18	Muscle - Skeletal
CADM2	rs1872557	1.80E-07	1	Heart - Left Ventricle
CADM2	rs1872558	2.50E-07	1	Heart - Left Ventricle
CADM2	rs1897698	1.40E-05	-0.74	Testis
CADM2		1.10E-10	-0.27	Adipose - Subcutaneous
CADM2		5.80E-08	-0.25	Adipose - Visceral (Omentum)
CADM2		3.60E-07	-0.41	Heart - Left Ventricle
CADM2	rs1897699	2.20E-17	-0.41	Lung
CADM2		7.10E-12	-0.29	Adipose - Subcutaneous
CADM2		1.60E-08	-0.25	Adipose - Visceral (Omentum)
CADM2		7.40E-07	-0.39	Heart - Left Ventricle
CADM2		2.50E-18	-0.41	Lung
CADM2	rs1900914	1.50E-04	0.1	Nerve - Tibial

CADM2		5.70E-12	-0.29	Adipose - Subcutaneous
CADM2		5.30E-08	-0.25	Adipose - Visceral (Omentum)
CADM2		1.30E-06	-0.38	Heart - Left Ventricle
CADM2	rs1900915	3.50E-17	-0.4	Lung
CADM2		3.70E-09	-0.24	Adipose - Subcutaneous
CADM2		4.30E-07	-0.23	Adipose - Visceral (Omentum)
CADM2		5.30E-07	-0.38	Heart - Left Ventricle
CADM2	rs1900916	2.30E-19	-0.41	Lung
CADM2	rs1900917	6.30E-08	-0.35	Lung
CADM2		2.90E-11	-0.28	Adipose - Subcutaneous
CADM2		3.80E-09	-0.27	Adipose - Visceral (Omentum)
CADM2		8.60E-06	-0.36	Heart - Left Ventricle
CADM2		4.00E-18	-0.41	Lung
CADM2	rs1900918	1.30E-04	0.1	Nerve - Tibial
CADM2		9.10E-05	0.18	Adipose - Subcutaneous
CADM2		8.00E-05	0.19	Adipose - Visceral (Omentum)
CADM2	rs1901985	5.70E-07	0.64	Brain - Spinal cord (cervical c-1)
CADM2	rs191761480	5.80E-05	1.4	Heart - Left Ventricle
CADM2-AS1		4.00E-05	-0.44	Brain - Cerebellar Hemisphere
CADM2-AS1	rs1947180	5.10E-06	-0.5	Brain - Nucleus accumbens (basal ganglia)
CADM2		4.00E-11	-0.28	Adipose - Subcutaneous
CADM2		6.50E-08	-0.24	Adipose - Visceral (Omentum)
CADM2		2.00E-06	-0.37	Heart - Left Ventricle
CADM2	rs1947221	5.30E-18	-0.41	Lung
CADM2	rs1968691	9.20E-06	-0.66	Brain - Hippocampus
CADM2		1.30E-06	-0.21	Adipose - Subcutaneous
CADM2	rs1972992	3.20E-09	-0.27	Lung
CADM2		1.30E-08	-0.24	Adipose - Subcutaneous
CADM2		6.10E-08	-0.25	Adipose - Visceral (Omentum)
CADM2		1.00E-06	-0.37	Heart - Left Ventricle
CADM2	rs1972994	2.70E-19	-0.41	Lung
CADM2		2.00E-11	-0.28	Adipose - Subcutaneous
CADM2		1.50E-08	-0.26	Adipose - Visceral (Omentum)
CADM2		1.50E-07	-0.42	Heart - Left Ventricle
CADM2		7.90E-17	-0.4	Lung
CADM2	rs1991872	1.70E-04	-0.14	Muscle - Skeletal
CADM2		3.60E-10	-0.26	Adipose - Subcutaneous
CADM2		2.30E-08	-0.26	Adipose - Visceral (Omentum)
CADM2		1.30E-07	-0.4	Heart - Left Ventricle
CADM2	rs1992967	1.10E-19	-0.42	Lung
CADM2		5.90E-05	0.17	Adipose - Subcutaneous
CADM2	rs1994315	1.50E-06	0.18	Muscle - Skeletal
CADM2	rs1994316	9.70E-08	1.2	Spleen
CADM2		5.80E-06	-0.19	Adipose - Subcutaneous
CADM2		4.90E-10	-0.28	Lung
CADM2-AS1	rs200136934	7.70E-05	0.26	Nerve - Tibial
CADM2	rs200438605	9.20E-06	-0.66	Brain - Hippocampus
CADM2	rs201193246	1.90E-06	1.2	Spleen
CADM2	rs201443308	9.20E-06	-0.66	Brain - Hippocampus
CADM2	rs2017293	2.80E-07	-0.34	Lung
CADM2-AS1	rs2017676	3.00E-05	-0.47	Brain - Nucleus accumbens (basal ganglia)
CADM2	rs2018416	1.50E-05	-0.74	Testis

CADM2	rs202024985	5.20E-07	-0.28	Muscle - Skeletal
CADM2-AS1		4.50E-05	-0.46	Brain - Cerebellar Hemisphere
CADM2-AS1	rs2029051	2.30E-05	-0.5	Brain - Nucleus accumbens (basal ganglia)
CADM2-AS1	rs2029052	4.20E-05	-0.47	Brain - Cerebellar Hemisphere
CADM2-AS1	rs2029053	4.20E-05	-0.47	Brain - Cerebellar Hemisphere
CADM2		2.30E-11	-0.28	Adipose - Subcutaneous
CADM2		1.70E-07	-0.24	Adipose - Visceral (Omentum)
CADM2		2.40E-06	-0.37	Heart - Left Ventricle
CADM2		2.00E-17	-0.4	Lung
CADM2	rs2029130	1.60E-04	0.099	Nerve - Tibial
CADM2		6.90E-12	-0.29	Adipose - Subcutaneous
CADM2		1.20E-08	-0.25	Adipose - Visceral (Omentum)
CADM2		1.50E-06	-0.38	Heart - Left Ventricle
CADM2		4.50E-19	-0.42	Lung
CADM2	rs2029131	1.70E-04	0.099	Nerve - Tibial
CADM2		6.80E-12	-0.29	Adipose - Subcutaneous
CADM2		1.50E-08	-0.25	Adipose - Visceral (Omentum)
CADM2		7.40E-07	-0.39	Heart - Left Ventricle
CADM2		1.80E-18	-0.41	Lung
CADM2	rs2029132	1.60E-04	0.099	Nerve - Tibial
CADM2		6.70E-12	-0.29	Adipose - Subcutaneous
CADM2		1.50E-08	-0.25	Adipose - Visceral (Omentum)
CADM2		7.30E-07	-0.39	Heart - Left Ventricle
CADM2		1.90E-18	-0.41	Lung
CADM2	rs2029133	1.70E-04	0.099	Nerve - Tibial
CADM2		1.00E-09	-0.25	Adipose - Subcutaneous
CADM2		1.00E-07	-0.24	Adipose - Visceral (Omentum)
CADM2		2.00E-07	-0.39	Heart - Left Ventricle
CADM2	rs2029134	8.80E-20	-0.41	Lung
CADM2		1.30E-10	-0.27	Adipose - Subcutaneous
CADM2		5.80E-08	-0.25	Adipose - Visceral (Omentum)
CADM2		3.60E-07	-0.41	Heart - Left Ventricle
CADM2	rs2033526	3.20E-17	-0.4	Lung
CADM2	rs2034619	9.20E-06	-0.66	Brain - Hippocampus
CADM2	rs2035560	2.30E-06	1.2	Spleen
CADM2	rs2035561	7.30E-06	0.18	Muscle - Skeletal
CADM2		1.30E-04	0.18	Adipose - Subcutaneous
CADM2	rs2035562	4.80E-06	0.18	Muscle - Skeletal
CADM2		2.30E-09	-0.25	Adipose - Subcutaneous
CADM2		2.90E-07	-0.23	Adipose - Visceral (Omentum)
CADM2		3.90E-07	-0.38	Heart - Left Ventricle
CADM2	rs2044723	1.60E-19	-0.41	Lung
CADM2		6.20E-06	-0.22	Adipose - Subcutaneous
CADM2	rs2044724	2.30E-06	-0.26	Lung
CADM2		1.40E-08	-0.23	Adipose - Subcutaneous
CADM2		6.70E-08	-0.25	Adipose - Visceral (Omentum)
CADM2		9.50E-07	-0.37	Heart - Left Ventricle
CADM2	rs2044725	3.20E-19	-0.41	Lung
CADM2		1.50E-05	-0.45	Heart - Left Ventricle
CADM2	rs2053103	8.80E-06	-0.29	Lung
CADM2	rs2053104	7.40E-05	-0.43	Heart - Left Ventricle

CADM2		2.50E-07	-0.34	Lung
CADM2	rs2053105	2.70E-07	-0.34	Lung
CADM2		7.90E-05	-0.43	Heart - Left Ventricle
CADM2	rs2053106	2.30E-07	-0.34	Lung
CADM2		1.20E-10	-0.27	Adipose - Subcutaneous
CADM2		2.80E-08	-0.25	Adipose - Visceral (Omentum)
CADM2		5.30E-07	-0.39	Heart - Left Ventricle
CADM2		4.10E-14	-0.35	Lung
CADM2	rs2053108	7.10E-05	-0.15	Muscle - Skeletal
CADM2		7.20E-05	0.16	Adipose - Subcutaneous
CADM2	rs2062431	3.60E-06	0.17	Muscle - Skeletal
CADM2		6.10E-12	-0.29	Adipose - Subcutaneous
CADM2		2.90E-08	-0.25	Adipose - Visceral (Omentum)
CADM2		6.60E-07	-0.39	Heart - Left Ventricle
CADM2	rs2069123	2.40E-17	-0.4	Lung
CADM2		3.00E-09	-0.24	Adipose - Subcutaneous
CADM2		1.10E-07	-0.24	Adipose - Visceral (Omentum)
CADM2		1.60E-07	-0.4	Heart - Left Ventricle
CADM2	rs2077839	2.40E-19	-0.41	Lung
CADM2		1.30E-10	-0.27	Adipose - Subcutaneous
CADM2		8.40E-08	-0.25	Adipose - Visceral (Omentum)
CADM2		3.30E-07	-0.42	Heart - Left Ventricle
CADM2	rs2082556	2.80E-17	-0.41	Lung
CADM2		4.70E-05	0.17	Adipose - Subcutaneous
CADM2	rs2087011	1.50E-06	0.17	Muscle - Skeletal
CADM2	rs2101343	1.20E-04	-0.94	Testis
CADM2		1.80E-05	0.18	Adipose - Subcutaneous
CADM2	rs2101344	2.90E-07	0.19	Muscle - Skeletal
CADM2		3.40E-08	0.26	Adipose - Subcutaneous
CADM2		3.30E-05	0.21	Adipose - Visceral (Omentum)
CADM2	rs2117151	1.00E-06	0.27	Lung
CADM2		2.90E-05	-0.18	Adipose - Subcutaneous
CADM2		1.70E-05	-0.2	Adipose - Visceral (Omentum)
CADM2	rs2117152	4.30E-07	-0.25	Lung
CADM2		2.70E-11	-0.28	Adipose - Subcutaneous
CADM2		4.40E-08	-0.25	Adipose - Visceral (Omentum)
CADM2		1.90E-07	-0.41	Heart - Left Ventricle
CADM2	rs2117153	1.20E-15	-0.38	Lung
CADM2	rs2122042	9.90E-05	0.21	Adipose - Subcutaneous
CADM2		6.80E-12	-0.29	Adipose - Subcutaneous
CADM2		6.80E-09	-0.26	Adipose - Visceral (Omentum)
CADM2		7.20E-07	-0.39	Heart - Left Ventricle
CADM2		3.80E-18	-0.4	Lung
CADM2	rs2122233	1.40E-04	0.1	Nerve - Tibial
CADM2		6.70E-12	-0.29	Adipose - Subcutaneous
CADM2		2.50E-08	-0.25	Adipose - Visceral (Omentum)
CADM2		7.90E-07	-0.39	Heart - Left Ventricle
CADM2	rs2122234	1.20E-18	-0.41	Lung
CADM2		5.50E-12	-0.29	Adipose - Subcutaneous
CADM2		1.20E-08	-0.25	Adipose - Visceral (Omentum)
CADM2		8.50E-07	-0.39	Heart - Left Ventricle
CADM2	rs2122235	2.00E-18	-0.41	Lung

CADM2		1.70E-04	0.099	Nerve - Tibial
CADM2		1.40E-05	-0.19	Adipose - Subcutaneous
CADM2	rs2122236	3.60E-09	-0.28	Lung
CADM2	rs2163970	3.90E-05	-0.68	Testis
CADM2		4.10E-11	-0.27	Adipose - Subcutaneous
CADM2		3.30E-06	-0.21	Adipose - Visceral (Omentum)
CADM2		1.50E-06	-0.37	Heart - Left Ventricle
CADM2		2.90E-12	-0.33	Lung
CADM2	rs2163971	1.50E-04	-0.14	Muscle - Skeletal
CADM2	rs2163972	5.80E-05	0.17	Adipose - Subcutaneous
CADM2		1.00E-11	-0.29	Adipose - Subcutaneous
CADM2		1.40E-08	-0.25	Adipose - Visceral (Omentum)
CADM2		9.00E-07	-0.39	Heart - Left Ventricle
CADM2		1.20E-18	-0.41	Lung
CADM2	rs2167043	1.20E-04	0.1	Nerve - Tibial
CADM2		7.00E-12	-0.29	Adipose - Subcutaneous
CADM2		1.40E-08	-0.25	Adipose - Visceral (Omentum)
CADM2		7.10E-07	-0.39	Heart - Left Ventricle
CADM2		1.50E-18	-0.41	Lung
CADM2	rs2167044	1.60E-04	0.099	Nerve - Tibial
CADM2		6.80E-12	-0.29	Adipose - Subcutaneous
CADM2		1.50E-08	-0.25	Adipose - Visceral (Omentum)
CADM2		7.40E-07	-0.39	Heart - Left Ventricle
CADM2		1.80E-18	-0.41	Lung
CADM2	rs2167045	1.60E-04	0.099	Nerve - Tibial
CADM2		9.00E-12	-0.29	Adipose - Subcutaneous
CADM2		1.00E-08	-0.26	Adipose - Visceral (Omentum)
CADM2		6.90E-07	-0.39	Heart - Left Ventricle
CADM2		1.80E-18	-0.41	Lung
CADM2	rs2167046	1.40E-04	0.1	Nerve - Tibial
CADM2		4.20E-12	-0.29	Adipose - Subcutaneous
CADM2		2.40E-08	-0.25	Adipose - Visceral (Omentum)
CADM2		6.90E-07	-0.39	Heart - Left Ventricle
CADM2		2.40E-18	-0.41	Lung
CADM2	rs2167047	1.50E-04	0.1	Nerve - Tibial
CADM2		2.70E-06	-0.2	Adipose - Subcutaneous
CADM2		6.10E-10	-0.28	Lung
CADM2-AS1	rs2167048	1.50E-04	0.25	Nerve - Tibial
CADM2		1.70E-05	0.16	Adipose - Subcutaneous
CADM2	rs2171140	1.50E-05	0.15	Muscle - Skeletal
CADM2		1.90E-05	0.19	Adipose - Subcutaneous
CADM2		2.70E-06	0.66	Brain - Spinal cord (cervical c-1)
CADM2	rs2171143	1.00E-05	0.17	Muscle - Skeletal
CADM2		7.90E-05	-0.43	Heart - Left Ventricle
CADM2	rs2196095	2.30E-07	-0.34	Lung
CADM2		5.80E-07	0.26	Adipose - Subcutaneous
CADM2		1.80E-05	0.24	Adipose - Visceral (Omentum)
CADM2	rs2196096	4.70E-09	0.34	Lung
CADM2		5.80E-07	0.26	Adipose - Subcutaneous
CADM2		1.80E-05	0.24	Adipose - Visceral (Omentum)
CADM2	rs2196097	4.70E-09	0.34	Lung
CADM2	rs2196098	9.80E-08	0.27	Adipose - Subcutaneous

CADM2		1.80E-05	0.24	Adipose - Visceral (Omentum)
CADM2		1.30E-08	0.33	Lung
CADM2		1.90E-10	-0.27	Adipose - Subcutaneous
CADM2		5.10E-08	-0.24	Adipose - Visceral (Omentum)
CADM2		1.70E-07	-0.41	Heart - Left Ventricle
CADM2		1.20E-14	-0.36	Lung
CADM2	rs2196099	4.10E-05	-0.15	Muscle - Skeletal
CADM2		1.00E-04	0.16	Adipose - Subcutaneous
CADM2	rs2200461	1.30E-06	0.18	Muscle - Skeletal
CADM2		7.30E-05	0.16	Adipose - Subcutaneous
CADM2	rs2200464	5.00E-05	0.14	Muscle - Skeletal
CADM2	rs2200467	2.00E-05	1.1	Spleen
CADM2		1.80E-10	-0.27	Adipose - Subcutaneous
CADM2		4.70E-08	-0.24	Adipose - Visceral (Omentum)
CADM2		2.10E-07	-0.4	Heart - Left Ventricle
CADM2		1.00E-14	-0.36	Lung
CADM2	rs2217720	4.40E-05	-0.15	Muscle - Skeletal
CADM2		8.30E-05	0.16	Adipose - Subcutaneous
CADM2	rs2220243	2.00E-06	0.17	Muscle - Skeletal
CADM2-AS1		1.30E-05	0.58	Brain - Caudate (basal ganglia)
CADM2-AS1		7.50E-08	0.66	Brain - Cerebellar Hemisphere
CADM2-AS1		1.80E-07	0.52	Brain - Cerebellum
CADM2-AS1		1.50E-05	0.51	Brain - Cortex
CADM2-AS1		2.80E-06	0.59	Brain - Frontal Cortex (BA9)
CADM2-AS1	rs2324937	2.20E-06	0.57	Brain - Nucleus accumbens (basal ganglia)
CADM2-AS1		1.20E-05	0.57	Brain - Caudate (basal ganglia)
CADM2-AS1		8.70E-07	0.61	Brain - Cerebellar Hemisphere
CADM2-AS1		2.10E-07	0.51	Brain - Cerebellum
CADM2-AS1		5.80E-06	0.58	Brain - Frontal Cortex (BA9)
CADM2-AS1	rs2324938	1.30E-05	0.52	Brain - Nucleus accumbens (basal ganglia)
CADM2-AS1		9.90E-08	0.65	Brain - Cerebellar Hemisphere
CADM2-AS1		1.60E-08	0.55	Brain - Cerebellum
CADM2-AS1		1.50E-06	0.61	Brain - Frontal Cortex (BA9)
CADM2-AS1	rs2324939	2.90E-06	0.55	Brain - Nucleus accumbens (basal ganglia)
CADM2-AS1		3.70E-05	0.55	Brain - Caudate (basal ganglia)
CADM2-AS1		1.60E-07	0.65	Brain - Cerebellar Hemisphere
CADM2-AS1		4.30E-08	0.53	Brain - Cerebellum
CADM2-AS1		2.00E-05	0.49	Brain - Cortex
CADM2-AS1		1.10E-06	0.61	Brain - Frontal Cortex (BA9)
CADM2-AS1	rs2324977	1.30E-06	0.57	Brain - Nucleus accumbens (basal ganglia)
CADM2-AS1		8.90E-06	0.59	Brain - Caudate (basal ganglia)
CADM2-AS1		5.10E-07	0.63	Brain - Cerebellar Hemisphere
CADM2-AS1		1.90E-07	0.52	Brain - Cerebellum
CADM2-AS1		2.60E-05	0.49	Brain - Cortex
CADM2-AS1		3.30E-06	0.6	Brain - Frontal Cortex (BA9)
CADM2-AS1	rs2324981	2.60E-06	0.57	Brain - Nucleus accumbens (basal ganglia)
CADM2-AS1		3.40E-05	0.54	Brain - Cerebellar Hemisphere
CADM2-AS1	rs2324983	2.10E-06	0.47	Brain - Cerebellum

CADM2-AS1		4.70E-05	0.47	Brain - Cortex
CADM2-AS1		3.40E-05	0.54	Brain - Cerebellar Hemisphere
CADM2-AS1		2.10E-06	0.47	Brain - Cerebellum
CADM2-AS1	rs2324984	4.70E-05	0.47	Brain - Cortex
CADM2-AS1		3.40E-05	0.54	Brain - Cerebellar Hemisphere
CADM2-AS1		2.10E-06	0.47	Brain - Cerebellum
CADM2-AS1	rs2324985	4.70E-05	0.47	Brain - Cortex
CADM2-AS1		1.60E-07	0.65	Brain - Cerebellar Hemisphere
CADM2-AS1		8.00E-08	0.51	Brain - Cerebellum
CADM2-AS1		2.80E-06	0.52	Brain - Cortex
CADM2-AS1		1.70E-06	0.6	Brain - Frontal Cortex (BA9)
CADM2-AS1	rs2325005	9.10E-07	0.58	Brain - Nucleus accumbens (basal ganglia)
CADM2-AS1		5.50E-07	0.6	Brain - Caudate (basal ganglia)
CADM2-AS1		1.60E-10	0.71	Brain - Cerebellar Hemisphere
CADM2-AS1		3.50E-11	0.62	Brain - Cerebellum
CADM2-AS1		3.20E-06	0.57	Brain - Frontal Cortex (BA9)
CADM2-AS1	rs2325006	5.20E-08	0.63	Brain - Nucleus accumbens (basal ganglia)
CADM2-AS1		2.80E-05	0.55	Brain - Caudate (basal ganglia)
CADM2-AS1		1.20E-08	0.69	Brain - Cerebellar Hemisphere
CADM2-AS1		7.50E-08	0.51	Brain - Cerebellum
CADM2-AS1		5.20E-06	0.51	Brain - Cortex
CADM2-AS1		5.20E-07	0.62	Brain - Frontal Cortex (BA9)
CADM2-AS1	rs2325007	8.60E-07	0.58	Brain - Nucleus accumbens (basal ganglia)
CADM2-AS1		8.90E-06	0.58	Brain - Caudate (basal ganglia)
CADM2-AS1	rs2325035	3.60E-06	0.5	Brain - Cerebellum
CADM2-AS1		2.20E-06	0.55	Brain - Caudate (basal ganglia)
CADM2-AS1		4.50E-05	0.46	Brain - Cerebellar Hemisphere
CADM2-AS1		1.60E-07	0.48	Brain - Cerebellum
CADM2-AS1	rs2325036	3.40E-06	0.52	Brain - Nucleus accumbens (basal ganglia)
CADM2-AS1		1.30E-06	0.54	Brain - Cerebellar Hemisphere
CADM2-AS1		4.30E-08	0.5	Brain - Cerebellum
CADM2-AS1	rs2325037	1.80E-05	0.5	Brain - Nucleus accumbens (basal ganglia)
CADM2	rs2325773	4.70E-05	0.21	Brain - Cerebellum
CADM2		1.50E-04	0.17	Adipose - Subcutaneous
CADM2	rs2326123	8.20E-06	0.18	Muscle - Skeletal
CADM2	rs2326125	1.80E-09	1.1	Spleen
CADM2	rs2326126	4.60E-06	0.17	Muscle - Skeletal
CADM2	rs2326128	9.50E-07	0.18	Muscle - Skeletal
CADM2	rs2326267	5.80E-05	-0.28	Lung
CADM2		4.40E-10	-0.26	Adipose - Subcutaneous
CADM2		1.90E-07	-0.24	Adipose - Visceral (Omentum)
CADM2		2.00E-07	-0.4	Heart - Left Ventricle
CADM2		3.00E-14	-0.36	Lung
CADM2	rs2326309	1.00E-04	-0.15	Muscle - Skeletal
CADM2		3.80E-10	-0.26	Adipose - Subcutaneous
CADM2		1.00E-07	-0.24	Adipose - Visceral (Omentum)
CADM2		2.00E-07	-0.4	Heart - Left Ventricle
CADM2	rs2326310	2.90E-14	-0.36	Lung

CADM2		7.70E-05	-0.15	Muscle - Skeletal
CADM2		9.80E-09	0.27	Adipose - Subcutaneous
CADM2		2.30E-05	0.22	Adipose - Visceral (Omentum)
CADM2	rs2326313	1.30E-06	0.27	Lung
CADM2	rs2326315	6.30E-08	-0.35	Lung
CADM2		6.20E-11	-0.28	Adipose - Subcutaneous
CADM2		1.10E-08	-0.27	Adipose - Visceral (Omentum)
CADM2		1.70E-05	-0.35	Heart - Left Ventricle
CADM2	rs2326316	9.30E-17	-0.4	Lung
CADM2		1.60E-12	-0.29	Adipose - Subcutaneous
CADM2		1.10E-08	-0.26	Adipose - Visceral (Omentum)
CADM2		9.00E-07	-0.38	Heart - Left Ventricle
CADM2	rs2326317	3.20E-19	-0.42	Lung
CADM2		8.50E-13	-0.3	Adipose - Subcutaneous
CADM2		8.90E-09	-0.26	Adipose - Visceral (Omentum)
CADM2		1.30E-06	-0.38	Heart - Left Ventricle
CADM2	rs2326318	2.40E-18	-0.41	Lung
CADM2		1.00E-09	-0.25	Adipose - Subcutaneous
CADM2		1.00E-07	-0.24	Adipose - Visceral (Omentum)
CADM2		2.00E-07	-0.39	Heart - Left Ventricle
CADM2	rs2326319	8.80E-20	-0.41	Lung
CADM2		7.30E-12	-0.28	Adipose - Subcutaneous
CADM2		1.10E-08	-0.25	Adipose - Visceral (Omentum)
CADM2		1.60E-06	-0.38	Heart - Left Ventricle
CADM2	rs2326320	1.40E-17	-0.4	Lung
CADM2	rs2326381	6.30E-08	-0.35	Lung
CADM2-AS1		7.90E-08	-0.64	Brain - Caudate (basal ganglia)
CADM2-AS1		4.40E-10	-0.71	Brain - Cerebellar Hemisphere
CADM2-AS1		1.10E-10	-0.61	Brain - Cerebellum
CADM2-AS1		4.10E-06	-0.56	Brain - Frontal Cortex (BA9)
CADM2-AS1		2.30E-05	-0.56	Brain - Hippocampus
CADM2-AS1		3.70E-07	-0.6	Brain - Nucleus accumbens (basal ganglia)
CADM2-AS1	rs28522091	1.30E-04	-0.25	Nerve - Tibial
CADM2-AS1	rs28628150	8.00E-05	-0.38	Nerve - Tibial
CADM2		7.70E-06	0.2	Adipose - Subcutaneous
CADM2		1.10E-08	0.71	Brain - Spinal cord (cervical c-1)
CADM2	rs28693686	1.80E-06	0.19	Muscle - Skeletal
CADM2		3.40E-06	-0.21	Adipose - Subcutaneous
CADM2		7.70E-08	-0.26	Adipose - Visceral (Omentum)
CADM2		1.80E-05	-0.22	Breast - Mammary Tissue
CADM2	rs28732378	3.00E-10	-0.32	Lung
CADM2-AS1	rs2875443	2.70E-05	-0.27	Nerve - Tibial
CADM2-AS1		1.60E-06	0.56	Brain - Caudate (basal ganglia)
CADM2-AS1		4.50E-05	0.46	Brain - Cerebellar Hemisphere
CADM2-AS1		1.60E-07	0.48	Brain - Cerebellum
CADM2-AS1	rs2875529	3.40E-06	0.52	Brain - Nucleus accumbens (basal ganglia)
CADM2	rs2875889	3.90E-06	-0.33	Lung
CADM2		3.00E-09	-0.24	Adipose - Subcutaneous
CADM2		1.10E-07	-0.24	Adipose - Visceral (Omentum)
CADM2		1.60E-07	-0.4	Heart - Left Ventricle
CADM2	rs2875907	2.40E-19	-0.41	Lung

CADM2		6.80E-12	-0.29	Adipose - Subcutaneous
CADM2		1.50E-08	-0.25	Adipose - Visceral (Omentum)
CADM2		7.40E-07	-0.39	Heart - Left Ventricle
CADM2		1.80E-18	-0.41	Lung
CADM2	rs2875908	1.60E-04	0.099	Nerve - Tibial
CADM2		7.10E-12	-0.29	Adipose - Subcutaneous
CADM2		1.50E-08	-0.25	Adipose - Visceral (Omentum)
CADM2		7.30E-07	-0.39	Heart - Left Ventricle
CADM2		1.80E-18	-0.41	Lung
CADM2	rs3086190	1.70E-04	0.099	Nerve - Tibial
CADM2-AS1	rs3158	6.50E-05	0.41	Brain - Cerebellum
CADM2	rs3159	1.10E-05	0.29	Adipose - Subcutaneous
CADM2	rs34049483	1.90E-07	0.66	Brain - Spinal cord (cervical c-1)
CADM2		1.10E-09	-0.25	Adipose - Subcutaneous
CADM2		1.70E-07	-0.23	Adipose - Visceral (Omentum)
CADM2		5.70E-08	-0.41	Heart - Left Ventricle
CADM2	rs34133544	9.50E-18	-0.39	Lung
CADM2-AS1		6.50E-07	0.63	Brain - Caudate (basal ganglia)
CADM2-AS1		2.60E-07	0.65	Brain - Cerebellar Hemisphere
CADM2-AS1		5.80E-07	0.5	Brain - Cerebellum
CADM2-AS1		2.60E-06	0.65	Brain - Frontal Cortex (BA9)
CADM2-AS1	rs34184235	5.00E-06	0.61	Brain - Nucleus accumbens (basal ganglia)
CADM2		8.20E-05	0.15	Adipose - Subcutaneous
CADM2	rs34201102	7.00E-06	0.16	Muscle - Skeletal
CADM2-AS1	rs34208095	6.00E-05	-0.29	Nerve - Tibial
CADM2-AS1		6.10E-07	0.62	Brain - Caudate (basal ganglia)
CADM2-AS1		2.50E-10	0.74	Brain - Cerebellar Hemisphere
CADM2-AS1		1.80E-12	0.65	Brain - Cerebellum
CADM2-AS1		1.50E-06	0.56	Brain - Cortex
CADM2-AS1		5.40E-07	0.64	Brain - Frontal Cortex (BA9)
CADM2-AS1		1.60E-06	0.67	Brain - Hippocampus
CADM2-AS1		9.70E-09	0.68	Brain - Nucleus accumbens (basal ganglia)
CADM2-AS1	rs34289000	1.80E-05	0.57	Brain - Putamen (basal ganglia)
CADM2-AS1	rs34325617	5.20E-05	-0.29	Nerve - Tibial
CADM2-AS1		3.30E-07	0.61	Brain - Caudate (basal ganglia)
CADM2-AS1		1.00E-09	0.69	Brain - Cerebellar Hemisphere
CADM2-AS1		2.90E-10	0.59	Brain - Cerebellum
CADM2-AS1		7.60E-07	0.58	Brain - Nucleus accumbens (basal ganglia)
CADM2-AS1	rs34420989	6.40E-05	0.26	Nerve - Tibial
CADM2-AS1	rs34436457	6.00E-05	-0.29	Nerve - Tibial
CADM2		1.70E-11	-0.28	Adipose - Subcutaneous
CADM2		2.80E-08	-0.25	Adipose - Visceral (Omentum)
CADM2		1.10E-06	-0.38	Heart - Left Ventricle
CADM2	rs34467301	5.20E-18	-0.4	Lung
CADM2-AS1	rs34482501	1.10E-04	-0.45	Nerve - Tibial
CADM2		2.50E-11	-0.28	Adipose - Subcutaneous
CADM2		1.90E-07	-0.24	Adipose - Visceral (Omentum)
CADM2		1.20E-06	-0.38	Heart - Left Ventricle
CADM2	rs34495106	2.30E-17	-0.4	Lung

CADM2-AS1	rs34647294	8.20E-05	-0.28	Nerve - Tibial
CADM2	rs34664500	1.20E-06	1.2	Spleen
CADM2	rs34672884	7.80E-06	0.17	Muscle - Skeletal
CADM2-AS1		4.40E-06	0.58	Brain - Caudate (basal ganglia)
CADM2-AS1		1.50E-09	0.72	Brain - Cerebellar Hemisphere
CADM2-AS1		1.80E-12	0.67	Brain - Cerebellum
CADM2-AS1		1.80E-05	0.51	Brain - Cortex
CADM2-AS1		1.40E-06	0.62	Brain - Frontal Cortex (BA9)
CADM2-AS1		5.80E-06	0.65	Brain - Hippocampus
CADM2-AS1		3.80E-08	0.67	Brain - Nucleus accumbens (basal ganglia)
CADM2-AS1	rs34754035	1.70E-05	0.57	Brain - Putamen (basal ganglia)
CADM2-AS1	rs34771604	3.50E-05	-0.39	Nerve - Tibial
CADM2-AS1		1.10E-06	0.59	Brain - Caudate (basal ganglia)
CADM2-AS1		2.90E-08	0.65	Brain - Cerebellar Hemisphere
CADM2-AS1		1.20E-10	0.61	Brain - Cerebellum
CADM2-AS1		1.60E-05	0.57	Brain - Hippocampus
CADM2-AS1	rs34803122	2.40E-06	0.55	Brain - Nucleus accumbens (basal ganglia)
CADM2-AS1		2.40E-06	0.58	Brain - Caudate (basal ganglia)
CADM2-AS1		4.90E-08	0.64	Brain - Cerebellar Hemisphere
CADM2-AS1		1.00E-09	0.55	Brain - Cerebellum
CADM2-AS1		1.30E-05	0.49	Brain - Cortex
CADM2-AS1		3.50E-06	0.62	Brain - Frontal Cortex (BA9)
CADM2-AS1		1.30E-05	0.59	Brain - Hippocampus
CADM2-AS1	rs34974070	1.90E-06	0.59	Brain - Nucleus accumbens (basal ganglia)
CADM2-AS1		1.40E-07	0.65	Brain - Caudate (basal ganglia)
CADM2-AS1		5.10E-11	0.75	Brain - Cerebellar Hemisphere
CADM2-AS1		6.70E-12	0.63	Brain - Cerebellum
CADM2-AS1		3.50E-07	0.58	Brain - Cortex
CADM2-AS1		1.50E-07	0.65	Brain - Frontal Cortex (BA9)
CADM2-AS1		1.60E-06	0.67	Brain - Hippocampus
CADM2-AS1		1.80E-09	0.71	Brain - Nucleus accumbens (basal ganglia)
CADM2-AS1	rs35012249	9.90E-05	0.25	Nerve - Tibial
CADM2-AS1		1.00E-07	0.67	Brain - Caudate (basal ganglia)
CADM2-AS1		1.60E-10	0.76	Brain - Cerebellar Hemisphere
CADM2-AS1		8.30E-11	0.63	Brain - Cerebellum
CADM2-AS1		1.10E-05	0.55	Brain - Cortex
CADM2-AS1		1.70E-06	0.64	Brain - Frontal Cortex (BA9)
CADM2-AS1		3.00E-06	0.65	Brain - Hippocampus
CADM2-AS1		2.40E-08	0.68	Brain - Nucleus accumbens (basal ganglia)
CADM2-AS1	rs35177461	1.80E-05	0.58	Brain - Putamen (basal ganglia)
CADM2-AS1		6.10E-07	0.62	Brain - Caudate (basal ganglia)
CADM2-AS1		2.50E-10	0.74	Brain - Cerebellar Hemisphere
CADM2-AS1		1.80E-12	0.65	Brain - Cerebellum
CADM2-AS1		1.50E-06	0.56	Brain - Cortex
CADM2-AS1		5.40E-07	0.64	Brain - Frontal Cortex (BA9)
CADM2-AS1	rs35242846	1.60E-06	0.67	Brain - Hippocampus

CADM2-AS1		9.70E-09	0.68	Brain - Nucleus accumbens (basal ganglia)
CADM2-AS1		1.80E-05	0.57	Brain - Putamen (basal ganglia)
CADM2-AS1		1.40E-07	0.65	Brain - Caudate (basal ganglia)
CADM2-AS1		5.10E-11	0.75	Brain - Cerebellar Hemisphere
CADM2-AS1		4.30E-12	0.64	Brain - Cerebellum
CADM2-AS1		2.70E-07	0.59	Brain - Cortex
CADM2-AS1		1.50E-07	0.65	Brain - Frontal Cortex (BA9)
CADM2-AS1		9.60E-07	0.68	Brain - Hippocampus
CADM2-AS1		1.80E-09	0.71	Brain - Nucleus accumbens (basal ganglia)
CADM2-AS1	rs35259978	1.10E-04	0.25	Nerve - Tibial
CADM2		3.00E-09	-0.24	Adipose - Subcutaneous
CADM2		1.10E-07	-0.24	Adipose - Visceral (Omentum)
CADM2		1.60E-07	-0.4	Heart - Left Ventricle
CADM2	rs35291972	2.40E-19	-0.41	Lung
CADM2		9.20E-08	0.28	Adipose - Subcutaneous
CADM2		8.60E-06	0.25	Adipose - Visceral (Omentum)
CADM2	rs35344466	8.30E-09	0.34	Lung
CADM2-AS1	rs35356576	7.70E-05	-0.37	Nerve - Tibial
CADM2		3.30E-08	0.29	Adipose - Subcutaneous
CADM2		3.40E-05	0.24	Adipose - Visceral (Omentum)
CADM2	rs35376282	1.90E-08	0.34	Lung
CADM2		5.90E-12	-0.29	Adipose - Subcutaneous
CADM2		6.00E-08	-0.24	Adipose - Visceral (Omentum)
CADM2		5.30E-07	-0.4	Heart - Left Ventricle
CADM2		1.10E-18	-0.41	Lung
CADM2	rs35438712	1.50E-04	0.1	Nerve - Tibial
CADM2-AS1		4.10E-07	0.6	Brain - Caudate (basal ganglia)
CADM2-AS1		8.20E-09	0.65	Brain - Cerebellar Hemisphere
CADM2-AS1		3.10E-10	0.59	Brain - Cerebellum
CADM2-AS1		1.70E-05	0.52	Brain - Frontal Cortex (BA9)
CADM2-AS1		1.40E-05	0.55	Brain - Hippocampus
CADM2-AS1	rs35498642	3.20E-06	0.53	Brain - Nucleus accumbens (basal ganglia)
CADM2		2.90E-11	-0.28	Adipose - Subcutaneous
CADM2		1.10E-08	-0.26	Adipose - Visceral (Omentum)
CADM2		6.50E-07	-0.39	Heart - Left Ventricle
CADM2		7.80E-18	-0.4	Lung
CADM2	rs35608699	1.80E-04	0.099	Nerve - Tibial
CADM2-AS1	rs35614120	1.30E-05	-0.41	Nerve - Tibial
CADM2		5.10E-07	-0.24	Lung
CADM2-AS1	rs35623690	1.50E-04	0.25	Nerve - Tibial
CADM2-AS1		4.30E-06	0.65	Brain - Caudate (basal ganglia)
CADM2-AS1	rs35679342	3.90E-06	0.55	Brain - Cerebellum
CADM2		9.50E-12	-0.29	Adipose - Subcutaneous
CADM2		2.50E-08	-0.25	Adipose - Visceral (Omentum)
CADM2		1.60E-06	-0.38	Heart - Left Ventricle
CADM2	rs35701422	2.10E-18	-0.41	Lung
CADM2	rs35736156	5.10E-06	0.64	Brain - Spinal cord (cervical c-1)
CADM2		4.40E-12	-0.29	Adipose - Subcutaneous
CADM2		1.50E-08	-0.25	Adipose - Visceral (Omentum)
CADM2	rs35738543	6.40E-07	-0.39	Heart - Left Ventricle

CADM2		3.90E-18	-0.4	Lung
CADM2-AS1		1.60E-06	0.6	Brain - Caudate (basal ganglia)
CADM2-AS1		2.50E-10	0.74	Brain - Cerebellar Hemisphere
CADM2-AS1		1.80E-12	0.65	Brain - Cerebellum
CADM2-AS1		1.50E-06	0.56	Brain - Cortex
CADM2-AS1		5.40E-07	0.64	Brain - Frontal Cortex (BA9)
CADM2-AS1		1.90E-06	0.66	Brain - Hippocampus
CADM2-AS1		9.70E-09	0.68	Brain - Nucleus accumbens (basal ganglia)
CADM2-AS1	rs35772683	1.80E-05	0.57	Brain - Putamen (basal ganglia)
CADM2		1.10E-10	-0.27	Adipose - Subcutaneous
CADM2		4.50E-08	-0.25	Adipose - Visceral (Omentum)
CADM2		3.50E-07	-0.41	Heart - Left Ventricle
CADM2	rs35827242	3.40E-17	-0.4	Lung
CADM2		1.20E-09	-0.25	Adipose - Subcutaneous
CADM2		8.10E-08	-0.24	Adipose - Visceral (Omentum)
CADM2		3.50E-07	-0.39	Heart - Left Ventricle
CADM2	rs35875166	8.50E-20	-0.42	Lung
CADM2-AS1		2.40E-06	0.58	Brain - Caudate (basal ganglia)
CADM2-AS1		4.90E-08	0.64	Brain - Cerebellar Hemisphere
CADM2-AS1		1.00E-09	0.55	Brain - Cerebellum
CADM2-AS1		5.80E-06	0.51	Brain - Cortex
CADM2-AS1		3.50E-06	0.62	Brain - Frontal Cortex (BA9)
CADM2-AS1		1.30E-05	0.59	Brain - Hippocampus
CADM2-AS1	rs35885688	1.90E-06	0.59	Brain - Nucleus accumbens (basal ganglia)
CADM2		3.60E-10	-0.26	Adipose - Subcutaneous
CADM2		4.40E-08	-0.25	Adipose - Visceral (Omentum)
CADM2		3.30E-07	-0.42	Heart - Left Ventricle
CADM2	rs35894540	2.70E-17	-0.4	Lung
CADM2	rs36041727	1.10E-04	0.31	Adipose - Subcutaneous
CADM2-AS1	rs36050412	6.30E-05	-0.29	Nerve - Tibial
CADM2-AS1		1.90E-07	0.63	Brain - Caudate (basal ganglia)
CADM2-AS1		5.60E-11	0.74	Brain - Cerebellar Hemisphere
CADM2-AS1		1.90E-11	0.63	Brain - Cerebellum
CADM2-AS1		5.40E-06	0.56	Brain - Frontal Cortex (BA9)
CADM2-AS1		8.40E-06	0.58	Brain - Hippocampus
CADM2-AS1		7.30E-08	0.64	Brain - Nucleus accumbens (basal ganglia)
CADM2-AS1	rs36080783	7.00E-05	0.26	Nerve - Tibial
CADM2		4.80E-06	0.19	Adipose - Subcutaneous
CADM2		3.50E-05	0.25	Brain - Caudate (basal ganglia)
CADM2	rs369165214	5.60E-06	0.16	Muscle - Skeletal
CADM2	rs369199567	3.00E-05	-0.18	Muscle - Skeletal
CADM2		9.00E-06	0.62	Brain - Spinal cord (cervical c-1)
CADM2	rs370387989	3.30E-06	0.17	Muscle - Skeletal
CADM2	rs373274293	4.80E-05	0.68	Spleen
CADM2	rs373387579	1.60E-05	-0.19	Muscle - Skeletal
CADM2		1.20E-12	-0.3	Adipose - Subcutaneous
CADM2		2.10E-08	-0.25	Adipose - Visceral (Omentum)
CADM2		3.60E-06	-0.36	Heart - Left Ventricle
CADM2	rs376830009	4.10E-18	-0.41	Lung

CADM2		1.60E-04	0.1	Nerve - Tibial
CADM2		6.20E-05	0.18	Adipose - Subcutaneous
CADM2	rs378157	1.90E-07	0.21	Muscle - Skeletal
CADM2		7.60E-05	0.18	Adipose - Subcutaneous
CADM2		1.20E-04	0.19	Lung
CADM2	rs382210	5.60E-08	0.21	Muscle - Skeletal
CADM2	rs386397241	1.60E-04	0.15	Adipose - Subcutaneous
CADM2		3.20E-08	0.29	Adipose - Subcutaneous
CADM2		5.30E-06	0.27	Adipose - Visceral (Omentum)
CADM2	rs3887138	1.40E-09	0.36	Lung
CADM2-AS1		9.40E-06	0.57	Brain - Caudate (basal ganglia)
CADM2-AS1		1.90E-06	0.58	Brain - Cerebellar Hemisphere
CADM2-AS1		1.20E-07	0.5	Brain - Cerebellum
CADM2-AS1		2.50E-05	0.53	Brain - Frontal Cortex (BA9)
CADM2-AS1	rs3901898	2.90E-05	0.49	Brain - Nucleus accumbens (basal ganglia)
CADM2-AS1		5.30E-07	0.58	Brain - Caudate (basal ganglia)
CADM2-AS1		2.20E-05	0.48	Brain - Cerebellar Hemisphere
CADM2-AS1	rs3902500	9.20E-08	0.5	Brain - Cerebellum
CADM2-AS1		6.50E-07	0.59	Brain - Cerebellar Hemisphere
CADM2-AS1		4.20E-07	0.49	Brain - Cerebellum
CADM2-AS1		2.10E-05	0.53	Brain - Frontal Cortex (BA9)
CADM2-AS1	rs3906942	1.60E-05	0.51	Brain - Nucleus accumbens (basal ganglia)
CADM2-AS1		3.80E-05	0.52	Brain - Caudate (basal ganglia)
CADM2-AS1		5.90E-07	0.59	Brain - Cerebellar Hemisphere
CADM2-AS1		7.20E-08	0.51	Brain - Cerebellum
CADM2-AS1		1.70E-05	0.53	Brain - Frontal Cortex (BA9)
CADM2-AS1	rs3911061	1.80E-05	0.5	Brain - Nucleus accumbens (basal ganglia)
CADM2-AS1		1.30E-06	0.56	Brain - Caudate (basal ganglia)
CADM2-AS1		3.60E-05	0.49	Brain - Cerebellar Hemisphere
CADM2-AS1		2.80E-08	0.52	Brain - Cerebellum
CADM2-AS1	rs3911063	1.10E-05	0.3	Nerve - Tibial
CADM2-AS1		7.00E-07	0.57	Brain - Caudate (basal ganglia)
CADM2-AS1		2.40E-05	0.48	Brain - Cerebellar Hemisphere
CADM2-AS1		4.70E-08	0.5	Brain - Cerebellum
CADM2-AS1	rs3916054	7.90E-05	0.26	Nerve - Tibial
CADM2	rs3925547	5.90E-05	0.5	Heart - Left Ventricle
CADM2-AS1		2.00E-06	0.53	Brain - Cerebellar Hemisphere
CADM2-AS1	rs3925704	4.70E-08	0.49	Brain - Cerebellum
CADM2-AS1		4.40E-05	0.53	Brain - Caudate (basal ganglia)
CADM2-AS1		2.00E-08	0.67	Brain - Cerebellar Hemisphere
CADM2-AS1		5.80E-08	0.51	Brain - Cerebellum
CADM2-AS1		8.40E-06	0.51	Brain - Cortex
CADM2-AS1		1.50E-05	0.55	Brain - Frontal Cortex (BA9)
CADM2-AS1	rs3930071	9.60E-06	0.53	Brain - Nucleus accumbens (basal ganglia)
CADM2	rs395807	2.80E-05	0.43	Heart - Left Ventricle
CADM2		1.00E-07	-0.25	Adipose - Subcutaneous
CADM2		8.60E-05	-0.2	Adipose - Visceral (Omentum)
CADM2	rs397688298	1.50E-06	-0.42	Heart - Left Ventricle

CADM2		1.70E-13	-0.38	Lung
CADM2		1.10E-04	0.11	Nerve - Tibial
CADM2		2.60E-12	-0.3	Adipose - Subcutaneous
CADM2		1.50E-08	-0.26	Adipose - Visceral (Omentum)
CADM2		1.40E-06	-0.38	Heart - Left Ventricle
CADM2		1.90E-18	-0.41	Lung
CADM2	rs397690245	1.00E-04	0.1	Nerve - Tibial
CADM2		2.00E-08	0.26	Adipose - Subcutaneous
CADM2		1.40E-05	0.22	Adipose - Visceral (Omentum)
CADM2	rs397692753	9.40E-07	0.27	Lung
CADM2		1.50E-05	0.18	Adipose - Subcutaneous
CADM2	rs397711229	4.30E-06	0.16	Muscle - Skeletal
CADM2-AS1		7.40E-07	0.63	Brain - Caudate (basal ganglia)
CADM2-AS1		2.20E-10	0.75	Brain - Cerebellar Hemisphere
CADM2-AS1		5.60E-12	0.64	Brain - Cerebellum
CADM2-AS1		1.40E-06	0.57	Brain - Cortex
CADM2-AS1		2.30E-06	0.62	Brain - Frontal Cortex (BA9)
CADM2-AS1		1.20E-06	0.67	Brain - Hippocampus
CADM2-AS1		4.80E-08	0.66	Brain - Nucleus accumbens (basal ganglia)
CADM2-AS1	rs397728982	8.80E-06	0.61	Brain - Putamen (basal ganglia)
CADM2		1.60E-09	-0.25	Adipose - Subcutaneous
CADM2		1.00E-07	-0.24	Adipose - Visceral (Omentum)
CADM2		1.60E-07	-0.4	Heart - Left Ventricle
CADM2	rs397737840	1.10E-19	-0.41	Lung
CADM2		1.20E-07	0.25	Adipose - Subcutaneous
CADM2	rs397750995	4.70E-05	0.23	Lung
CADM2	rs397757098	1.70E-05	-0.31	Lung
CADM2	rs397766287	2.10E-07	-0.34	Lung
CADM2	rs397781416	1.10E-04	0.17	Adipose - Subcutaneous
CADM2		4.30E-05	0.16	Adipose - Subcutaneous
CADM2	rs397791212	3.20E-05	0.15	Muscle - Skeletal
CADM2		1.60E-04	0.18	Adipose - Subcutaneous
CADM2		3.70E-07	0.65	Brain - Spinal cord (cervical c-1)
CADM2	rs397796216	1.90E-05	0.18	Muscle - Skeletal
CADM2-AS1	rs397804234	1.40E-04	-0.31	Nerve - Tibial
CADM2-AS1		2.40E-06	0.54	Brain - Cerebellar Hemisphere
CADM2-AS1		1.10E-08	0.51	Brain - Cerebellum
CADM2-AS1	rs397812745	2.70E-05	0.5	Brain - Nucleus accumbens (basal ganglia)
CADM2	rs397843291	1.10E-06	1.2	Spleen
CADM2-AS1	rs397874503	2.40E-05	0.51	Brain - Cerebellar Hemisphere
CADM2		5.60E-12	-0.29	Adipose - Subcutaneous
CADM2		1.40E-08	-0.25	Adipose - Visceral (Omentum)
CADM2		6.40E-07	-0.39	Heart - Left Ventricle
CADM2	rs397875084	1.00E-17	-0.4	Lung
CADM2		3.20E-09	-0.24	Adipose - Subcutaneous
CADM2		1.10E-07	-0.24	Adipose - Visceral (Omentum)
CADM2		1.50E-07	-0.4	Heart - Left Ventricle
CADM2	rs397876109	3.10E-19	-0.41	Lung
CADM2		6.80E-12	-0.29	Adipose - Subcutaneous
CADM2		1.50E-08	-0.25	Adipose - Visceral (Omentum)
CADM2	rs397876462	7.40E-07	-0.39	Heart - Left Ventricle

CADM2		1.80E-18	-0.41	Lung
CADM2		1.60E-04	0.099	Nerve - Tibial
CADM2		9.30E-12	-0.29	Adipose - Subcutaneous
CADM2		2.10E-08	-0.25	Adipose - Visceral (Omentum)
CADM2		6.70E-07	-0.39	Heart - Left Ventricle
CADM2		1.70E-18	-0.41	Lung
CADM2	rs397876568	1.30E-04	0.1	Nerve - Tibial
CADM2		7.80E-12	-0.29	Adipose - Subcutaneous
CADM2		1.50E-08	-0.26	Adipose - Visceral (Omentum)
CADM2		6.40E-07	-0.4	Heart - Left Ventricle
CADM2	rs397877117	1.20E-16	-0.39	Lung
CADM2		5.20E-06	-0.48	Heart - Left Ventricle
CADM2	rs397950878	3.20E-06	-0.3	Lung
CADM2	rs397990406	1.40E-04	0.19	Adipose - Subcutaneous
CADM2		2.40E-05	-0.45	Heart - Left Ventricle
CADM2	rs397990409	2.10E-05	-0.28	Lung
CADM2		1.10E-07	0.27	Adipose - Subcutaneous
CADM2		2.10E-05	0.24	Adipose - Visceral (Omentum)
CADM2	rs397990411	5.40E-09	0.34	Lung
CADM2		7.30E-08	0.29	Adipose - Subcutaneous
CADM2		7.50E-06	0.25	Adipose - Visceral (Omentum)
CADM2		2.10E-10	0.37	Lung
CADM2	rs397990418	5.30E-05	-0.14	Nerve - Tibial
CADM2-AS1		1.60E-07	0.62	Brain - Caudate (basal ganglia)
CADM2-AS1		6.50E-09	0.66	Brain - Cerebellar Hemisphere
CADM2-AS1		2.90E-10	0.58	Brain - Cerebellum
CADM2-AS1		1.70E-05	0.52	Brain - Frontal Cortex (BA9)
CADM2-AS1		1.10E-05	0.56	Brain - Hippocampus
CADM2-AS1	rs397990429	3.50E-06	0.53	Brain - Nucleus accumbens (basal ganglia)
CADM2		7.70E-12	-0.29	Adipose - Subcutaneous
CADM2		1.40E-08	-0.25	Adipose - Visceral (Omentum)
CADM2		7.70E-07	-0.39	Heart - Left Ventricle
CADM2		1.50E-18	-0.41	Lung
CADM2	rs398062541	1.70E-04	0.099	Nerve - Tibial
CADM2		1.90E-09	-0.26	Adipose - Subcutaneous
CADM2		6.10E-06	-0.22	Adipose - Visceral (Omentum)
CADM2		3.20E-05	-0.33	Heart - Left Ventricle
CADM2		5.10E-16	-0.39	Lung
CADM2	rs398062542	9.00E-05	0.11	Nerve - Tibial
CADM2		6.00E-11	-0.28	Adipose - Subcutaneous
CADM2		8.20E-08	-0.25	Adipose - Visceral (Omentum)
CADM2		7.10E-07	-0.4	Heart - Left Ventricle
CADM2		2.70E-17	-0.4	Lung
CADM2	rs398071877	1.00E-04	0.1	Nerve - Tibial
CADM2		2.30E-09	-0.25	Adipose - Subcutaneous
CADM2		2.80E-07	-0.23	Adipose - Visceral (Omentum)
CADM2		3.70E-07	-0.38	Heart - Left Ventricle
CADM2	rs398071879	1.40E-19	-0.41	Lung
CADM2		7.20E-10	-0.27	Adipose - Subcutaneous
CADM2		1.30E-07	-0.24	Adipose - Visceral (Omentum)
CADM2	rs398106121	8.00E-07	-0.4	Heart - Left Ventricle

CADM2		4.00E-16	-0.39	Lung
CADM2		1.80E-04	0.1	Nerve - Tibial
CADM2		8.90E-12	-0.29	Adipose - Subcutaneous
CADM2		4.00E-07	-0.24	Adipose - Visceral (Omentum)
CADM2		4.20E-07	-0.41	Heart - Left Ventricle
CADM2	rs398106122	1.30E-13	-0.37	Lung
CADM2	rs398106124	1.20E-05	0.27	Lung
CADM2		1.50E-10	-0.27	Adipose - Subcutaneous
CADM2		1.40E-08	-0.25	Adipose - Visceral (Omentum)
CADM2		2.20E-07	-0.4	Heart - Left Ventricle
CADM2	rs4053301	1.80E-14	-0.36	Lung
CADM2-AS1		4.80E-06	0.58	Brain - Caudate (basal ganglia)
CADM2-AS1		5.60E-08	0.64	Brain - Cerebellar Hemisphere
CADM2-AS1		1.10E-07	0.49	Brain - Cerebellum
CADM2-AS1		4.00E-06	0.52	Brain - Cortex
CADM2-AS1		1.40E-06	0.59	Brain - Frontal Cortex (BA9)
CADM2-AS1	rs4091990	2.70E-06	0.54	Brain - Nucleus accumbens (basal ganglia)
CADM2-AS1	rs4129298	3.00E-05	-0.47	Brain - Nucleus accumbens (basal ganglia)
CADM2		9.90E-05	0.22	Adipose - Visceral (Omentum)
CADM2	rs4129299	3.60E-06	0.28	Lung
CADM2-AS1		4.80E-06	0.58	Brain - Caudate (basal ganglia)
CADM2-AS1		5.60E-08	0.64	Brain - Cerebellar Hemisphere
CADM2-AS1		1.10E-07	0.49	Brain - Cerebellum
CADM2-AS1		4.00E-06	0.52	Brain - Cortex
CADM2-AS1		1.40E-06	0.59	Brain - Frontal Cortex (BA9)
CADM2-AS1	rs4129497	2.70E-06	0.54	Brain - Nucleus accumbens (basal ganglia)
CADM2-AS1	rs4133180	3.80E-05	-0.3	Nerve - Tibial
CADM2-AS1	rs4133181	3.80E-05	-0.3	Nerve - Tibial
CADM2		1.40E-05	0.2	Adipose - Subcutaneous
CADM2	rs420318	6.10E-07	0.2	Muscle - Skeletal
CADM2	rs421983	7.60E-07	-0.57	Brain - Spinal cord (cervical c-1)
CADM2-AS1		3.90E-05	0.53	Brain - Caudate (basal ganglia)
CADM2-AS1		2.60E-07	0.62	Brain - Cerebellar Hemisphere
CADM2-AS1		6.00E-08	0.51	Brain - Cerebellum
CADM2-AS1		8.70E-07	0.6	Brain - Frontal Cortex (BA9)
CADM2-AS1	rs4234702	5.70E-06	0.53	Brain - Nucleus accumbens (basal ganglia)
CADM2	rs4261888	2.60E-07	0.66	Brain - Spinal cord (cervical c-1)
CADM2	rs426444	6.00E-06	0.18	Muscle - Skeletal
CADM2-AS1		2.40E-06	0.58	Brain - Caudate (basal ganglia)
CADM2-AS1		4.90E-08	0.64	Brain - Cerebellar Hemisphere
CADM2-AS1		1.00E-09	0.55	Brain - Cerebellum
CADM2-AS1		5.80E-06	0.51	Brain - Cortex
CADM2-AS1		3.50E-06	0.62	Brain - Frontal Cortex (BA9)
CADM2-AS1		1.30E-05	0.59	Brain - Hippocampus
CADM2-AS1	rs4266190	1.90E-06	0.59	Brain - Nucleus accumbens (basal ganglia)
CADM2		6.00E-08	0.25	Adipose - Subcutaneous
CADM2		2.00E-05	0.21	Adipose - Visceral (Omentum)
CADM2	rs4279114	2.10E-06	0.26	Lung
CADM2-AS1	rs4287911	5.00E-06	0.61	Brain - Caudate (basal ganglia)

CADM2-AS1		8.20E-08	0.67	Brain - Cerebellar Hemisphere
CADM2-AS1		1.40E-06	0.48	Brain - Cerebellum
CADM2-AS1		2.80E-05	0.49	Brain - Cortex
CADM2-AS1		2.00E-06	0.61	Brain - Frontal Cortex (BA9)
CADM2-AS1		2.80E-06	0.57	Brain - Nucleus accumbens (basal ganglia)
CADM2-AS1		3.80E-05	0.5	Brain - Cerebellar Hemisphere
CADM2-AS1	rs4290815	3.10E-05	0.48	Brain - Nucleus accumbens (basal ganglia)
CADM2		6.80E-12	-0.29	Adipose - Subcutaneous
CADM2		1.50E-08	-0.25	Adipose - Visceral (Omentum)
CADM2		7.40E-07	-0.39	Heart - Left Ventricle
CADM2		1.80E-18	-0.41	Lung
CADM2	rs4301022	1.60E-04	0.099	Nerve - Tibial
CADM2		5.90E-05	0.16	Adipose - Subcutaneous
CADM2	rs4301023	5.10E-05	0.14	Muscle - Skeletal
CADM2-AS1		4.80E-06	0.58	Brain - Caudate (basal ganglia)
CADM2-AS1		5.60E-08	0.64	Brain - Cerebellar Hemisphere
CADM2-AS1		1.10E-07	0.49	Brain - Cerebellum
CADM2-AS1		4.00E-06	0.52	Brain - Cortex
CADM2-AS1		1.40E-06	0.59	Brain - Frontal Cortex (BA9)
CADM2-AS1	rs4302393	2.70E-06	0.54	Brain - Nucleus accumbens (basal ganglia)
CADM2-AS1		4.20E-08	0.66	Brain - Cerebellar Hemisphere
CADM2-AS1		7.90E-08	0.51	Brain - Cerebellum
CADM2-AS1		1.30E-05	0.49	Brain - Cortex
CADM2-AS1		5.00E-07	0.62	Brain - Frontal Cortex (BA9)
CADM2-AS1	rs4302394	5.30E-06	0.54	Brain - Nucleus accumbens (basal ganglia)
CADM2-AS1		1.30E-05	0.53	Brain - Caudate (basal ganglia)
CADM2-AS1		1.60E-10	0.71	Brain - Cerebellar Hemisphere
CADM2-AS1		2.00E-10	0.59	Brain - Cerebellum
CADM2-AS1		8.60E-07	0.58	Brain - Frontal Cortex (BA9)
CADM2-AS1		3.20E-05	0.54	Brain - Hippocampus
CADM2-AS1		1.20E-08	0.64	Brain - Nucleus accumbens (basal ganglia)
CADM2-AS1	rs4303861	1.20E-04	0.25	Nerve - Tibial
CADM2-AS1	rs4305433	3.10E-05	0.47	Brain - Nucleus accumbens (basal ganglia)
CADM2	rs4306872	1.20E-06	0.29	Lung
CADM2-AS1		1.30E-05	0.57	Brain - Caudate (basal ganglia)
CADM2-AS1		6.20E-08	0.66	Brain - Cerebellar Hemisphere
CADM2-AS1		1.90E-07	0.49	Brain - Cerebellum
CADM2-AS1		9.90E-06	0.5	Brain - Cortex
CADM2-AS1		1.30E-06	0.61	Brain - Frontal Cortex (BA9)
CADM2-AS1	rs4308293	4.60E-06	0.55	Brain - Nucleus accumbens (basal ganglia)
CADM2		6.40E-11	-0.27	Adipose - Subcutaneous
CADM2		3.50E-08	-0.25	Adipose - Visceral (Omentum)
CADM2		1.50E-07	-0.42	Heart - Left Ventricle
CADM2	rs4308294	8.30E-17	-0.4	Lung
CADM2-AS1	rs4312666	3.10E-05	0.47	Brain - Nucleus accumbens (basal ganglia)
CADM2-AS1	rs4320069	3.00E-05	0.5	Brain - Nucleus accumbens (basal ganglia)
CADM2-AS1	rs4321539	1.40E-06	0.53	Brain - Cerebellar Hemisphere

CADM2-AS1		6.90E-05	0.41	Brain - Cerebellum
CADM2-AS1		2.80E-06	0.53	Brain - Nucleus accumbens (basal ganglia)
CADM2-AS1		3.80E-05	0.5	Brain - Cerebellar Hemisphere
CADM2-AS1	rs4355292	3.10E-05	0.48	Brain - Nucleus accumbens (basal ganglia)
CADM2		1.30E-06	-0.21	Adipose - Subcutaneous
CADM2	rs4355295	3.50E-09	-0.27	Lung
CADM2-AS1		1.30E-05	0.57	Brain - Caudate (basal ganglia)
CADM2-AS1		6.20E-08	0.66	Brain - Cerebellar Hemisphere
CADM2-AS1		1.90E-07	0.49	Brain - Cerebellum
CADM2-AS1		9.90E-06	0.5	Brain - Cortex
CADM2-AS1		1.30E-06	0.61	Brain - Frontal Cortex (BA9)
CADM2-AS1	rs4364177	4.60E-06	0.55	Brain - Nucleus accumbens (basal ganglia)
CADM2-AS1		6.30E-08	0.65	Brain - Caudate (basal ganglia)
CADM2-AS1		1.80E-10	0.72	Brain - Cerebellar Hemisphere
CADM2-AS1		6.10E-12	0.63	Brain - Cerebellum
CADM2-AS1		4.60E-06	0.56	Brain - Frontal Cortex (BA9)
CADM2-AS1		1.10E-05	0.58	Brain - Hippocampus
CADM2-AS1		5.30E-08	0.64	Brain - Nucleus accumbens (basal ganglia)
CADM2-AS1	rs4368495	6.10E-05	0.27	Nerve - Tibial
CADM2		1.10E-05	0.17	Adipose - Subcutaneous
CADM2	rs4410457	3.90E-05	0.14	Muscle - Skeletal
CADM2-AS1	rs4416395	2.80E-05	0.49	Brain - Nucleus accumbens (basal ganglia)
CADM2	rs4422326	1.60E-04	0.13	Muscle - Skeletal
CADM2		8.20E-07	-0.21	Adipose - Subcutaneous
CADM2		2.10E-09	-0.27	Lung
CADM2-AS1	rs4426693	1.10E-04	0.26	Nerve - Tibial
CADM2		2.70E-11	-0.28	Adipose - Subcutaneous
CADM2		3.20E-07	-0.23	Adipose - Visceral (Omentum)
CADM2		6.60E-07	-0.4	Heart - Left Ventricle
CADM2		9.90E-18	-0.4	Lung
CADM2	rs4441668	9.20E-05	0.1	Nerve - Tibial
CADM2	rs4444725	1.10E-04	0.2	Adipose - Subcutaneous
CADM2	rs4452341	4.80E-06	-0.22	Lung
CADM2-AS1		6.80E-05	0.49	Brain - Cerebellar Hemisphere
CADM2-AS1	rs4456873	1.10E-05	0.51	Brain - Nucleus accumbens (basal ganglia)
CADM2-AS1		3.80E-05	0.5	Brain - Cerebellar Hemisphere
CADM2-AS1	rs4461436	3.10E-05	0.48	Brain - Nucleus accumbens (basal ganglia)
CADM2		6.80E-05	0.23	Adipose - Visceral (Omentum)
CADM2	rs4464471	2.60E-06	0.28	Lung
CADM2-AS1		3.80E-05	0.5	Brain - Cerebellar Hemisphere
CADM2-AS1	rs4476513	3.10E-05	0.48	Brain - Nucleus accumbens (basal ganglia)
CADM2-AS1	rs4482674	2.60E-05	0.5	Brain - Nucleus accumbens (basal ganglia)
CADM2-AS1		6.80E-06	0.58	Brain - Caudate (basal ganglia)
CADM2-AS1		1.20E-08	0.66	Brain - Cerebellar Hemisphere
CADM2-AS1		1.40E-07	0.5	Brain - Cerebellum
CADM2-AS1		4.70E-06	0.51	Brain - Cortex
CADM2-AS1		2.10E-06	0.58	Brain - Frontal Cortex (BA9)
CADM2-AS1	rs4488844	4.90E-07	0.59	Brain - Nucleus accumbens (basal ganglia)

CADM2-AS1		3.80E-05	0.5	Brain - Cerebellar Hemisphere
CADM2-AS1	rs4490378	3.10E-05	0.48	Brain - Nucleus accumbens (basal ganglia)
CADM2	rs4499618	3.20E-07	0.19	Muscle - Skeletal
CADM2		6.80E-12	-0.29	Adipose - Subcutaneous
CADM2		1.50E-08	-0.25	Adipose - Visceral (Omentum)
CADM2		7.40E-07	-0.39	Heart - Left Ventricle
CADM2		1.70E-18	-0.41	Lung
CADM2	rs4502590	1.60E-04	0.099	Nerve - Tibial
CADM2-AS1		3.40E-06	0.54	Brain - Cerebellar Hemisphere
CADM2-AS1		1.00E-07	0.5	Brain - Cerebellum
CADM2-AS1	rs4504176	2.60E-05	0.5	Brain - Nucleus accumbens (basal ganglia)
CADM2-AS1		8.40E-07	0.59	Brain - Caudate (basal ganglia)
CADM2-AS1		6.60E-11	0.73	Brain - Cerebellar Hemisphere
CADM2-AS1		7.90E-12	0.62	Brain - Cerebellum
CADM2-AS1		1.50E-06	0.57	Brain - Frontal Cortex (BA9)
CADM2-AS1		4.90E-06	0.6	Brain - Hippocampus
CADM2-AS1		3.20E-08	0.64	Brain - Nucleus accumbens (basal ganglia)
CADM2-AS1	rs4508773	9.90E-05	0.25	Nerve - Tibial
CADM2		2.30E-11	-0.28	Adipose - Subcutaneous
CADM2		4.30E-08	-0.24	Adipose - Visceral (Omentum)
CADM2		1.50E-06	-0.38	Heart - Left Ventricle
CADM2		4.80E-18	-0.4	Lung
CADM2	rs4508796	1.30E-04	0.1	Nerve - Tibial
CADM2		4.60E-06	-0.2	Adipose - Subcutaneous
CADM2	rs4508797	4.00E-09	-0.27	Lung
CADM2-AS1		1.60E-06	0.53	Brain - Cerebellar Hemisphere
CADM2-AS1		2.10E-05	0.42	Brain - Cerebellum
CADM2-AS1	rs4513466	3.10E-06	0.53	Brain - Nucleus accumbens (basal ganglia)
CADM2		9.70E-06	0.32	Brain - Hippocampus
CADM2	rs4516626	1.40E-04	0.13	Muscle - Skeletal
CADM2		2.30E-11	-0.28	Adipose - Subcutaneous
CADM2		2.30E-07	-0.24	Adipose - Visceral (Omentum)
CADM2		4.30E-07	-0.4	Heart - Left Ventricle
CADM2		1.20E-16	-0.4	Lung
CADM2	rs4543024	8.50E-05	0.11	Nerve - Tibial
CADM2-AS1		2.20E-06	0.54	Brain - Cerebellar Hemisphere
CADM2-AS1		1.10E-08	0.51	Brain - Cerebellum
CADM2-AS1	rs4555536	2.70E-05	0.5	Brain - Nucleus accumbens (basal ganglia)
CADM2-AS1		1.00E-05	0.56	Brain - Caudate (basal ganglia)
CADM2-AS1		1.50E-07	0.63	Brain - Cerebellar Hemisphere
CADM2-AS1		2.50E-08	0.52	Brain - Cerebellum
CADM2-AS1		2.80E-05	0.48	Brain - Cortex
CADM2-AS1		3.90E-06	0.58	Brain - Frontal Cortex (BA9)
CADM2-AS1	rs4555539	1.40E-05	0.51	Brain - Nucleus accumbens (basal ganglia)
CADM2-AS1		3.80E-05	0.5	Brain - Cerebellar Hemisphere
CADM2-AS1	rs4558768	3.10E-05	0.48	Brain - Nucleus accumbens (basal ganglia)
CADM2	rs4569672	2.60E-06	0.19	Muscle - Skeletal
CADM2-AS1	rs4575895	2.40E-06	0.58	Brain - Caudate (basal ganglia)

CADM2-AS1		4.90E-08	0.64	Brain - Cerebellar Hemisphere
CADM2-AS1		1.00E-09	0.55	Brain - Cerebellum
CADM2-AS1		5.80E-06	0.51	Brain - Cortex
CADM2-AS1		3.50E-06	0.62	Brain - Frontal Cortex (BA9)
CADM2-AS1		1.30E-05	0.59	Brain - Hippocampus
CADM2-AS1		1.90E-06	0.59	Brain - Nucleus accumbens (basal ganglia)
CADM2-AS1		2.80E-05	0.51	Brain - Cerebellar Hemisphere
CADM2-AS1	rs4597723	1.70E-05	0.5	Brain - Nucleus accumbens (basal ganglia)
CADM2-AS1		4.90E-07	0.6	Brain - Caudate (basal ganglia)
CADM2-AS1		6.40E-10	0.7	Brain - Cerebellar Hemisphere
CADM2-AS1		2.30E-10	0.59	Brain - Cerebellum
CADM2-AS1		1.40E-05	0.53	Brain - Frontal Cortex (BA9)
CADM2-AS1		2.00E-05	0.56	Brain - Hippocampus
CADM2-AS1	rs4600827	1.10E-06	0.57	Brain - Nucleus accumbens (basal ganglia)
CADM2		6.80E-12	-0.29	Adipose - Subcutaneous
CADM2		1.50E-08	-0.25	Adipose - Visceral (Omentum)
CADM2		7.40E-07	-0.39	Heart - Left Ventricle
CADM2		1.80E-18	-0.41	Lung
CADM2	rs4603966	1.60E-04	0.099	Nerve - Tibial
CADM2-AS1		2.20E-05	0.55	Brain - Cerebellar Hemisphere
CADM2-AS1	rs4607104	1.10E-06	0.48	Brain - Cerebellum
CADM2-AS1		1.90E-06	0.56	Brain - Cerebellar Hemisphere
CADM2-AS1	rs4615092	8.80E-08	0.51	Brain - Cerebellum
CADM2-AS1	rs4618235	5.90E-05	0.46	Brain - Cerebellar Hemisphere
CADM2-AS1		4.40E-06	0.54	Brain - Cerebellar Hemisphere
CADM2-AS1		1.00E-07	0.5	Brain - Cerebellum
CADM2-AS1	rs4619794	2.90E-05	0.49	Brain - Nucleus accumbens (basal ganglia)
CADM2	rs4635723	6.20E-06	0.16	Muscle - Skeletal
CADM2		5.10E-09	-0.24	Adipose - Subcutaneous
CADM2		1.10E-07	-0.24	Adipose - Visceral (Omentum)
CADM2		1.00E-07	-0.4	Heart - Left Ventricle
CADM2	rs4637303	7.10E-19	-0.41	Lung
CADM2-AS1	rs4688889	1.20E-04	-0.28	Nerve - Tibial
CADM2-AS1		4.50E-07	0.62	Brain - Caudate (basal ganglia)
CADM2-AS1		1.10E-10	0.74	Brain - Cerebellar Hemisphere
CADM2-AS1		2.60E-11	0.62	Brain - Cerebellum
CADM2-AS1		8.80E-07	0.57	Brain - Cortex
CADM2-AS1		1.90E-07	0.65	Brain - Frontal Cortex (BA9)
CADM2-AS1		4.40E-06	0.64	Brain - Hippocampus
CADM2-AS1	rs4688895	4.00E-09	0.7	Brain - Nucleus accumbens (basal ganglia)
CADM2-AS1		4.40E-05	-0.54	Brain - Caudate (basal ganglia)
CADM2-AS1		2.20E-07	-0.64	Brain - Cerebellar Hemisphere
CADM2-AS1		2.20E-07	-0.51	Brain - Cerebellum
CADM2-AS1		2.20E-06	-0.6	Brain - Frontal Cortex (BA9)
CADM2-AS1	rs4688896	4.80E-06	-0.55	Brain - Nucleus accumbens (basal ganglia)
CADM2		2.40E-05	-0.18	Adipose - Subcutaneous
CADM2		1.60E-05	-0.2	Adipose - Visceral (Omentum)
CADM2	rs4856269	4.00E-07	-0.25	Lung

CADM2	rs4856270	1.30E-05	-0.74	Testis
CADM2		6.20E-12	-0.29	Adipose - Subcutaneous
CADM2		1.70E-08	-0.25	Adipose - Visceral (Omentum)
CADM2		4.70E-07	-0.4	Heart - Left Ventricle
CADM2	rs4856271	3.10E-18	-0.41	Lung
CADM2	rs4856272	7.00E-08	-0.35	Lung
CADM2		6.50E-12	-0.29	Adipose - Subcutaneous
CADM2		1.50E-08	-0.25	Adipose - Visceral (Omentum)
CADM2		8.00E-07	-0.39	Heart - Left Ventricle
CADM2		2.20E-18	-0.41	Lung
CADM2	rs4856273	1.70E-04	0.099	Nerve - Tibial
CADM2		7.20E-10	-0.26	Adipose - Subcutaneous
CADM2		4.60E-07	-0.23	Adipose - Visceral (Omentum)
CADM2		1.60E-07	-0.4	Heart - Left Ventricle
CADM2	rs4856274	5.40E-18	-0.4	Lung
CADM2		1.70E-11	-0.28	Adipose - Subcutaneous
CADM2		5.00E-08	-0.25	Adipose - Visceral (Omentum)
CADM2		5.90E-07	-0.4	Heart - Left Ventricle
CADM2	rs4856275	5.70E-16	-0.38	Lung
CADM2		1.10E-11	-0.29	Adipose - Subcutaneous
CADM2		2.50E-07	-0.24	Adipose - Visceral (Omentum)
CADM2		6.30E-06	-0.37	Heart - Left Ventricle
CADM2	rs4856276	5.60E-16	-0.4	Lung
CADM2		2.40E-11	-0.28	Adipose - Subcutaneous
CADM2		2.50E-07	-0.24	Adipose - Visceral (Omentum)
CADM2		1.10E-06	-0.39	Heart - Left Ventricle
CADM2	rs4856277	2.70E-18	-0.42	Lung
CADM2		5.70E-06	-0.39	Heart - Left Ventricle
CADM2	rs4856278	4.30E-12	-0.36	Lung
CADM2-AS1		2.40E-05	0.54	Brain - Caudate (basal ganglia)
CADM2-AS1		1.40E-06	0.58	Brain - Cerebellar Hemisphere
CADM2-AS1		7.60E-07	0.48	Brain - Cerebellum
CADM2-AS1		6.20E-06	0.56	Brain - Frontal Cortex (BA9)
CADM2-AS1	rs4856279	2.00E-05	0.5	Brain - Nucleus accumbens (basal ganglia)
CADM2		3.50E-05	0.19	Adipose - Subcutaneous
CADM2	rs4856553	2.80E-07	0.21	Muscle - Skeletal
CADM2		9.00E-06	0.62	Brain - Spinal cord (cervical c-1)
CADM2	rs4856554	2.60E-06	0.17	Muscle - Skeletal
CADM2	rs4856556	1.90E-06	1.2	Spleen
CADM2	rs4856557	2.80E-06	1.2	Spleen
CADM2	rs4856558	3.00E-10	1.1	Spleen
CADM2		1.20E-04	0.17	Adipose - Subcutaneous
CADM2	rs4856559	8.50E-07	0.19	Muscle - Skeletal
CADM2		1.80E-05	0.19	Adipose - Subcutaneous
CADM2	rs4856560	1.20E-05	0.17	Muscle - Skeletal
CADM2	rs4856568	3.30E-05	-0.67	Heart - Left Ventricle
CADM2		1.80E-10	-0.27	Adipose - Subcutaneous
CADM2		4.80E-08	-0.25	Adipose - Visceral (Omentum)
CADM2		3.60E-07	-0.41	Heart - Left Ventricle
CADM2	rs4856569	3.30E-17	-0.4	Lung
CADM2	rs4856570	7.90E-05	-0.43	Heart - Left Ventricle

CADM2		2.30E-07	-0.34	Lung
CADM2		3.10E-10	-0.26	Adipose - Subcutaneous
CADM2		4.10E-08	-0.25	Adipose - Visceral (Omentum)
CADM2		3.60E-07	-0.41	Heart - Left Ventricle
CADM2	rs4856571	1.50E-17	-0.4	Lung
CADM2		1.20E-10	-0.27	Adipose - Subcutaneous
CADM2		5.80E-08	-0.25	Adipose - Visceral (Omentum)
CADM2		2.90E-07	-0.42	Heart - Left Ventricle
CADM2		1.20E-16	-0.39	Lung
CADM2	rs4856572	1.70E-04	0.1	Nerve - Tibial
CADM2		1.50E-10	-0.27	Adipose - Subcutaneous
CADM2		1.20E-07	-0.24	Adipose - Visceral (Omentum)
CADM2		1.40E-07	-0.41	Heart - Left Ventricle
CADM2	rs4856573	9.00E-16	-0.38	Lung
CADM2		6.40E-12	-0.29	Adipose - Subcutaneous
CADM2		7.50E-09	-0.26	Adipose - Visceral (Omentum)
CADM2		5.80E-07	-0.39	Heart - Left Ventricle
CADM2		6.90E-18	-0.41	Lung
CADM2	rs4856579	1.40E-04	0.1	Nerve - Tibial
CADM2		3.90E-12	-0.29	Adipose - Subcutaneous
CADM2		1.40E-08	-0.25	Adipose - Visceral (Omentum)
CADM2		6.10E-07	-0.39	Heart - Left Ventricle
CADM2		5.80E-18	-0.4	Lung
CADM2	rs4856580	1.30E-04	0.1	Nerve - Tibial
CADM2		4.40E-12	-0.29	Adipose - Subcutaneous
CADM2		1.50E-08	-0.25	Adipose - Visceral (Omentum)
CADM2		6.40E-07	-0.39	Heart - Left Ventricle
CADM2	rs4856581	3.90E-18	-0.4	Lung
CADM2	rs4856582	6.30E-08	-0.35	Lung
CADM2		4.40E-12	-0.29	Adipose - Subcutaneous
CADM2		1.60E-08	-0.25	Adipose - Visceral (Omentum)
CADM2		6.40E-07	-0.39	Heart - Left Ventricle
CADM2	rs4856583	3.10E-18	-0.41	Lung
CADM2		2.00E-09	-0.25	Adipose - Subcutaneous
CADM2		1.10E-07	-0.24	Adipose - Visceral (Omentum)
CADM2		1.30E-07	-0.4	Heart - Left Ventricle
CADM2	rs4856584	2.40E-19	-0.41	Lung
CADM2		6.50E-12	-0.29	Adipose - Subcutaneous
CADM2		1.50E-08	-0.25	Adipose - Visceral (Omentum)
CADM2		8.00E-07	-0.39	Heart - Left Ventricle
CADM2		2.20E-18	-0.41	Lung
CADM2	rs4856585	1.70E-04	0.099	Nerve - Tibial
CADM2		9.10E-12	-0.29	Adipose - Subcutaneous
CADM2		4.40E-08	-0.25	Adipose - Visceral (Omentum)
CADM2		7.10E-07	-0.39	Heart - Left Ventricle
CADM2	rs4856586	2.00E-16	-0.39	Lung
CADM2		2.30E-12	-0.3	Adipose - Subcutaneous
CADM2		1.60E-06	-0.23	Adipose - Visceral (Omentum)
CADM2		5.90E-07	-0.41	Heart - Left Ventricle
CADM2		8.10E-15	-0.39	Lung
CADM2	rs4856587	1.70E-04	-0.15	Muscle - Skeletal
CADM2	rs4856588	7.40E-12	-0.29	Adipose - Subcutaneous

CADM2		1.40E-08	-0.25	Adipose - Visceral (Omentum)
CADM2		7.60E-07	-0.39	Heart - Left Ventricle
CADM2		1.50E-18	-0.41	Lung
CADM2		1.70E-04	0.099	Nerve - Tibial
CADM2		5.50E-11	-0.28	Adipose - Subcutaneous
CADM2		7.90E-08	-0.25	Adipose - Visceral (Omentum)
CADM2		7.00E-07	-0.39	Heart - Left Ventricle
CADM2	rs4856589	7.50E-19	-0.42	Lung
CADM2		1.70E-11	-0.28	Adipose - Subcutaneous
CADM2		3.50E-08	-0.25	Adipose - Visceral (Omentum)
CADM2		1.70E-06	-0.38	Heart - Left Ventricle
CADM2	rs4856590	8.10E-18	-0.4	Lung
CADM2		6.30E-07	-0.22	Adipose - Subcutaneous
CADM2		2.10E-06	-0.22	Adipose - Visceral (Omentum)
CADM2		1.70E-06	-0.39	Heart - Left Ventricle
CADM2	rs4856591	6.30E-16	-0.39	Lung
CADM2		1.40E-11	-0.28	Adipose - Subcutaneous
CADM2		1.50E-08	-0.25	Adipose - Visceral (Omentum)
CADM2		7.40E-07	-0.39	Heart - Left Ventricle
CADM2		1.70E-18	-0.41	Lung
CADM2	rs4856592	1.60E-04	0.099	Nerve - Tibial
CADM2		6.80E-12	-0.29	Adipose - Subcutaneous
CADM2		1.50E-08	-0.25	Adipose - Visceral (Omentum)
CADM2		7.40E-07	-0.39	Heart - Left Ventricle
CADM2		1.80E-18	-0.41	Lung
CADM2	rs4856593	1.60E-04	0.099	Nerve - Tibial
CADM2		2.10E-11	-0.28	Adipose - Subcutaneous
CADM2		4.60E-08	-0.24	Adipose - Visceral (Omentum)
CADM2		1.30E-06	-0.38	Heart - Left Ventricle
CADM2	rs4856596	1.80E-18	-0.41	Lung
CADM2	rs4856597	6.30E-08	-0.35	Lung
CADM2		5.70E-11	-0.28	Adipose - Subcutaneous
CADM2		2.60E-07	-0.24	Adipose - Visceral (Omentum)
CADM2		1.50E-06	-0.38	Heart - Left Ventricle
CADM2	rs4856598	6.20E-17	-0.4	Lung
CADM2	rs4856599	2.60E-05	-0.28	Lung
CADM2		9.50E-05	-0.16	Adipose - Subcutaneous
CADM2	rs4856600	6.60E-12	-0.33	Lung
CADM2-AS1		1.00E-05	0.59	Brain - Caudate (basal ganglia)
CADM2-AS1	rs4856604	9.20E-07	0.53	Brain - Cerebellum
CADM2-AS1		3.60E-07	0.63	Brain - Caudate (basal ganglia)
CADM2-AS1		1.80E-06	0.58	Brain - Cerebellar Hemisphere
CADM2-AS1		5.10E-09	0.58	Brain - Cerebellum
CADM2-AS1	rs4856605	2.00E-05	0.29	Nerve - Tibial
CADM2-AS1		1.30E-05	0.57	Brain - Caudate (basal ganglia)
CADM2-AS1		6.20E-08	0.66	Brain - Cerebellar Hemisphere
CADM2-AS1		1.90E-07	0.49	Brain - Cerebellum
CADM2-AS1		9.90E-06	0.5	Brain - Cortex
CADM2-AS1		1.30E-06	0.61	Brain - Frontal Cortex (BA9)
CADM2-AS1	rs4856606	4.60E-06	0.55	Brain - Nucleus accumbens (basal ganglia)

CADM2-AS1		3.80E-05	0.53	Brain - Caudate (basal ganglia)
CADM2-AS1		1.70E-07	0.62	Brain - Cerebellar Hemisphere
CADM2-AS1		1.30E-07	0.5	Brain - Cerebellum
CADM2-AS1		1.30E-06	0.6	Brain - Frontal Cortex (BA9)
CADM2-AS1	rs4856607	5.50E-06	0.53	Brain - Nucleus accumbens (basal ganglia)
CADM2	rs487539	7.20E-05	-0.14	Muscle - Skeletal
CADM2	rs515207	3.70E-05	-0.15	Muscle - Skeletal
CADM2		2.00E-09	-0.27	Adipose - Subcutaneous
CADM2		4.60E-07	-0.24	Adipose - Visceral (Omentum)
CADM2		8.20E-08	-0.43	Heart - Left Ventricle
CADM2		2.10E-14	-0.38	Lung
CADM2	rs553890444	1.30E-04	-0.15	Muscle - Skeletal
CADM2		4.20E-12	-0.29	Adipose - Subcutaneous
CADM2		3.80E-09	-0.27	Adipose - Visceral (Omentum)
CADM2		8.00E-07	-0.39	Heart - Left Ventricle
CADM2	rs55667839	2.20E-17	-0.4	Lung
CADM2		1.50E-06	-0.2	Adipose - Subcutaneous
CADM2	rs55677942	5.80E-09	-0.27	Lung
CADM2		1.00E-06	-0.23	Lung
CADM2-AS1	rs55686445	1.60E-05	0.28	Nerve - Tibial
CADM2	rs55692107	1.70E-07	1.2	Spleen
CADM2	rs55692455	1.10E-04	0.26	Lung
CADM2		1.10E-07	0.26	Adipose - Subcutaneous
CADM2		2.70E-05	0.22	Adipose - Visceral (Omentum)
CADM2	rs55743890	1.30E-06	0.27	Lung
CADM2	rs55753638	6.60E-07	0.35	Lung
CADM2		4.50E-09	-0.24	Adipose - Subcutaneous
CADM2		3.60E-07	-0.23	Adipose - Visceral (Omentum)
CADM2		3.80E-08	-0.42	Heart - Left Ventricle
CADM2	rs557667709	9.50E-19	-0.41	Lung
CADM2-AS1		1.50E-07	0.62	Brain - Caudate (basal ganglia)
CADM2-AS1		2.80E-06	0.54	Brain - Cerebellar Hemisphere
CADM2-AS1		3.00E-08	0.53	Brain - Cerebellum
CADM2-AS1	rs55776605	4.30E-05	0.46	Brain - Cortex
CADM2-AS1		1.50E-07	0.62	Brain - Caudate (basal ganglia)
CADM2-AS1		2.80E-06	0.54	Brain - Cerebellar Hemisphere
CADM2-AS1		3.00E-08	0.53	Brain - Cerebellum
CADM2-AS1	rs55782528	4.30E-05	0.46	Brain - Cortex
CADM2	rs55804926	1.70E-06	1.2	Spleen
CADM2-AS1	rs55829275	6.00E-05	0.39	Brain - Cerebellum
CADM2	rs55843200	7.70E-05	0.27	Lung
CADM2	rs55873202	3.70E-05	0.28	Lung
CADM2	rs55905356	2.60E-05	0.29	Lung
CADM2		1.30E-12	-0.3	Adipose - Subcutaneous
CADM2		1.10E-08	-0.25	Adipose - Visceral (Omentum)
CADM2		7.40E-07	-0.39	Heart - Left Ventricle
CADM2	rs55939743	1.10E-17	-0.4	Lung
CADM2	rs55964300	4.40E-05	0.27	Lung
CADM2-AS1	rs55971481	3.50E-07	0.61	Brain - Caudate (basal ganglia)

CADM2-AS1		9.10E-10	0.69	Brain - Cerebellar Hemisphere
CADM2-AS1		2.10E-10	0.59	Brain - Cerebellum
CADM2-AS1		1.70E-05	0.52	Brain - Frontal Cortex (BA9)
CADM2-AS1		2.20E-05	0.56	Brain - Hippocampus
CADM2-AS1		1.00E-06	0.57	Brain - Nucleus accumbens (basal ganglia)
CADM2	rs55980308	3.00E-06	1.2	Spleen
CADM2		1.10E-09	-0.25	Adipose - Subcutaneous
CADM2		1.50E-07	-0.24	Adipose - Visceral (Omentum)
CADM2		2.30E-07	-0.39	Heart - Left Ventricle
CADM2	rs56031423	4.00E-19	-0.41	Lung
CADM2-AS1		1.40E-07	0.59	Brain - Caudate (basal ganglia)
CADM2-AS1		3.30E-05	0.47	Brain - Cerebellar Hemisphere
CADM2-AS1	rs56040638	7.70E-08	0.49	Brain - Cerebellum
CADM2		1.00E-06	-0.21	Adipose - Subcutaneous
CADM2	rs56063967	5.10E-09	-0.27	Lung
CADM2-AS1		8.50E-06	0.59	Brain - Cerebellar Hemisphere
CADM2-AS1		2.30E-07	0.52	Brain - Cerebellum
CADM2-AS1		2.70E-05	0.5	Brain - Cortex
CADM2-AS1		5.70E-06	0.6	Brain - Frontal Cortex (BA9)
CADM2-AS1	rs56128407	1.70E-05	0.53	Brain - Nucleus accumbens (basal ganglia)
CADM2		1.80E-12	-0.29	Adipose - Subcutaneous
CADM2		1.50E-08	-0.25	Adipose - Visceral (Omentum)
CADM2		7.40E-07	-0.39	Heart - Left Ventricle
CADM2		4.50E-18	-0.4	Lung
CADM2	rs56187922	1.70E-04	0.099	Nerve - Tibial
CADM2		5.20E-05	0.17	Adipose - Subcutaneous
CADM2		5.00E-06	0.61	Brain - Spinal cord (cervical c-1)
CADM2	rs562272869	1.70E-06	0.17	Muscle - Skeletal
CADM2-AS1		1.20E-05	0.56	Brain - Cerebellar Hemisphere
CADM2-AS1		3.80E-07	0.58	Brain - Cerebellum
CADM2-AS1		4.20E-06	0.61	Brain - Nucleus accumbens (basal ganglia)
CADM2-AS1	rs56262138	3.60E-05	0.6	Brain - Putamen (basal ganglia)
CADM2-AS1		4.90E-08	0.65	Brain - Cerebellar Hemisphere
CADM2-AS1		2.10E-07	0.49	Brain - Cerebellum
CADM2-AS1		1.00E-05	0.5	Brain - Cortex
CADM2-AS1		4.90E-06	0.57	Brain - Frontal Cortex (BA9)
CADM2-AS1	rs56282418	7.30E-06	0.54	Brain - Nucleus accumbens (basal ganglia)
CADM2-AS1		7.30E-08	0.66	Brain - Caudate (basal ganglia)
CADM2-AS1		1.80E-10	0.74	Brain - Cerebellar Hemisphere
CADM2-AS1		1.20E-11	0.62	Brain - Cerebellum
CADM2-AS1		3.50E-07	0.58	Brain - Cortex
CADM2-AS1		1.80E-07	0.65	Brain - Frontal Cortex (BA9)
CADM2-AS1		9.50E-07	0.68	Brain - Hippocampus
CADM2-AS1		3.20E-09	0.7	Brain - Nucleus accumbens (basal ganglia)
CADM2-AS1	rs56339824	1.10E-04	0.25	Nerve - Tibial
CADM2	rs56701506	1.20E-04	-0.94	Testis
CADM2	rs567367148	5.80E-05	1.4	Heart - Left Ventricle
CADM2-AS1	rs56760958	3.80E-07	0.61	Brain - Caudate (basal ganglia)

CADM2-AS1		2.20E-10	0.71	Brain - Cerebellar Hemisphere
CADM2-AS1		4.90E-12	0.62	Brain - Cerebellum
CADM2-AS1		2.40E-06	0.57	Brain - Frontal Cortex (BA9)
CADM2-AS1		2.40E-05	0.56	Brain - Hippocampus
CADM2-AS1		7.70E-08	0.62	Brain - Nucleus accumbens (basal ganglia)
CADM2-AS1		1.00E-04	0.25	Nerve - Tibial
CADM2		1.80E-12	-0.3	Adipose - Subcutaneous
CADM2		1.30E-08	-0.25	Adipose - Visceral (Omentum)
CADM2		1.50E-06	-0.38	Heart - Left Ventricle
CADM2	rs56777875	1.50E-17	-0.4	Lung
CADM2		4.40E-12	-0.29	Adipose - Subcutaneous
CADM2		1.40E-08	-0.25	Adipose - Visceral (Omentum)
CADM2		6.10E-07	-0.39	Heart - Left Ventricle
CADM2	rs56778912	3.50E-18	-0.4	Lung
CADM2		1.10E-11	-0.28	Adipose - Subcutaneous
CADM2		3.20E-08	-0.25	Adipose - Visceral (Omentum)
CADM2		6.60E-07	-0.39	Heart - Left Ventricle
CADM2	rs56779213	3.90E-18	-0.4	Lung
CADM2		3.90E-12	-0.29	Adipose - Subcutaneous
CADM2		2.00E-08	-0.25	Adipose - Visceral (Omentum)
CADM2		4.30E-07	-0.39	Heart - Left Ventricle
CADM2		1.60E-17	-0.4	Lung
CADM2	rs56816351	1.40E-04	-0.14	Muscle - Skeletal
CADM2	rs569250722	5.50E-05	1.4	Heart - Left Ventricle
CADM2		6.80E-12	-0.29	Adipose - Subcutaneous
CADM2		3.10E-08	-0.25	Adipose - Visceral (Omentum)
CADM2		6.90E-07	-0.39	Heart - Left Ventricle
CADM2		1.70E-18	-0.41	Lung
CADM2	rs570875041	1.60E-04	0.099	Nerve - Tibial
CADM2-AS1		1.70E-06	0.56	Brain - Caudate (basal ganglia)
CADM2-AS1		2.70E-05	0.49	Brain - Cerebellar Hemisphere
CADM2-AS1		2.40E-08	0.52	Brain - Cerebellum
CADM2-AS1	rs57153235	3.80E-06	0.31	Nerve - Tibial
CADM2	rs57155100	2.30E-05	-0.18	Muscle - Skeletal
CADM2		6.80E-12	-0.29	Adipose - Subcutaneous
CADM2		1.50E-08	-0.25	Adipose - Visceral (Omentum)
CADM2		7.40E-07	-0.39	Heart - Left Ventricle
CADM2		1.80E-18	-0.41	Lung
CADM2	rs57276248	1.60E-04	0.099	Nerve - Tibial
CADM2		1.20E-10	-0.28	Adipose - Subcutaneous
CADM2		6.50E-07	-0.23	Adipose - Visceral (Omentum)
CADM2		9.60E-07	-0.39	Heart - Left Ventricle
CADM2	rs57400427	1.10E-15	-0.39	Lung
CADM2-AS1		1.30E-06	0.57	Brain - Caudate (basal ganglia)
CADM2-AS1		3.60E-05	0.49	Brain - Cerebellar Hemisphere
CADM2-AS1		2.80E-08	0.52	Brain - Cerebellum
CADM2-AS1	rs57533494	1.20E-05	0.29	Nerve - Tibial
CADM2		1.30E-10	-0.27	Adipose - Subcutaneous
CADM2		1.20E-07	-0.24	Adipose - Visceral (Omentum)
CADM2	rs57630146	1.50E-07	-0.41	Heart - Left Ventricle

CADM2		2.70E-15	-0.37	Lung
CADM2		1.80E-12	-0.29	Adipose - Subcutaneous
CADM2		1.50E-08	-0.25	Adipose - Visceral (Omentum)
CADM2		7.40E-07	-0.39	Heart - Left Ventricle
CADM2		3.90E-18	-0.4	Lung
CADM2		1.70E-04	0.099	Nerve - Tibial
CADM2		3.00E-09	-0.24	Adipose - Subcutaneous
CADM2		1.10E-07	-0.24	Adipose - Visceral (Omentum)
CADM2		1.60E-07	-0.4	Heart - Left Ventricle
CADM2	rs57755423	2.40E-19	-0.41	Lung
CADM2		3.90E-12	-0.29	Adipose - Subcutaneous
CADM2		1.90E-08	-0.25	Adipose - Visceral (Omentum)
CADM2		1.20E-06	-0.38	Heart - Left Ventricle
CADM2		2.00E-18	-0.41	Lung
CADM2	rs57756567	1.80E-04	0.099	Nerve - Tibial
CADM2		8.60E-11	-0.29	Adipose - Subcutaneous
CADM2		1.00E-06	-0.23	Adipose - Visceral (Omentum)
CADM2		4.40E-07	-0.41	Heart - Left Ventricle
CADM2	rs57813006	4.80E-15	-0.39	Lung
CADM2		5.40E-12	-0.29	Adipose - Subcutaneous
CADM2		9.80E-09	-0.26	Adipose - Visceral (Omentum)
CADM2		6.50E-07	-0.39	Heart - Left Ventricle
CADM2	rs57872438	1.70E-17	-0.4	Lung
CADM2		4.20E-12	-0.29	Adipose - Subcutaneous
CADM2		2.50E-09	-0.27	Adipose - Visceral (Omentum)
CADM2		7.10E-07	-0.39	Heart - Left Ventricle
CADM2	rs58175373	1.40E-17	-0.4	Lung
CADM2-AS1		3.60E-07	0.6	Brain - Caudate (basal ganglia)
CADM2-AS1		5.50E-06	0.53	Brain - Cerebellar Hemisphere
CADM2-AS1	rs58382609	3.00E-08	0.53	Brain - Cerebellum
CADM2		1.20E-11	-0.29	Adipose - Subcutaneous
CADM2		2.90E-07	-0.23	Adipose - Visceral (Omentum)
CADM2		3.90E-07	-0.4	Heart - Left Ventricle
CADM2	rs5850689	1.60E-17	-0.4	Lung
CADM2		5.60E-12	-0.29	Adipose - Subcutaneous
CADM2		1.50E-08	-0.25	Adipose - Visceral (Omentum)
CADM2		7.40E-07	-0.39	Heart - Left Ventricle
CADM2		1.80E-18	-0.41	Lung
CADM2	rs5850691	1.60E-04	0.099	Nerve - Tibial
CADM2		1.60E-11	-0.28	Adipose - Subcutaneous
CADM2		2.80E-08	-0.25	Adipose - Visceral (Omentum)
CADM2		1.70E-07	-0.41	Heart - Left Ventricle
CADM2	rs5850698	3.50E-17	-0.39	Lung
CADM2-AS1		1.60E-06	0.53	Brain - Cerebellar Hemisphere
CADM2-AS1		1.20E-05	0.43	Brain - Cerebellum
CADM2-AS1	rs5850707	3.10E-06	0.53	Brain - Nucleus accumbens (basal ganglia)
CADM2		2.60E-11	-0.28	Adipose - Subcutaneous
CADM2		4.50E-08	-0.24	Adipose - Visceral (Omentum)
CADM2		1.90E-07	-0.41	Heart - Left Ventricle
CADM2	rs58545525	1.20E-15	-0.38	Lung
CADM2-AS1	rs58783194	6.90E-07	0.61	Brain - Caudate (basal ganglia)

CADM2-AS1		1.90E-07	0.66	Brain - Cerebellar Hemisphere
CADM2-AS1		9.80E-09	0.56	Brain - Cerebellum
CADM2-AS1		4.10E-07	0.69	Brain - Frontal Cortex (BA9)
CADM2-AS1		1.10E-05	0.57	Brain - Nucleus accumbens (basal ganglia)
CADM2		4.40E-12	-0.29	Adipose - Subcutaneous
CADM2		1.40E-08	-0.25	Adipose - Visceral (Omentum)
CADM2		6.10E-07	-0.39	Heart - Left Ventricle
CADM2	rs58828944	3.50E-18	-0.4	Lung
CADM2		1.40E-06	-0.21	Adipose - Subcutaneous
CADM2		6.00E-09	-0.27	Lung
CADM2-AS1	rs58834564	5.70E-05	0.27	Nerve - Tibial
CADM2		6.80E-12	-0.29	Adipose - Subcutaneous
CADM2		1.50E-08	-0.25	Adipose - Visceral (Omentum)
CADM2		7.40E-07	-0.39	Heart - Left Ventricle
CADM2		1.80E-18	-0.41	Lung
CADM2	rs58889493	1.60E-04	0.099	Nerve - Tibial
CADM2		3.50E-12	-0.29	Adipose - Subcutaneous
CADM2		3.10E-08	-0.25	Adipose - Visceral (Omentum)
CADM2		6.40E-07	-0.39	Heart - Left Ventricle
CADM2		7.30E-18	-0.4	Lung
CADM2	rs58919842	1.10E-04	0.1	Nerve - Tibial
CADM2		6.80E-12	-0.29	Adipose - Subcutaneous
CADM2		1.50E-08	-0.25	Adipose - Visceral (Omentum)
CADM2		7.40E-07	-0.39	Heart - Left Ventricle
CADM2		1.80E-18	-0.41	Lung
CADM2	rs59073108	1.60E-04	0.099	Nerve - Tibial
CADM2		5.10E-12	-0.29	Adipose - Subcutaneous
CADM2		3.10E-08	-0.25	Adipose - Visceral (Omentum)
CADM2		5.10E-07	-0.4	Heart - Left Ventricle
CADM2		1.60E-18	-0.41	Lung
CADM2	rs59211082	1.50E-04	0.1	Nerve - Tibial
CADM2		2.20E-11	-0.28	Adipose - Subcutaneous
CADM2		1.70E-08	-0.25	Adipose - Visceral (Omentum)
CADM2		6.70E-07	-0.39	Heart - Left Ventricle
CADM2	rs59225869	2.30E-18	-0.41	Lung
CADM2		1.30E-11	-0.28	Adipose - Subcutaneous
CADM2		1.50E-08	-0.25	Adipose - Visceral (Omentum)
CADM2		1.10E-06	-0.38	Heart - Left Ventricle
CADM2		1.80E-18	-0.41	Lung
CADM2	rs59417256	1.00E-04	0.1	Nerve - Tibial
CADM2		6.90E-12	-0.29	Adipose - Subcutaneous
CADM2		3.60E-08	-0.25	Adipose - Visceral (Omentum)
CADM2		7.50E-07	-0.39	Heart - Left Ventricle
CADM2	rs59491876	4.20E-18	-0.4	Lung
CADM2	rs59523340	1.20E-04	-0.94	Testis
CADM2	rs59741403	1.20E-05	-1.5	Heart - Atrial Appendage
CADM2		7.60E-12	-0.29	Adipose - Subcutaneous
CADM2		3.30E-08	-0.25	Adipose - Visceral (Omentum)
CADM2		1.30E-06	-0.38	Heart - Left Ventricle
CADM2		2.00E-18	-0.41	Lung
CADM2	rs59770976	1.80E-04	0.098	Nerve - Tibial

CADM2		4.70E-12	-0.29	Adipose - Subcutaneous
CADM2		1.60E-08	-0.25	Adipose - Visceral (Omentum)
CADM2		6.90E-07	-0.39	Heart - Left Ventricle
CADM2	rs59825726	3.90E-18	-0.4	Lung
CADM2		6.80E-12	-0.29	Adipose - Subcutaneous
CADM2		1.50E-08	-0.25	Adipose - Visceral (Omentum)
CADM2		7.40E-07	-0.39	Heart - Left Ventricle
CADM2		1.80E-18	-0.41	Lung
CADM2	rs59835020	1.60E-04	0.099	Nerve - Tibial
CADM2-AS1		9.70E-06	0.58	Brain - Caudate (basal ganglia)
CADM2-AS1		5.80E-08	0.66	Brain - Cerebellar Hemisphere
CADM2-AS1		2.00E-07	0.49	Brain - Cerebellum
CADM2-AS1		8.70E-06	0.51	Brain - Cortex
CADM2-AS1		1.20E-06	0.61	Brain - Frontal Cortex (BA9)
CADM2-AS1	rs59835871	5.50E-06	0.54	Brain - Nucleus accumbens (basal ganglia)
CADM2-AS1		1.30E-06	0.56	Brain - Caudate (basal ganglia)
CADM2-AS1		3.60E-05	0.49	Brain - Cerebellar Hemisphere
CADM2-AS1		2.80E-08	0.52	Brain - Cerebellum
CADM2-AS1	rs59937260	1.60E-05	0.29	Nerve - Tibial
CADM2		4.20E-12	-0.29	Adipose - Subcutaneous
CADM2		1.40E-08	-0.25	Adipose - Visceral (Omentum)
CADM2		6.20E-07	-0.39	Heart - Left Ventricle
CADM2	rs59967234	3.50E-18	-0.4	Lung
CADM2	rs60186266	1.60E-07	-0.34	Lung
CADM2		3.10E-10	-0.26	Adipose - Subcutaneous
CADM2		4.30E-08	-0.25	Adipose - Visceral (Omentum)
CADM2		3.20E-07	-0.42	Heart - Left Ventricle
CADM2	rs60311538	1.90E-17	-0.4	Lung
CADM2		4.40E-12	-0.29	Adipose - Subcutaneous
CADM2		1.40E-08	-0.25	Adipose - Visceral (Omentum)
CADM2		6.10E-07	-0.39	Heart - Left Ventricle
CADM2	rs60427790	3.50E-18	-0.4	Lung
CADM2		1.00E-09	-0.25	Adipose - Subcutaneous
CADM2		1.00E-07	-0.24	Adipose - Visceral (Omentum)
CADM2		2.00E-07	-0.39	Heart - Left Ventricle
CADM2	rs60541362	8.80E-20	-0.41	Lung
CADM2-AS1		1.50E-07	0.62	Brain - Caudate (basal ganglia)
CADM2-AS1		2.80E-06	0.54	Brain - Cerebellar Hemisphere
CADM2-AS1	rs60732670	1.10E-07	0.52	Brain - Cerebellum
CADM2-AS1		1.20E-05	0.59	Brain - Caudate (basal ganglia)
CADM2-AS1		1.50E-06	0.61	Brain - Cerebellar Hemisphere
CADM2-AS1		1.40E-07	0.52	Brain - Cerebellum
CADM2-AS1		2.20E-05	0.49	Brain - Cortex
CADM2-AS1		2.90E-06	0.61	Brain - Frontal Cortex (BA9)
CADM2-AS1	rs60756742	2.20E-06	0.57	Brain - Nucleus accumbens (basal ganglia)
CADM2		3.90E-11	-0.28	Adipose - Subcutaneous
CADM2		6.20E-08	-0.24	Adipose - Visceral (Omentum)
CADM2		1.90E-07	-0.41	Heart - Left Ventricle
CADM2	rs60759992	4.50E-16	-0.38	Lung
CADM2	rs608760	2.60E-05	0.18	Adipose - Visceral (Omentum)

CADM2		6.40E-09	-0.24	Adipose - Subcutaneous
CADM2		2.20E-08	-0.26	Adipose - Visceral (Omentum)
CADM2		4.90E-07	-0.38	Heart - Left Ventricle
CADM2	rs60880662	2.60E-19	-0.41	Lung
CADM2		6.50E-12	-0.29	Adipose - Subcutaneous
CADM2		1.40E-08	-0.25	Adipose - Visceral (Omentum)
CADM2		7.40E-07	-0.39	Heart - Left Ventricle
CADM2		1.60E-18	-0.41	Lung
CADM2	rs61316596	1.60E-04	0.099	Nerve - Tibial
CADM2-AS1		3.60E-07	0.6	Brain - Caudate (basal ganglia)
CADM2-AS1		5.50E-06	0.53	Brain - Cerebellar Hemisphere
CADM2-AS1	rs61439785	3.00E-08	0.53	Brain - Cerebellum
CADM2		3.00E-12	-0.29	Adipose - Subcutaneous
CADM2		6.50E-08	-0.25	Adipose - Visceral (Omentum)
CADM2		1.60E-06	-0.38	Heart - Left Ventricle
CADM2		5.20E-17	-0.4	Lung
CADM2	rs61586601	1.40E-04	0.1	Nerve - Tibial
CADM2		2.30E-11	-0.28	Adipose - Subcutaneous
CADM2		2.20E-08	-0.25	Adipose - Visceral (Omentum)
CADM2		3.40E-06	-0.36	Heart - Left Ventricle
CADM2		7.20E-19	-0.41	Lung
CADM2	rs62250463	1.60E-04	0.1	Nerve - Tibial
CADM2		1.00E-06	-0.21	Adipose - Subcutaneous
CADM2	rs62250464	1.80E-09	-0.27	Lung
CADM2		1.50E-11	-0.28	Adipose - Subcutaneous
CADM2		1.50E-08	-0.25	Adipose - Visceral (Omentum)
CADM2		7.50E-07	-0.39	Heart - Left Ventricle
CADM2		3.50E-18	-0.41	Lung
CADM2	rs62250465	1.10E-04	0.1	Nerve - Tibial
CADM2		6.80E-12	-0.29	Adipose - Subcutaneous
CADM2		1.50E-08	-0.25	Adipose - Visceral (Omentum)
CADM2		7.40E-07	-0.39	Heart - Left Ventricle
CADM2		1.80E-18	-0.41	Lung
CADM2	rs62250467	1.60E-04	0.099	Nerve - Tibial
CADM2		2.70E-12	-0.29	Adipose - Subcutaneous
CADM2		1.50E-08	-0.26	Adipose - Visceral (Omentum)
CADM2		6.50E-07	-0.39	Heart - Left Ventricle
CADM2		5.20E-18	-0.41	Lung
CADM2	rs62250468	1.20E-04	0.1	Nerve - Tibial
CADM2		1.00E-06	-0.21	Adipose - Subcutaneous
CADM2	rs62250471	2.60E-09	-0.27	Lung
CADM2		6.70E-12	-0.29	Adipose - Subcutaneous
CADM2		1.40E-08	-0.25	Adipose - Visceral (Omentum)
CADM2		7.40E-07	-0.39	Heart - Left Ventricle
CADM2		1.70E-18	-0.41	Lung
CADM2	rs62250472	1.60E-04	0.099	Nerve - Tibial
CADM2		8.60E-12	-0.29	Adipose - Subcutaneous
CADM2		5.80E-08	-0.25	Adipose - Visceral (Omentum)
CADM2		8.60E-07	-0.39	Heart - Left Ventricle
CADM2		1.20E-17	-0.4	Lung
CADM2	rs62250489	9.70E-05	0.1	Nerve - Tibial

CADM2		1.70E-11	-0.28	Adipose - Subcutaneous
CADM2		8.30E-08	-0.24	Adipose - Visceral (Omentum)
CADM2		9.50E-07	-0.39	Heart - Left Ventricle
CADM2		4.90E-17	-0.4	Lung
CADM2	rs62250490	1.30E-04	0.1	Nerve - Tibial
CADM2		1.20E-11	-0.28	Adipose - Subcutaneous
CADM2		1.50E-08	-0.25	Adipose - Visceral (Omentum)
CADM2		1.60E-06	-0.38	Heart - Left Ventricle
CADM2		1.80E-18	-0.41	Lung
CADM2	rs62250491	1.80E-04	0.099	Nerve - Tibial
CADM2		1.20E-11	-0.28	Adipose - Subcutaneous
CADM2		1.50E-08	-0.25	Adipose - Visceral (Omentum)
CADM2		1.60E-06	-0.38	Heart - Left Ventricle
CADM2		1.80E-18	-0.41	Lung
CADM2	rs62250492	1.80E-04	0.099	Nerve - Tibial
CADM2		6.60E-12	-0.29	Adipose - Subcutaneous
CADM2		6.60E-09	-0.26	Adipose - Visceral (Omentum)
CADM2		6.70E-07	-0.39	Heart - Left Ventricle
CADM2		3.10E-17	-0.4	Lung
CADM2	rs62250496	1.00E-04	0.1	Nerve - Tibial
CADM2		2.20E-11	-0.28	Adipose - Subcutaneous
CADM2		4.80E-08	-0.24	Adipose - Visceral (Omentum)
CADM2		2.50E-06	-0.37	Heart - Left Ventricle
CADM2	rs62250500	4.40E-18	-0.4	Lung
CADM2		4.10E-11	-0.28	Adipose - Subcutaneous
CADM2		1.70E-07	-0.23	Adipose - Visceral (Omentum)
CADM2		1.10E-06	-0.38	Heart - Left Ventricle
CADM2	rs62250501	5.00E-19	-0.41	Lung
CADM2		3.70E-11	-0.28	Adipose - Subcutaneous
CADM2		4.00E-08	-0.24	Adipose - Visceral (Omentum)
CADM2		2.00E-06	-0.37	Heart - Left Ventricle
CADM2	rs62250502	3.10E-18	-0.41	Lung
CADM2		2.60E-11	-0.28	Adipose - Subcutaneous
CADM2		9.50E-08	-0.24	Adipose - Visceral (Omentum)
CADM2		2.50E-06	-0.37	Heart - Left Ventricle
CADM2		1.70E-18	-0.41	Lung
CADM2	rs62250503	1.60E-04	0.1	Nerve - Tibial
CADM2		2.30E-06	-0.2	Adipose - Subcutaneous
CADM2	rs62250504	4.30E-09	-0.27	Lung
CADM2		2.70E-09	-0.24	Adipose - Subcutaneous
CADM2		2.40E-07	-0.23	Adipose - Visceral (Omentum)
CADM2		3.60E-07	-0.38	Heart - Left Ventricle
CADM2	rs62250537	1.30E-19	-0.41	Lung
CADM2		2.40E-10	-0.27	Adipose - Subcutaneous
CADM2		2.40E-07	-0.24	Adipose - Visceral (Omentum)
CADM2		2.80E-06	-0.37	Heart - Left Ventricle
CADM2	rs62250540	1.60E-18	-0.42	Lung
CADM2		9.10E-05	0.15	Adipose - Subcutaneous
CADM2	rs62250629	2.30E-05	0.15	Muscle - Skeletal
CADM2		5.20E-07	-0.2	Adipose - Subcutaneous
CADM2		5.40E-06	-0.19	Adipose - Visceral (Omentum)
CADM2	rs62250661	1.00E-08	-0.26	Lung

CADM2		9.90E-09	-0.24	Adipose - Subcutaneous
CADM2		5.70E-08	-0.25	Adipose - Visceral (Omentum)
CADM2		3.70E-07	-0.42	Heart - Left Ventricle
CADM2		1.70E-17	-0.41	Lung
CADM2	rs62250685	1.00E-04	0.1	Nerve - Tibial
CADM2		5.10E-09	-0.26	Adipose - Subcutaneous
CADM2		4.00E-06	-0.23	Adipose - Visceral (Omentum)
CADM2		3.80E-06	-0.4	Heart - Left Ventricle
CADM2	rs62250686	1.60E-11	-0.35	Lung
CADM2		1.50E-10	-0.27	Adipose - Subcutaneous
CADM2		7.00E-08	-0.24	Adipose - Visceral (Omentum)
CADM2		2.00E-07	-0.4	Heart - Left Ventricle
CADM2		2.40E-14	-0.36	Lung
CADM2	rs62250687	1.50E-04	-0.14	Muscle - Skeletal
CADM2	rs62250693	1.80E-06	0.34	Lung
CADM2	rs62250710	1.80E-06	0.33	Lung
CADM2		4.10E-11	-0.28	Adipose - Subcutaneous
CADM2		6.40E-08	-0.24	Adipose - Visceral (Omentum)
CADM2		1.90E-07	-0.41	Heart - Left Ventricle
CADM2		4.70E-16	-0.38	Lung
CADM2	rs62250711	1.70E-04	-0.14	Muscle - Skeletal
CADM2		1.00E-10	-0.27	Adipose - Subcutaneous
CADM2		6.70E-08	-0.25	Adipose - Visceral (Omentum)
CADM2		7.40E-07	-0.4	Heart - Left Ventricle
CADM2	rs62250712	3.40E-17	-0.4	Lung
CADM2		5.90E-10	-0.26	Adipose - Subcutaneous
CADM2		8.30E-08	-0.25	Adipose - Visceral (Omentum)
CADM2		4.40E-07	-0.41	Heart - Left Ventricle
CADM2	rs62250713	6.20E-19	-0.42	Lung
CADM2		1.30E-12	-0.3	Adipose - Subcutaneous
CADM2		3.70E-08	-0.25	Adipose - Visceral (Omentum)
CADM2		2.10E-07	-0.4	Heart - Left Ventricle
CADM2		1.10E-16	-0.39	Lung
CADM2	rs62250714	1.40E-04	-0.14	Muscle - Skeletal
CADM2		4.90E-13	-0.3	Adipose - Subcutaneous
CADM2		2.00E-08	-0.25	Adipose - Visceral (Omentum)
CADM2		1.70E-07	-0.41	Heart - Left Ventricle
CADM2		1.60E-16	-0.39	Lung
CADM2	rs62250715	8.30E-05	-0.15	Muscle - Skeletal
CADM2		3.50E-06	-0.2	Adipose - Subcutaneous
CADM2		2.10E-09	-0.27	Lung
CADM2-AS1	rs62250716	1.20E-04	0.26	Nerve - Tibial
CADM2		1.70E-06	-0.21	Adipose - Subcutaneous
CADM2		7.90E-10	-0.28	Lung
CADM2-AS1	rs62250717	5.70E-05	0.27	Nerve - Tibial
CADM2		1.60E-05	-0.22	Adipose - Subcutaneous
CADM2		2.40E-05	-0.23	Adipose - Visceral (Omentum)
CADM2	rs62250718	9.90E-05	-0.23	Lung
CADM2		5.90E-11	-0.28	Adipose - Subcutaneous
CADM2		1.40E-07	-0.24	Adipose - Visceral (Omentum)
CADM2		9.70E-07	-0.39	Heart - Left Ventricle
CADM2	rs62250719	2.20E-16	-0.4	Lung

CADM2	rs62250722	9.30E-05	0.3	Lung
CADM2		1.30E-05	-0.21	Adipose - Subcutaneous
CADM2		8.10E-05	-0.2	Adipose - Visceral (Omentum)
CADM2	rs62250723	9.80E-07	-0.26	Lung
CADM2		4.30E-06	-0.2	Adipose - Subcutaneous
CADM2	rs62250724	4.80E-10	-0.28	Lung
CADM2		9.40E-06	-0.21	Adipose - Subcutaneous
CADM2	rs62250748	3.10E-06	-0.24	Lung
CADM2		1.50E-12	-0.3	Adipose - Subcutaneous
CADM2		1.10E-08	-0.26	Adipose - Visceral (Omentum)
CADM2		1.20E-06	-0.38	Heart - Left Ventricle
CADM2	rs62250750	2.70E-17	-0.4	Lung
CADM2		4.30E-12	-0.29	Adipose - Subcutaneous
CADM2		4.00E-09	-0.27	Adipose - Visceral (Omentum)
CADM2		9.90E-07	-0.38	Heart - Left Ventricle
CADM2	rs62250752	2.20E-17	-0.4	Lung
CADM2		4.40E-12	-0.29	Adipose - Subcutaneous
CADM2		1.50E-08	-0.25	Adipose - Visceral (Omentum)
CADM2		6.40E-07	-0.39	Heart - Left Ventricle
CADM2	rs62250754	3.90E-18	-0.4	Lung
CADM2		1.40E-05	-0.19	Adipose - Subcutaneous
CADM2		1.20E-09	-0.28	Lung
CADM2-AS1	rs62250755	8.70E-05	0.26	Nerve - Tibial
CADM2		1.50E-10	-0.28	Adipose - Subcutaneous
CADM2		1.10E-07	-0.26	Adipose - Visceral (Omentum)
CADM2		7.50E-07	-0.4	Heart - Left Ventricle
CADM2		8.70E-15	-0.38	Lung
CADM2	rs62250758	1.60E-04	0.1	Nerve - Tibial
CADM2		2.60E-06	-0.2	Adipose - Subcutaneous
CADM2		7.20E-10	-0.28	Lung
CADM2-AS1	rs62250759	1.30E-04	0.25	Nerve - Tibial
CADM2	rs62251966	6.10E-05	-0.15	Muscle - Skeletal
CADM2		3.40E-06	-0.2	Adipose - Subcutaneous
CADM2	rs62252460	9.70E-10	-0.28	Lung
CADM2		2.10E-06	-0.2	Adipose - Subcutaneous
CADM2		2.10E-09	-0.27	Lung
CADM2-AS1	rs62252461	1.10E-04	0.26	Nerve - Tibial
CADM2		4.40E-12	-0.29	Adipose - Subcutaneous
CADM2		1.50E-08	-0.25	Adipose - Visceral (Omentum)
CADM2		6.40E-07	-0.39	Heart - Left Ventricle
CADM2	rs62252462	3.90E-18	-0.4	Lung
CADM2		5.70E-12	-0.29	Adipose - Subcutaneous
CADM2		1.50E-08	-0.25	Adipose - Visceral (Omentum)
CADM2		6.50E-07	-0.39	Heart - Left Ventricle
CADM2		1.40E-17	-0.4	Lung
CADM2	rs62252463	1.70E-04	0.1	Nerve - Tibial
CADM2		3.80E-12	-0.29	Adipose - Subcutaneous
CADM2		1.40E-08	-0.25	Adipose - Visceral (Omentum)
CADM2		1.10E-06	-0.38	Heart - Left Ventricle
CADM2	rs62252464	6.40E-18	-0.4	Lung
CADM2		3.80E-12	-0.29	Adipose - Subcutaneous
CADM2	rs62252465	1.40E-08	-0.25	Adipose - Visceral (Omentum)

CADM2		1.10E-06	-0.38	Heart - Left Ventricle
CADM2		6.40E-18	-0.4	Lung
CADM2		7.10E-12	-0.29	Adipose - Subcutaneous
CADM2		1.70E-08	-0.25	Adipose - Visceral (Omentum)
CADM2		5.70E-07	-0.39	Heart - Left Ventricle
CADM2	rs62252466	1.50E-17	-0.4	Lung
CADM2		5.10E-12	-0.29	Adipose - Subcutaneous
CADM2		1.90E-08	-0.25	Adipose - Visceral (Omentum)
CADM2		6.40E-07	-0.39	Heart - Left Ventricle
CADM2	rs62252467	7.70E-18	-0.4	Lung
CADM2		1.80E-12	-0.29	Adipose - Subcutaneous
CADM2		1.50E-08	-0.25	Adipose - Visceral (Omentum)
CADM2		7.40E-07	-0.39	Heart - Left Ventricle
CADM2		3.90E-18	-0.4	Lung
CADM2	rs62252495	1.70E-04	0.099	Nerve - Tibial
CADM2		6.70E-13	-0.3	Adipose - Subcutaneous
CADM2		1.40E-08	-0.25	Adipose - Visceral (Omentum)
CADM2		1.70E-06	-0.38	Heart - Left Ventricle
CADM2	rs62252496	1.70E-17	-0.4	Lung
CADM2		1.80E-12	-0.29	Adipose - Subcutaneous
CADM2		1.50E-08	-0.25	Adipose - Visceral (Omentum)
CADM2		7.40E-07	-0.39	Heart - Left Ventricle
CADM2		3.90E-18	-0.4	Lung
CADM2	rs62252497	1.70E-04	0.099	Nerve - Tibial
CADM2		2.40E-11	-0.28	Adipose - Subcutaneous
CADM2		6.90E-08	-0.24	Adipose - Visceral (Omentum)
CADM2		3.50E-06	-0.36	Heart - Left Ventricle
CADM2	rs62252499	2.70E-17	-0.4	Lung
CADM2		2.70E-12	-0.29	Adipose - Subcutaneous
CADM2		3.40E-08	-0.25	Adipose - Visceral (Omentum)
CADM2		8.10E-07	-0.38	Heart - Left Ventricle
CADM2	rs62252500	3.90E-18	-0.4	Lung
CADM2		2.80E-11	-0.28	Adipose - Subcutaneous
CADM2		7.60E-07	-0.23	Adipose - Visceral (Omentum)
CADM2		5.70E-07	-0.39	Heart - Left Ventricle
CADM2	rs62252501	4.90E-18	-0.4	Lung
CADM2		3.20E-12	-0.29	Adipose - Subcutaneous
CADM2		9.80E-09	-0.26	Adipose - Visceral (Omentum)
CADM2		7.40E-07	-0.39	Heart - Left Ventricle
CADM2		1.80E-18	-0.41	Lung
CADM2	rs62252503	1.50E-04	0.1	Nerve - Tibial
CADM2		1.00E-06	-0.21	Adipose - Subcutaneous
CADM2	rs62252504	1.80E-09	-0.27	Lung
CADM2		2.90E-12	-0.29	Adipose - Subcutaneous
CADM2		9.30E-09	-0.26	Adipose - Visceral (Omentum)
CADM2		7.40E-07	-0.39	Heart - Left Ventricle
CADM2		1.80E-18	-0.41	Lung
CADM2	rs62252505	1.50E-04	0.1	Nerve - Tibial
CADM2		1.90E-11	-0.28	Adipose - Subcutaneous
CADM2		1.50E-08	-0.25	Adipose - Visceral (Omentum)
CADM2		6.50E-07	-0.39	Heart - Left Ventricle
CADM2	rs62252506	1.80E-18	-0.41	Lung

CADM2		2.70E-11	-0.28	Adipose - Subcutaneous
CADM2		1.30E-08	-0.25	Adipose - Visceral (Omentum)
CADM2		8.00E-07	-0.39	Heart - Left Ventricle
CADM2		2.50E-18	-0.41	Lung
CADM2	rs62252507	1.60E-04	0.099	Nerve - Tibial
CADM2		1.00E-09	-0.25	Adipose - Subcutaneous
CADM2		1.00E-07	-0.24	Adipose - Visceral (Omentum)
CADM2		2.00E-07	-0.39	Heart - Left Ventricle
CADM2	rs62252508	8.80E-20	-0.41	Lung
CADM2		6.80E-12	-0.29	Adipose - Subcutaneous
CADM2		1.50E-08	-0.25	Adipose - Visceral (Omentum)
CADM2		7.40E-07	-0.39	Heart - Left Ventricle
CADM2		1.80E-18	-0.41	Lung
CADM2	rs62252509	1.60E-04	0.099	Nerve - Tibial
CADM2		1.10E-11	-0.28	Adipose - Subcutaneous
CADM2		3.60E-08	-0.25	Adipose - Visceral (Omentum)
CADM2		1.30E-06	-0.38	Heart - Left Ventricle
CADM2	rs62252512	5.70E-18	-0.4	Lung
CADM2		5.90E-12	-0.29	Adipose - Subcutaneous
CADM2		1.50E-08	-0.25	Adipose - Visceral (Omentum)
CADM2		9.90E-07	-0.39	Heart - Left Ventricle
CADM2		2.50E-18	-0.41	Lung
CADM2	rs62252513	1.60E-04	0.099	Nerve - Tibial
CADM2		1.20E-11	-0.28	Adipose - Subcutaneous
CADM2		6.50E-08	-0.24	Adipose - Visceral (Omentum)
CADM2		5.50E-07	-0.4	Heart - Left Ventricle
CADM2	rs62252516	2.40E-17	-0.4	Lung
CADM2	rs62252517	2.80E-06	0.34	Lung
CADM2		2.70E-12	-0.29	Adipose - Subcutaneous
CADM2		9.40E-09	-0.26	Adipose - Visceral (Omentum)
CADM2		7.00E-07	-0.39	Heart - Left Ventricle
CADM2		1.70E-18	-0.41	Lung
CADM2	rs62252518	1.50E-04	0.1	Nerve - Tibial
CADM2		3.20E-12	-0.29	Adipose - Subcutaneous
CADM2		2.00E-08	-0.25	Adipose - Visceral (Omentum)
CADM2		6.90E-07	-0.39	Heart - Left Ventricle
CADM2		1.80E-18	-0.41	Lung
CADM2	rs62252519	1.50E-04	0.1	Nerve - Tibial
CADM2	rs62253085	6.10E-06	-0.18	Adipose - Subcutaneous
CADM2	rs62253086	8.90E-05	0.27	Lung
CADM2		4.20E-06	-0.2	Adipose - Subcutaneous
CADM2		6.50E-05	-0.19	Adipose - Visceral (Omentum)
CADM2	rs62253088	7.40E-07	-0.25	Lung
CADM2		1.60E-09	-0.28	Adipose - Subcutaneous
CADM2		2.30E-07	-0.26	Adipose - Visceral (Omentum)
CADM2		4.40E-05	-0.21	Breast - Mammary Tissue
CADM2	rs62253107	3.80E-10	-0.33	Lung
CADM2		9.30E-07	-0.21	Adipose - Subcutaneous
CADM2	rs62253963	2.10E-09	-0.27	Lung
CADM2		3.20E-12	-0.29	Adipose - Subcutaneous
CADM2		9.80E-09	-0.26	Adipose - Visceral (Omentum)
CADM2	rs62253964	7.40E-07	-0.39	Heart - Left Ventricle

CADM2		1.80E-18	-0.41	Lung
CADM2		1.50E-04	0.1	Nerve - Tibial
CADM2		3.20E-12	-0.29	Adipose - Subcutaneous
CADM2		2.10E-08	-0.25	Adipose - Visceral (Omentum)
CADM2		4.70E-07	-0.4	Heart - Left Ventricle
CADM2		7.80E-18	-0.4	Lung
CADM2	rs62253965	1.50E-04	0.1	Nerve - Tibial
CADM2		6.60E-12	-0.29	Adipose - Subcutaneous
CADM2		1.50E-08	-0.25	Adipose - Visceral (Omentum)
CADM2		7.50E-07	-0.39	Heart - Left Ventricle
CADM2		2.30E-18	-0.41	Lung
CADM2	rs62253966	1.60E-04	0.099	Nerve - Tibial
CADM2		1.00E-09	-0.25	Adipose - Subcutaneous
CADM2		1.00E-07	-0.24	Adipose - Visceral (Omentum)
CADM2		2.00E-07	-0.39	Heart - Left Ventricle
CADM2	rs62253967	8.80E-20	-0.41	Lung
CADM2		1.00E-09	-0.25	Adipose - Subcutaneous
CADM2		1.00E-07	-0.24	Adipose - Visceral (Omentum)
CADM2		2.00E-07	-0.39	Heart - Left Ventricle
CADM2	rs62253968	8.80E-20	-0.41	Lung
CADM2		6.50E-12	-0.29	Adipose - Subcutaneous
CADM2		1.50E-08	-0.25	Adipose - Visceral (Omentum)
CADM2		7.50E-07	-0.39	Heart - Left Ventricle
CADM2		1.80E-18	-0.41	Lung
CADM2	rs62253969	1.70E-04	0.099	Nerve - Tibial
CADM2		6.80E-12	-0.29	Adipose - Subcutaneous
CADM2		1.60E-08	-0.25	Adipose - Visceral (Omentum)
CADM2		7.40E-07	-0.39	Heart - Left Ventricle
CADM2		1.80E-18	-0.41	Lung
CADM2	rs62253970	1.60E-04	0.099	Nerve - Tibial
CADM2		6.80E-12	-0.29	Adipose - Subcutaneous
CADM2		1.50E-08	-0.25	Adipose - Visceral (Omentum)
CADM2		7.40E-07	-0.39	Heart - Left Ventricle
CADM2		1.80E-18	-0.41	Lung
CADM2	rs62253971	1.60E-04	0.099	Nerve - Tibial
CADM2-AS1		1.40E-07	0.65	Brain - Caudate (basal ganglia)
CADM2-AS1		5.10E-11	0.75	Brain - Cerebellar Hemisphere
CADM2-AS1		6.70E-12	0.63	Brain - Cerebellum
CADM2-AS1		3.50E-07	0.58	Brain - Cortex
CADM2-AS1		1.50E-07	0.65	Brain - Frontal Cortex (BA9)
CADM2-AS1		1.60E-06	0.67	Brain - Hippocampus
CADM2-AS1		1.80E-09	0.71	Brain - Nucleus accumbens (basal ganglia)
CADM2-AS1	rs62261591	9.90E-05	0.25	Nerve - Tibial
CADM2-AS1		1.40E-07	0.65	Brain - Caudate (basal ganglia)
CADM2-AS1		5.10E-11	0.75	Brain - Cerebellar Hemisphere
CADM2-AS1		3.70E-12	0.64	Brain - Cerebellum
CADM2-AS1		2.60E-07	0.59	Brain - Cortex
CADM2-AS1		1.50E-07	0.65	Brain - Frontal Cortex (BA9)
CADM2-AS1		1.60E-06	0.67	Brain - Hippocampus
CADM2-AS1	rs62261592	1.80E-09	0.71	Brain - Nucleus accumbens (basal ganglia)

CADM2-AS1		9.10E-05	0.25	Nerve - Tibial
CADM2		7.30E-07	-0.23	Lung
CADM2-AS1	rs62261667	1.70E-05	0.28	Nerve - Tibial
CADM2	rs62261671	8.10E-05	0.27	Lung
CADM2-AS1	rs62261676	2.70E-05	0.61	Brain - Caudate (basal ganglia)
CADM2-AS1	rs62261700	6.60E-05	0.41	Brain - Cerebellum
CADM2-AS1		1.20E-07	0.63	Brain - Caudate (basal ganglia)
CADM2-AS1		9.30E-06	0.52	Brain - Cerebellar Hemisphere
CADM2-AS1	rs62261721	1.70E-08	0.55	Brain - Cerebellum
CADM2-AS1		6.60E-07	0.63	Brain - Caudate (basal ganglia)
CADM2-AS1		1.50E-06	0.59	Brain - Cerebellar Hemisphere
CADM2-AS1		4.80E-09	0.59	Brain - Cerebellum
CADM2-AS1	rs62261725	1.50E-05	0.3	Nerve - Tibial
CADM2-AS1		8.80E-07	0.6	Brain - Caudate (basal ganglia)
CADM2-AS1		4.60E-09	0.68	Brain - Cerebellar Hemisphere
CADM2-AS1		1.30E-10	0.62	Brain - Cerebellum
CADM2-AS1		2.10E-05	0.56	Brain - Hippocampus
CADM2-AS1	rs62261744	4.40E-06	0.54	Brain - Nucleus accumbens (basal ganglia)
CADM2-AS1		2.30E-05	0.56	Brain - Caudate (basal ganglia)
CADM2-AS1		4.90E-07	0.61	Brain - Cerebellar Hemisphere
CADM2-AS1		3.00E-07	0.52	Brain - Cerebellum
CADM2-AS1		2.90E-05	0.6	Brain - Hippocampus
CADM2-AS1		1.70E-05	0.54	Brain - Nucleus accumbens (basal ganglia)
CADM2-AS1	rs62261746	6.50E-06	0.32	Nerve - Tibial
CADM2-AS1		3.50E-07	0.61	Brain - Caudate (basal ganglia)
CADM2-AS1		9.10E-10	0.69	Brain - Cerebellar Hemisphere
CADM2-AS1		1.10E-09	0.57	Brain - Cerebellum
CADM2-AS1		1.30E-05	0.53	Brain - Frontal Cortex (BA9)
CADM2-AS1		2.20E-05	0.56	Brain - Hippocampus
CADM2-AS1	rs62261747	1.00E-06	0.57	Brain - Nucleus accumbens (basal ganglia)
CADM2-AS1		9.70E-07	0.58	Brain - Caudate (basal ganglia)
CADM2-AS1		1.10E-09	0.69	Brain - Cerebellar Hemisphere
CADM2-AS1		2.10E-10	0.59	Brain - Cerebellum
CADM2-AS1		1.60E-05	0.52	Brain - Frontal Cortex (BA9)
CADM2-AS1		3.00E-05	0.55	Brain - Hippocampus
CADM2-AS1		9.60E-07	0.57	Brain - Nucleus accumbens (basal ganglia)
CADM2-AS1	rs62261750	9.60E-05	0.25	Nerve - Tibial
CADM2-AS1		6.30E-07	0.62	Brain - Caudate (basal ganglia)
CADM2-AS1		2.50E-10	0.74	Brain - Cerebellar Hemisphere
CADM2-AS1		1.80E-12	0.65	Brain - Cerebellum
CADM2-AS1		1.50E-06	0.56	Brain - Cortex
CADM2-AS1		5.10E-07	0.64	Brain - Frontal Cortex (BA9)
CADM2-AS1		1.60E-06	0.67	Brain - Hippocampus
CADM2-AS1		9.70E-09	0.68	Brain - Nucleus accumbens (basal ganglia)
CADM2-AS1	rs62263319	1.80E-05	0.57	Brain - Putamen (basal ganglia)
CADM2-AS1	rs62263322	9.20E-07	0.61	Brain - Caudate (basal ganglia)

CADM2-AS1		5.30E-10	0.73	Brain - Cerebellar Hemisphere
CADM2-AS1		9.40E-12	0.64	Brain - Cerebellum
CADM2-AS1		5.50E-06	0.54	Brain - Cortex
CADM2-AS1		9.30E-07	0.63	Brain - Frontal Cortex (BA9)
CADM2-AS1		1.60E-06	0.67	Brain - Hippocampus
CADM2-AS1		1.20E-08	0.67	Brain - Nucleus accumbens (basal ganglia)
CADM2-AS1		1.30E-05	0.58	Brain - Putamen (basal ganglia)
CADM2-AS1		6.80E-06	0.57	Brain - Caudate (basal ganglia)
CADM2-AS1		3.40E-10	0.73	Brain - Cerebellar Hemisphere
CADM2-AS1		7.60E-12	0.64	Brain - Cerebellum
CADM2-AS1		1.60E-06	0.55	Brain - Cortex
CADM2-AS1		1.50E-06	0.62	Brain - Frontal Cortex (BA9)
CADM2-AS1		7.90E-07	0.68	Brain - Hippocampus
CADM2-AS1		1.60E-08	0.67	Brain - Nucleus accumbens (basal ganglia)
CADM2-AS1	rs62263325	9.60E-06	0.59	Brain - Putamen (basal ganglia)
CADM2-AS1		2.40E-06	0.58	Brain - Caudate (basal ganglia)
CADM2-AS1		4.90E-08	0.64	Brain - Cerebellar Hemisphere
CADM2-AS1		1.00E-09	0.55	Brain - Cerebellum
CADM2-AS1		5.80E-06	0.51	Brain - Cortex
CADM2-AS1		3.50E-06	0.62	Brain - Frontal Cortex (BA9)
CADM2-AS1		1.30E-05	0.59	Brain - Hippocampus
CADM2-AS1	rs62263332	1.90E-06	0.59	Brain - Nucleus accumbens (basal ganglia)
CADM2		7.40E-12	-0.29	Adipose - Subcutaneous
CADM2		4.80E-08	-0.25	Adipose - Visceral (Omentum)
CADM2		1.20E-06	-0.38	Heart - Left Ventricle
CADM2	rs62263910	1.00E-17	-0.4	Lung
CADM2		2.00E-11	-0.28	Adipose - Subcutaneous
CADM2		3.00E-07	-0.23	Adipose - Visceral (Omentum)
CADM2		7.90E-07	-0.39	Heart - Left Ventricle
CADM2	rs62263912	2.00E-17	-0.4	Lung
CADM2		3.30E-06	-0.22	Lung
CADM2-AS1	rs62263913	2.30E-05	0.27	Nerve - Tibial
CADM2	rs62263914	5.00E-06	-0.21	Lung
CADM2		3.80E-05	-0.18	Adipose - Subcutaneous
CADM2	rs62263915	4.60E-11	-0.33	Lung
CADM2		1.30E-06	-0.21	Adipose - Subcutaneous
CADM2		7.50E-05	-0.18	Adipose - Visceral (Omentum)
CADM2	rs62263916	5.50E-11	-0.32	Lung
CADM2		6.50E-05	-0.17	Adipose - Subcutaneous
CADM2	rs62263917	4.00E-12	-0.33	Lung
CADM2		4.60E-06	-0.2	Adipose - Subcutaneous
CADM2		7.70E-05	-0.18	Adipose - Visceral (Omentum)
CADM2	rs62263918	3.40E-11	-0.33	Lung
CADM2		1.90E-06	-0.23	Lung
CADM2-AS1	rs62263923	9.00E-06	0.29	Nerve - Tibial
CADM2	rs62263924	1.10E-04	0.27	Lung
CADM2		1.10E-04	-0.18	Adipose - Subcutaneous
CADM2		8.20E-06	-0.22	Lung
CADM2-AS1	rs62263929	5.30E-05	0.27	Nerve - Tibial

CADM2	rs649851	7.30E-05	-0.14	Muscle - Skeletal
CADM2	rs653481	3.10E-06	0.18	Muscle - Skeletal
CADM2	rs6549007	1.50E-04	0.16	Muscle - Skeletal
CADM2		9.60E-05	0.17	Adipose - Subcutaneous
CADM2	rs6549009	4.00E-07	0.19	Muscle - Skeletal
CADM2		1.80E-05	0.16	Adipose - Subcutaneous
CADM2	rs6549011	3.30E-05	0.14	Muscle - Skeletal
CADM2		1.70E-05	0.16	Adipose - Subcutaneous
CADM2	rs6549012	3.20E-05	0.14	Muscle - Skeletal
CADM2	rs6549022	1.80E-05	-0.73	Testis
CADM2	rs6549023	2.30E-07	-0.34	Lung
CADM2		2.00E-08	0.26	Adipose - Subcutaneous
CADM2		1.90E-05	0.22	Adipose - Visceral (Omentum)
CADM2	rs6549024	7.40E-07	0.28	Lung
CADM2		1.50E-05	-0.46	Heart - Left Ventricle
CADM2	rs6549027	8.10E-07	-0.32	Lung
CADM2		2.10E-09	-0.25	Adipose - Subcutaneous
CADM2		6.70E-08	-0.24	Adipose - Visceral (Omentum)
CADM2		1.10E-07	-0.4	Heart - Left Ventricle
CADM2	rs6549030	5.00E-19	-0.41	Lung
CADM2	rs6549031	6.30E-08	-0.35	Lung
CADM2		3.70E-11	-0.28	Adipose - Subcutaneous
CADM2		1.10E-07	-0.24	Adipose - Visceral (Omentum)
CADM2		8.40E-07	-0.39	Heart - Left Ventricle
CADM2	rs6549033	2.90E-18	-0.41	Lung
CADM2		6.80E-12	-0.29	Adipose - Subcutaneous
CADM2		1.50E-08	-0.25	Adipose - Visceral (Omentum)
CADM2		7.40E-07	-0.39	Heart - Left Ventricle
CADM2		1.80E-18	-0.41	Lung
CADM2	rs6549034	1.60E-04	0.099	Nerve - Tibial
CADM2		7.10E-10	-0.25	Adipose - Subcutaneous
CADM2		7.00E-08	-0.25	Adipose - Visceral (Omentum)
CADM2		2.10E-07	-0.4	Heart - Left Ventricle
CADM2	rs6549035	5.10E-19	-0.41	Lung
CADM2		6.80E-12	-0.29	Adipose - Subcutaneous
CADM2		1.70E-08	-0.25	Adipose - Visceral (Omentum)
CADM2		7.40E-07	-0.39	Heart - Left Ventricle
CADM2		1.80E-18	-0.41	Lung
CADM2	rs6549036	1.40E-04	0.1	Nerve - Tibial
CADM2		6.80E-12	-0.29	Adipose - Subcutaneous
CADM2		1.50E-08	-0.25	Adipose - Visceral (Omentum)
CADM2		7.40E-07	-0.39	Heart - Left Ventricle
CADM2		1.80E-18	-0.41	Lung
CADM2	rs6549037	1.60E-04	0.099	Nerve - Tibial
CADM2	rs6549038	6.30E-08	-0.35	Lung
CADM2		8.90E-12	-0.29	Adipose - Subcutaneous
CADM2		9.00E-08	-0.24	Adipose - Visceral (Omentum)
CADM2		4.40E-07	-0.4	Heart - Left Ventricle
CADM2		3.00E-17	-0.4	Lung
CADM2	rs6549039	9.40E-05	0.1	Nerve - Tibial
CADM2	rs6549040	8.50E-12	-0.29	Adipose - Subcutaneous

CADM2		7.70E-08	-0.24	Adipose - Visceral (Omentum)
CADM2		4.70E-07	-0.4	Heart - Left Ventricle
CADM2		1.50E-17	-0.4	Lung
CADM2		1.20E-04	0.1	Nerve - Tibial
CADM2		1.00E-11	-0.28	Adipose - Subcutaneous
CADM2		2.40E-08	-0.25	Adipose - Visceral (Omentum)
CADM2		1.50E-06	-0.38	Heart - Left Ventricle
CADM2	rs6549041	3.40E-18	-0.4	Lung
CADM2		2.60E-11	-0.28	Adipose - Subcutaneous
CADM2		4.50E-08	-0.25	Adipose - Visceral (Omentum)
CADM2		1.30E-06	-0.38	Heart - Left Ventricle
CADM2	rs6549042	3.20E-18	-0.4	Lung
CADM2		3.10E-11	-0.28	Adipose - Subcutaneous
CADM2		4.30E-08	-0.24	Adipose - Visceral (Omentum)
CADM2		2.10E-06	-0.37	Heart - Left Ventricle
CADM2	rs6549043	3.70E-18	-0.4	Lung
CADM2		2.00E-11	-0.28	Adipose - Subcutaneous
CADM2		4.30E-08	-0.24	Adipose - Visceral (Omentum)
CADM2		1.30E-06	-0.38	Heart - Left Ventricle
CADM2	rs6549044	2.40E-18	-0.41	Lung
CADM2		9.50E-06	-0.19	Adipose - Subcutaneous
CADM2	rs6549045	1.30E-10	-0.32	Lung
CADM2		2.30E-06	0.2	Adipose - Subcutaneous
CADM2		4.30E-05	0.19	Adipose - Visceral (Omentum)
CADM2	rs6549046	1.00E-10	0.32	Lung
CADM2-AS1	rs6549048	3.00E-05	-0.47	Brain - Nucleus accumbens (basal ganglia)
CADM2-AS1		2.70E-06	0.53	Brain - Cerebellar Hemisphere
CADM2-AS1		1.80E-08	0.51	Brain - Cerebellum
CADM2-AS1	rs6549053	1.90E-05	0.51	Brain - Nucleus accumbens (basal ganglia)
CADM2-AS1		2.90E-06	0.53	Brain - Cerebellar Hemisphere
CADM2-AS1		1.20E-08	0.51	Brain - Cerebellum
CADM2-AS1	rs6549054	2.70E-05	0.5	Brain - Nucleus accumbens (basal ganglia)
CADM2-AS1		2.20E-06	0.55	Brain - Caudate (basal ganglia)
CADM2-AS1		3.50E-05	0.47	Brain - Cerebellar Hemisphere
CADM2-AS1		3.80E-07	0.48	Brain - Cerebellum
CADM2-AS1	rs6549055	3.40E-06	0.52	Brain - Nucleus accumbens (basal ganglia)
CADM2-AS1		2.20E-06	0.54	Brain - Cerebellar Hemisphere
CADM2-AS1		3.80E-09	0.53	Brain - Cerebellum
CADM2-AS1	rs6549056	2.70E-05	0.5	Brain - Nucleus accumbens (basal ganglia)
CADM2-AS1		4.80E-07	0.58	Brain - Caudate (basal ganglia)
CADM2-AS1		1.70E-05	0.49	Brain - Cerebellar Hemisphere
CADM2-AS1	rs6549058	1.20E-08	0.53	Brain - Cerebellum
CADM2-AS1		6.00E-06	-0.51	Brain - Cerebellar Hemisphere
CADM2-AS1		1.80E-05	-0.46	Brain - Cortex
CADM2-AS1		1.90E-05	-0.53	Brain - Frontal Cortex (BA9)
CADM2-AS1	rs6549061	1.40E-05	-0.48	Brain - Nucleus accumbens (basal ganglia)
CADM2-AS1		4.00E-05	0.53	Brain - Caudate (basal ganglia)
CADM2-AS1		2.20E-07	0.62	Brain - Cerebellar Hemisphere
CADM2-AS1	rs6549063	9.70E-08	0.49	Brain - Cerebellum

CADM2-AS1		2.60E-05	0.47	Brain - Cortex
CADM2-AS1		8.70E-07	0.6	Brain - Frontal Cortex (BA9)
CADM2-AS1		5.70E-06	0.53	Brain - Nucleus accumbens (basal ganglia)
CADM2-AS1		2.30E-05	0.54	Brain - Caudate (basal ganglia)
CADM2-AS1		1.30E-06	0.58	Brain - Cerebellar Hemisphere
CADM2-AS1		5.50E-08	0.5	Brain - Cerebellum
CADM2-AS1		2.80E-05	0.46	Brain - Cortex
CADM2-AS1		4.10E-05	0.51	Brain - Frontal Cortex (BA9)
CADM2-AS1		1.60E-06	0.56	Brain - Nucleus accumbens (basal ganglia)
CADM2-AS1	rs6549064	3.70E-05	0.58	Brain - Putamen (basal ganglia)
CADM2-AS1		2.30E-05	0.54	Brain - Caudate (basal ganglia)
CADM2-AS1		1.30E-06	0.58	Brain - Cerebellar Hemisphere
CADM2-AS1		3.70E-08	0.5	Brain - Cerebellum
CADM2-AS1		1.60E-05	0.47	Brain - Cortex
CADM2-AS1		4.10E-05	0.51	Brain - Frontal Cortex (BA9)
CADM2-AS1		1.60E-06	0.56	Brain - Nucleus accumbens (basal ganglia)
CADM2-AS1	rs6549065	3.70E-05	0.58	Brain - Putamen (basal ganglia)
CADM2-AS1		1.50E-05	0.67	Brain - Cerebellar Hemisphere
CADM2-AS1		1.10E-05	0.56	Brain - Cortex
CADM2-AS1	rs6549068	3.00E-06	0.64	Brain - Nucleus accumbens (basal ganglia)
CADM2		1.10E-11	-0.29	Adipose - Subcutaneous
CADM2		2.80E-08	-0.25	Adipose - Visceral (Omentum)
CADM2		8.60E-07	-0.39	Heart - Left Ventricle
CADM2		2.60E-16	-0.39	Lung
CADM2	rs66499081	1.50E-04	0.1	Nerve - Tibial
CADM2		4.70E-05	0.16	Adipose - Subcutaneous
CADM2	rs66500121	6.30E-06	0.16	Muscle - Skeletal
CADM2		4.00E-12	-0.29	Adipose - Subcutaneous
CADM2		1.80E-08	-0.25	Adipose - Visceral (Omentum)
CADM2		6.20E-07	-0.39	Heart - Left Ventricle
CADM2	rs66505109	4.30E-17	-0.39	Lung
CADM2	rs66516423	1.30E-05	-0.21	Lung
CADM2		9.10E-11	-0.27	Adipose - Subcutaneous
CADM2		5.00E-07	-0.23	Adipose - Visceral (Omentum)
CADM2		1.40E-07	-0.41	Heart - Left Ventricle
CADM2		2.50E-17	-0.4	Lung
CADM2	rs66544515	7.50E-05	0.1	Nerve - Tibial
CADM2		7.60E-12	-0.29	Adipose - Subcutaneous
CADM2		3.30E-08	-0.25	Adipose - Visceral (Omentum)
CADM2		1.40E-06	-0.38	Heart - Left Ventricle
CADM2		1.60E-18	-0.41	Lung
CADM2	rs66625173	1.80E-04	0.098	Nerve - Tibial
CADM2		6.10E-12	-0.29	Adipose - Subcutaneous
CADM2		2.80E-08	-0.25	Adipose - Visceral (Omentum)
CADM2		5.20E-07	-0.4	Heart - Left Ventricle
CADM2		1.70E-18	-0.41	Lung
CADM2	rs66631011	1.30E-04	0.1	Nerve - Tibial
CADM2		2.10E-11	-0.28	Adipose - Subcutaneous
CADM2	rs66632973	3.40E-08	-0.25	Adipose - Visceral (Omentum)

CADM2		3.30E-06	-0.37	Heart - Left Ventricle
CADM2		1.30E-18	-0.41	Lung
CADM2		4.00E-06	-0.22	Adipose - Subcutaneous
CADM2	rs66653632	2.00E-05	-0.23	Lung
CADM2-AS1		5.20E-07	0.62	Brain - Caudate (basal ganglia)
CADM2-AS1		4.50E-09	0.69	Brain - Cerebellar Hemisphere
CADM2-AS1		1.50E-10	0.62	Brain - Cerebellum
CADM2-AS1		1.60E-05	0.53	Brain - Frontal Cortex (BA9)
CADM2-AS1		1.20E-07	0.62	Brain - Nucleus accumbens (basal ganglia)
CADM2-AS1	rs66680800	7.70E-05	0.25	Nerve - Tibial
CADM2		5.80E-05	0.18	Adipose - Subcutaneous
CADM2		3.90E-07	0.74	Brain - Spinal cord (cervical c-1)
CADM2	rs66701594	3.10E-05	0.16	Muscle - Skeletal
CADM2		3.40E-11	-0.28	Adipose - Subcutaneous
CADM2		1.50E-07	-0.24	Adipose - Visceral (Omentum)
CADM2		3.60E-07	-0.41	Heart - Left Ventricle
CADM2	rs66781790	1.60E-17	-0.4	Lung
CADM2	rs66853163	2.30E-05	-0.19	Muscle - Skeletal
CADM2	rs66855166	8.40E-05	-0.18	Muscle - Skeletal
CADM2		4.50E-05	0.18	Adipose - Subcutaneous
CADM2		2.60E-05	0.26	Brain - Caudate (basal ganglia)
CADM2		7.80E-07	0.69	Brain - Spinal cord (cervical c-1)
CADM2	rs66887106	1.70E-05	0.17	Muscle - Skeletal
CADM2		8.20E-12	-0.29	Adipose - Subcutaneous
CADM2		1.70E-08	-0.25	Adipose - Visceral (Omentum)
CADM2		8.60E-07	-0.39	Heart - Left Ventricle
CADM2		3.20E-18	-0.4	Lung
CADM2	rs66922686	1.70E-04	0.099	Nerve - Tibial
CADM2		2.70E-06	-0.2	Adipose - Subcutaneous
CADM2		1.10E-09	-0.28	Lung
CADM2-AS1	rs66963835	7.60E-05	0.26	Nerve - Tibial
CADM2		6.80E-12	-0.29	Adipose - Subcutaneous
CADM2		1.50E-08	-0.25	Adipose - Visceral (Omentum)
CADM2		7.40E-07	-0.39	Heart - Left Ventricle
CADM2		1.80E-18	-0.41	Lung
CADM2	rs67018424	1.60E-04	0.099	Nerve - Tibial
CADM2		2.60E-06	-0.2	Adipose - Subcutaneous
CADM2		3.30E-06	-0.23	Lung
CADM2	rs67028245	1.20E-04	-0.15	Muscle - Skeletal
CADM2		4.90E-05	0.18	Adipose - Subcutaneous
CADM2	rs67033670	2.10E-06	0.7	Brain - Spinal cord (cervical c-1)
CADM2	rs67219198	9.50E-07	-0.24	Lung
CADM2		1.30E-11	-0.28	Adipose - Subcutaneous
CADM2		2.60E-07	-0.24	Adipose - Visceral (Omentum)
CADM2		1.30E-06	-0.38	Heart - Left Ventricle
CADM2	rs67277278	4.10E-18	-0.41	Lung
CADM2		8.60E-12	-0.29	Adipose - Subcutaneous
CADM2		2.80E-08	-0.25	Adipose - Visceral (Omentum)
CADM2		3.70E-07	-0.4	Heart - Left Ventricle
CADM2	rs67336646	9.20E-18	-0.4	Lung
CADM2	rs67391933	3.70E-05	-0.17	Adipose - Subcutaneous

CADM2		9.40E-05	-0.31	Heart - Left Ventricle
CADM2		1.10E-12	-0.34	Lung
CADM2		2.10E-09	-0.25	Adipose - Subcutaneous
CADM2		1.10E-07	-0.24	Adipose - Visceral (Omentum)
CADM2		1.40E-07	-0.4	Heart - Left Ventricle
CADM2		3.10E-19	-0.41	Lung
CADM2		7.60E-12	-0.29	Adipose - Subcutaneous
CADM2		3.50E-08	-0.25	Adipose - Visceral (Omentum)
CADM2		1.10E-06	-0.39	Heart - Left Ventricle
CADM2		5.20E-18	-0.4	Lung
CADM2	rs67416405	1.50E-04	0.1	Nerve - Tibial
CADM2		1.70E-11	-0.28	Adipose - Subcutaneous
CADM2		1.30E-08	-0.25	Adipose - Visceral (Omentum)
CADM2		6.00E-07	-0.39	Heart - Left Ventricle
CADM2		2.90E-18	-0.41	Lung
CADM2	rs67568006	1.50E-04	0.1	Nerve - Tibial
CADM2		1.00E-09	-0.25	Adipose - Subcutaneous
CADM2		2.30E-07	-0.23	Adipose - Visceral (Omentum)
CADM2		1.80E-07	-0.4	Heart - Left Ventricle
CADM2	rs67584463	1.10E-19	-0.42	Lung
CADM2	rs6762253	3.30E-05	-0.67	Heart - Left Ventricle
CADM2		2.90E-10	-0.27	Adipose - Subcutaneous
CADM2		5.60E-08	-0.25	Adipose - Visceral (Omentum)
CADM2		4.30E-07	-0.41	Heart - Left Ventricle
CADM2	rs6762267	4.60E-17	-0.4	Lung
CADM2		2.20E-11	-0.28	Adipose - Subcutaneous
CADM2		4.50E-08	-0.24	Adipose - Visceral (Omentum)
CADM2		1.40E-06	-0.38	Heart - Left Ventricle
CADM2	rs6762535	3.20E-18	-0.4	Lung
CADM2		4.60E-11	-0.28	Adipose - Subcutaneous
CADM2		3.60E-07	-0.23	Adipose - Visceral (Omentum)
CADM2		4.10E-07	-0.4	Heart - Left Ventricle
CADM2	rs6762695	8.40E-15	-0.37	Lung
CADM2		2.40E-11	-0.28	Adipose - Subcutaneous
CADM2		4.50E-08	-0.24	Adipose - Visceral (Omentum)
CADM2		1.40E-06	-0.38	Heart - Left Ventricle
CADM2	rs6762733	4.00E-18	-0.4	Lung
CADM2	rs6762830	1.60E-05	-0.19	Muscle - Skeletal
CADM2-AS1		2.60E-05	0.64	Brain - Anterior cingulate cortex (BA24)
CADM2-AS1		1.90E-06	0.6	Brain - Cerebellar Hemisphere
CADM2-AS1		9.40E-09	0.54	Brain - Cerebellum
CADM2-AS1		1.20E-05	0.55	Brain - Frontal Cortex (BA9)
CADM2-AS1	rs6762937	6.40E-07	0.6	Brain - Nucleus accumbens (basal ganglia)
CADM2		2.90E-07	0.67	Brain - Spinal cord (cervical c-1)
CADM2	rs6764277	3.70E-05	0.17	Muscle - Skeletal
CADM2		1.20E-04	0.17	Adipose - Subcutaneous
CADM2	rs6764381	5.70E-07	0.64	Brain - Spinal cord (cervical c-1)
CADM2		1.20E-04	0.17	Adipose - Subcutaneous
CADM2	rs6764547	5.70E-07	0.64	Brain - Spinal cord (cervical c-1)
CADM2		4.10E-12	-0.29	Adipose - Subcutaneous
CADM2		3.20E-08	-0.25	Adipose - Visceral (Omentum)
CADM2	rs6764988	1.10E-06	-0.38	Heart - Left Ventricle

CADM2		4.20E-18	-0.41	Lung
CADM2		1.40E-05	0.16	Adipose - Subcutaneous
CADM2	rs6765011	3.60E-05	0.14	Muscle - Skeletal
CADM2-AS1		1.60E-06	0.53	Brain - Cerebellar Hemisphere
CADM2-AS1		1.20E-05	0.43	Brain - Cerebellum
CADM2-AS1	rs6765645	3.10E-06	0.53	Brain - Nucleus accumbens (basal ganglia)
CADM2		9.30E-12	-0.29	Adipose - Subcutaneous
CADM2		9.50E-09	-0.26	Adipose - Visceral (Omentum)
CADM2		2.30E-06	-0.37	Heart - Left Ventricle
CADM2	rs6765881	3.80E-18	-0.41	Lung
CADM2		5.90E-12	-0.29	Adipose - Subcutaneous
CADM2		1.70E-08	-0.25	Adipose - Visceral (Omentum)
CADM2		1.40E-06	-0.38	Heart - Left Ventricle
CADM2	rs6765991	1.20E-18	-0.41	Lung
CADM2		5.60E-12	-0.29	Adipose - Subcutaneous
CADM2		3.10E-08	-0.25	Adipose - Visceral (Omentum)
CADM2		6.90E-07	-0.39	Heart - Left Ventricle
CADM2		1.80E-18	-0.41	Lung
CADM2	rs6766493	1.60E-04	0.099	Nerve - Tibial
CADM2		1.60E-11	-0.28	Adipose - Subcutaneous
CADM2		2.50E-08	-0.25	Adipose - Visceral (Omentum)
CADM2		2.70E-06	-0.37	Heart - Left Ventricle
CADM2	rs6766690	7.60E-18	-0.4	Lung
CADM2	rs6767407	1.10E-05	-0.19	Muscle - Skeletal
CADM2		1.40E-04	0.16	Adipose - Subcutaneous
CADM2	rs6768455	2.60E-05	0.15	Muscle - Skeletal
CADM2		6.50E-05	0.16	Adipose - Subcutaneous
CADM2	rs6768559	5.60E-07	0.18	Muscle - Skeletal
CADM2-AS1		3.70E-05	0.54	Brain - Caudate (basal ganglia)
CADM2-AS1		2.90E-08	0.67	Brain - Cerebellar Hemisphere
CADM2-AS1		5.70E-08	0.51	Brain - Cerebellum
CADM2-AS1		1.00E-05	0.5	Brain - Cortex
CADM2-AS1		5.20E-07	0.62	Brain - Frontal Cortex (BA9)
CADM2-AS1	rs6769012	2.40E-06	0.55	Brain - Nucleus accumbens (basal ganglia)
CADM2		3.40E-05	0.19	Adipose - Subcutaneous
CADM2		1.20E-06	0.6	Brain - Spinal cord (cervical c-1)
CADM2	rs6770276	1.90E-06	0.19	Muscle - Skeletal
CADM2		6.50E-06	0.21	Adipose - Subcutaneous
CADM2		1.10E-08	0.71	Brain - Spinal cord (cervical c-1)
CADM2	rs6771804	3.20E-06	0.19	Muscle - Skeletal
CADM2		7.10E-05	-0.43	Heart - Left Ventricle
CADM2	rs6772750	2.70E-07	-0.33	Lung
CADM2		6.30E-06	-0.48	Heart - Left Ventricle
CADM2	rs6772890	2.60E-06	-0.31	Lung
CADM2		2.20E-11	-0.28	Adipose - Subcutaneous
CADM2		4.30E-08	-0.24	Adipose - Visceral (Omentum)
CADM2		1.30E-06	-0.38	Heart - Left Ventricle
CADM2	rs6772956	3.00E-18	-0.4	Lung
CADM2		2.70E-08	0.26	Adipose - Subcutaneous
CADM2		2.10E-05	0.22	Adipose - Visceral (Omentum)
CADM2	rs6772991	8.50E-07	0.27	Lung

CADM2		2.30E-11	-0.28	Adipose - Subcutaneous
CADM2		5.50E-08	-0.24	Adipose - Visceral (Omentum)
CADM2		2.70E-07	-0.4	Heart - Left Ventricle
CADM2		4.60E-16	-0.39	Lung
CADM2	rs6774985	1.70E-04	-0.14	Muscle - Skeletal
CADM2-AS1		3.90E-05	0.56	Brain - Caudate (basal ganglia)
CADM2-AS1		2.10E-07	0.68	Brain - Cerebellar Hemisphere
CADM2-AS1		1.50E-07	0.52	Brain - Cerebellum
CADM2-AS1		2.10E-05	0.51	Brain - Cortex
CADM2-AS1		5.90E-07	0.66	Brain - Frontal Cortex (BA9)
CADM2-AS1	rs6775290	2.60E-06	0.57	Brain - Nucleus accumbens (basal ganglia)
CADM2		8.00E-05	0.15	Adipose - Subcutaneous
CADM2	rs6775464	4.40E-05	0.14	Muscle - Skeletal
CADM2		7.40E-05	0.15	Adipose - Subcutaneous
CADM2	rs6775752	6.40E-06	0.16	Muscle - Skeletal
CADM2		5.30E-09	-0.27	Adipose - Subcutaneous
CADM2		1.20E-06	-0.24	Adipose - Visceral (Omentum)
CADM2		6.10E-05	-0.21	Breast - Mammary Tissue
CADM2		4.60E-05	-0.35	Heart - Left Ventricle
CADM2	rs6776114	7.50E-10	-0.33	Lung
CADM2		3.20E-12	-0.29	Adipose - Subcutaneous
CADM2		9.80E-09	-0.26	Adipose - Visceral (Omentum)
CADM2		7.40E-07	-0.39	Heart - Left Ventricle
CADM2		1.80E-18	-0.41	Lung
CADM2	rs6777456	1.50E-04	0.1	Nerve - Tibial
CADM2		3.20E-12	-0.29	Adipose - Subcutaneous
CADM2		9.80E-09	-0.26	Adipose - Visceral (Omentum)
CADM2		7.40E-07	-0.39	Heart - Left Ventricle
CADM2		1.80E-18	-0.41	Lung
CADM2	rs6777458	1.50E-04	0.1	Nerve - Tibial
CADM2		2.90E-07	0.67	Brain - Spinal cord (cervical c-1)
CADM2	rs6777505	3.70E-05	0.17	Muscle - Skeletal
CADM2		8.70E-06	-0.47	Heart - Left Ventricle
CADM2	rs6778189	1.40E-06	-0.31	Lung
CADM2	rs6778223	7.70E-08	-0.35	Lung
CADM2		3.20E-12	-0.29	Adipose - Subcutaneous
CADM2		2.00E-08	-0.25	Adipose - Visceral (Omentum)
CADM2		6.90E-07	-0.39	Heart - Left Ventricle
CADM2		1.80E-18	-0.41	Lung
CADM2	rs6779501	1.50E-04	0.1	Nerve - Tibial
CADM2		5.00E-06	-0.19	Adipose - Subcutaneous
CADM2	rs6779752	4.20E-10	-0.31	Lung
CADM2		7.40E-11	-0.27	Adipose - Subcutaneous
CADM2		2.60E-08	-0.25	Adipose - Visceral (Omentum)
CADM2		2.50E-07	-0.4	Heart - Left Ventricle
CADM2		4.50E-19	-0.41	Lung
CADM2	rs6780346	1.40E-04	0.1	Nerve - Tibial
CADM2		1.40E-10	-0.27	Adipose - Subcutaneous
CADM2		3.30E-08	-0.24	Adipose - Visceral (Omentum)
CADM2		2.10E-07	-0.4	Heart - Left Ventricle
CADM2	rs6780968	1.20E-14	-0.36	Lung

CADM2		1.10E-04	-0.14	Muscle - Skeletal
CADM2		9.70E-06	0.32	Brain - Hippocampus
CADM2	rs67813378	1.10E-04	0.14	Muscle - Skeletal
CADM2-AS1		3.70E-05	0.55	Brain - Caudate (basal ganglia)
CADM2-AS1		1.60E-07	0.65	Brain - Cerebellar Hemisphere
CADM2-AS1		7.20E-08	0.52	Brain - Cerebellum
CADM2-AS1		1.60E-05	0.49	Brain - Cortex
CADM2-AS1		1.10E-06	0.61	Brain - Frontal Cortex (BA9)
CADM2-AS1	rs6781407	1.30E-06	0.57	Brain - Nucleus accumbens (basal ganglia)
CADM2	rs6781515	2.30E-07	-0.34	Lung
CADM2-AS1	rs6781999	3.00E-05	-0.47	Brain - Nucleus accumbens (basal ganglia)
CADM2		3.60E-09	-0.24	Adipose - Subcutaneous
CADM2		4.80E-07	-0.23	Adipose - Visceral (Omentum)
CADM2		6.00E-07	-0.38	Heart - Left Ventricle
CADM2	rs6782190	1.50E-19	-0.41	Lung
CADM2		6.40E-07	-0.19	Adipose - Subcutaneous
CADM2		1.10E-07	-0.25	Lung
CADM2	rs67824428	1.50E-05	-0.15	Muscle - Skeletal
CADM2-AS1		3.70E-05	0.55	Brain - Caudate (basal ganglia)
CADM2-AS1		1.60E-07	0.65	Brain - Cerebellar Hemisphere
CADM2-AS1		7.20E-08	0.52	Brain - Cerebellum
CADM2-AS1		6.00E-06	0.51	Brain - Cortex
CADM2-AS1		1.10E-06	0.61	Brain - Frontal Cortex (BA9)
CADM2-AS1	rs6782553	1.30E-06	0.57	Brain - Nucleus accumbens (basal ganglia)
CADM2	rs6782644	4.80E-05	0.68	Spleen
CADM2-AS1	rs6782780	6.40E-05	-0.28	Nerve - Tibial
CADM2		9.70E-12	-0.28	Adipose - Subcutaneous
CADM2		1.90E-08	-0.25	Adipose - Visceral (Omentum)
CADM2		7.40E-07	-0.39	Heart - Left Ventricle
CADM2	rs6783137	5.50E-18	-0.4	Lung
CADM2		9.70E-12	-0.28	Adipose - Subcutaneous
CADM2		1.90E-08	-0.25	Adipose - Visceral (Omentum)
CADM2		7.40E-07	-0.39	Heart - Left Ventricle
CADM2	rs6783138	5.50E-18	-0.4	Lung
CADM2	rs6785531	2.30E-05	-0.2	Lung
CADM2		2.30E-06	-0.2	Adipose - Subcutaneous
CADM2	rs67874038	1.70E-09	-0.28	Lung
CADM2		4.80E-06	-0.2	Adipose - Subcutaneous
CADM2		3.80E-09	-0.27	Lung
CADM2-AS1	rs67874563	1.10E-04	0.26	Nerve - Tibial
CADM2-AS1		1.50E-07	0.62	Brain - Caudate (basal ganglia)
CADM2-AS1		2.80E-06	0.54	Brain - Cerebellar Hemisphere
CADM2-AS1		3.00E-08	0.53	Brain - Cerebellum
CADM2-AS1	rs6787851	4.30E-05	0.46	Brain - Cortex
CADM2-AS1		3.00E-05	0.54	Brain - Caudate (basal ganglia)
CADM2-AS1		2.40E-08	0.65	Brain - Cerebellar Hemisphere
CADM2-AS1		1.90E-07	0.49	Brain - Cerebellum
CADM2-AS1		4.70E-06	0.51	Brain - Cortex
CADM2-AS1	rs6787901	1.00E-05	0.54	Brain - Frontal Cortex (BA9)

CADM2-AS1		6.80E-07	0.58	Brain - Nucleus accumbens (basal ganglia)
CADM2		2.10E-11	-0.28	Adipose - Subcutaneous
CADM2		4.60E-08	-0.24	Adipose - Visceral (Omentum)
CADM2		1.30E-06	-0.38	Heart - Left Ventricle
CADM2	rs6788098	1.80E-18	-0.41	Lung
CADM2		8.00E-05	0.15	Adipose - Subcutaneous
CADM2	rs6788656	4.40E-05	0.14	Muscle - Skeletal
CADM2		6.80E-12	-0.29	Adipose - Subcutaneous
CADM2		1.50E-08	-0.25	Adipose - Visceral (Omentum)
CADM2		7.40E-07	-0.39	Heart - Left Ventricle
CADM2		1.80E-18	-0.41	Lung
CADM2	rs6790090	1.60E-04	0.099	Nerve - Tibial
CADM2-AS1		2.70E-05	0.56	Brain - Caudate (basal ganglia)
CADM2-AS1		2.30E-07	0.64	Brain - Cerebellar Hemisphere
CADM2-AS1		1.40E-07	0.51	Brain - Cerebellum
CADM2-AS1		6.00E-06	0.51	Brain - Cortex
CADM2-AS1		1.70E-06	0.6	Brain - Frontal Cortex (BA9)
CADM2-AS1	rs6790226	1.20E-06	0.57	Brain - Nucleus accumbens (basal ganglia)
CADM2		2.20E-11	-0.28	Adipose - Subcutaneous
CADM2		4.50E-08	-0.24	Adipose - Visceral (Omentum)
CADM2		1.40E-06	-0.38	Heart - Left Ventricle
CADM2	rs67904150	3.20E-18	-0.4	Lung
CADM2		2.10E-11	-0.28	Adipose - Subcutaneous
CADM2		4.60E-08	-0.24	Adipose - Visceral (Omentum)
CADM2		1.30E-06	-0.38	Heart - Left Ventricle
CADM2	rs6790699	1.80E-18	-0.41	Lung
CADM2		1.90E-05	0.16	Adipose - Subcutaneous
CADM2	rs6790864	1.30E-04	0.13	Muscle - Skeletal
CADM2		8.90E-05	0.15	Adipose - Subcutaneous
CADM2	rs6791035	1.50E-05	0.15	Muscle - Skeletal
CADM2		2.10E-07	0.27	Adipose - Subcutaneous
CADM2		2.00E-05	0.24	Adipose - Visceral (Omentum)
CADM2	rs6791236	1.00E-08	0.34	Lung
CADM2		7.90E-05	0.16	Adipose - Subcutaneous
CADM2		1.70E-05	0.26	Brain - Caudate (basal ganglia)
CADM2		1.20E-05	0.6	Brain - Spinal cord (cervical c-1)
CADM2	rs6791363	1.20E-05	0.16	Muscle - Skeletal
CADM2		6.80E-12	-0.29	Adipose - Subcutaneous
CADM2		1.50E-08	-0.25	Adipose - Visceral (Omentum)
CADM2		7.40E-07	-0.39	Heart - Left Ventricle
CADM2		1.80E-18	-0.41	Lung
CADM2	rs6792295	1.60E-04	0.099	Nerve - Tibial
CADM2-AS1		6.10E-05	-0.46	Brain - Cerebellar Hemisphere
CADM2-AS1	rs6792588	2.90E-05	-0.49	Brain - Nucleus accumbens (basal ganglia)
CADM2-AS1	rs6794094	7.90E-05	-0.37	Nerve - Tibial
CADM2		2.20E-11	-0.28	Adipose - Subcutaneous
CADM2		7.20E-08	-0.24	Adipose - Visceral (Omentum)
CADM2		1.20E-06	-0.38	Heart - Left Ventricle
CADM2	rs6794866	6.40E-18	-0.4	Lung
CADM2		8.70E-11	-0.28	Adipose - Subcutaneous
CADM2		3.10E-07	-0.24	Adipose - Visceral (Omentum)
CADM2	rs67955391	1.30E-06	-0.38	Heart - Left Ventricle

CADM2		1.30E-18	-0.42	Lung
CADM2-AS1		2.80E-06	0.54	Brain - Cerebellar Hemisphere
CADM2-AS1	rs6795667	1.30E-08	0.51	Brain - Cerebellum
CADM2	rs6796226	6.30E-08	-0.35	Lung
CADM2		9.50E-05	0.15	Adipose - Subcutaneous
CADM2	rs6796256	8.90E-05	0.14	Muscle - Skeletal
CADM2-AS1		8.20E-06	0.58	Brain - Caudate (basal ganglia)
CADM2-AS1		1.20E-08	0.66	Brain - Cerebellar Hemisphere
CADM2-AS1		7.70E-08	0.5	Brain - Cerebellum
CADM2-AS1		3.70E-06	0.52	Brain - Cortex
CADM2-AS1		1.50E-06	0.59	Brain - Frontal Cortex (BA9)
CADM2-AS1	rs6796380	4.30E-07	0.59	Brain - Nucleus accumbens (basal ganglia)
CADM2		1.40E-05	0.17	Adipose - Subcutaneous
CADM2	rs6796393	1.20E-04	0.13	Muscle - Skeletal
CADM2-AS1		5.00E-06	0.58	Brain - Caudate (basal ganglia)
CADM2-AS1		5.60E-08	0.64	Brain - Cerebellar Hemisphere
CADM2-AS1		1.40E-07	0.49	Brain - Cerebellum
CADM2-AS1		4.90E-06	0.51	Brain - Cortex
CADM2-AS1		1.40E-06	0.59	Brain - Frontal Cortex (BA9)
CADM2-AS1	rs6796402	3.20E-06	0.53	Brain - Nucleus accumbens (basal ganglia)
CADM2-AS1	rs6797109	2.50E-05	0.51	Brain - Cerebellar Hemisphere
CADM2		3.80E-06	-0.49	Heart - Left Ventricle
CADM2	rs6797581	2.20E-05	-0.28	Lung
CADM2-AS1	rs6797840	3.40E-06	-0.28	Nerve - Tibial
CADM2		4.80E-05	0.16	Adipose - Subcutaneous
CADM2	rs6798922	7.20E-05	0.14	Muscle - Skeletal
CADM2-AS1		1.30E-05	0.55	Brain - Caudate (basal ganglia)
CADM2-AS1		6.30E-07	0.59	Brain - Cerebellar Hemisphere
CADM2-AS1		5.90E-08	0.5	Brain - Cerebellum
CADM2-AS1		5.30E-06	0.49	Brain - Cortex
CADM2-AS1		6.30E-06	0.54	Brain - Frontal Cortex (BA9)
CADM2-AS1	rs6798987	7.20E-06	0.51	Brain - Nucleus accumbens (basal ganglia)
CADM2		6.90E-05	-0.17	Adipose - Subcutaneous
CADM2	rs6799195	5.20E-12	-0.33	Lung
CADM2-AS1		2.40E-06	0.54	Brain - Cerebellar Hemisphere
CADM2-AS1		1.10E-08	0.51	Brain - Cerebellum
CADM2-AS1	rs6799453	2.70E-05	0.5	Brain - Nucleus accumbens (basal ganglia)
CADM2		1.40E-11	-0.28	Adipose - Subcutaneous
CADM2		2.50E-07	-0.24	Adipose - Visceral (Omentum)
CADM2		9.00E-07	-0.39	Heart - Left Ventricle
CADM2		4.40E-17	-0.4	Lung
CADM2	rs67999244	1.10E-04	0.1	Nerve - Tibial
CADM2		2.30E-11	-0.28	Adipose - Subcutaneous
CADM2		8.80E-08	-0.24	Adipose - Visceral (Omentum)
CADM2		1.30E-06	-0.38	Heart - Left Ventricle
CADM2	rs68001049	7.00E-18	-0.4	Lung
CADM2		1.10E-04	0.15	Adipose - Subcutaneous
CADM2	rs6801271	2.50E-07	0.18	Muscle - Skeletal
CADM2	rs6801823	6.80E-12	-0.29	Adipose - Subcutaneous

CADM2		1.50E-08	-0.25	Adipose - Visceral (Omentum)
CADM2		7.40E-07	-0.39	Heart - Left Ventricle
CADM2		1.80E-18	-0.41	Lung
CADM2		1.60E-04	0.099	Nerve - Tibial
CADM2	rs6802287	2.10E-05	0.52	Heart - Left Ventricle
CADM2	rs6802399	7.00E-08	-0.35	Lung
CADM2		3.90E-11	-0.28	Adipose - Subcutaneous
CADM2		2.50E-07	-0.24	Adipose - Visceral (Omentum)
CADM2		8.20E-07	-0.39	Heart - Left Ventricle
CADM2		4.20E-17	-0.4	Lung
CADM2	rs68028504	1.30E-04	0.1	Nerve - Tibial
CADM2-AS1		4.90E-06	-0.62	Brain - Caudate (basal ganglia)
CADM2-AS1		7.20E-06	-0.58	Brain - Cerebellar Hemisphere
CADM2-AS1		7.20E-07	-0.5	Brain - Cerebellum
CADM2-AS1		3.40E-06	-0.63	Brain - Frontal Cortex (BA9)
CADM2-AS1	rs68033001	1.00E-05	-0.55	Brain - Nucleus accumbens (basal ganglia)
CADM2		7.20E-06	0.2	Adipose - Subcutaneous
CADM2		1.10E-08	0.71	Brain - Spinal cord (cervical c-1)
CADM2	rs6803322	1.50E-06	0.19	Muscle - Skeletal
CADM2		1.00E-11	-0.28	Adipose - Subcutaneous
CADM2		1.60E-08	-0.25	Adipose - Visceral (Omentum)
CADM2		7.40E-07	-0.39	Heart - Left Ventricle
CADM2	rs6804626	5.20E-18	-0.4	Lung
CADM2		5.00E-12	-0.29	Adipose - Subcutaneous
CADM2		1.60E-08	-0.25	Adipose - Visceral (Omentum)
CADM2		6.00E-07	-0.39	Heart - Left Ventricle
CADM2	rs6804845	4.20E-18	-0.4	Lung
CADM2		4.00E-12	-0.29	Adipose - Subcutaneous
CADM2		9.20E-09	-0.26	Adipose - Visceral (Omentum)
CADM2		7.40E-07	-0.39	Heart - Left Ventricle
CADM2		2.70E-18	-0.41	Lung
CADM2	rs68049270	1.30E-04	0.1	Nerve - Tibial
CADM2-AS1		2.10E-06	0.6	Brain - Caudate (basal ganglia)
CADM2-AS1		8.40E-09	0.7	Brain - Cerebellar Hemisphere
CADM2-AS1		3.00E-11	0.64	Brain - Cerebellum
CADM2-AS1		3.90E-06	0.55	Brain - Cortex
CADM2-AS1		1.20E-06	0.64	Brain - Frontal Cortex (BA9)
CADM2-AS1		1.90E-06	0.68	Brain - Hippocampus
CADM2-AS1	rs68058340	9.60E-09	0.7	Brain - Nucleus accumbens (basal ganglia)
CADM2	rs6807445	4.30E-07	-0.33	Lung
CADM2		6.10E-09	0.29	Adipose - Subcutaneous
CADM2		4.50E-05	0.23	Adipose - Visceral (Omentum)
CADM2	rs6807456	7.20E-06	0.26	Lung
CADM2	rs6807461	2.10E-07	-0.34	Lung
CADM2		1.00E-09	-0.25	Adipose - Subcutaneous
CADM2		1.00E-07	-0.24	Adipose - Visceral (Omentum)
CADM2		2.00E-07	-0.39	Heart - Left Ventricle
CADM2	rs6807666	8.80E-20	-0.41	Lung
CADM2		3.30E-08	0.26	Adipose - Subcutaneous
CADM2		3.20E-05	0.22	Adipose - Visceral (Omentum)
CADM2	rs6808159	1.30E-06	0.27	Lung

CADM2		5.80E-11	-0.27	Adipose - Subcutaneous
CADM2		9.10E-08	-0.24	Adipose - Visceral (Omentum)
CADM2		2.20E-07	-0.4	Heart - Left Ventricle
CADM2		2.50E-14	-0.35	Lung
CADM2	rs6808400	8.70E-05	-0.15	Muscle - Skeletal
CADM2		9.70E-06	0.32	Brain - Hippocampus
CADM2	rs6808586	1.70E-04	0.13	Muscle - Skeletal
CADM2-AS1		3.80E-05	0.5	Brain - Cerebellar Hemisphere
CADM2-AS1	rs6809333	3.30E-05	0.48	Brain - Nucleus accumbens (basal ganglia)
CADM2		5.90E-08	0.28	Adipose - Subcutaneous
CADM2		6.80E-06	0.25	Adipose - Visceral (Omentum)
CADM2	rs6809805	6.00E-09	0.34	Lung
CADM2		2.50E-06	-0.2	Adipose - Subcutaneous
CADM2		8.00E-10	-0.28	Lung
CADM2-AS1	rs68102233	8.20E-05	0.26	Nerve - Tibial
CADM2		9.80E-05	-0.42	Heart - Left Ventricle
CADM2	rs6810225	2.90E-07	-0.34	Lung
CADM2		1.70E-05	0.19	Adipose - Subcutaneous
CADM2		4.20E-07	0.65	Brain - Spinal cord (cervical c-1)
CADM2	rs6810250	1.20E-04	0.15	Muscle - Skeletal
CADM2	rs694795	7.30E-05	-0.14	Muscle - Skeletal
CADM2		1.30E-07	0.28	Adipose - Subcutaneous
CADM2		6.40E-05	0.23	Adipose - Visceral (Omentum)
CADM2	rs71108282	8.60E-10	0.38	Lung
CADM2-AS1		1.60E-06	0.53	Brain - Cerebellar Hemisphere
CADM2-AS1		4.00E-05	0.42	Brain - Cerebellum
CADM2-AS1	rs71108301	6.70E-06	0.52	Brain - Nucleus accumbens (basal ganglia)
CADM2-AS1	rs71128120	3.80E-05	-0.39	Nerve - Tibial
CADM2	rs71316810	1.40E-04	0.31	Adipose - Subcutaneous
CADM2-AS1		2.30E-07	0.62	Brain - Caudate (basal ganglia)
CADM2-AS1		3.10E-09	0.69	Brain - Cerebellar Hemisphere
CADM2-AS1		1.70E-09	0.58	Brain - Cerebellum
CADM2-AS1	rs71316818	2.20E-05	0.51	Brain - Nucleus accumbens (basal ganglia)
CADM2-AS1	rs71318645	1.30E-05	-0.41	Nerve - Tibial
CADM2		1.60E-08	0.27	Adipose - Subcutaneous
CADM2		2.10E-05	0.22	Adipose - Visceral (Omentum)
CADM2	rs71626887	1.60E-06	0.27	Lung
CADM2	rs71626890	1.80E-05	0.27	Lung
CADM2		2.60E-12	-0.29	Adipose - Subcutaneous
CADM2		2.10E-08	-0.25	Adipose - Visceral (Omentum)
CADM2		8.20E-07	-0.39	Heart - Left Ventricle
CADM2	rs724304	3.00E-17	-0.4	Lung
CADM2		3.40E-05	-0.18	Adipose - Subcutaneous
CADM2		9.00E-05	-0.19	Adipose - Visceral (Omentum)
CADM2	rs72615721	4.00E-07	-0.25	Lung
CADM2		8.20E-07	-0.21	Adipose - Subcutaneous
CADM2		2.10E-09	-0.27	Lung
CADM2-AS1	rs72615725	1.10E-04	0.26	Nerve - Tibial
CADM2		2.10E-12	-0.3	Adipose - Subcutaneous
CADM2		5.30E-09	-0.26	Adipose - Visceral (Omentum)
CADM2	rs72615726	7.60E-07	-0.39	Heart - Left Ventricle

CADM2		5.60E-18	-0.41	Lung
CADM2		1.70E-04	0.1	Nerve - Tibial
CADM2		6.80E-12	-0.29	Adipose - Subcutaneous
CADM2		1.50E-08	-0.25	Adipose - Visceral (Omentum)
CADM2		7.40E-07	-0.39	Heart - Left Ventricle
CADM2		1.80E-18	-0.41	Lung
CADM2	rs72615727	1.60E-04	0.099	Nerve - Tibial
CADM2-AS1		2.40E-06	0.54	Brain - Cerebellar Hemisphere
CADM2-AS1		1.10E-08	0.51	Brain - Cerebellum
CADM2-AS1	rs726453	2.70E-05	0.5	Brain - Nucleus accumbens (basal ganglia)
CADM2		9.10E-09	-0.24	Adipose - Subcutaneous
CADM2		2.00E-08	-0.26	Adipose - Visceral (Omentum)
CADM2		5.70E-07	-0.38	Heart - Left Ventricle
CADM2	rs726610	1.30E-19	-0.41	Lung
CADM2	rs72905270	1.20E-04	-0.94	Testis
CADM2		6.80E-12	-0.29	Adipose - Subcutaneous
CADM2		3.30E-08	-0.25	Adipose - Visceral (Omentum)
CADM2		1.30E-06	-0.38	Heart - Left Ventricle
CADM2	rs73130364	2.50E-18	-0.41	Lung
CADM2		6.80E-12	-0.29	Adipose - Subcutaneous
CADM2		3.30E-08	-0.25	Adipose - Visceral (Omentum)
CADM2		1.30E-06	-0.38	Heart - Left Ventricle
CADM2	rs73130366	2.50E-18	-0.41	Lung
CADM2	rs73132094	8.20E-05	-0.19	Muscle - Skeletal
CADM2	rs73132101	4.30E-05	-0.2	Muscle - Skeletal
CADM2-AS1		2.40E-07	-0.66	Brain - Cerebellar Hemisphere
CADM2-AS1		1.20E-07	-0.53	Brain - Cerebellum
CADM2-AS1		3.20E-06	-0.59	Brain - Frontal Cortex (BA9)
CADM2-AS1	rs73132654	2.60E-06	-0.57	Brain - Nucleus accumbens (basal ganglia)
CADM2	rs73133670	1.80E-05	-0.19	Muscle - Skeletal
CADM2		5.30E-11	-0.27	Adipose - Subcutaneous
CADM2		1.50E-08	-0.25	Adipose - Visceral (Omentum)
CADM2		6.50E-07	-0.39	Heart - Left Ventricle
CADM2	rs73133799	3.80E-18	-0.4	Lung
CADM2		3.10E-11	-0.28	Adipose - Subcutaneous
CADM2		1.40E-08	-0.25	Adipose - Visceral (Omentum)
CADM2		6.80E-07	-0.39	Heart - Left Ventricle
CADM2	rs73133800	3.80E-18	-0.4	Lung
CADM2	rs73135623	3.80E-05	-0.18	Muscle - Skeletal
CADM2	rs73135629	4.40E-05	-0.18	Muscle - Skeletal
CADM2	rs73135634	1.10E-05	-0.19	Muscle - Skeletal
CADM2	rs73137638	1.90E-06	1.2	Spleen
CADM2	rs73137655	2.40E-06	1.2	Spleen
CADM2	rs73137667	2.20E-05	1.1	Spleen
CADM2	rs73137668	2.10E-06	1.2	Spleen
CADM2	rs73137671	1.90E-06	1.2	Spleen
CADM2	rs73137672	2.10E-06	1.2	Spleen
CADM2	rs73137679	1.80E-06	1.2	Spleen
CADM2	rs73137895	1.00E-06	-0.21	Adipose - Subcutaneous

CADM2		1.80E-09	-0.27	Lung
CADM2-AS1		6.80E-07	0.61	Brain - Cerebellar Hemisphere
CADM2-AS1		5.10E-09	0.6	Brain - Cerebellum
CADM2-AS1		3.60E-07	0.64	Brain - Nucleus accumbens (basal ganglia)
CADM2-AS1		8.60E-06	0.59	Brain - Putamen (basal ganglia)
CADM2-AS1	rs73138150	1.40E-05	0.31	Nerve - Tibial
CADM2	rs73139653	1.50E-06	1.2	Spleen
CADM2	rs73139656	4.00E-06	1.3	Spleen
CADM2	rs73139658	1.50E-06	1.2	Spleen
CADM2	rs73139660	1.90E-05	1.1	Spleen
CADM2	rs73139669	1.90E-06	1.2	Spleen
CADM2	rs73139677	2.30E-06	1.2	Spleen
CADM2	rs73139679	9.70E-08	1.2	Spleen
CADM2		7.80E-06	-0.21	Lung
CADM2-AS1	rs73141547	1.30E-04	0.25	Nerve - Tibial
CADM2-AS1	rs73142849	4.20E-05	-0.47	Brain - Cerebellar Hemisphere
CADM2-AS1		4.50E-05	-0.46	Brain - Cerebellar Hemisphere
CADM2-AS1	rs73142850	2.30E-05	-0.5	Brain - Nucleus accumbens (basal ganglia)
CADM2-AS1	rs73142855	4.20E-05	-0.47	Brain - Cerebellar Hemisphere
CADM2-AS1	rs73147239	1.40E-05	-0.5	Brain - Nucleus accumbens (basal ganglia)
CADM2-AS1	rs73147242	4.20E-05	-0.47	Brain - Cerebellar Hemisphere
CADM2-AS1	rs73147253	4.50E-05	-0.47	Brain - Cerebellar Hemisphere
CADM2	rs734298	6.30E-08	-0.35	Lung
CADM2-AS1	rs7355787	6.40E-05	-0.28	Nerve - Tibial
CADM2		6.00E-05	0.18	Adipose - Subcutaneous
CADM2	rs7355926	7.00E-07	0.62	Brain - Spinal cord (cervical c-1)
CADM2		2.30E-06	0.6	Brain - Spinal cord (cervical c-1)
CADM2	rs7355930	8.30E-05	0.17	Muscle - Skeletal
CADM2	rs7355953	7.30E-06	0.27	Lung
CADM2-AS1		6.80E-06	0.58	Brain - Caudate (basal ganglia)
CADM2-AS1		1.20E-08	0.66	Brain - Cerebellar Hemisphere
CADM2-AS1		7.60E-08	0.5	Brain - Cerebellum
CADM2-AS1		4.70E-06	0.51	Brain - Cortex
CADM2-AS1		1.80E-06	0.58	Brain - Frontal Cortex (BA9)
CADM2-AS1	rs7426412	4.30E-07	0.59	Brain - Nucleus accumbens (basal ganglia)
CADM2		4.40E-12	-0.29	Adipose - Subcutaneous
CADM2		1.40E-08	-0.25	Adipose - Visceral (Omentum)
CADM2		6.10E-07	-0.39	Heart - Left Ventricle
CADM2		3.50E-18	-0.4	Lung
CADM2-AS1		2.70E-05	-0.41	Nerve - Tibial
CADM2-AS1		6.40E-06	0.52	Brain - Cerebellar Hemisphere
CADM2-AS1	rs7427346	1.80E-07	0.49	Brain - Cerebellum
CADM2-AS1		2.70E-07	0.59	Brain - Caudate (basal ganglia)
CADM2-AS1		4.60E-05	0.47	Brain - Cerebellar Hemisphere
CADM2-AS1	rs7431895	8.10E-08	0.5	Brain - Cerebellum
CADM2-AS1		2.90E-05	0.56	Brain - Cerebellar Hemisphere
CADM2-AS1	rs7432003	1.80E-06	0.49	Brain - Cerebellum
CADM2	rs74345243	2.10E-05	0.52	Heart - Left Ventricle

CADM2		5.20E-12	-0.29	Adipose - Subcutaneous
CADM2		2.90E-08	-0.25	Adipose - Visceral (Omentum)
CADM2		6.90E-07	-0.39	Heart - Left Ventricle
CADM2		1.70E-18	-0.41	Lung
CADM2	rs74384786	1.60E-04	0.099	Nerve - Tibial
CADM2		4.60E-11	-0.28	Adipose - Subcutaneous
CADM2		2.20E-07	-0.24	Adipose - Visceral (Omentum)
CADM2		1.50E-06	-0.38	Heart - Left Ventricle
CADM2		1.90E-18	-0.41	Lung
CADM2	rs74420518	1.30E-04	0.1	Nerve - Tibial
CADM2	rs74439542	5.90E-05	0.5	Heart - Left Ventricle
CADM2		4.50E-08	0.26	Adipose - Subcutaneous
CADM2		4.00E-05	0.21	Adipose - Visceral (Omentum)
CADM2	rs744580	2.20E-06	0.26	Lung
CADM2	rs74462524	1.70E-05	-0.65	Brain - Hippocampus
CADM2	rs74476348	4.30E-05	0.19	Brain - Cerebellum
CADM2-AS1		1.60E-06	0.56	Brain - Caudate (basal ganglia)
CADM2-AS1		4.50E-05	0.46	Brain - Cerebellar Hemisphere
CADM2-AS1		1.60E-07	0.48	Brain - Cerebellum
CADM2-AS1	rs74955449	3.40E-06	0.52	Brain - Nucleus accumbens (basal ganglia)
CADM2-AS1		3.40E-05	0.54	Brain - Cerebellar Hemisphere
CADM2-AS1		2.10E-06	0.47	Brain - Cerebellum
CADM2-AS1	rs75065681	4.70E-05	0.47	Brain - Cortex
CADM2	rs75082056	5.30E-05	0.5	Heart - Left Ventricle
CADM2	rs75135056	2.60E-08	1	Heart - Left Ventricle
CADM2	rs75176793	9.20E-06	-0.66	Brain - Hippocampus
CADM2	rs75442558	5.90E-05	0.5	Heart - Left Ventricle
CADM2	rs75501065	1.50E-06	0.83	Heart - Left Ventricle
CADM2	rs75520167	9.20E-06	-0.66	Brain - Hippocampus
CADM2	rs75744227	8.20E-06	-0.66	Brain - Hippocampus
CADM2		3.00E-12	-0.29	Adipose - Subcutaneous
CADM2		1.90E-08	-0.25	Adipose - Visceral (Omentum)
CADM2		6.90E-07	-0.39	Heart - Left Ventricle
CADM2		1.50E-18	-0.41	Lung
CADM2	rs76034006	1.50E-04	0.1	Nerve - Tibial
CADM2		8.90E-11	-0.27	Adipose - Subcutaneous
CADM2		3.60E-08	-0.24	Adipose - Visceral (Omentum)
CADM2		5.00E-08	-0.42	Heart - Left Ventricle
CADM2		1.40E-14	-0.36	Lung
CADM2	rs7609594	6.70E-05	-0.15	Muscle - Skeletal
CADM2-AS1		4.50E-05	0.52	Brain - Caudate (basal ganglia)
CADM2-AS1		3.10E-07	0.62	Brain - Cerebellar Hemisphere
CADM2-AS1		1.30E-07	0.49	Brain - Cerebellum
CADM2-AS1		2.50E-05	0.47	Brain - Cortex
CADM2-AS1		1.10E-06	0.61	Brain - Frontal Cortex (BA9)
CADM2-AS1	rs7609709	7.20E-06	0.53	Brain - Nucleus accumbens (basal ganglia)
CADM2-AS1	rs7610076	6.50E-05	-0.28	Nerve - Tibial
CADM2	rs7610238	1.80E-06	-0.51	Heart - Left Ventricle
CADM2	rs7610238	4.70E-06	-0.3	Lung
CADM2		6.80E-12	-0.29	Adipose - Subcutaneous
CADM2	rs76105429	1.30E-08	-0.25	Adipose - Visceral (Omentum)

CADM2		6.80E-07	-0.39	Heart - Left Ventricle
CADM2		1.90E-18	-0.41	Lung
CADM2		1.70E-04	0.099	Nerve - Tibial
CADM2-AS1		3.70E-06	0.53	Brain - Cerebellar Hemisphere
CADM2-AS1		7.20E-08	0.51	Brain - Cerebellum
CADM2-AS1	rs7611409	2.60E-05	0.5	Brain - Nucleus accumbens (basal ganglia)
CADM2	rs7612213	5.80E-06	0.28	Lung
CADM2-AS1		3.90E-06	0.53	Brain - Cerebellar Hemisphere
CADM2-AS1		7.50E-08	0.5	Brain - Cerebellum
CADM2-AS1		3.10E-05	0.5	Brain - Frontal Cortex (BA9)
CADM2-AS1	rs7612371	1.60E-05	0.51	Brain - Nucleus accumbens (basal ganglia)
CADM2-AS1		3.40E-05	0.54	Brain - Cerebellar Hemisphere
CADM2-AS1		2.10E-06	0.47	Brain - Cerebellum
CADM2-AS1	rs7612458	4.70E-05	0.47	Brain - Cortex
CADM2-AS1		2.20E-06	0.55	Brain - Caudate (basal ganglia)
CADM2-AS1		1.60E-05	0.49	Brain - Cerebellar Hemisphere
CADM2-AS1		3.20E-08	0.51	Brain - Cerebellum
CADM2-AS1	rs7613695	1.10E-04	0.25	Nerve - Tibial
CADM2-AS1		1.20E-05	0.57	Brain - Caudate (basal ganglia)
CADM2-AS1		5.20E-08	0.65	Brain - Cerebellar Hemisphere
CADM2-AS1		1.00E-06	0.47	Brain - Cerebellum
CADM2-AS1		1.00E-05	0.57	Brain - Frontal Cortex (BA9)
CADM2-AS1	rs7614073	8.40E-06	0.54	Brain - Nucleus accumbens (basal ganglia)
CADM2		5.90E-12	-0.29	Adipose - Subcutaneous
CADM2		8.70E-08	-0.24	Adipose - Visceral (Omentum)
CADM2		4.80E-07	-0.4	Heart - Left Ventricle
CADM2		9.60E-17	-0.39	Lung
CADM2	rs7614148	1.50E-04	0.1	Nerve - Tibial
CADM2		2.30E-08	-0.23	Adipose - Subcutaneous
CADM2		1.50E-07	-0.23	Adipose - Visceral (Omentum)
CADM2		5.30E-08	-0.41	Heart - Left Ventricle
CADM2	rs7614552	3.20E-19	-0.41	Lung
CADM2		4.70E-12	-0.29	Adipose - Subcutaneous
CADM2		1.50E-08	-0.25	Adipose - Visceral (Omentum)
CADM2		7.50E-07	-0.39	Heart - Left Ventricle
CADM2		3.00E-18	-0.41	Lung
CADM2	rs7616458	1.30E-04	0.1	Nerve - Tibial
CADM2		1.30E-12	-0.3	Adipose - Subcutaneous
CADM2		1.60E-08	-0.26	Adipose - Visceral (Omentum)
CADM2		9.10E-07	-0.39	Heart - Left Ventricle
CADM2		3.60E-17	-0.4	Lung
CADM2	rs7617306	9.60E-05	0.1	Nerve - Tibial
CADM2		6.50E-11	-0.27	Adipose - Subcutaneous
CADM2		2.40E-07	-0.23	Adipose - Visceral (Omentum)
CADM2		1.20E-06	-0.38	Heart - Left Ventricle
CADM2	rs7617323	1.80E-18	-0.41	Lung
CADM2		2.10E-11	-0.28	Adipose - Subcutaneous
CADM2		4.30E-08	-0.24	Adipose - Visceral (Omentum)
CADM2	rs7617356	1.50E-06	-0.38	Heart - Left Ventricle

CADM2		2.90E-18	-0.41	Lung
CADM2		6.00E-05	0.18	Adipose - Subcutaneous
CADM2	rs7617565	7.00E-07	0.62	Brain - Spinal cord (cervical c-1)
CADM2		1.20E-08	-0.26	Adipose - Subcutaneous
CADM2		4.60E-07	-0.27	Adipose - Visceral (Omentum)
CADM2		8.40E-08	-0.45	Heart - Left Ventricle
CADM2	rs7618124	3.90E-14	-0.4	Lung
CADM2		7.00E-12	-0.29	Adipose - Subcutaneous
CADM2		1.90E-08	-0.25	Adipose - Visceral (Omentum)
CADM2		6.00E-07	-0.39	Heart - Left Ventricle
CADM2	rs7618429	2.10E-17	-0.4	Lung
CADM2	rs76184669	7.90E-05	0.54	Heart - Atrial Appendage
CADM2		1.80E-09	-0.25	Adipose - Subcutaneous
CADM2		1.00E-07	-0.24	Adipose - Visceral (Omentum)
CADM2		1.30E-07	-0.4	Heart - Left Ventricle
CADM2	rs7618494	2.40E-19	-0.41	Lung
CADM2		1.00E-11	-0.29	Adipose - Subcutaneous
CADM2		6.40E-09	-0.26	Adipose - Visceral (Omentum)
CADM2		7.40E-07	-0.39	Heart - Left Ventricle
CADM2		1.80E-18	-0.41	Lung
CADM2	rs7620313	1.60E-04	0.099	Nerve - Tibial
CADM2	rs7620339	8.30E-05	0.21	Adipose - Subcutaneous
CADM2-AS1		4.10E-06	0.6	Brain - Caudate (basal ganglia)
CADM2-AS1		5.10E-08	0.65	Brain - Cerebellar Hemisphere
CADM2-AS1		1.40E-07	0.5	Brain - Cerebellum
CADM2-AS1		6.30E-06	0.5	Brain - Cortex
CADM2-AS1		1.90E-06	0.59	Brain - Frontal Cortex (BA9)
CADM2-AS1	rs7620544	1.30E-06	0.57	Brain - Nucleus accumbens (basal ganglia)
CADM2-AS1		6.60E-07	0.56	Brain - Caudate (basal ganglia)
CADM2-AS1		3.80E-07	0.58	Brain - Cerebellar Hemisphere
CADM2-AS1	rs7620825	8.40E-09	0.52	Brain - Cerebellum
CADM2-AS1		1.70E-05	0.55	Brain - Caudate (basal ganglia)
CADM2-AS1		6.50E-07	0.59	Brain - Cerebellar Hemisphere
CADM2-AS1		1.20E-07	0.5	Brain - Cerebellum
CADM2-AS1		2.50E-05	0.52	Brain - Frontal Cortex (BA9)
CADM2-AS1	rs7621146	1.30E-05	0.51	Brain - Nucleus accumbens (basal ganglia)
CADM2		3.80E-12	-0.29	Adipose - Subcutaneous
CADM2		8.20E-09	-0.26	Adipose - Visceral (Omentum)
CADM2		9.90E-07	-0.38	Heart - Left Ventricle
CADM2	rs7621381	9.50E-18	-0.4	Lung
CADM2	rs76214943	2.80E-08	1	Heart - Left Ventricle
CADM2-AS1	rs7622475	1.40E-04	-0.31	Nerve - Tibial
CADM2-AS1	rs7623220	1.00E-05	-0.41	Nerve - Tibial
CADM2		6.90E-05	-0.43	Heart - Left Ventricle
CADM2	rs7623369	2.30E-07	-0.34	Lung
CADM2	rs7623474	6.30E-08	-0.35	Lung
CADM2	rs7623480	9.10E-06	-0.21	Lung
CADM2	rs7623714	8.30E-06	0.16	Muscle - Skeletal
CADM2-AS1		4.30E-05	0.53	Brain - Caudate (basal ganglia)
CADM2-AS1	rs7623735	4.80E-08	0.66	Brain - Cerebellar Hemisphere

CADM2-AS1		5.00E-08	0.51	Brain - Cerebellum
CADM2-AS1		1.10E-05	0.49	Brain - Cortex
CADM2-AS1		5.20E-07	0.62	Brain - Frontal Cortex (BA9)
CADM2-AS1		3.70E-06	0.54	Brain - Nucleus accumbens (basal ganglia)
CADM2	rs7623947	6.30E-08	-0.35	Lung
CADM2		4.70E-06	-0.2	Adipose - Subcutaneous
CADM2	rs7624108	6.10E-10	-0.28	Lung
CADM2	rs76243389	9.20E-06	-0.66	Brain - Hippocampus
CADM2-AS1		1.60E-05	0.55	Brain - Caudate (basal ganglia)
CADM2-AS1		1.60E-07	0.62	Brain - Cerebellar Hemisphere
CADM2-AS1		7.30E-08	0.51	Brain - Cerebellum
CADM2-AS1		8.70E-07	0.6	Brain - Frontal Cortex (BA9)
CADM2-AS1	rs7625223	4.20E-06	0.53	Brain - Nucleus accumbens (basal ganglia)
CADM2	rs7625311	4.00E-06	-0.52	Brain - Spinal cord (cervical c-1)
CADM2-AS1		1.40E-05	0.55	Brain - Caudate (basal ganglia)
CADM2-AS1		4.00E-07	0.6	Brain - Cerebellar Hemisphere
CADM2-AS1		5.30E-08	0.5	Brain - Cerebellum
CADM2-AS1		5.40E-06	0.56	Brain - Frontal Cortex (BA9)
CADM2-AS1	rs7625546	2.30E-05	0.49	Brain - Nucleus accumbens (basal ganglia)
CADM2-AS1		3.70E-05	0.55	Brain - Caudate (basal ganglia)
CADM2-AS1		1.60E-07	0.65	Brain - Cerebellar Hemisphere
CADM2-AS1		7.20E-08	0.52	Brain - Cerebellum
CADM2-AS1		6.00E-06	0.51	Brain - Cortex
CADM2-AS1		1.10E-06	0.61	Brain - Frontal Cortex (BA9)
CADM2-AS1	rs7626485	1.30E-06	0.57	Brain - Nucleus accumbens (basal ganglia)
CADM2		2.30E-06	-0.2	Adipose - Subcutaneous
CADM2		7.20E-09	-0.27	Lung
CADM2-AS1	rs7626594	5.40E-05	0.27	Nerve - Tibial
CADM2-AS1		3.20E-05	0.53	Brain - Caudate (basal ganglia)
CADM2-AS1		1.10E-07	0.63	Brain - Cerebellar Hemisphere
CADM2-AS1		6.00E-08	0.5	Brain - Cerebellum
CADM2-AS1		2.30E-05	0.47	Brain - Cortex
CADM2-AS1		8.70E-07	0.6	Brain - Frontal Cortex (BA9)
CADM2-AS1	rs7626873	3.80E-06	0.54	Brain - Nucleus accumbens (basal ganglia)
CADM2		9.20E-11	-0.27	Adipose - Subcutaneous
CADM2		6.30E-08	-0.25	Adipose - Visceral (Omentum)
CADM2		7.40E-07	-0.4	Heart - Left Ventricle
CADM2	rs7627044	5.00E-17	-0.4	Lung
CADM2-AS1		2.60E-07	0.57	Brain - Caudate (basal ganglia)
CADM2-AS1		3.30E-07	0.57	Brain - Cerebellar Hemisphere
CADM2-AS1	rs7627287	9.00E-10	0.54	Brain - Cerebellum
CADM2		4.80E-05	0.17	Adipose - Subcutaneous
CADM2		8.40E-05	0.31	Heart - Left Ventricle
CADM2	rs7627780	3.40E-11	0.32	Lung
CADM2		1.80E-06	-0.23	Adipose - Subcutaneous
CADM2		1.70E-05	-0.23	Adipose - Visceral (Omentum)
CADM2		1.20E-05	-0.23	Breast - Mammary Tissue
CADM2	rs7627971	1.80E-09	-0.33	Lung
CADM2	rs7628129	1.20E-05	0.32	Brain - Hippocampus

CADM2		9.60E-05	0.14	Muscle - Skeletal
CADM2		3.00E-12	-0.3	Adipose - Subcutaneous
CADM2		2.00E-08	-0.25	Adipose - Visceral (Omentum)
CADM2		8.30E-07	-0.39	Heart - Left Ventricle
CADM2	rs7628237	3.00E-17	-0.4	Lung
CADM2		5.00E-08	0.28	Adipose - Subcutaneous
CADM2		6.80E-06	0.25	Adipose - Visceral (Omentum)
CADM2	rs7628948	5.40E-09	0.34	Lung
CADM2		7.50E-12	-0.29	Adipose - Subcutaneous
CADM2		7.30E-08	-0.24	Adipose - Visceral (Omentum)
CADM2		5.00E-07	-0.4	Heart - Left Ventricle
CADM2	rs7629091	2.10E-17	-0.4	Lung
CADM2-AS1		2.40E-06	0.54	Brain - Caudate (basal ganglia)
CADM2-AS1		2.40E-05	0.48	Brain - Cerebellar Hemisphere
CADM2-AS1		4.70E-08	0.5	Brain - Cerebellum
CADM2-AS1	rs7629375	1.20E-04	0.25	Nerve - Tibial
CADM2		8.80E-06	0.32	Brain - Hippocampus
CADM2	rs7630382	6.30E-05	0.14	Muscle - Skeletal
CADM2	rs76306335	9.20E-06	-0.66	Brain - Hippocampus
CADM2-AS1		7.20E-06	0.59	Brain - Caudate (basal ganglia)
CADM2-AS1		1.20E-06	0.61	Brain - Cerebellar Hemisphere
CADM2-AS1		9.20E-06	0.44	Brain - Cerebellum
CADM2-AS1		2.50E-05	0.5	Brain - Cortex
CADM2-AS1		4.80E-06	0.59	Brain - Frontal Cortex (BA9)
CADM2-AS1	rs7630752	3.80E-06	0.58	Brain - Nucleus accumbens (basal ganglia)
CADM2		3.30E-06	-0.2	Adipose - Subcutaneous
CADM2	rs7632056	3.80E-11	-0.32	Lung
CADM2		4.20E-05	0.19	Adipose - Subcutaneous
CADM2		1.70E-06	0.59	Brain - Spinal cord (cervical c-1)
CADM2	rs7632161	5.90E-06	0.18	Muscle - Skeletal
CADM2-AS1		1.30E-05	0.56	Brain - Caudate (basal ganglia)
CADM2-AS1		5.20E-08	0.65	Brain - Cerebellar Hemisphere
CADM2-AS1		3.40E-08	0.51	Brain - Cerebellum
CADM2-AS1		1.30E-05	0.5	Brain - Cortex
CADM2-AS1		4.80E-06	0.57	Brain - Frontal Cortex (BA9)
CADM2-AS1	rs7632162	4.60E-06	0.55	Brain - Nucleus accumbens (basal ganglia)
CADM2	rs7632628	1.30E-04	0.15	Adipose - Subcutaneous
CADM2	rs7632909	1.50E-04	0.15	Adipose - Subcutaneous
CADM2	rs7633256	1.00E-04	0.23	Muscle - Skeletal
CADM2-AS1		3.80E-05	0.55	Brain - Caudate (basal ganglia)
CADM2-AS1		2.20E-07	0.64	Brain - Cerebellar Hemisphere
CADM2-AS1		2.60E-08	0.54	Brain - Cerebellum
CADM2-AS1		2.00E-05	0.49	Brain - Cortex
CADM2-AS1		1.10E-06	0.61	Brain - Frontal Cortex (BA9)
CADM2-AS1	rs7634560	1.30E-06	0.57	Brain - Nucleus accumbens (basal ganglia)
CADM2		3.40E-11	-0.28	Adipose - Subcutaneous
CADM2		4.40E-08	-0.24	Adipose - Visceral (Omentum)
CADM2		2.20E-06	-0.37	Heart - Left Ventricle
CADM2	rs7634761	4.50E-18	-0.4	Lung
CADM2	rs7635835	5.80E-05	0.18	Adipose - Subcutaneous

CADM2		2.70E-06	0.57	Brain - Spinal cord (cervical c-1)
CADM2		2.00E-06	0.19	Muscle - Skeletal
CADM2		1.20E-05	0.17	Adipose - Subcutaneous
CADM2	rs7636138	2.80E-05	0.14	Muscle - Skeletal
CADM2-AS1		8.10E-06	0.59	Brain - Caudate (basal ganglia)
CADM2-AS1		7.10E-08	0.65	Brain - Cerebellar Hemisphere
CADM2-AS1		2.80E-07	0.5	Brain - Cerebellum
CADM2-AS1		4.20E-06	0.59	Brain - Frontal Cortex (BA9)
CADM2-AS1	rs7636206	7.50E-06	0.55	Brain - Nucleus accumbens (basal ganglia)
CADM2		2.70E-10	-0.26	Adipose - Subcutaneous
CADM2		3.30E-08	-0.24	Adipose - Visceral (Omentum)
CADM2		2.10E-07	-0.4	Heart - Left Ventricle
CADM2		1.00E-14	-0.36	Lung
CADM2	rs7636243	7.10E-05	-0.15	Muscle - Skeletal
CADM2-AS1		2.70E-05	0.53	Brain - Caudate (basal ganglia)
CADM2-AS1		9.40E-07	0.58	Brain - Cerebellar Hemisphere
CADM2-AS1		6.10E-08	0.52	Brain - Cerebellum
CADM2-AS1		2.90E-05	0.53	Brain - Frontal Cortex (BA9)
CADM2-AS1	rs7637221	1.90E-05	0.5	Brain - Nucleus accumbens (basal ganglia)
CADM2		6.80E-12	-0.29	Adipose - Subcutaneous
CADM2		1.50E-08	-0.25	Adipose - Visceral (Omentum)
CADM2		7.40E-07	-0.39	Heart - Left Ventricle
CADM2	rs7637527	1.80E-18	-0.41	Lung
CADM2		3.60E-06	-0.2	Adipose - Subcutaneous
CADM2	rs7637879	3.40E-09	-0.27	Lung
CADM2-AS1		2.40E-06	0.54	Brain - Cerebellar Hemisphere
CADM2-AS1		1.10E-08	0.51	Brain - Cerebellum
CADM2-AS1	rs7638774	2.70E-05	0.5	Brain - Nucleus accumbens (basal ganglia)
CADM2-AS1	rs7638804	2.60E-06	-0.45	Nerve - Tibial
CADM2		2.70E-10	-0.26	Adipose - Subcutaneous
CADM2		3.30E-08	-0.24	Adipose - Visceral (Omentum)
CADM2		2.10E-07	-0.4	Heart - Left Ventricle
CADM2		1.00E-14	-0.36	Lung
CADM2	rs7638953	7.10E-05	-0.15	Muscle - Skeletal
CADM2-AS1		5.40E-07	0.66	Brain - Caudate (basal ganglia)
CADM2-AS1		2.80E-08	0.67	Brain - Cerebellar Hemisphere
CADM2-AS1		3.10E-07	0.5	Brain - Cerebellum
CADM2-AS1		5.40E-06	0.52	Brain - Cortex
CADM2-AS1		4.30E-07	0.63	Brain - Frontal Cortex (BA9)
CADM2-AS1	rs7640041	6.00E-07	0.59	Brain - Nucleus accumbens (basal ganglia)
CADM2	rs7640660	1.10E-04	0.2	Adipose - Subcutaneous
CADM2		1.40E-09	-0.25	Adipose - Subcutaneous
CADM2		1.10E-07	-0.24	Adipose - Visceral (Omentum)
CADM2		2.00E-07	-0.39	Heart - Left Ventricle
CADM2	rs7640828	8.20E-20	-0.42	Lung
CADM2-AS1		1.00E-05	0.57	Brain - Caudate (basal ganglia)
CADM2-AS1		1.20E-08	0.66	Brain - Cerebellar Hemisphere
CADM2-AS1		7.60E-08	0.5	Brain - Cerebellum
CADM2-AS1	rs7642281	4.70E-06	0.51	Brain - Cortex

CADM2-AS1		1.30E-06	0.59	Brain - Frontal Cortex (BA9)
CADM2-AS1		6.80E-07	0.58	Brain - Nucleus accumbens (basal ganglia)
CADM2		1.00E-04	0.2	Adipose - Subcutaneous
CADM2	rs7643091	1.80E-06	0.27	Lung
CADM2-AS1		3.80E-05	0.5	Brain - Cerebellar Hemisphere
CADM2-AS1	rs7643464	3.10E-05	0.48	Brain - Nucleus accumbens (basal ganglia)
CADM2	rs76439809	2.80E-05	0.29	Lung
CADM2-AS1		1.70E-06	0.57	Brain - Caudate (basal ganglia)
CADM2-AS1		4.20E-05	0.48	Brain - Cerebellar Hemisphere
CADM2-AS1		3.90E-08	0.49	Brain - Cerebellum
CADM2-AS1	rs7644190	9.80E-06	0.5	Brain - Nucleus accumbens (basal ganglia)
CADM2		4.20E-12	-0.29	Adipose - Subcutaneous
CADM2		8.30E-09	-0.26	Adipose - Visceral (Omentum)
CADM2		9.90E-07	-0.38	Heart - Left Ventricle
CADM2	rs764523228	1.60E-17	-0.4	Lung
CADM2		2.50E-05	0.61	Brain - Spinal cord (cervical c-1)
CADM2	rs7645633	1.10E-05	0.16	Muscle - Skeletal
CADM2		5.20E-05	0.17	Adipose - Subcutaneous
CADM2	rs7645952	3.60E-07	0.19	Muscle - Skeletal
CADM2	rs7646739	1.50E-04	0.15	Adipose - Subcutaneous
CADM2	rs7647649	1.10E-06	0.18	Muscle - Skeletal
CADM2		2.20E-09	-0.25	Adipose - Subcutaneous
CADM2		2.60E-07	-0.23	Adipose - Visceral (Omentum)
CADM2		8.50E-07	-0.38	Heart - Left Ventricle
CADM2	rs7647981	5.30E-19	-0.41	Lung
CADM2		4.20E-05	0.19	Adipose - Subcutaneous
CADM2		2.50E-06	0.57	Brain - Spinal cord (cervical c-1)
CADM2	rs7648172	3.30E-06	0.18	Muscle - Skeletal
CADM2-AS1		3.80E-05	0.53	Brain - Caudate (basal ganglia)
CADM2-AS1		1.70E-07	0.62	Brain - Cerebellar Hemisphere
CADM2-AS1		8.50E-08	0.5	Brain - Cerebellum
CADM2-AS1		2.30E-05	0.47	Brain - Cortex
CADM2-AS1		8.70E-07	0.6	Brain - Frontal Cortex (BA9)
CADM2-AS1	rs7648895	5.70E-06	0.53	Brain - Nucleus accumbens (basal ganglia)
CADM2		3.60E-05	0.18	Adipose - Subcutaneous
CADM2	rs7649296	1.50E-04	0.14	Muscle - Skeletal
CADM2		2.70E-10	-0.26	Adipose - Subcutaneous
CADM2		3.30E-08	-0.24	Adipose - Visceral (Omentum)
CADM2		2.10E-07	-0.4	Heart - Left Ventricle
CADM2		1.00E-14	-0.36	Lung
CADM2	rs7650284	7.10E-05	-0.15	Muscle - Skeletal
CADM2-AS1		6.60E-07	0.62	Brain - Cerebellar Hemisphere
CADM2-AS1		1.40E-07	0.51	Brain - Cerebellum
CADM2-AS1		6.00E-06	0.51	Brain - Cortex
CADM2-AS1		1.70E-06	0.61	Brain - Frontal Cortex (BA9)
CADM2-AS1	rs7650420	2.60E-06	0.56	Brain - Nucleus accumbens (basal ganglia)
CADM2		2.60E-08	0.26	Adipose - Subcutaneous
CADM2		2.20E-05	0.22	Adipose - Visceral (Omentum)
CADM2	rs7650834	1.20E-06	0.27	Lung
CADM2	rs76508707	1.30E-09	-0.24	Adipose - Subcutaneous

CADM2		2.20E-05	-0.18	Adipose - Visceral (Omentum)
CADM2		3.60E-08	-0.26	Lung
CADM2		3.90E-06	-0.16	Muscle - Skeletal
CADM2		2.80E-05	-0.46	Heart - Left Ventricle
CADM2	rs7651113	1.90E-06	-0.32	Lung
CADM2		7.20E-05	0.16	Adipose - Subcutaneous
CADM2	rs7651996	4.40E-05	0.14	Muscle - Skeletal
CADM2		9.70E-09	-0.24	Adipose - Subcutaneous
CADM2		2.20E-08	-0.26	Adipose - Visceral (Omentum)
CADM2		5.70E-07	-0.38	Heart - Left Ventricle
CADM2	rs7652683	1.90E-19	-0.41	Lung
CADM2		9.70E-09	-0.24	Adipose - Subcutaneous
CADM2		2.20E-08	-0.26	Adipose - Visceral (Omentum)
CADM2		5.70E-07	-0.38	Heart - Left Ventricle
CADM2	rs7652808	1.90E-19	-0.41	Lung
CADM2-AS1		4.10E-06	0.54	Brain - Cerebellar Hemisphere
CADM2-AS1		9.60E-08	0.5	Brain - Cerebellum
CADM2-AS1	rs7653470	2.60E-05	0.5	Brain - Nucleus accumbens (basal ganglia)
CADM2		1.00E-11	-0.28	Adipose - Subcutaneous
CADM2		1.40E-07	-0.24	Adipose - Visceral (Omentum)
CADM2		1.00E-06	-0.39	Heart - Left Ventricle
CADM2		6.50E-17	-0.4	Lung
CADM2	rs7653790	1.20E-04	0.1	Nerve - Tibial
CADM2	rs765461	5.40E-06	0.17	Muscle - Skeletal
CADM2	rs76617147	7.30E-06	-0.67	Brain - Hippocampus
CADM2	rs76633736	9.20E-06	-0.66	Brain - Hippocampus
CADM2	rs76668519	9.20E-06	-0.66	Brain - Hippocampus
CADM2		3.80E-06	-0.2	Adipose - Subcutaneous
CADM2	rs76791867	4.00E-09	-0.27	Lung
CADM2		7.20E-06	0.2	Adipose - Subcutaneous
CADM2		1.10E-08	0.71	Brain - Spinal cord (cervical c-1)
CADM2	rs76847001	2.10E-06	0.19	Muscle - Skeletal
CADM2-AS1	rs77025486	3.80E-05	0.99	Brain - Frontal Cortex (BA9)
CADM2-AS1	rs77036893	3.20E-05	0.97	Brain - Anterior cingulate cortex (BA24)
CADM2-AS1		3.80E-05	0.53	Brain - Caudate (basal ganglia)
CADM2-AS1		1.70E-07	0.62	Brain - Cerebellar Hemisphere
CADM2-AS1		8.50E-08	0.5	Brain - Cerebellum
CADM2-AS1		2.30E-05	0.47	Brain - Cortex
CADM2-AS1		8.70E-07	0.6	Brain - Frontal Cortex (BA9)
CADM2-AS1	rs77073785	5.70E-06	0.53	Brain - Nucleus accumbens (basal ganglia)
CADM2	rs77159288	9.20E-06	-0.66	Brain - Hippocampus
CADM2	rs77210894	1.70E-05	0.56	Heart - Left Ventricle
CADM2	rs77340049	2.60E-08	1	Heart - Left Ventricle
CADM2	rs77548471	1.00E-05	-0.65	Brain - Hippocampus
CADM2		4.30E-12	-0.29	Adipose - Subcutaneous
CADM2		1.00E-08	-0.26	Adipose - Visceral (Omentum)
CADM2		9.90E-07	-0.38	Heart - Left Ventricle
CADM2	rs775856991	2.20E-17	-0.4	Lung
CADM2		6.50E-12	-0.29	Adipose - Subcutaneous
CADM2		1.50E-08	-0.25	Adipose - Visceral (Omentum)
CADM2	rs77657121	7.40E-07	-0.39	Heart - Left Ventricle

CADM2		1.80E-18	-0.41	Lung
CADM2		1.60E-04	0.099	Nerve - Tibial
CADM2	rs77684423	8.50E-06	-0.68	Brain - Hippocampus
CADM2	rs77792514	2.10E-05	0.52	Heart - Left Ventricle
CADM2		6.80E-12	-0.29	Adipose - Subcutaneous
CADM2		1.50E-08	-0.25	Adipose - Visceral (Omentum)
CADM2		7.40E-07	-0.39	Heart - Left Ventricle
CADM2		1.80E-18	-0.41	Lung
CADM2	rs77852438	1.60E-04	0.099	Nerve - Tibial
CADM2	rs77906057	2.60E-07	0.98	Heart - Left Ventricle
CADM2	rs77922770	2.10E-05	0.52	Heart - Left Ventricle
CADM2		6.70E-12	-0.29	Adipose - Subcutaneous
CADM2		1.60E-08	-0.25	Adipose - Visceral (Omentum)
CADM2		7.40E-07	-0.39	Heart - Left Ventricle
CADM2		1.20E-18	-0.41	Lung
CADM2	rs77984526	1.60E-04	0.099	Nerve - Tibial
CADM2		6.20E-12	-0.29	Adipose - Subcutaneous
CADM2		1.60E-08	-0.25	Adipose - Visceral (Omentum)
CADM2		2.00E-06	-0.37	Heart - Left Ventricle
CADM2	rs78015688	5.30E-19	-0.41	Lung
CADM2	rs78069683	2.90E-05	0.2	Brain - Cerebellum
CADM2	rs78192226	6.40E-05	0.35	Adipose - Subcutaneous
CADM2	rs78201743	9.20E-06	-0.66	Brain - Hippocampus
CADM2		6.80E-12	-0.29	Adipose - Subcutaneous
CADM2		1.50E-08	-0.25	Adipose - Visceral (Omentum)
CADM2		7.40E-07	-0.39	Heart - Left Ventricle
CADM2		1.80E-18	-0.41	Lung
CADM2	rs78288623	1.60E-04	0.099	Nerve - Tibial
CADM2	rs78430777	2.10E-05	0.52	Heart - Left Ventricle
CADM2	rs78459071	8.60E-06	0.18	Muscle - Skeletal
CADM2-AS1	rs78484867	6.00E-05	-0.29	Nerve - Tibial
CADM2		6.80E-12	-0.29	Adipose - Subcutaneous
CADM2		1.40E-08	-0.25	Adipose - Visceral (Omentum)
CADM2		7.00E-07	-0.39	Heart - Left Ventricle
CADM2		1.60E-18	-0.41	Lung
CADM2	rs78661387	1.60E-04	0.1	Nerve - Tibial
CADM2-AS1	rs78662240	1.40E-04	-0.36	Nerve - Tibial
CADM2	rs78735252	9.20E-06	-0.66	Brain - Hippocampus
CADM2	rs78764344	1.90E-08	1	Heart - Left Ventricle
CADM2	rs78775281	5.70E-06	-0.69	Brain - Hippocampus
CADM2	rs78950219	7.10E-05	0.54	Heart - Atrial Appendage
CADM2-AS1		4.00E-05	-0.47	Brain - Cerebellar Hemisphere
CADM2-AS1	rs79170600	2.50E-05	-0.5	Brain - Nucleus accumbens (basal ganglia)
CADM2-AS1	rs79188076	4.40E-05	1.5	Brain - Frontal Cortex (BA9)
CADM2	rs79321616	7.40E-06	-0.66	Brain - Hippocampus
CADM2	rs79360418	9.20E-06	-0.66	Brain - Hippocampus
CADM2	rs79395252	9.20E-06	-0.66	Brain - Hippocampus
CADM2	rs79398710	1.50E-04	0.4	Adipose - Subcutaneous
CADM2		1.00E-04	0.18	Lung
CADM2	rs796609163	3.90E-05	0.15	Muscle - Skeletal
CADM2	rs796862844	2.80E-05	-0.59	Brain - Hippocampus
CADM2-AS1	rs79750320	4.60E-05	-0.29	Nerve - Tibial
CADM2	rs79953206	9.20E-06	-0.66	Brain - Hippocampus

CADM2-AS1	rs79953227	8.80E-05	-0.28	Nerve - Tibial
CADM2	rs79961087	1.10E-07	0.92	Heart - Left Ventricle
CADM2	rs79994609	2.00E-07	1	Heart - Left Ventricle
CADM2	rs80025546	8.10E-08	-0.35	Lung
CADM2	rs80305277	1.30E-08	1.1	Heart - Left Ventricle
CADM2	rs80309450	2.60E-08	1	Heart - Left Ventricle
CADM2	rs80354540	9.20E-06	-0.66	Brain - Hippocampus
CADM2	rs818215	9.40E-06	0.18	Adipose - Subcutaneous
CADM2		3.80E-06	0.19	Adipose - Subcutaneous
CADM2	rs818219	1.20E-04	0.14	Muscle - Skeletal
CADM2	rs818225	4.20E-05	0.17	Adipose - Subcutaneous
CADM2		9.00E-06	0.62	Brain - Spinal cord (cervical c-1)
CADM2	rs869074626	1.90E-06	0.17	Muscle - Skeletal
CADM2-AS1		3.30E-05	0.63	Brain - Anterior cingulate cortex (BA24)
CADM2-AS1		2.60E-05	0.54	Brain - Caudate (basal ganglia)
CADM2-AS1		2.40E-06	0.58	Brain - Cerebellar Hemisphere
CADM2-AS1		1.40E-08	0.52	Brain - Cerebellum
CADM2-AS1		4.20E-05	0.51	Brain - Frontal Cortex (BA9)
CADM2-AS1	rs869256184	1.90E-06	0.57	Brain - Nucleus accumbens (basal ganglia)
CADM2		1.00E-04	0.18	Adipose - Subcutaneous
CADM2		2.50E-06	0.57	Brain - Spinal cord (cervical c-1)
CADM2	rs869274315	1.20E-06	0.19	Muscle - Skeletal
CADM2-AS1		1.50E-05	0.63	Brain - Caudate (basal ganglia)
CADM2-AS1		1.40E-06	0.66	Brain - Cerebellar Hemisphere
CADM2-AS1		2.80E-05	0.51	Brain - Cerebellum
CADM2-AS1	rs869281592	9.00E-07	0.71	Brain - Frontal Cortex (BA9)
CADM2-AS1	rs883776	8.90E-05	-0.29	Nerve - Tibial
CADM2	rs892365	8.30E-05	-0.43	Heart - Left Ventricle
CADM2	rs892365	4.30E-07	-0.33	Lung
CADM2	rs9284802	1.40E-05	0.15	Muscle - Skeletal
CADM2		3.40E-05	0.18	Adipose - Subcutaneous
CADM2		2.60E-05	0.26	Brain - Caudate (basal ganglia)
CADM2		7.80E-07	0.69	Brain - Spinal cord (cervical c-1)
CADM2	rs9309967	1.40E-05	0.17	Muscle - Skeletal
CADM2		5.20E-05	0.16	Adipose - Subcutaneous
CADM2	rs9309969	1.20E-05	0.15	Muscle - Skeletal
CADM2		8.20E-05	0.16	Adipose - Subcutaneous
CADM2	rs9309970	4.70E-05	0.14	Muscle - Skeletal
CADM2	rs9309974	6.70E-06	-0.66	Testis
CADM2	rs9309976	1.50E-05	-0.75	Testis
CADM2		9.10E-09	0.27	Adipose - Subcutaneous
CADM2		1.30E-05	0.22	Adipose - Visceral (Omentum)
CADM2	rs9309977	1.20E-06	0.27	Lung
CADM2	rs9309978	2.30E-07	-0.34	Lung
CADM2	rs9309979	1.70E-06	-0.32	Lung
CADM2		7.30E-08	0.26	Adipose - Subcutaneous
CADM2		1.30E-05	0.22	Adipose - Visceral (Omentum)
CADM2	rs9309980	9.40E-07	0.28	Lung
CADM2		1.00E-08	0.28	Adipose - Subcutaneous
CADM2		4.40E-05	0.21	Adipose - Visceral (Omentum)
CADM2	rs9309981	1.00E-06	0.28	Lung

CADM2		1.10E-04	-0.12	Nerve - Tibial
CADM2		1.60E-08	0.27	Adipose - Subcutaneous
CADM2		1.50E-05	0.22	Adipose - Visceral (Omentum)
CADM2	rs9309982	1.20E-06	0.27	Lung
CADM2		8.90E-05	0.2	Adipose - Subcutaneous
CADM2		3.20E-05	0.25	Lung
CADM2-AS1	rs9309985	3.90E-05	-0.31	Nerve - Tibial
CADM2		6.80E-05	0.23	Adipose - Visceral (Omentum)
CADM2	rs9309986	2.60E-06	0.28	Lung
CADM2-AS1		2.90E-05	0.53	Brain - Caudate (basal ganglia)
CADM2-AS1		6.50E-07	0.59	Brain - Cerebellar Hemisphere
CADM2-AS1		8.00E-08	0.52	Brain - Cerebellum
CADM2-AS1		2.10E-05	0.53	Brain - Frontal Cortex (BA9)
CADM2-AS1	rs9309992	2.00E-05	0.5	Brain - Nucleus accumbens (basal ganglia)
CADM2-AS1		7.60E-06	0.61	Brain - Caudate (basal ganglia)
CADM2-AS1		1.00E-08	0.69	Brain - Cerebellar Hemisphere
CADM2-AS1		1.20E-06	0.49	Brain - Cerebellum
CADM2-AS1		1.10E-05	0.51	Brain - Cortex
CADM2-AS1		8.20E-07	0.61	Brain - Frontal Cortex (BA9)
CADM2-AS1	rs9309996	1.80E-06	0.58	Brain - Nucleus accumbens (basal ganglia)
CADM2-AS1		9.70E-08	0.66	Brain - Cerebellar Hemisphere
CADM2-AS1		8.60E-08	0.52	Brain - Cerebellum
CADM2-AS1		5.00E-06	0.52	Brain - Cortex
CADM2-AS1		3.40E-07	0.64	Brain - Frontal Cortex (BA9)
CADM2-AS1	rs9310000	9.00E-07	0.59	Brain - Nucleus accumbens (basal ganglia)
CADM2		2.90E-11	-0.28	Adipose - Subcutaneous
CADM2		3.60E-07	-0.23	Adipose - Visceral (Omentum)
CADM2		6.00E-07	-0.39	Heart - Left Ventricle
CADM2	rs956281	2.80E-17	-0.4	Lung
CADM2		7.10E-06	-0.19	Adipose - Subcutaneous
CADM2	rs960986	1.80E-09	-0.27	Lung
CADM2		4.80E-05	0.19	Adipose - Subcutaneous
CADM2		1.40E-06	0.68	Brain - Spinal cord (cervical c-1)
CADM2	rs9647393	2.60E-05	0.17	Muscle - Skeletal
CADM2		1.70E-05	0.2	Adipose - Subcutaneous
CADM2		6.20E-06	0.59	Brain - Spinal cord (cervical c-1)
CADM2	rs9654015	2.10E-06	0.2	Muscle - Skeletal
CADM2		6.80E-05	-0.43	Heart - Left Ventricle
CADM2	rs9654023	2.80E-07	-0.34	Lung
CADM2-AS1		2.40E-06	0.54	Brain - Cerebellar Hemisphere
CADM2-AS1		1.10E-08	0.51	Brain - Cerebellum
CADM2-AS1	rs9681744	2.70E-05	0.5	Brain - Nucleus accumbens (basal ganglia)
CADM2	rs9682566	1.60E-04	0.16	Adipose - Subcutaneous
CADM2-AS1		1.10E-06	0.59	Brain - Caudate (basal ganglia)
CADM2-AS1		1.90E-10	0.72	Brain - Cerebellar Hemisphere
CADM2-AS1		8.30E-11	0.6	Brain - Cerebellum
CADM2-AS1		1.10E-06	0.57	Brain - Frontal Cortex (BA9)
CADM2-AS1		2.30E-05	0.56	Brain - Hippocampus
CADM2-AS1	rs9713669	4.30E-08	0.62	Brain - Nucleus accumbens (basal ganglia)

CADM2-AS1		4.20E-05	0.26	Nerve - Tibial
CADM2-AS1		3.90E-08	0.66	Brain - Cerebellar Hemisphere
CADM2-AS1		8.80E-08	0.51	Brain - Cerebellum
CADM2-AS1		8.70E-06	0.5	Brain - Cortex
CADM2-AS1		5.00E-07	0.62	Brain - Frontal Cortex (BA9)
CADM2-AS1	rs9713674	3.60E-06	0.54	Brain - Nucleus accumbens (basal ganglia)
CADM2		8.90E-07	-0.21	Adipose - Subcutaneous
CADM2		6.00E-09	-0.27	Lung
CADM2-AS1	rs9713905	1.40E-04	0.25	Nerve - Tibial
CADM2-AS1		1.30E-05	0.57	Brain - Caudate (basal ganglia)
CADM2-AS1		6.20E-08	0.66	Brain - Cerebellar Hemisphere
CADM2-AS1		1.70E-07	0.5	Brain - Cerebellum
CADM2-AS1		9.90E-06	0.5	Brain - Cortex
CADM2-AS1		1.30E-06	0.61	Brain - Frontal Cortex (BA9)
CADM2-AS1	rs9714165	4.60E-06	0.55	Brain - Nucleus accumbens (basal ganglia)
CADM2	rs9754640	6.10E-05	0.41	Heart - Left Ventricle
CADM2	rs9809258	7.20E-06	0.19	Adipose - Subcutaneous
CADM2	rs9809258	1.10E-04	0.2	Lung
CADM2-AS1	rs9809586	2.50E-05	-0.29	Nerve - Tibial
CADM2		1.00E-07	0.28	Adipose - Subcutaneous
CADM2		1.10E-05	0.25	Adipose - Visceral (Omentum)
CADM2	rs9810724	1.60E-08	0.33	Lung
CADM2-AS1		3.70E-05	0.55	Brain - Caudate (basal ganglia)
CADM2-AS1		1.60E-07	0.65	Brain - Cerebellar Hemisphere
CADM2-AS1		4.30E-08	0.53	Brain - Cerebellum
CADM2-AS1		2.00E-05	0.49	Brain - Cortex
CADM2-AS1		1.10E-06	0.61	Brain - Frontal Cortex (BA9)
CADM2-AS1	rs9810861	1.30E-06	0.57	Brain - Nucleus accumbens (basal ganglia)
CADM2		1.00E-04	0.18	Adipose - Subcutaneous
CADM2	rs9811546	1.10E-06	0.72	Brain - Spinal cord (cervical c-1)
CADM2		1.20E-05	0.62	Brain - Spinal cord (cervical c-1)
CADM2	rs9812061	1.60E-06	0.18	Muscle - Skeletal
CADM2-AS1		5.00E-06	-0.56	Brain - Caudate (basal ganglia)
CADM2-AS1		1.60E-09	-0.7	Brain - Cerebellar Hemisphere
CADM2-AS1		3.00E-11	-0.62	Brain - Cerebellum
CADM2-AS1		5.20E-06	-0.55	Brain - Frontal Cortex (BA9)
CADM2-AS1		2.00E-05	-0.56	Brain - Hippocampus
CADM2-AS1		6.20E-08	-0.62	Brain - Nucleus accumbens (basal ganglia)
CADM2-AS1		3.20E-05	-0.54	Brain - Putamen (basal ganglia)
CADM2-AS1	rs9812779	3.10E-05	-0.26	Nerve - Tibial
CADM2		7.20E-06	0.2	Adipose - Subcutaneous
CADM2		1.10E-08	0.71	Brain - Spinal cord (cervical c-1)
CADM2	rs9812833	1.50E-06	0.19	Muscle - Skeletal
CADM2		2.40E-05	0.25	Lung
CADM2-AS1	rs9812845	1.30E-04	-0.28	Nerve - Tibial
CADM2		4.60E-05	0.22	Adipose - Subcutaneous
CADM2	rs9813436	4.70E-08	0.32	Lung
CADM2		2.20E-08	0.26	Adipose - Subcutaneous
CADM2	rs9813512	1.80E-05	0.22	Adipose - Visceral (Omentum)

CADM2		1.20E-06	0.27	Lung
CADM2-AS1	rs9814386	6.40E-05	-0.28	Nerve - Tibial
CADM2		7.40E-05	0.16	Adipose - Subcutaneous
CADM2	rs9814390	3.30E-06	0.17	Muscle - Skeletal
CADM2		1.90E-07	0.24	Adipose - Subcutaneous
CADM2		8.20E-05	0.2	Adipose - Visceral (Omentum)
CADM2		4.90E-05	0.21	Breast - Mammary Tissue
CADM2	rs9814516	2.10E-06	0.26	Lung
CADM2		9.30E-05	0.15	Adipose - Subcutaneous
CADM2	rs9814600	3.00E-05	0.14	Muscle - Skeletal
CADM2		1.40E-05	0.16	Adipose - Subcutaneous
CADM2	rs9814835	3.40E-05	0.14	Muscle - Skeletal
CADM2-AS1	rs9814869	8.40E-05	-0.28	Nerve - Tibial
CADM2		9.80E-09	0.27	Adipose - Subcutaneous
CADM2		2.30E-05	0.22	Adipose - Visceral (Omentum)
CADM2	rs9814919	1.20E-06	0.27	Lung
CADM2		1.40E-05	0.19	Adipose - Subcutaneous
CADM2		1.90E-05	0.26	Brain - Caudate (basal ganglia)
CADM2		2.50E-06	0.62	Brain - Spinal cord (cervical c-1)
CADM2	rs9815258	4.80E-07	0.19	Muscle - Skeletal
CADM2		7.20E-05	0.17	Adipose - Subcutaneous
CADM2	rs9816536	1.10E-11	0.33	Lung
CADM2		1.80E-05	-0.19	Muscle - Skeletal
CADM2		3.80E-05	0.17	Adipose - Subcutaneous
CADM2	rs9816552	5.00E-05	0.14	Muscle - Skeletal
CADM2-AS1		6.50E-05	0.45	Brain - Cerebellar Hemisphere
CADM2-AS1	rs9817232	1.70E-05	0.48	Brain - Nucleus accumbens (basal ganglia)
CADM2-AS1		3.40E-05	0.54	Brain - Cerebellar Hemisphere
CADM2-AS1		2.10E-06	0.47	Brain - Cerebellum
CADM2-AS1	rs9817262	4.70E-05	0.47	Brain - Cortex
CADM2	rs9818067	9.20E-06	-0.66	Brain - Hippocampus
CADM2	rs9818122	5.70E-05	0.22	Adipose - Subcutaneous
CADM2	rs9818381	2.90E-05	-0.18	Muscle - Skeletal
CADM2		3.40E-05	0.18	Adipose - Subcutaneous
CADM2		4.30E-06	0.62	Brain - Spinal cord (cervical c-1)
CADM2	rs9818659	6.80E-07	0.19	Muscle - Skeletal
CADM2	rs9819278	8.10E-06	0.16	Muscle - Skeletal
CADM2		5.00E-05	0.21	Adipose - Subcutaneous
CADM2		5.60E-07	0.3	Lung
CADM2-AS1	rs9819476	8.30E-05	-0.3	Nerve - Tibial
CADM2		4.20E-08	0.26	Adipose - Subcutaneous
CADM2		1.70E-05	0.22	Adipose - Visceral (Omentum)
CADM2	rs9819830	1.50E-06	0.27	Lung
CADM2		4.30E-07	0.26	Adipose - Subcutaneous
CADM2		2.00E-05	0.24	Adipose - Visceral (Omentum)
CADM2	rs9820228	7.00E-09	0.34	Lung
CADM2		2.40E-05	0.16	Adipose - Subcutaneous
CADM2	rs9820587	9.70E-05	0.14	Muscle - Skeletal
CADM2		2.70E-08	0.27	Adipose - Subcutaneous
CADM2		1.90E-05	0.22	Adipose - Visceral (Omentum)
CADM2	rs9821041	1.20E-06	0.27	Lung
CADM2	rs9821126	1.10E-04	0.35	Muscle - Skeletal
CADM2	rs9821807	8.70E-05	0.18	Adipose - Subcutaneous

CADM2		2.30E-05	0.27	Brain - Caudate (basal ganglia)
CADM2		1.20E-06	0.68	Brain - Spinal cord (cervical c-1)
CADM2		1.30E-05	0.17	Muscle - Skeletal
CADM2		2.20E-07	0.27	Adipose - Subcutaneous
CADM2		1.50E-05	0.24	Adipose - Visceral (Omentum)
CADM2	rs9822731	2.50E-09	0.35	Lung
CADM2		7.80E-06	0.19	Adipose - Subcutaneous
CADM2		5.30E-06	0.28	Brain - Caudate (basal ganglia)
CADM2		1.50E-05	0.6	Brain - Spinal cord (cervical c-1)
CADM2	rs9823454	6.10E-07	0.19	Muscle - Skeletal
CADM2	rs9824071	4.50E-07	-0.33	Lung
CADM2-AS1		2.70E-06	0.61	Brain - Caudate (basal ganglia)
CADM2-AS1		5.00E-07	0.61	Brain - Cerebellar Hemisphere
CADM2-AS1		8.90E-08	0.5	Brain - Cerebellum
CADM2-AS1		2.40E-06	0.51	Brain - Cortex
CADM2-AS1		5.90E-06	0.57	Brain - Frontal Cortex (BA9)
CADM2-AS1	rs9824085	3.10E-06	0.55	Brain - Nucleus accumbens (basal ganglia)
CADM2		1.60E-07	-0.23	Adipose - Subcutaneous
CADM2		1.20E-07	-0.26	Adipose - Visceral (Omentum)
CADM2		3.30E-06	-0.23	Breast - Mammary Tissue
CADM2	rs9824178	2.80E-09	-0.31	Lung
CADM2		5.60E-06	-0.22	Lung
CADM2-AS1	rs9824301	1.90E-05	0.28	Nerve - Tibial
CADM2	rs9824386	1.20E-06	-0.32	Lung
CADM2	rs9824692	2.80E-06	-0.34	Lung
CADM2		6.00E-05	-0.42	Heart - Left Ventricle
CADM2	rs9824840	5.20E-06	-0.3	Lung
CADM2		8.80E-05	-0.43	Heart - Left Ventricle
CADM2	rs9825345	2.30E-07	-0.34	Lung
CADM2-AS1	rs9825758	1.80E-06	-0.45	Nerve - Tibial
CADM2	rs9825885	1.80E-05	0.27	Lung
CADM2		7.00E-08	0.28	Adipose - Subcutaneous
CADM2		3.60E-06	0.26	Adipose - Visceral (Omentum)
CADM2	rs9826458	4.10E-09	0.34	Lung
CADM2-AS1	rs9826482	1.20E-04	-0.32	Nerve - Tibial
CADM2		6.60E-05	0.17	Adipose - Subcutaneous
CADM2		6.40E-05	0.32	Heart - Left Ventricle
CADM2	rs9826759	7.70E-12	0.33	Lung
CADM2		2.70E-05	0.19	Adipose - Subcutaneous
CADM2		1.70E-06	0.59	Brain - Spinal cord (cervical c-1)
CADM2	rs9827318	1.50E-06	0.19	Muscle - Skeletal
CADM2-AS1	rs9827621	6.90E-05	-0.29	Nerve - Tibial
CADM2		2.70E-05	0.19	Adipose - Subcutaneous
CADM2		1.70E-06	0.59	Brain - Spinal cord (cervical c-1)
CADM2	rs9827763	1.50E-06	0.19	Muscle - Skeletal
CADM2		7.00E-05	0.19	Adipose - Subcutaneous
CADM2		3.70E-07	0.65	Brain - Spinal cord (cervical c-1)
CADM2	rs9827900	1.70E-05	0.18	Muscle - Skeletal
CADM2		3.40E-05	0.19	Adipose - Subcutaneous
CADM2		2.60E-05	0.26	Brain - Caudate (basal ganglia)
CADM2		7.80E-07	0.69	Brain - Spinal cord (cervical c-1)
CADM2	rs9828496	1.40E-05	0.17	Muscle - Skeletal

CADM2		1.30E-07	0.28	Adipose - Subcutaneous
CADM2		1.70E-05	0.24	Adipose - Visceral (Omentum)
CADM2	rs9828679	7.00E-09	0.34	Lung
CADM2-AS1	rs9828703	8.10E-05	-0.36	Nerve - Tibial
CADM2	rs9829032	1.90E-08	0.27	Lung
CADM2-AS1	rs9829298	1.50E-05	-0.39	Nerve - Tibial
CADM2-AS1		1.50E-05	0.56	Brain - Caudate (basal ganglia)
CADM2-AS1		2.70E-07	0.62	Brain - Cerebellar Hemisphere
CADM2-AS1		1.80E-07	0.5	Brain - Cerebellum
CADM2-AS1		1.80E-06	0.59	Brain - Frontal Cortex (BA9)
CADM2-AS1	rs9829446	6.40E-06	0.53	Brain - Nucleus accumbens (basal ganglia)
CADM2	rs9830359	1.00E-05	-0.31	Lung
CADM2-AS1		5.80E-06	0.58	Brain - Caudate (basal ganglia)
CADM2-AS1		1.70E-06	0.58	Brain - Cerebellar Hemisphere
CADM2-AS1		1.70E-07	0.52	Brain - Cerebellum
CADM2-AS1	rs9830554	2.40E-05	0.53	Brain - Frontal Cortex (BA9)
CADM2		3.90E-05	0.17	Adipose - Subcutaneous
CADM2	rs9831123	6.20E-05	0.14	Muscle - Skeletal
CADM2		1.10E-04	0.19	Adipose - Subcutaneous
CADM2	rs9831374	3.30E-07	0.77	Brain - Spinal cord (cervical c-1)
CADM2		6.40E-05	0.17	Adipose - Subcutaneous
CADM2		9.10E-05	0.31	Heart - Left Ventricle
CADM2	rs9831610	2.70E-12	0.33	Lung
CADM2	rs9831848	6.90E-08	-0.35	Lung
CADM2		5.00E-06	0.21	Adipose - Subcutaneous
CADM2	rs9832119	8.70E-07	0.2	Muscle - Skeletal
CADM2	rs9833049	6.10E-05	-0.25	Lung
CADM2	rs9833081	3.10E-05	0.15	Muscle - Skeletal
CADM2		7.00E-05	0.19	Adipose - Subcutaneous
CADM2		3.70E-07	0.65	Brain - Spinal cord (cervical c-1)
CADM2	rs9833312	1.70E-05	0.18	Muscle - Skeletal
CADM2		4.20E-08	0.26	Adipose - Subcutaneous
CADM2		1.90E-05	0.22	Adipose - Visceral (Omentum)
CADM2	rs9833314	8.00E-07	0.28	Lung
CADM2	rs9833391	2.40E-06	-0.3	Lung
CADM2	rs9833972	1.30E-05	-0.74	Testis
CADM2		1.80E-05	0.17	Adipose - Subcutaneous
CADM2	rs9834688	6.00E-05	0.14	Muscle - Skeletal
CADM2		1.20E-04	0.2	Adipose - Subcutaneous
CADM2		9.50E-05	0.22	Adipose - Visceral (Omentum)
CADM2	rs9834708	2.10E-06	0.28	Lung
CADM2-AS1		2.20E-05	0.52	Brain - Cerebellar Hemisphere
CADM2-AS1	rs9835176	1.40E-05	0.51	Brain - Nucleus accumbens (basal ganglia)
CADM2		2.70E-08	0.26	Adipose - Subcutaneous
CADM2		2.80E-05	0.21	Adipose - Visceral (Omentum)
CADM2	rs9835441	8.20E-07	0.28	Lung
CADM2		3.30E-06	0.21	Adipose - Subcutaneous
CADM2		9.40E-09	0.71	Brain - Spinal cord (cervical c-1)
CADM2	rs9835724	1.50E-06	0.19	Muscle - Skeletal
CADM2		5.10E-05	0.23	Adipose - Visceral (Omentum)
CADM2	rs9835772	6.40E-06	0.27	Lung

CADM2		4.20E-06	0.33	Brain - Hippocampus
CADM2	rs9835904	8.80E-05	0.14	Muscle - Skeletal
CADM2	rs9836020	2.00E-05	-0.19	Muscle - Skeletal
CADM2	rs9836407	9.50E-06	-0.19	Muscle - Skeletal
CADM2	rs9836542	1.70E-05	-0.19	Muscle - Skeletal
CADM2-AS1		4.90E-06	0.53	Brain - Caudate (basal ganglia)
CADM2-AS1		4.50E-05	0.46	Brain - Cerebellar Hemisphere
CADM2-AS1		1.60E-07	0.48	Brain - Cerebellum
CADM2-AS1	rs9836755	3.40E-06	0.52	Brain - Nucleus accumbens (basal ganglia)
CADM2	rs9836888	3.00E-05	0.15	Muscle - Skeletal
CADM2		4.70E-05	0.21	Adipose - Subcutaneous
CADM2		1.10E-04	0.22	Adipose - Visceral (Omentum)
CADM2		8.50E-06	0.26	Lung
CADM2-AS1	rs9836967	1.30E-04	-0.29	Nerve - Tibial
CADM2		2.30E-06	0.72	Brain - Spinal cord (cervical c-1)
CADM2-AS1	rs9837462	1.40E-05	0.59	Brain - Caudate (basal ganglia)
CADM2	rs9838091	1.80E-05	0.31	Brain - Hippocampus
CADM2		3.00E-08	0.27	Adipose - Subcutaneous
CADM2		2.00E-05	0.22	Adipose - Visceral (Omentum)
CADM2	rs9838811	1.80E-06	0.27	Lung
CADM2		7.20E-05	0.15	Adipose - Subcutaneous
CADM2	rs9839194	6.40E-06	0.16	Muscle - Skeletal
CADM2		9.60E-06	0.19	Adipose - Subcutaneous
CADM2	rs9839708	1.90E-06	0.17	Muscle - Skeletal
CADM2		2.50E-05	-0.44	Heart - Left Ventricle
CADM2	rs9839731	2.80E-06	-0.31	Lung
CADM2		9.60E-07	0.2	Adipose - Subcutaneous
CADM2	rs9840607	6.80E-08	0.19	Muscle - Skeletal
CADM2		7.50E-05	0.23	Adipose - Visceral (Omentum)
CADM2	rs9840636	2.40E-06	0.29	Lung
CADM2		4.40E-06	-0.22	Lung
CADM2-AS1	rs9841044	3.20E-05	0.27	Nerve - Tibial
CADM2		2.60E-07	0.27	Adipose - Subcutaneous
CADM2		1.40E-05	0.25	Adipose - Visceral (Omentum)
CADM2	rs9841144	2.20E-09	0.36	Lung
CADM2-AS1		8.30E-08	0.64	Brain - Cerebellar Hemisphere
CADM2-AS1		1.10E-07	0.5	Brain - Cerebellum
CADM2-AS1		3.30E-06	0.51	Brain - Cortex
CADM2-AS1		5.70E-07	0.6	Brain - Frontal Cortex (BA9)
CADM2-AS1	rs9841158	7.10E-07	0.58	Brain - Nucleus accumbens (basal ganglia)
CADM2-AS1	rs9841778	4.80E-05	-0.29	Nerve - Tibial
CADM2		2.60E-08	0.3	Adipose - Subcutaneous
CADM2		6.80E-05	0.23	Adipose - Visceral (Omentum)
CADM2	rs9841829	2.80E-08	0.34	Lung
CADM2-AS1		7.70E-06	0.58	Brain - Caudate (basal ganglia)
CADM2-AS1		2.10E-08	0.66	Brain - Cerebellar Hemisphere
CADM2-AS1		2.60E-07	0.5	Brain - Cerebellum
CADM2-AS1		1.40E-05	0.49	Brain - Cortex
CADM2-AS1		3.60E-07	0.62	Brain - Frontal Cortex (BA9)
CADM2-AS1	rs9841973	7.50E-07	0.58	Brain - Nucleus accumbens (basal ganglia)

CADM2-AS1		3.80E-05	0.5	Brain - Cerebellar Hemisphere
CADM2-AS1	rs9842249	3.00E-05	0.48	Brain - Nucleus accumbens (basal ganglia)
CADM2-AS1		2.40E-06	0.54	Brain - Cerebellar Hemisphere
CADM2-AS1	rs9843797	1.30E-07	0.5	Brain - Cerebellum
CADM2		2.60E-06	-0.51	Heart - Left Ventricle
CADM2	rs9843953	6.50E-07	-0.32	Lung
CADM2		5.90E-08	0.28	Adipose - Subcutaneous
CADM2		6.80E-06	0.25	Adipose - Visceral (Omentum)
CADM2	rs9844512	4.70E-09	0.34	Lung
CADM2		3.20E-05	-0.45	Heart - Left Ventricle
CADM2	rs9846153	7.20E-08	-0.35	Lung
CADM2		4.10E-07	0.26	Adipose - Subcutaneous
CADM2		3.00E-05	0.23	Adipose - Visceral (Omentum)
CADM2	rs9846211	9.70E-09	0.33	Lung
CADM2-AS1	rs9846522	3.50E-05	-0.3	Nerve - Tibial
CADM2		9.80E-06	0.32	Brain - Hippocampus
CADM2	rs9846802	8.50E-05	0.14	Muscle - Skeletal
CADM2-AS1		7.50E-06	0.58	Brain - Caudate (basal ganglia)
CADM2-AS1		2.60E-08	0.66	Brain - Cerebellar Hemisphere
CADM2-AS1		3.60E-07	0.49	Brain - Cerebellum
CADM2-AS1		4.60E-06	0.51	Brain - Cortex
CADM2-AS1		3.70E-07	0.62	Brain - Frontal Cortex (BA9)
CADM2-AS1	rs9846878	5.80E-07	0.58	Brain - Nucleus accumbens (basal ganglia)
CADM2	rs9847448	1.70E-05	-0.19	Muscle - Skeletal
CADM2		6.20E-05	0.18	Adipose - Subcutaneous
CADM2		2.30E-06	0.58	Brain - Spinal cord (cervical c-1)
CADM2	rs9847516	1.50E-06	0.19	Muscle - Skeletal
CADM2	rs9847561	5.60E-06	-0.3	Lung
CADM2	rs9847870	1.40E-07	-0.35	Lung
CADM2		4.60E-08	-0.37	Lung
CADM2		3.30E-07	0.66	Brain - Spinal cord (cervical c-1)
CADM2	rs9848213	3.50E-05	0.17	Muscle - Skeletal
CADM2		1.10E-05	0.62	Brain - Spinal cord (cervical c-1)
CADM2	rs9849305	1.80E-06	0.18	Muscle - Skeletal
CADM2		7.20E-06	0.2	Adipose - Subcutaneous
CADM2		1.10E-08	0.71	Brain - Spinal cord (cervical c-1)
CADM2	rs9849768	1.50E-06	0.19	Muscle - Skeletal
CADM2-AS1		3.90E-05	0.53	Brain - Caudate (basal ganglia)
CADM2-AS1		3.10E-07	0.61	Brain - Cerebellar Hemisphere
CADM2-AS1		3.00E-07	0.49	Brain - Cerebellum
CADM2-AS1		3.00E-06	0.58	Brain - Frontal Cortex (BA9)
CADM2-AS1	rs9850482	1.20E-05	0.52	Brain - Nucleus accumbens (basal ganglia)
CADM2		5.90E-08	0.28	Adipose - Subcutaneous
CADM2		4.30E-06	0.26	Adipose - Visceral (Omentum)
CADM2	rs9851444	7.20E-09	0.34	Lung
CADM2-AS1		1.10E-05	0.59	Brain - Caudate (basal ganglia)
CADM2-AS1		1.20E-06	0.62	Brain - Cerebellar Hemisphere
CADM2-AS1		1.90E-06	0.48	Brain - Cerebellum
CADM2-AS1	rs9851502	1.80E-05	0.5	Brain - Cortex

CADM2-AS1		2.10E-06	0.64	Brain - Frontal Cortex (BA9)
CADM2-AS1		3.30E-06	0.58	Brain - Nucleus accumbens (basal ganglia)
CADM2-AS1	rs9852147	6.80E-05	-0.28	Nerve - Tibial
CADM2-AS1	rs9853144	8.10E-05	-0.36	Nerve - Tibial
CADM2	rs9853314	6.30E-08	-0.35	Lung
CADM2-AS1	rs9854293	1.80E-05	-0.41	Nerve - Tibial
CADM2		6.00E-07	0.27	Adipose - Subcutaneous
CADM2		1.50E-05	0.25	Adipose - Visceral (Omentum)
CADM2	rs9854869	2.50E-08	0.33	Lung
CADM2		1.30E-05	-0.46	Heart - Left Ventricle
CADM2	rs9854888	1.40E-06	-0.31	Lung
CADM2		3.70E-06	0.18	Adipose - Subcutaneous
CADM2	rs9855054	1.00E-05	0.16	Muscle - Skeletal
CADM2-AS1	rs9856337	5.00E-05	-0.29	Nerve - Tibial
CADM2-AS1	rs9857114	6.40E-05	-0.28	Nerve - Tibial
CADM2	rs9858244	1.30E-05	0.27	Lung
CADM2		2.70E-05	0.18	Adipose - Subcutaneous
CADM2		1.90E-05	0.26	Brain - Caudate (basal ganglia)
CADM2		4.60E-06	0.61	Brain - Spinal cord (cervical c-1)
CADM2	rs9860513	6.60E-07	0.19	Muscle - Skeletal
CADM2-AS1	rs9860698	3.50E-05	-0.39	Nerve - Tibial
CADM2-AS1		2.40E-06	0.54	Brain - Caudate (basal ganglia)
CADM2-AS1		2.40E-08	0.61	Brain - Cerebellar Hemisphere
CADM2-AS1		6.70E-10	0.55	Brain - Cerebellum
CADM2-AS1	rs9860792	2.60E-05	0.47	Brain - Nucleus accumbens (basal ganglia)
CADM2		1.00E-05	-0.46	Heart - Left Ventricle
CADM2	rs9861269	8.20E-06	-0.29	Lung
CADM2	rs986132	1.70E-04	0.13	Muscle - Skeletal
CADM2		1.50E-07	-0.23	Adipose - Subcutaneous
CADM2		9.70E-08	-0.26	Adipose - Visceral (Omentum)
CADM2		8.30E-06	-0.22	Breast - Mammary Tissue
CADM2	rs9861451	1.80E-09	-0.31	Lung
CADM2		8.60E-05	0.18	Adipose - Subcutaneous
CADM2		3.70E-07	0.65	Brain - Spinal cord (cervical c-1)
CADM2	rs9861497	2.90E-05	0.17	Muscle - Skeletal
CADM2-AS1		2.90E-07	0.58	Brain - Caudate (basal ganglia)
CADM2-AS1		3.00E-08	0.62	Brain - Cerebellar Hemisphere
CADM2-AS1		1.90E-10	0.56	Brain - Cerebellum
CADM2-AS1		1.10E-05	0.54	Brain - Frontal Cortex (BA9)
CADM2-AS1	rs9863150	1.90E-05	0.47	Brain - Nucleus accumbens (basal ganglia)
CADM2		1.70E-09	-0.26	Adipose - Subcutaneous
CADM2		4.10E-06	-0.22	Adipose - Visceral (Omentum)
CADM2		7.10E-07	-0.39	Heart - Left Ventricle
CADM2		1.30E-12	-0.34	Lung
CADM2	rs9863488	5.20E-05	-0.16	Muscle - Skeletal
CADM2		2.50E-08	0.26	Adipose - Subcutaneous
CADM2		1.80E-05	0.22	Adipose - Visceral (Omentum)
CADM2	rs9863620	8.50E-07	0.27	Lung
CADM2-AS1		3.90E-05	0.51	Brain - Caudate (basal ganglia)
CADM2-AS1	rs9864042	9.30E-06	0.52	Brain - Cerebellar Hemisphere

CADM2-AS1		3.60E-07	0.5	Brain - Cerebellum
CADM2	rs9864170	2.60E-06	-0.3	Lung
CADM2	rs9864651	1.10E-04	0.35	Muscle - Skeletal
CADM2		3.10E-08	0.25	Adipose - Subcutaneous
CADM2		3.80E-05	0.2	Adipose - Visceral (Omentum)
CADM2	rs9865191	2.60E-06	0.26	Lung
CADM2		2.20E-08	0.26	Adipose - Subcutaneous
CADM2		1.90E-05	0.22	Adipose - Visceral (Omentum)
CADM2	rs9865745	1.30E-06	0.27	Lung
CADM2		3.00E-07	0.24	Adipose - Subcutaneous
CADM2		4.90E-05	0.2	Adipose - Visceral (Omentum)
CADM2	rs9866089	8.00E-07	0.27	Lung
CADM2	rs9866182	1.50E-05	-0.74	Testis
CADM2-AS1		2.20E-05	0.56	Brain - Caudate (basal ganglia)
CADM2-AS1		5.10E-08	0.65	Brain - Cerebellar Hemisphere
CADM2-AS1		1.80E-07	0.51	Brain - Cerebellum
CADM2-AS1		4.50E-05	0.47	Brain - Cortex
CADM2-AS1		5.20E-07	0.62	Brain - Frontal Cortex (BA9)
CADM2-AS1	rs9866273	3.10E-06	0.55	Brain - Nucleus accumbens (basal ganglia)
CADM2		2.20E-07	0.28	Adipose - Subcutaneous
CADM2	rs9866322	1.90E-09	0.38	Lung
CADM2		6.30E-06	-0.21	Lung
CADM2-AS1	rs9866968	5.80E-05	0.26	Nerve - Tibial
CADM2-AS1	rs9867437	6.10E-05	0.24	Nerve - Tibial
CADM2		2.70E-08	0.27	Adipose - Subcutaneous
CADM2		1.90E-05	0.22	Adipose - Visceral (Omentum)
CADM2	rs9868427	1.20E-06	0.27	Lung
CADM2	rs9869121	2.30E-07	-0.34	Lung
CADM2-AS1	rs9869320	7.00E-05	-0.34	Nerve - Tibial
CADM2		6.50E-05	0.16	Adipose - Subcutaneous
CADM2	rs9869902	5.90E-07	0.18	Muscle - Skeletal
CADM2		1.70E-06	0.21	Adipose - Subcutaneous
CADM2	rs9870384	4.70E-05	0.15	Muscle - Skeletal
CADM2		1.10E-07	0.28	Adipose - Subcutaneous
CADM2		2.00E-05	0.25	Adipose - Visceral (Omentum)
CADM2	rs9870448	8.30E-09	0.34	Lung
CADM2	rs9871198	4.70E-05	0.15	Muscle - Skeletal
CADM2		8.80E-08	0.28	Adipose - Subcutaneous
CADM2		7.50E-06	0.25	Adipose - Visceral (Omentum)
CADM2	rs9873400	4.20E-09	0.34	Lung
CADM2-AS1	rs9873879	2.40E-05	-0.4	Nerve - Tibial
CADM2		5.00E-05	0.18	Adipose - Subcutaneous
CADM2		2.60E-05	0.26	Brain - Caudate (basal ganglia)
CADM2		7.80E-07	0.69	Brain - Spinal cord (cervical c-1)
CADM2	rs9874302	1.50E-05	0.17	Muscle - Skeletal
CADM2		1.90E-07	0.27	Adipose - Subcutaneous
CADM2		2.10E-05	0.24	Adipose - Visceral (Omentum)
CADM2	rs9874491	8.90E-09	0.34	Lung
CADM2		1.70E-08	0.27	Adipose - Subcutaneous
CADM2		2.10E-05	0.22	Adipose - Visceral (Omentum)
CADM2	rs9874740	1.20E-06	0.27	Lung
CADM2	rs9875381	1.40E-04	0.15	Adipose - Subcutaneous

CADM2		2.50E-05	0.15	Muscle - Skeletal
CADM2-AS1		2.70E-06	0.62	Brain - Caudate (basal ganglia)
CADM2-AS1		1.00E-07	0.66	Brain - Cerebellar Hemisphere
CADM2-AS1		1.30E-06	0.49	Brain - Cerebellum
CADM2-AS1		3.50E-05	0.49	Brain - Cortex
CADM2-AS1		3.60E-06	0.59	Brain - Frontal Cortex (BA9)
CADM2-AS1	rs9875386	2.40E-06	0.57	Brain - Nucleus accumbens (basal ganglia)
CADM2-AS1	rs9875880	3.70E-05	-0.34	Nerve - Tibial
CADM2		3.20E-05	0.17	Adipose - Subcutaneous
CADM2		5.00E-06	0.61	Brain - Spinal cord (cervical c-1)
CADM2	rs9876378	2.10E-06	0.17	Muscle - Skeletal
CADM2	rs9876406	1.60E-07	-0.35	Lung
CADM2-AS1		1.20E-05	0.51	Brain - Caudate (basal ganglia)
CADM2-AS1		5.50E-05	0.47	Brain - Cerebellar Hemisphere
CADM2-AS1		1.80E-07	0.5	Brain - Cerebellum
CADM2-AS1	rs9876664	3.20E-05	0.48	Brain - Nucleus accumbens (basal ganglia)
CADM2-AS1	rs9877447	7.50E-05	-0.37	Nerve - Tibial
CADM2		2.90E-05	0.26	Brain - Caudate (basal ganglia)
CADM2		1.50E-05	0.64	Brain - Spinal cord (cervical c-1)
CADM2	rs9877460	1.40E-05	0.17	Muscle - Skeletal
CADM2		3.20E-05	0.19	Adipose - Subcutaneous
CADM2		1.70E-06	0.59	Brain - Spinal cord (cervical c-1)
CADM2	rs9877971	5.50E-06	0.18	Muscle - Skeletal
CADM2	rs9878205	8.10E-05	-0.18	Muscle - Skeletal
CADM2		8.80E-06	0.32	Brain - Hippocampus
CADM2	rs9878472	5.80E-05	0.14	Muscle - Skeletal
CADM2	rs9878707	8.20E-05	0.17	Muscle - Skeletal
CADM2-AS1		3.80E-05	0.5	Brain - Cerebellar Hemisphere
CADM2-AS1	rs9878876	3.10E-05	0.48	Brain - Nucleus accumbens (basal ganglia)
CADM2		5.30E-08	0.25	Adipose - Subcutaneous
CADM2		4.00E-05	0.21	Adipose - Visceral (Omentum)
CADM2	rs9879025	4.50E-07	0.28	Lung
CADM2-AS1		1.20E-06	-0.59	Brain - Caudate (basal ganglia)
CADM2-AS1		2.30E-09	-0.69	Brain - Cerebellar Hemisphere
CADM2-AS1		8.50E-12	-0.64	Brain - Cerebellum
CADM2-AS1		1.50E-05	-0.54	Brain - Frontal Cortex (BA9)
CADM2-AS1		2.90E-05	-0.56	Brain - Hippocampus
CADM2-AS1	rs9879903	1.80E-07	-0.6	Brain - Nucleus accumbens (basal ganglia)
CADM2		9.20E-05	0.2	Adipose - Subcutaneous
CADM2		2.70E-05	0.24	Lung
CADM2-AS1	rs9880010	1.40E-04	-0.28	Nerve - Tibial
CADM2		1.30E-04	0.2	Adipose - Subcutaneous
CADM2		8.00E-05	0.23	Adipose - Visceral (Omentum)
CADM2	rs9880272	2.00E-06	0.29	Lung
CADM2		2.60E-08	0.26	Adipose - Subcutaneous
CADM2		1.80E-05	0.22	Adipose - Visceral (Omentum)
CADM2	rs9880919	4.90E-07	0.28	Lung
CADM2		4.50E-05	0.19	Adipose - Subcutaneous
CADM2		2.10E-06	0.59	Brain - Spinal cord (cervical c-1)
CADM2	rs9880953	3.80E-06	0.19	Muscle - Skeletal

CADM2		1.20E-05	0.19	Adipose - Subcutaneous
CADM2		1.90E-05	0.26	Brain - Caudate (basal ganglia)
CADM2		3.90E-06	0.62	Brain - Spinal cord (cervical c-1)
CADM2	rs9881257	5.30E-07	0.19	Muscle - Skeletal
CADM2-AS1		4.80E-06	0.53	Brain - Cerebellar Hemisphere
CADM2-AS1	rs9881886	1.70E-07	0.49	Brain - Cerebellum
CADM2	rs9881963	1.60E-05	-0.19	Muscle - Skeletal
CADM2-AS1	rs9882158	7.30E-05	-0.28	Nerve - Tibial
CADM2		3.80E-05	0.19	Adipose - Subcutaneous
CADM2		4.50E-07	0.64	Brain - Spinal cord (cervical c-1)
CADM2	rs9882516	2.80E-05	0.17	Muscle - Skeletal
CADM2		7.70E-05	0.15	Adipose - Subcutaneous
CADM2	rs9883252	9.90E-06	0.15	Muscle - Skeletal
CADM2		7.20E-05	0.18	Adipose - Subcutaneous
CADM2		2.40E-05	0.27	Brain - Caudate (basal ganglia)
CADM2		1.00E-06	0.69	Brain - Spinal cord (cervical c-1)
CADM2	rs9883443	2.20E-05	0.17	Muscle - Skeletal
CADM2		3.90E-05	0.19	Adipose - Subcutaneous
CADM2		4.90E-07	0.65	Brain - Spinal cord (cervical c-1)
CADM2	rs9883729	4.10E-05	0.17	Muscle - Skeletal
CADM2	rs9883919	1.90E-05	-0.2	Lung
CADM2		8.70E-06	-0.47	Heart - Left Ventricle
CADM2	rs993135	1.50E-06	-0.31	Lung
CADM2		4.00E-11	-0.28	Adipose - Subcutaneous
CADM2		1.20E-07	-0.24	Adipose - Visceral (Omentum)
CADM2		3.60E-07	-0.4	Heart - Left Ventricle
CADM2	rs993136	1.50E-15	-0.38	Lung
CADM2		4.80E-10	-0.26	Adipose - Subcutaneous
CADM2		2.80E-07	-0.24	Adipose - Visceral (Omentum)
CADM2		5.30E-07	-0.41	Heart - Left Ventricle
CADM2		5.50E-17	-0.4	Lung
CADM2	rs993137	1.30E-04	-0.15	Muscle - Skeletal
CADM2		8.00E-12	-0.29	Adipose - Subcutaneous
CADM2		7.80E-08	-0.25	Adipose - Visceral (Omentum)
CADM2		2.40E-07	-0.4	Heart - Left Ventricle
CADM2		3.40E-15	-0.38	Lung
CADM2	rs993716	1.30E-04	-0.15	Muscle - Skeletal
CADM2-AS1		4.40E-05	0.54	Brain - Caudate (basal ganglia)
CADM2-AS1		8.70E-07	0.6	Brain - Cerebellar Hemisphere
CADM2-AS1		7.00E-09	0.53	Brain - Cerebellum
CADM2-AS1		2.50E-05	0.46	Brain - Cortex
CADM2-AS1		2.00E-05	0.53	Brain - Frontal Cortex (BA9)
CADM2-AS1	rs9985228	4.80E-07	0.59	Brain - Nucleus accumbens (basal ganglia)
CADM2-AS1		8.70E-07	0.6	Brain - Cerebellar Hemisphere
CADM2-AS1		9.90E-09	0.52	Brain - Cerebellum
CADM2-AS1		2.10E-05	0.53	Brain - Frontal Cortex (BA9)
CADM2-AS1	rs9985441	4.80E-07	0.59	Brain - Nucleus accumbens (basal ganglia)
CADM2	rs9990095	1.50E-05	-0.74	Testis
CADM2		2.70E-05	-0.18	Adipose - Subcutaneous
CADM2		1.20E-05	-0.2	Adipose - Visceral (Omentum)
CADM2	rs9990096	4.80E-07	-0.25	Lung

CADM2-AS1	rs9990223	2.30E-05	-0.41	Nerve - Tibial

Supplementary Table 4: Associations of published CADM2 SNPs with phenotypes reported in this study

Previously reported							UKB		UKB Risk-taking (n=326432)			UKB mood instability (n=391271)			UKB neuroticism score (n=326191)			Meta-analysis CRP (N=14799)					meta-analysis BMI (N=416136)					Meta-analysis SBP (N=397163)			
PMID	DISEASE/TRAIT	POS	STRONGEST SNI	RAF	Effect	P-VALUE	A1	A1F	BETA	SE	P	BETA	SE	P_moodinst	BETA	SE	P_neuro	A1	A1F	BETA	SE	P	A1	A1F	BETA	SE	P	A1F	BETA	SE	P
25869804	Information processing speed	18991328	rs664154-A	0.24	5.03	5.00E-07																									
29221444	Coronary artery calcified atherosclerotic plaque (130 HU threshold) in type 2 diabetes	84956870	rs450495-?	NR	0.17	3.00E-06	T	0.08	0.00	0.01	0.8674	-0.01	0.01	0.2853	-0.03	0.01	0.0627						T	0.08	-0.01	0.02	0.7266	0.08	0.03	0.08	0.7002
29899525	Vigorous physical activity	84966628	rs1248860-?	0.52	1.04167	**1.00E-13**	G	0.49	-0.0234	0.0058	5.51E-05	0.0118	0.0046	0.0103	0.025	0.0079	0.0016	a	0.50	0.013	0.014	0.3484	A	0.51	0.03	0.010	1.34E-03	0.51	-0.08	0.024	9.52E-04
29899525	Moderate to vigorous physical activity levels	85007370	rs2035562-A	0.33	0.014	**4.00E-09**	A	0.33	-0.015	0.0062	0.0154	0.0093	0.0049	0.0583	0.0213	0.0085	0.0120	a	0.34	0.005	0.016	0.7492	A	0.33	-0.05	0.010	1.43E-05	0.33	0.08	0.026	2.19E-03
28937693	Alcohol consumption	85187275	rs1376935-A	0.32	0.03	**4.00E-10**	A	0.31	0.02	0.01	0.0003	-0.02	0.00	0.0001	-0.03	0.01	0.0002	A	0.31	0.00	0.02	0.8179	A	0.31	0.04	0.01	1.54E-04	0.31	-0.07	0.03	0.0044
28937693	Alcohol consumption in current drinkers	85215116	rs13078384-A	0.31	0.02	**2.00E-09**	A	0.31	0.02	0.01	0.0003	-0.02	0.00	0.0003	-0.03	0.01	6.68E-05	A	0.31	-0.01	0.02	0.4844	A	0.31	0.04	0.01	6.74E-05	0.31	-0.07	0.02	0.0076
28937693	Alcohol consumption	85345622	rs67028245-A	0.4	0.02	**4.00E-08**	A	0.40	-0.03	0.01	2.19E-05	0.01	0.00	0.0187	0.03	0.01	5.30E-05						A	0.40	-0.04	0.01	4.39E-05	0.40	0.17	0.04	9.43E-05
29326435	Intelligence (multi-trait analysis)	85347968	rs9830359-T	NR	0.03	**4.00E-08**	T	0.11	0.03	0.01	0.0005	0.01	0.01	0.0601	0.00	0.01	0.7644						T	0.11	0.02	0.02	0.2071	0.11	-0.18	0.07	0.0082
29899525	Strenuous sports or other exercises	85351651	rs62253088-T	0.33	1.05	**1.00E-19**	T	0.33	**0.034**	**0.0062**	**3.23E-08**	-0.0171	0.0049	0.0005	**-0.0376**	**0.0084**	**8.32E-06**						T	0.33	0.04	0.011	1.75E-04	0.33	-0.14	0.046	2.65E-03
22589738	Visceral fat	85453695	rs13323436-A	0.1		3.00E-06	A	0.08	**0.05**	**0.01**	**1.04E-06**	-0.01	0.01	0.1166	-0.03	0.01	0.0657						A	0.08	0.07	0.02	4.23E-04	0.08	-0.06	0.08	0.4363
25199915	Longevity (90 years and older)	85482049	rs9841144-?	0.21	1.23	9.00E-07	T	0.23	**0.05**	**0.01**	**4.67E-13**	-0.02	0.01	0.0041	-0.04	0.01	2.02E-05	A	0.78	-0.01	0.02	0.4685	A	**0.78**	**-0.07**	**0.01**	**1.09E-08**	0.79	0.05	0.03	0.0895
29391395	Self-reported risk-taking behaviour	85519876	rs62250759-G	NR	-0.06	**5.00E-12**	G	0.37	**-0.05**	**0.01**	**7.24E-15**	0.00	0.00	0.3959	0.01	0.01	0.1814						A	**0.63**	**0.07**	**0.01**	**5.44E-12**	0.63	-0.01	0.04	0.8642
28937693	Alcohol consumption	85520211	rs9841829-G	0.23	0.02	**3.00E-10**	G	0.23	**0.05**	**0.01**	**3.94E-13**	-0.02	0.01	0.0037	-0.04	0.01	2.11E-05	T	0.78	-0.01	0.02	0.4629	T	**0.78**	**-0.07**	**0.01**	**1.96E-08**	0.79	0.05	0.03	0.0771
25644384	Cognitive function	85555773	rs17518584-?	NR	0.03	1.00E-06	C	0.37	**0.05**	**0.01**	**1.11E-17**	-0.01	0.00	0.1315	-0.03	0.01	5.84E-05	T	0.65	-0.03	0.01	0.0511	T	**0.63**	**-0.06**	**0.01**	**6.70E-11**	0.65	0.06	0.02	0.0095
25869804	Information processing speed	85555773	rs17518584-T	0.64	5.92	**3.00E-09**																									
27046643	Educational attainment	85622526	rs55686445-?	NR	0.03	**5.00E-12**	C	0.35	**-0.04**	**0.01**	**7.32E-13**	-0.01	0.00	0.1201	0.00	0.01	0.9205	T	0.63	0.06	0.01	1.07E-04	T	**0.64**	**0.09**	**0.01**	**3.97E-18**	0.63	0.05	0.02	0.0333
27225129	Educational attainment (years of education)	85625640	rs62263923-A	0.639	0.02	**1.00E-13**	G	0.35	**-0.04**	**0.01**	**1.43E-12**	-0.01	0.00	0.1451	0.00	0.01	0.8233	A	0.63	0.06	0.01	1.03E-04	A	**0.64**	**0.09**	**0.01**	**3.31E-18**	0.62	0.05	0.02	0.0344
29326435	Intelligence (multi-trait analysis)	85750927	rs10511071-T	NR	0.03	**1.00E-08**	C	0.11	**-0.05**	**0.01**	**1.32E-06**	-0.01	0.01	0.0840	0.00	0.01	0.7210	T	0.89	0.03	0.02	0.1807	T	**0.89**	**0.09**	**0.02**	**2.97E-08**	0.89	0.01	0.04	0.8674
28443625	Body mass index (joint analysis main effects and smoking interaction)	85758440	rs13078960-T	0.807		**3.00E-09**																									
28443625	BMI (adjusted for smoking behaviour)	85758440	rs13078960-T	0.807	0.03	**1.00E-10**																									
28443625	BMI in non-smokers	85758440	rs13078960-T	0.807	0.02	1.00E-06																									
25673413	Body mass index	85758440	rs13078960-G	0.193	0.03	**1.00E-14**	G	0.20	0.03	0.01	5.79E-05	-0.01	0.01	0.0114	-0.03	0.01	0.0032	T	0.81	-0.01	0.02	0.6218	T	**0.80**	**-0.09**	**0.01**	**2.63E-13**	0.82	0.08	0.03	0.0085
25673412	Waist circumference	85770262	rs2325036-A	0.604	0.02	**2.00E-11**	C	0.38	**-0.03**	**0.01**	**8.34E-07**	-0.01	0.00	0.1800	0.00	0.01	0.9851	A	0.61	0.06	0.01	2.43E-05	A	**0.62**	**0.08**	**0.01**	**1.72E-17**	0.60	0.04	0.02	0.0658
25673412	Hip circumference	85771031	rs13098327-A	0.196	0.03	**1.00E-09**	A	0.20	0.03	0.01	9.21E-05	-0.01	0.01	0.0094	-0.03	0.01	0.0032	A	0.19	0.01	0.02	0.6474	A	**0.20**	**0.09**	**0.01**	**3.22E-13**	0.18	-0.07	0.03	0.0093
28448500	Body mass index (joint analysis main effects and physical activity interaction)	85777586	rs9852127-?	NR		**7.00E-09**	A	0.20	0.03	0.01	8.93E-05	-0.01	0.01	0.0094	-0.03	0.01	0.0032	A	0.19	0.01	0.02	0.6495	A	**0.20**	**0.09**	**0.01**	**3.19E-13**	0.18	-0.07	0.03	0.0093
28448500	Body mass index	85777586	rs9852127-A	0.187	0.03	**2.00E-09**																									
28448500	Body mass index in physically active individuals	85777586	rs9852127-A	0.187	0.03	1.00E-07																									
28448500	Body mass index	85793191	rs9852859-C	0.191	0.03	**2.00E-09**	C	0.20	0.03	0.01	8.47E-05	-0.01	0.01	0.0090	-0.03	0.01	0.0031	t	0.81	-0.01	0.02	0.6673	T	**0.80**	**-0.09**	**0.01**	**2.94E-13**	0.82	0.07	0.03	0.0090
28448500	Body mass index in physically active individuals	85811914	rs9818122-C	0.203	0.03	**9.00E-08**	C	0.21	0.03	0.01	3.74E-05	-0.01	0.01	0.0082	-0.03	0.01	0.0042	T	0.80	-0.01	0.02	0.4846	T	**0.80**	**-0.09**	**0.01**	**6.39E-14**	0.81	0.08	0.03	0.0038
22832960	Temperament	85825326	rs12494658-T	0.752	0.08	5.00E-06	C	0.24	-0.01	0.01	0.1867	0.00	0.01	0.8289	0.01	0.01	0.4890	T	0.76	0.06	0.02	3.88E-04	T	**0.76**	**0.06**	**0.01**	**1.64E-08**	0.75	0.05	0.03	0.0599
23563607	Obesity	85835000	rs13078807-G	0.2	1.06	**3.00E-11**	G	0.20	0.03	0.01	9.28E-05	-0.02	0.01	0.0074	-0.03	0.01	0.0035	A	0.81	-0.01	0.02	0.7238	A	**0.80**	**-0.09**	**0.01**	**4.11E-13**	0.82	0.07	0.03	0.0118
20935630	Body mass index	85835000	rs13078807-G	0.2	0.10	**4.00E-11**																									
28892062	Body mass index	85836927	rs12495178-C	0.37	0.01	**3.00E-09**	C	0.35	**-0.03**	**0.01**	**2.77E-06**	0.00	0.00	0.3685	0.01	0.01	0.4718	T	0.64	0.06	0.01	1.40E-05	T	**0.64**	**0.09**	**0.01**	**1.52E-18**	0.62	0.04	0.02	0.0551
25778476	Alzheimer's disease in APOE e4+ carriers	85855884	rs71316816-C	0.92	1.25	9.00E-06	T	0.07	0.02	0.01	0.1379	-0.02	0.01	0.0854	-0.02	0.02	0.2767	T	0.07	0.02	0.03	0.4992	T	0.07	0.09	0.02	2.04E-06	0.06	-0.11	0.05	0.0226
27046643	Educational attainment	85897356	rs112374913-?	NR	0.03	**3.00E-09**	A	0.40	-0.02	0.01	0.0026	-0.01	0.00	0.0290	0.00	0.01	0.8902						A	**0.40**	**-0.06**	**0.01**	**3.75E-08**	0.40	-0.03	0.04	0.5747
22589738	Subcutaneous adipose tissue	86109735	rs2324999-T	0.2		2.00E-06	T	0.20	0.03	0.01	0.0003	0.00	0.01	0.5995	-0.02	0.01	0.0515	T	0.21	0.03	0.02	0.0728	T	0.20	0.06	0.01	6.50E-08	0.21	0.01	0.03	0.7616
27225129	Educational attainment (years of education)	86134566	rs56262138-A	0.298	0.01	**2.00E-08**	A	0.30	-0.03	0.01	2.98E-05	-0.01	0.01	0.0375	0.00	0.01	0.6574						A	**0.30**	**-0.08**	**0.01**	**3.74E-11**	0.30	0.04	0.05	0.4278
25869804	Information processing speed	113415768	rs2734839-T	0.61	4.91	9.00E-07																									
25869804	Information processing speed	222235804	rs2118666-T	0.46	4.95	7.00E-07																									
